# T‐Shaped Stibenium(III) Cation: Hydrostibination Without Sb─H Bond

**DOI:** 10.1002/anie.7924667

**Published:** 2026-07-11

**Authors:** Ekta Nag, Lars Ole Busse, Andreas Albers, Manuel Schmitt, Lutz Greb

**Affiliations:** ^1^ Anorganisch‐Chemisches Institut Ruprecht‐Karls‐Universität Heidelberg Heidelberg Germany

**Keywords:** *anti*‐Markovnikov, antimony, element–ligand cooperativity, hydroelementation, hydrostibination

## Abstract

The hydrostibination of alkenes represents a largely underdeveloped transformation, owing to the intrinsic lability of the required Sb─H reagents. We now show that an Sb─H bond is not needed. The structurally constrained, amidophenolato‐pyridyl supported T‐shaped stibenium(III) ion undergoes *anti*‐Markovnikov hydrostibination of a broad range of alkenes in excellent yields. Spectroscopic and computational analyses reveal a polarizable, redox‐confused Sb–*π* system in the cation, whereas its reactivity follows closed‐shell ionic pathways characteristic of a Lewis‐acidic Sb(III) center. Mechanistic studies support element–ligand cooperativity (ELC) with the hydride equivalent for hydrostibination originating from the ligand scaffold after substrate activation at antimony. The resulting stiba‐alkanes can be transformed quantitatively into haloalkanes. This work establishes a viable strategy for hydroelementation with heavy *p*‐block elements while circumventing weak and labile E─H bonds.

## Graphical abstract

A cationic, structurally constrained stibenium platform mediates *anti*‐Markovnikov hydrostibination of alkenes via element–ligand cooperativity (ELC), thereby bypassing the need for weak Sb─H bonds.

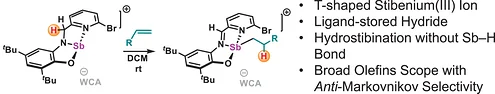

## 1 Introduction

The addition of element hydrides (E–H) across carbon–carbon double bonds, commonly termed hydroelementation, represents one of the most atom‐economic strategies for E─C bond formation. Particularly when applied to abundant olefin feedstocks, hydroelementation may provide direct access to reactive E─C motifs for rich downstream diversification. Since the seminal discovery of hydroboration by Brown and coworkers [1, 2, 3, 4], hydroelementation chemistry has expanded far beyond polarized (semi)metal hydrides [5, 6, 7, 8, 9, 10, 11], encompassing a wide range of main‐group E─H bonds [12, 13, 14, 15, 16, 17, 18, 19, 20, 21, 22, 23]. While extensive progress has been made for the lighter p‐block elements, hydroelementation chemistry with the heavier elements, in particular the pnictogens (E = Sb, Bi), remains far less developed. This imbalance largely reflects the properties of the required E─H bonds, which are relatively weak and labile with respect to dehydrogenative decomposition.

**FIGURE 1:**
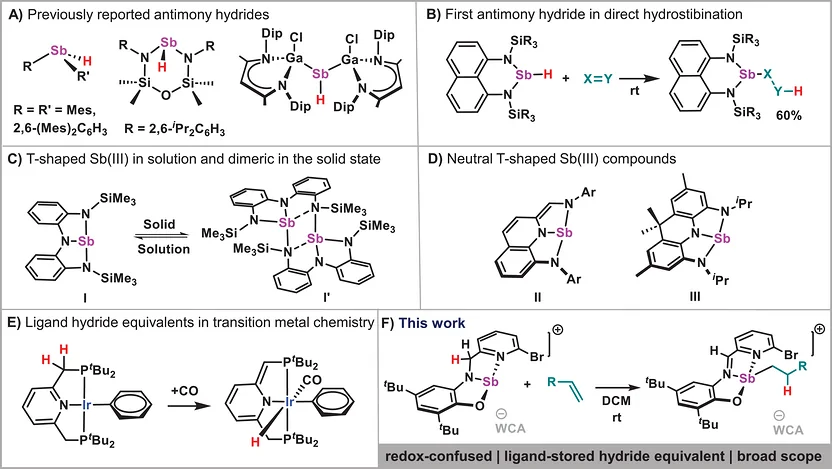
(A) Examples of previously reported antimony hydrides. (B) Example of catalyst‐free hydrostibination from antimony hydride. (C) Planarization of Sb(III) compound in solution and the pyramidal structure in solid‐state. (D) Isolated neutral T‐shaped Sb(III) compounds. (E) Transition metals in bond activation via metal–ligand cooperativity. (F) Hydrostibination of alkenes without the Sb─H bond is reported herein.

Although several stable heavy pnictogen hydrides have been reported (Figure 1A for representative examples with Sb) [22, 24, 25], such species are usually prone to thermal or photochemical dehydrocoupling, leading to E─E bond formation or precipitation of elemental antimony/bismuth [26, 27, 28, 29, 30]. Accordingly, E─H compounds capable of selective hydroelementation reactions are exceedingly rare [31, 32, 33]. To date, only a sole example of hydrostibination without transition‐metal catalysts, Lewis acids, or radical initiators has been demonstrated (Figure 1B) [34]. An elegant diamino‐naphthalene‐supported antimony hydride enabled the *anti*‐Markovnikov hydrostibination of an electron‐deficient alkene and azobenzene, while less efficient addition was observed with more electron‐rich olefins. The hydrostibination of terminal alkynes with the same reagent was shown to proceed via a radical mechanism [35]. Notably, organoantimony compounds are regaining attention as strategic intermediates for selective organic synthesis [36]. Despite this potential, their broader exploration remains limited by the narrow synthetic access to Sb─C bonds.

An emerging strategy to expand the reactivity of p‐block compounds is the deliberate imposition of structural constraints [37, 38]. Enforcing a T‐shape at naturally pyramidal pnictogen(III) centers profoundly alters frontier orbital energetics, enhances Lewis's acidity and ambiphilicity, and enables the activation of O─H or N─H bonds and H_2_ [39, 40, 41, 42].

Marczenko and coworkers demonstrated that NNN‐pincer ligands can induce pronounced deformation at Sb(III), yielding species **I** that are planar in solution yet dimeric in the solid‐state (Figure 1C, **I′**) [43]. T‐shaped Sb(III) species that are monomeric also in the solid‐state have been isolated later on by Kim (**II**) [44] or Deuter (**III**) [45], showcasing remarkable photophysical properties or reactivity toward dioxygen (Figure 1D). Beyond these studies, antimony chemistry with and without structural constraints is recently gaining further attention, with advances spanning electromerism, redox cycling and alternative bond‐activation processes [46, 47, 48, 49, 50, 51, 52].

A complementary approach for the expanded reactivity of p‐block elements is element–ligand cooperativity (ELC), wherein the ligand framework actively participates in respective transformations [53]. While examples of antimony–ligand cooperativity have been documented [54, 55, 56], the integration of latent ligand‐centered hydride equivalents, analogous to transition‐metal systems (Figure 1E) [57, 58, 59, 60, 61, 62], remains unexplored. Importantly, such an approach would decouple hydroelementation reactivity from the presence of E─H bonds, thereby circumventing the lability of heavy pnictogen hydrides.

Building on our work on amidophenolato‐pyridyl supported phosphenium ions, which activate inert C─H bonds [63], we sought to transfer this system to antimony. Herein, we report the synthesis of the first planar, T‐shaped stibenium(III) ion (Figure 1F). By combining structural constraint with a ligand‐embedded hydride equivalent, the system enables hydrostibination of alkenes without the need for an antimony‐hydrogen bond. The strategy affords *anti*‐Markovnikov hydrostibination across a broad substrate scope—including styrenes, aliphatic alkenes, dienes, and strained bicyclic systems—delivering stiba‐alkane products quantitatively.

## 2 Results and Discussion

**FIGURE 2:**
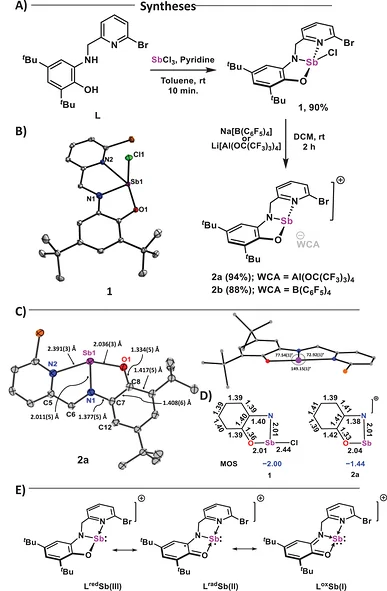
(A) Synthesis of stibenium salts **2a** and **2b** [L‐Sb][WCA]. (B, C) Molecular structures of **1** and **2a**. Thermal ellipsoids are set at a 50% probability level. The counteranion [Al(OC(CF_3_)_3_)_4_]^−^ in **2a** and all carbon‐based hydrogen atoms have been omitted for clarity. Selected bond distances (Å) and angles (deg) of **1**: d(Sb1‐O1) = 2.018(3), d(Sb1‐N2) = 2.020(4), d(Sb1‐N1) = 2.506(4), d(O1‐C8) = 1.374(6), d(N2‐C7) = 1.398(6), ∠O1‐Sb1‐N2 = 80.02(1), ∠N2‐Sb1‐N1 = 70.90(1), ∠O1‐Sb1‐N1 = 150.92(1), and **2a**: d(Sb1‐N1) = 2.011(5), d(Sb1‐O1) = 2.036(3), d(Sb1‐N2) = 2.391(4), d(O1‐C8) = 1.334(5), d(N1‐C7) = 1.377(5), ∠N1‐Sb1–O1 = 77.54(1), ∠N1‐Sb1–N2 = 72.92(1), ∠O1‐Sb1–N2 = 149.15(1). (D) Relevant XRD metric parameters for determination of metrical oxidation states (MOS, blue numbers) of **1** and **2a**. (E) Possible resonance structures of **2a**.

The amidophenolato‐pyridyl ligand **L** was prepared by reductive amination of the commercially available 4‐bromopyridine‐2‐carboxaldehyde and 3,5‐di‐*tert*‐butyl aminophenol on a multigram scale [64]. Treatment of **L** with SbCl_3_ in the presence of pyridine in toluene at rt resulted in the formation of chlorostibane **1** as an orange crystalline solid in 90% yield (Figure 2A). Single crystals suitable for x‐ray diffraction of **1** were grown by vapor diffusion of *n*‐pentane into a concentrated DCM solution of **1** at rt, showing expected structural features (Figure 2B).

Treatment of **1** with 1.1 equiv. of chloride abstracting agents Li[Al(OC(CF_3_)_3_)_4_] or Na[B(C_6_F_5_)_4_] in DCM at rt yielded the desired stibenium salts **2a/b** [L‐Sb][WCA] (WCA = Al(OC(CF_3_)_3_)_4_ for **2a** and B(C_6_F_5_)_4_ for **2b**) as dark‐red solids in excellent yields (**2a** (94%) and **2b** (88%)). While ^1^H NMR spectroscopy of the chlorido adduct **1** shows the ligand methylene (─CH_2_) singlet at *δ* = 5.27 ppm in CD_2_Cl_2_, it becomes significantly deshielded to *δ* = 6.56–6.58 ppm in **2a/b**. Compounds **2a/b** are only sparingly soluble in nonpolar solvents (benzene, toluene) but completely dissolve in more polar solvents (DCM, ACN, and *o*‐DFB). They exhibit sensitivity to air and moisture but are stable in the solid‐state and halogenated solvents at rt for months when stored under an inert atmosphere. Heating solutions of **2a/b** in DCM above 70°C leads to partial ligand oxidation under participation of the methylene hydride (compound **4**, Figure 4C and Scheme S2), along with the deposition of elemental antimony. This observation indicated the potential redox involvement of the ligand, as it will become of relevance at a later stage. Dark‐red single crystals suitable for x‐ray diffraction of **2a** were grown via layering of a concentrated solution in DCM with *n*‐hexane at −40 °C (Figure 2C). The Sb1–N1 (2.011(5) Å) and Sb1–O1 (2.036(3) Å) bond lengths in **2a** match the sum of covalent radii (Σ_cov_(N–Sb) = 2.11 Å / Σ_cov_(O–Sb) = 2.05 Å) [65], and are comparable to the reported Sb─N in planar compound **II** (Figure 1D) (2.070(2) Å) [44] or in a donor‐free stibenium(III) ion (1.993(4) Å) [66]. The pyridine nitrogen N_2_ coordinates to Sb with a bond length of 2.391(4) Å, which is significantly shorter than the intermolecular dative interaction observed in the dimeric **I′** (2.633(4) Å, Figure 1C) [43]. Most interestingly, **2a** features an antimony atom with nearly planar T‐shaped coordination geometry [N1–Sb1–O1–N2 dihedral angle = 174.55(1)], consistent with the neutral T‐shaped **II** (∠N1–Sb1–N2–N3) = 179.73° [44]. While neutral **I** dimerizes in the solid‐state [43], and **II** is sterically protected from dimerization, Coulomb repulsion can be made responsible for preventing such aggregation in the case of cationic **2a**. The observed planarity of **2a** contrasts the bent structure of the phosphorus congener [63] but can be explained by similar arguments as discussed previously [43, 67]. The local T‐shape is hinting to the presence of five stereochemically active electron pairs at antimony (three bonding, two nonbonding), suggesting a partially oxidized ligand and reduced Sb center (Figure 2E). Indeed, the O1–C8 (1.335(5)Å) and the N1–C7 (1.377(5) Å) bond length in **2a** is significantly shortened in comparison to **1** (1.374(6) Å/1.398(6) Å). Accordingly, the calculated metrical oxidation state (MOS) for the amidophenolate moiety in **2a** (−1.44) is increased compared to **1** (−2.00; Figure 2D), supporting a ligand‐to‐metal charge transfer [68].

**FIGURE 3:**
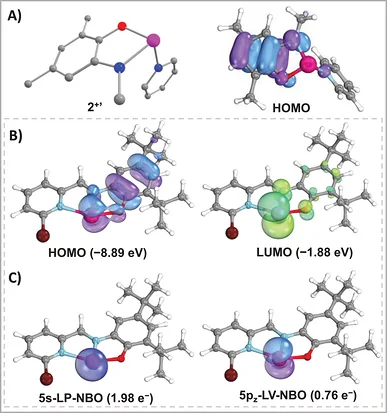
(A) Computed structure and HOMO of unrestricted stibenium ion (**2^+^′**). (B) HOMO and LUMO of **2^+^. (**C) LP‐*s*‐type and LV‐*p*‐type NBO of **2^+^**.

Ambiguities in assigning the oxidation state of planarized heavier pnictogen compounds supported by rigid NNN pincer ligands have been the subject of debate in the past, with interpretations ranging from E(I) and E(III) oxidation state over biradicaloid character [69, 70, 71]. Hence, the electronic structure of the stibenium ion (**2^+^**) in **2a** was evaluated by computational methods. Restricted, scalar‐relativistic DFT (X2C‐ωB97‐D4/X2C‐TZVPPall) optimized geometrical parameters are matching well with the solid‐state structure (Table S9). For comparison, we also computed the structure of a related unconstrained stibenium ion **2^+^′** (Figure 3A), which exhibits a global minimum with pyramidal geometry around Sb (∠O–Sb–N1 = 83.59°; ∠O–Sb–N2 = 90.61°, and ∠N1–Sb–N2 = 94.11°). This comparison indicates that the unusual T‐shaped geometry arises from the interplay of structural constraint and ligand redox non‐innocence. The HOMO of **2^+^′** is entirely located at the amidophenolate moiety (Figure 3A). In contrast, the HOMO of **2^+^** is localized on the ligand framework and at Sb (Figure 3B). Natural bond orbital (NBO) analysis of **2^+^** reveals an *s*‐type lone pair (LP) at Sb (88.11% *s* and 11.85% *p*, 1.98 e^−^), along with a partially occupied (0.76 e^−^) *p*‐type lone vacancy (LV), confirming substantial charge transfer from the electron‐rich amidophenolate part toward antimony (Figure 3C). This feature is lacking for **2^+^′** (Section S8). In addition, the Wiberg bond index for the C─N bond in **2^+^** (1.29) is larger as in **1** (1.084) and **2^+^′** (1.085), corroborating structural constraints and the low coordination state at antimony as a requirement for this delocalization. Remarkably, despite its cationic nature, the NPA charge at Sb in **2^+^** is lower (+1.53) compared to **1** (+1.67) and **2^+^′** (+1.92). The triplet state of **2^+^** is 17.8 kcal mol^−1^ above the restricted closed‐shell solution, in line with sharp ^1^H NMR signals and the absence of any EPR activity. However, fractional occupation density (FOD = 0.6, see Section S8) indicated non‐negligible static electron correlation and the possible involvement of an open‐shell singlet state [72, 73]. Thus, CASSCF computations were performed on **2^+^**. State‐specific CASSCF(10/9) revealed that the ground state can be adequately described by a single configuration state function ($c_{0}^{2}=84\%$). The contribution of the double excited configuration is minor ($c_{d}^{2}=4.1\%$), indicating a small diradical character of $\beta =2{\;c}_{d}^{2}=8.3\%$, which can be attributed to dynamic electron correlation effects rather than an open‐shell singlet state [74, 75, 76]. To get further insights into the electronic structure, UV/Vis spectra of **2a** were recorded in DCM solution (Figure S91), showing a well‐resolved, intense band at 490 nm, in line with the deep red color, and weaker bands at 303 and 258 nm. TDDFT + NTO analysis and CASSCF computations allowed to assign the lowest energy band as a *π*→π* transition (HOMO → LUMO). Assignment of the next bands was more ambiguous and remained inconclusive (see Section S8). Overall, this analysis confirmed the partially reduced, redox‐confused nature of antimony [69], exemplified by the increased MOS of the ligand and the lowered NPA charge at antimony.

**FIGURE 4:**
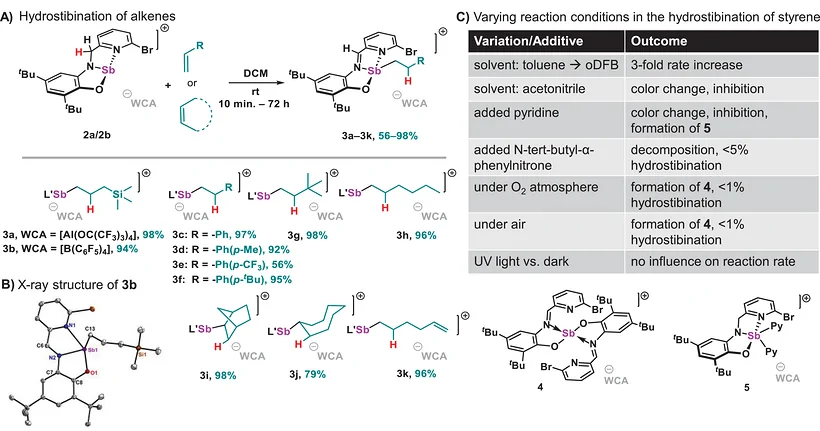
(A) Hydrostibination of alkenes. Isolated yields of products are given. (B) Molecular structure of **3b**. Thermal ellipsoid plot of **3b** at 50% probability. All hydrogen atoms and the counter anion [B(C_6_F_5_)_4_]^−^ were omitted for clarity. Selected bond distances (Å) and angles (deg) of **3b**: d(Sb1–O1) = 2.0572(12), d(Sb1–N2) = 2.1824(14); d(Sb1–N1) = 2.3985(14); d(O1–C8) = 1.3378(19), d(N2–C7 = 1.402(2); ∠O1–Sb1–N2 = 76.01(5), ∠N2–Sb1–N1 = 70.36(5), ∠O1–Sb1–N1 = 145.35(5). (C) Varying reaction conditions and additives were probed during the hydrostibination of styrene, with the observed outcome.

Next, the reactivity of these species was investigated. Treating salts **2a/b** with Lewis acids (CuCl/W(CO)_6_, B(C_6_F_5_)_3_), potentially binding to antimony, did not induce notable spectral changes, consistent with predominant electrophilic rather than Lewis basic behavior at Sb. By consequence, the reactivity with nucleophilic, unsaturated organic substrates was tested (Figure 4A). Compound **2b** was treated with 3‐(trimethylsilyl)propene in a 1:1 ratio in CD_2_Cl_2_ at rt, and the reaction was monitored by NMR spectroscopy. The ^1^H NMR spectra showed the appearance of a new singlet resonance at *δ* = 9.38 ppm, indicative of an aldimine signal, accompanied by multiplets in the aliphatic range (*δ* = 2.29–0.54 ppm). Stirring the reaction mixture for 24 h at rt resulted in complete, clean conversion (94% isolated yield), accompanied by a noticeable change in color from dark red to intense purple. Compound **3b** was crystallized at –40 °C from a diluted DCM/*n*‐hexane solution, affording purple, needle‐like single crystals. Single‐crystal x‐ray diffraction analysis confirmed the *anti*‐Markovnikov hydrostibination, in which the ligand methylene served as the hydride source (Figure 4B).

A similar reaction was observed with **2a** and styrene, leading to product **3c** in 97% isolated yield. Attempts to crystallize **3c** afforded only poor‐quality crystals unsuitable for x‐ray diffraction analysis, while identical NMR spectroscopic patterns and mass spectrometry confirmed the *anti*‐Markovnikov hydrostibination. Comparable reactivity of **2a** with more electron‐rich 4‐Me‐styrene and 4‐*^t^* Bu‐styrene was also achieved. The reaction of 4‐CF_3_‐styrene at rt took 1 week to reach 36% yield of stiba‐alkane compound (**3e**), along with some follow‐up chemistry. When the same substrates were heated to 60 °C, a product mixture was obtained in a 1:0.3 ratio, comprising the hydrostibinated product **3e** and a cycloaddition product **3e′** (Figure S30). Apparently, primary product **3e** undergoes [4+2] cycloaddition with an additional alkene substrate across the heterodiene backbone, resulting in the formation of a six‐membered ring product. With the substantially more electron‐poor methyl vinyl ketone as a substrate, undesirable side reactions were observed, whereas more electron‐rich substrates like 4‐methoxystyrene, furane, and ethyl vinyl ether led to polymeric products and decomposition of **2a**, showcasing the limitations of this system. However, compound **2a** further reacted smoothly with the strained bicyclic alkene norbornene (5 min at rt), resulting in the isolation of *exo*‐stiba‐norbornyl derivative (**3i**) in high yield (>95%), as well as with 1‐octene, leading to **3j** in 79% yield. No reactivity was observed with cyclohexene, cyclopentene, cis/trans‐stilbene, and 1,1‐diphenylethylene, indicating that the hydrostibination reactions are significantly influenced by the steric profile of alkene substrates, and not purely determined by their nucleophilicity. In the case of 1,5‐hexadiene, reaction with stibenium salt **2a** led to selective addition across only one side of the C═C bond (**3k**), while the other terminal alkene moiety remained unaffected. This mono‐addition is remarkable: comparable hydroelementation reactions lead to statistical mixtures of mono‐ and bisfunctionalized or cyclic products [77, 78, 79, 80, 81], while the mono‐selective functionalization of non‐conjugated dienes requires multisite‐binding transition‐metal catalysis, directing groups or templating strategies [8, 82, 83]. We explain this selectivity by the bound stibenium part, causing Coulomb repulsion or deactivation during the second hydrostibination.

**FIGURE 5:**
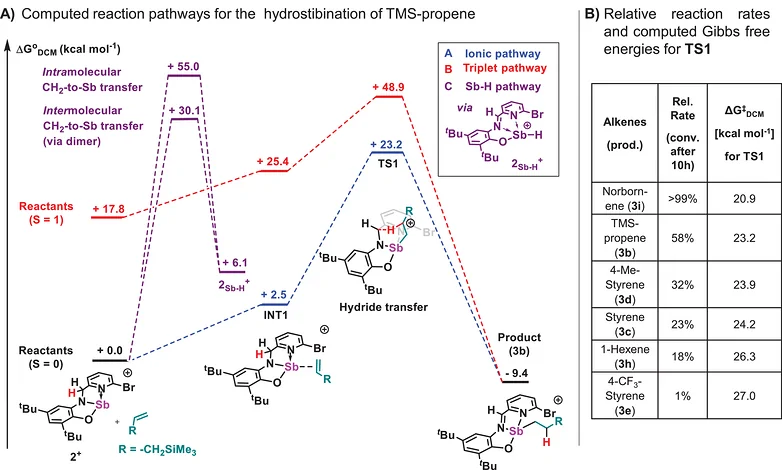
(A) Computed Gibbs free energies for the hydrostibination of TMS‐propene to **2a** (X2C‐ωB97‐D4/X2C‐TZVPall/CPCM(DCM) along pathways **A**‐**C**. (B) Relative rates, given as % of conversion after 10 h, and solvation‐corrected Gibbs free energies of the hydride‐transfer transition state of various alkenes.

The mechanism of the hydrostibination reaction was studied in more detail. Three different pathways were considered as possible (Figure 5): (A) ionic mechanism via olefin activation and hydride transfer, (B) open‐shell pathway via triplet intermediates, or (C) H‐atom/hydride transfer to an intermediately formed Sb─H species, followed by the previously reported hydrostibination [35]. To gauge the likelihoods of these mechanisms experimentally, the hydrostibination of styrene was investigated under varying reaction conditions (Figure 4C and Table S1). Using a nonpolar solvent such as toluene‐*d_8_* (*ε* = 2.4), **2a** exhibited only limited solubility. While the expected hydrostibination of styrene was still observed, the reaction only occurred at 60 °C. In contrast, the use of *o*‐DFB (*o*‐difluorobenzene, *ε* = 13.8) resulted in a threefold increase in the reaction rate compared to DCM (*ε* = 8.9). The faster reaction rate in more polar, non‐coordinating solvents contrasts with the observations for the radical hydrostibination of alkynes [35] but is more in line with an ionic pathway. Since *o*‐DFB also led to the partial polymerization of styrene, DCM was used as the solvent of choice for the other substrates. Performing the hydrostibination in acetonitrile (ACN) as the solvent resulted in a color change from dark red to orange, with hydrostibination of the olefinic substrates fully inhibited. Coordination of solvent molecules to antimony was suspected as the origin of this effect. Accordingly, conducting the reaction of **2a** in DCM but in the presence of pyridine resulted in the formation of a bis(pyridine) adduct **5** as a yellow solid, as confirmed by ^1^H NMR spectroscopy (see Section S3.2.2). Again, this compound was found unreactive toward olefins. The color change from red to yellow can be attributed to the disturbance of the *π*→π* transition, as was supported by TDDFT (see Section S8). The coordination can be reversed by using the stronger Lewis acid B(C_6_F_5_)_3_, abstracting the pyridine from **5**, regenerating **2a**, essentially enabling on/off control of this hydrostibination protocol (Scheme S3). Carrying out the reaction in the presence of radical trap N‐*tert*‐butyl‐α‐phenylnitrone (PBN), only 5% conversion to the hydrostibinated product was observed after 2 days at rt, along with the formation of unidentified side products. While this observation might indicate the involvement of radical pathway **B**, we assume that, just as with pyridine and ACN, coordination of this relatively potent Lewis base is causing the inhibition. When the reactions were performed in the presence of pure O_2_ or air, <1% of the hydrostibinated product was formed along with compound **4** and an unidentified mixture of products, accompanied by the formation of elemental antimony (Section S3.2.1). Under UV irradiation or in the dark, no significant changes in reaction rates were observed compared to ambient light, ruling out the involvement of a light‐induced pathway.

Since all these observations still did not allow to unequivocally distinguish between the possible pathways, combined kinetic studies and mechanistic computations were performed (Figure 5). Following the reactions in a 1:1 stoichiometry (**2a**: substrate) provided qualitative insights into reactivity rates. Comparing the amount of conversion after 10 h among the considered substrates gave the relative trend in the order **3i**>**3b**>**3d**>**3c**>**3h**>**3e** (Figure 5B and Section S3.3.1). For the series of isostructural *para*‐substituted styrenes, the reaction rate increased with more electron‐donating substituents, consistent with higher Mayr's nucleophilicity of 4‐Me‐styrene (*N* = 1.70) relative to unsubstituted styrene (*N* = 0.78) [84].

Accordingly, a 10‐fold decrease in rate was observed for the weakly nucleophilicity 4‐CF_3_‐styrene. However, Me_3_Si‐propene reacted noticeably faster, despite possessing a slightly lower nucleophilicity value (*N* = 1.68) than 4‐Me‐styrene. Hence, while being generally in line with ionic pathway **A**, factors beyond the nucleophilicity seemed to control the rate of reaction. Thus, all three possible mechanisms were examined via DFT calculations (X2C‐ωB97‐D4/X2C‐TZVPPall/CPCM(DCM); the [WCA]^−^ anions were omitted). The overall reaction is exergonic for all substrates (Table S10). In pathway **A**, the alkenes form a *π*‐complex intermediate **INT1** with the electrophilic Sb(III) center. This association was found to occur barrierless on the enthalpic potential energy surface, with **INT1** lying 2.5 kcal mol^−1^ uphill in free energy. No stable terminal‐C‐Sb *σ*‐complexes could be optimized, but the structures consistently relaxed to the *π*‐complex. *σ*‐bond formation is found only in the transition state **TS1**, with simultaneous hydride transfer from the ligand ─CH_2_ to the *β*‐carbo cation. The collapse of this **TS1** to the hydrostibination product in the forward direction and to the *π*‐complex in the backward direction was confirmed by IRC calculation (see Figure S85). Most importantly, the computed reaction barriers are in perfect agreement with the experimental trends (Figure 5B), strongly supporting this hydride transfer‐controlled pathway **A** as the most plausible mechanism. Since the CASSCF computations for **2^+^** indicated minor singlet diradicaloid character (vide infra), it is surprising that the reaction barriers are well reproduced with a single reference DFT method. We assumed that the *π*‐complex **INT1** is possessing a pure Sb(III) ground state, rendering the closed‐shell ionic pathway as suitable for being captured by the given method. Indeed, it was found that the involvement of the doubly excited configuration state function in **INT1** is significantly reduced (see Section S8). Arguments against triplet diradical pathway **B** are the much higher triplet state energy of **2^+^** (17.8 kcal mol^−1^, vide infra), as well as the *π*‐complexes or transition states that are consistently higher in energy compared to that of the restricted computations. Of note, stable Sb–C σ‐complex intermediates were found in the triplet manifold, but 3.9 kcal mol^−1^ (Me_3_Si‐propene) higher in energy compared to the singlet state *π*‐complex **INT1**. Finally, pathway **C** was investigated computationally, which would involve the formation of an Sb─H intermediate, **2_Sb‐H_^+^**, by intra‐ or intermolecular hydrogen migration, followed by hydrostibination according to the previous report [34]. Indeed, the putative Sb–H isomer **2_Sb‐H_^+^** is calculated to lie only 6.1 kcal mol^−^^1^ above **2^+^**. While the barrier for intramolecular hydrogen atom transfer from **2^+^** to **2_Sb‐H_^+^** was found energetically inaccessible with TS barriers of 55.0 kcal mol^−1^ (*S* = 0) and 71.82 kcal mol^−1^ (*S* = 1), an intermolecular CH_2_‐to‐Sb transfer barrier was found energetically more feasible (Δ*G* ^‡^_DCM_ = 30.1 kcal mol^−1^). Hence, since process **C** lies 6.9 kcal mol^−1^ above the direct hydride transfer pathway **A**, it can be considered unlikely. However, additional kinetic studies were performed to further rule out pathway **C**. The formation of **2_Sb‐H_^+^** via the only feasible computed intermolecular CH_2_‐to‐Sb transfer would imply a second‐order behavior of **2a** in the reaction. However, variable time normalization analysis (VTNA) [85] of the hydrostibination of 3,3‐dimethyl‐1‐butene (yielding **3g**) revealed a reaction that is first order in **2a** and in the olefin substrate (Section S3.3.2). Hence, all experimental and computational results are in strong favor of hydride pathway **A**, representing a unique element–ligand cooperative hydrostibination of olefins.

**FIGURE 6:**
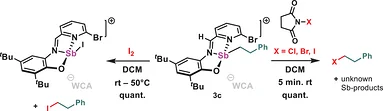
Syntheses of haloalkanes from **3c**.

To touch upon the synthetic relevance emerging from these Sb─C bonds, some preliminary reactivity was screened (Figure 6). Reaction of stiba‐alkane **3c** with I_2_ at rt leads to the selective and complete formation of the corresponding iodoalkanes and the iodo‐stibenium ion. Similarly, treating **3c** with NCS, NBS, and NIS (N‐halo‐succinimide) reagents led to immediate and clean conversion to 2‐haloethyl benzene and succinimide addition to stibenium ion, which decomposes further in reaction. All products were purified and confirmed by ^1^H NMR (see Section S5). Analogous reactivity was observed for **3c–3f**, which underwent smooth halogenation with I_2_ and NCS reagents to afford the corresponding haloalkanes, underscoring the potential of hydrostibination products for downstream functionalization.

**FIGURE 7:**
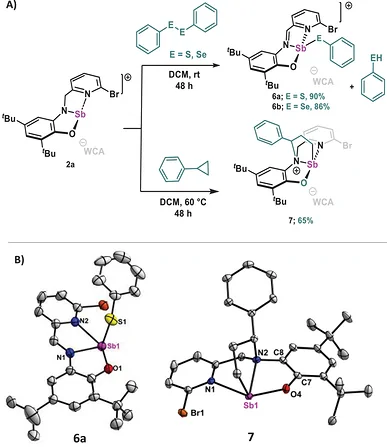
(A) Syntheses of compounds **6a, 6b**, and **7**. (B) Molecular structure of **6a** (left) and **7** (right). Thermal ellipsoid plot of **6a** and **7** at 50% probability. All hydrogen atoms and the counter anion [Al(OC(CF_3_)_3_)_4_]^−^ were omitted for clarity. Selected bond distances (Å) and angles (deg) of **6a**: d(Sb1–O1) = 2.039(3), d(Sb1–N1) = 2.204(4); d(Sb1–N2) = 2.460(4); d(O1–C8) = 1.348(6), d(N1–C7) = 1.399(6); ∠O1‐Sb1‐N1 = 76.48(1), ∠N2‐Sb1‐N1 = 69.55(1), ∠O1‐Sb1–N1 = 145.27(1). Selected bond distances (Å) and angles (deg) of **7**: d(Sb1–O4) = 2.0467(1), d(Sb1–N2) = 2.245(1); d(Sb1–N1) = 2.436(1); d(O4–C7) = 1.350(2), d(N2–C8 = 1.479(2); ∠O1–Sb1–N2 = 75.98(5), ∠N2–Sb1–N1 = 70.47(5), ∠O1–Sb1–N1 = 146.21(5).

To probe the reactivity of **2a** with other substrates, additional reactions were performed. The reaction between **2a** and Ph_2_Ch_2_ (Ch = S, Se) in a 1:1 ratio in DCM at rt led to the formation of [L‐Sb(ChPh)][WCA] (**6a** and **6b**) in yields of 90% and 86%, along with H‐ChPh formation (Figure 7A, top). Dark green color crystals of **6a** were grown and characterized using single X‐ray diffraction (Figure 7B, left) and NMR spectroscopy (see Section S4). Related reactions have been observed with Sb(III) and Sb(I) species [47, 86, 87, 88]. Finally, **2a** was reacted with cyclopropyl benzene in DCM at 60°C, leading to the formation of a 5‐membered ring via addition at the Sb─N bond of **2a** as a major product **7** (Figure 7B, left). Again, this reaction can be rationalized via an ionic pathway with a computed activation barrier of 32.2 kcal mol^−1^, in line with the need for heating to 60 °C over 2 days.

## 3 Conclusion

In summary, we report a structurally constrained, T‐shaped stibenium(III) ion supported by an amidophenolato‐pyridyl ligand and its unique hydrostibination reactivity via ELC. Spectroscopic and computational analyses reveal a highly polarizable Sb–*π* system with partially reduced antimony, yet reactions proceeding through a well‐defined ionic pathway characteristic of a Lewis‐acidic Sb(III) center. The additive‐ and radical‐free hydrostibination delivers *anti*‐Markovnikov stiba‐alkanes in excellent yields across a broad alkene scope, and the products are readily transformed into haloalkanes in quantitative conversion. It represents a rare example of a synthetically relevant transformation at a redox‐confused pnictogen, highlighting the potential of this substance class [89].

More generally, by embedding the hydride equivalent within the ligand scaffold, this approach circumvents the relative instability of antimony hydrides and provides a complementary alternative to direct Sb─H‐based strategies. In light of the growing interest in organoantimony chemistry [36], particularly Sb–alkyl motifs [90], this cooperative strategy offers a valuable platform for advancing organo‐heavy *p*‐block element chemistry. More broadly, it might establish a blueprint for hydroelementation with heavy *p*‐block elements and weak or inaccessible E─H bonds.

## Author Contributions

**Ekta Nag**: investigation, formal analysis, data curation, writing – original draft, conceptualization, visualization. **Lars Ole Busse**: validation, investigation, formal analysis, data curation, writing – review and editing. **Andreas Albers**: methodology, validation, writing – review and editing, formal analysis, investigation. **Manuel Schmitt**: formal analysis, writing – review and editing, validation. **Lutz Greb**: supervision, conceptualization, formal analysis, funding acquisition, writing – review and editing, methodology, resources, project administration, visualization.

## Conflicts of Interest

The authors declare no conflicts of interest.

## Supporting information



The data that support the findings of this study are available in the Supporting Information of this article. Deposition Numbers 2537307–2537311 contain the supplementary crystallographic data for this paper. These data can be obtained free of charge from the Cambridge Crystallographic Data Centre.
**Supporting File**: anie73553‐sup‐0001‐SuppMat.pdf.

## Data Availability

The data that support the findings of this study are available in the Supporting Information of this article.
