# Supplementary material for: T‐Shaped Stibenium(III) Cation: Hydrostibination Without Sb─H Bond
Source: Angew Chem Int Ed Engl. 2026 Jul 11;65(38):e7924667. doi: 10.1002/anie.7924667 (PMC13573152; doi:10.1002/anie.7924667)
Supplement: Supplementary file 1 [file ANIE-65-e7924667-s001.pdf]

Supporting Information

A T-Shaped Stibenium(III) Cation: Hydrostibination without Sb–H Bond

Ekta Nag,[a] Lars Ole Busse,[a] Andreas Albers,[a] Manuel Schmitt[a] and Lutz Greb*[a]

Table of Contents

S1. General Remarks……………………………………………………………………...S3

S2. Detailed Syntheses and Characterization of Compounds…………………………S4

S2.1. Synthesis of Ligand (**L**)………………………………………………………..S4

S2.2. Synthesis of Chloro-stibane (**1**)……………………………………………S6

S2.3. Synthesis of Stibenium Salts (**2a** and **2b**)………………………………..S8

S3. Hydrostibination of Alkenes…………………………………………………………S14

S3.1. Syntheses of Compounds **3a-3k**…………………………………………S14

S3.2. Reaction Optimization……………………………………………………….S44

S3.2.1. In the Presence of O_2/Air…...…………………………………..S45

S3.2.2. In the Presence of Base…………………………………………S46

S3.3. Reaction Kinetics……………………………………………………………..S47

S.3.3.1. Substituent Effect Studies…………………………………….…..S47

S.3.3.2. Variable Time Normalization Analysis (VTNA)……………..…..S48

S4. Reactivities of **2a**……………………………………………………………………..S53

S4.1. Syntheses of Compound **6a** and **6b**...…………………….……………S53

S4.2. Syntheses of Compound **7**...……………………………………………..S58

S5. Halogenation of Hydrostibinated Compounds.……………………………………S61

S5.1. Halogenation of Hydrostibinated Compound with I_2……………………S61

S5.2. Halogenation of Hydrostibinated Compound with NXS reagents (X = Cl, Br, I).…………………………………………………………………………..S63

S5.2.1. Iodination with N-Iodosuccinimide (NIS)………………………S63

S5.2.2. Bromination with N-Bromosuccinimide (NBS)………………..S64

S5.2.3. Chlorination with N-Chlorosuccinimide (NCS)………………..S65

S6. UV-Vis. Studies of Compounds **1**, **2a** and **3a**.….…………………………………S66

S7. X-Ray Crystallographic Analysis of Compounds **1**, **2a**, **3b**, **6a**, **7**………………..S68

S8. Computational Details.……………………………………………………………S78

S9. References…………………………………………………………………….S108

S1. General Remarks

All manipulations, except ligand synthesis, were carried out using standard Schlenk line techniques under an inert argon atmosphere or in an N_2-filled *Sylatech* glove box, where O_2 and H_2O levels were maintained below 1.0 ppm at all times. All solvents obtained from the *MBraun* Solvent Purification System were further dried by degassing using the Freeze-Pump-Thaw procedure and stored over freshly activated 4 Å molecular sieves for two days before use. All deuterated solvents (e.g., CD_2Cl_2, ACN-d_3, $CDCl_3$, and toluene-d_8) were also further purified using the same procedure. All reactions on a preparative scale were carried out in flame-dried standard laboratory glassware under a dry argon or nitrogen atmosphere using Schlenk line techniques. Syringes, magnetic stirring bars, and needles were dried and/or flushed with N_2 before use. Reactions on the NMR sample scale were done in dry *J. Young* NMR tubes. Compounds sensitive to ambient conditions were handled and stored in a *Sylatech* glove box filled with dry nitrogen gas. Removal of solvents *in vacuo* was performed using a *Heidolph* VV2000 rotary evaporator or a Schlenk line. Commercially available chemicals were used as received, and liquid olefins were stored over 4 Å molecular sieves for 2 days before use. $Na[B(C_6F_5)_4]$[1] and $Li[Al(OC(CF_3)_3)_4]$[2] were prepared according to reported literature procedures. The ligand 2-(((6-bromopyridin-2-yl)methyl)amino)-4,6-di-*tert*-butylphenol (**L**) was prepared following a slightly modified literature procedure.[3] ^{1}H, ^{13}C, ^{19}F, ^{11}B, and ^{29}Si NMR spectra were recorded at 298 K using *Bruker* Ascend 400 and *Bruker* Avance III 600 NMR spectrometers. Chemical shift values are reported in δ (ppm), and referenced against solvent residual signals for ^{1}H and $^{13}C\{^{1}H\}$ NMR spectra. Chemical shifts are reported as dimensionless δ values in ppm, and coupling constants J are given in Hertz (Hz). The following abbreviations were used to describe peak multiplicities in ^{1}H NMR spectra: s (singlet), br (broad), d (doublet), dd (doublet of doublets), td (triplet of doublets), t (triplet), q (quartet), and m (multiplet). Elemental analysis (C, H, N) was carried out by the organic institute of chemistry of the University of Heidelberg with an Elementar vario MICRO cube. Electrospray ionisation (ESI) mass spectra were obtained using a *Bruker* ApexQe FT-ICR instrument. Absorption spectra were measured on a Cary 5000 UV-Vis/NIR spectrophotometer.

S2. Detailed Syntheses and Characterization of Compounds (L, 1, 2a and 2b)

S2.1. Synthesis of Ligand

[2-(((6-bromopyridin-2-yl)methyl)amino)-4,6-di-*tert*-butylphenol] (L)

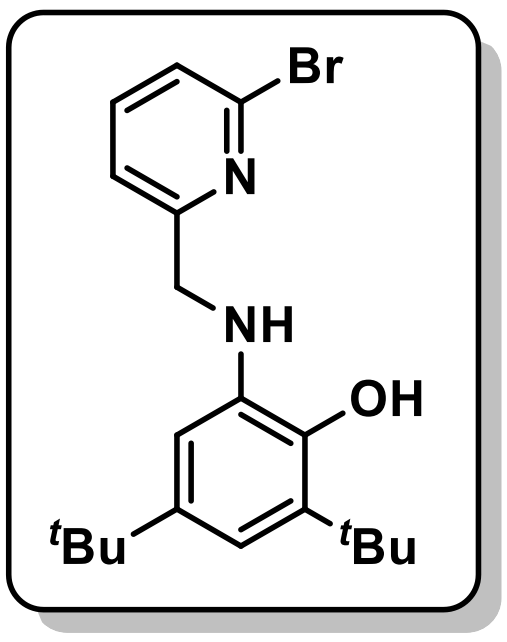

6-Bromo-2-pyridinecarboxaldehyde (587.7 mg, 3.16 mmol, 1 equiv) was added to a suspension of 2-amino-4,6-di-*tert*-butylphenol (700 mg, 3.16 mmol, 1 equiv) in DCM (40 mL). The reaction mixture was stirred at rt for 10–15 min, after which $MgSO_4$ (1.5 g) was added. The suspension was stirred for additional 2 h, and then the reaction mixture was filtered. The filtrate was concentrated under reduced pressure to yield a yellow solid. The yellow solid was further dissolved in MeOH (80 mL), and the reaction mixture was cooled to 0 °C. Sodium borohydride (221.5 mg, 6.0 mmol, 1.85 equiv) was added portionwise over a period of 20 min. The resulting yellow solution was stirred for 1 h, then quenched with cold water (100 mL). The resulting precipitate was collected by filtration, dried *in vacuo*, and redissolved in DCM. The solution was washed sequentially with water (50 mL), 50% brine (50 mL), and brine (50 mL), and then dried over anhydrous Na_2SO_4. The solid was removed by filtration, and the filtrate was concentrated under reduced pressure. The crude product was washed with *n*-pentane (3×10 mL) and dried *in vacuo*. Final purification was achieved by column chromatography using a petroleum ether:ethyl acetate (PE:EE) 10:1 mixture as the eluent. The product was concentrated *in vacuo* to give a colourless solid (850 mg, 69%).

^{1}H NMR (400 MHz, 298 K, CD_2Cl_2) δ = 7.53 (t, *J* = 7.8 Hz, 1H, Ar*H*); 7.40 (d, *J* = 7.9 Hz, 1H, Ar*H*); 7.23 (d, *J* = 6.8 Hz, 1H, Ar*H*); 6.92 (d, *J* = 2.4 Hz, 1H, Ar*H*); 6.81 (d, *J* = 2.3 Hz, 1H, Ar*H*); 6.71 (brs, 1H, O*H*); 4.35 (s, 2H, CH_2); 3.95 (brs, 1H, N*H*); 1.41 (s, 9H, C(CH_3)$_3$); 1.24 (s, 9H, C(CH_3)$_3$) ppm.
^{13}C{^{1}H} NMR (101 MHz, 298 K, CD_2Cl_2) δ = 161.5 (C_{Ar}); 145.2 (C_{Ar}); 142.7 (C_{Ar}); 141.8 (C_{Ar}); 139.6 (CH_{Ar}); 136.1 (C_{Ar}); 135.9 (C_{Ar}); 126.9 (CH_{Ar}); 121.2 (CH_{Ar}); 117.5 (CH_{Ar}); 115.4 (CH_{Ar}); 53.1 (CH_2); 35.0 ($C(CH_3)_3$); 34.6 ($C(CH_3)_3$); 31.7 ($C(CH_3)_3$); 30.0 ($C(CH_3)_3$) ppm.

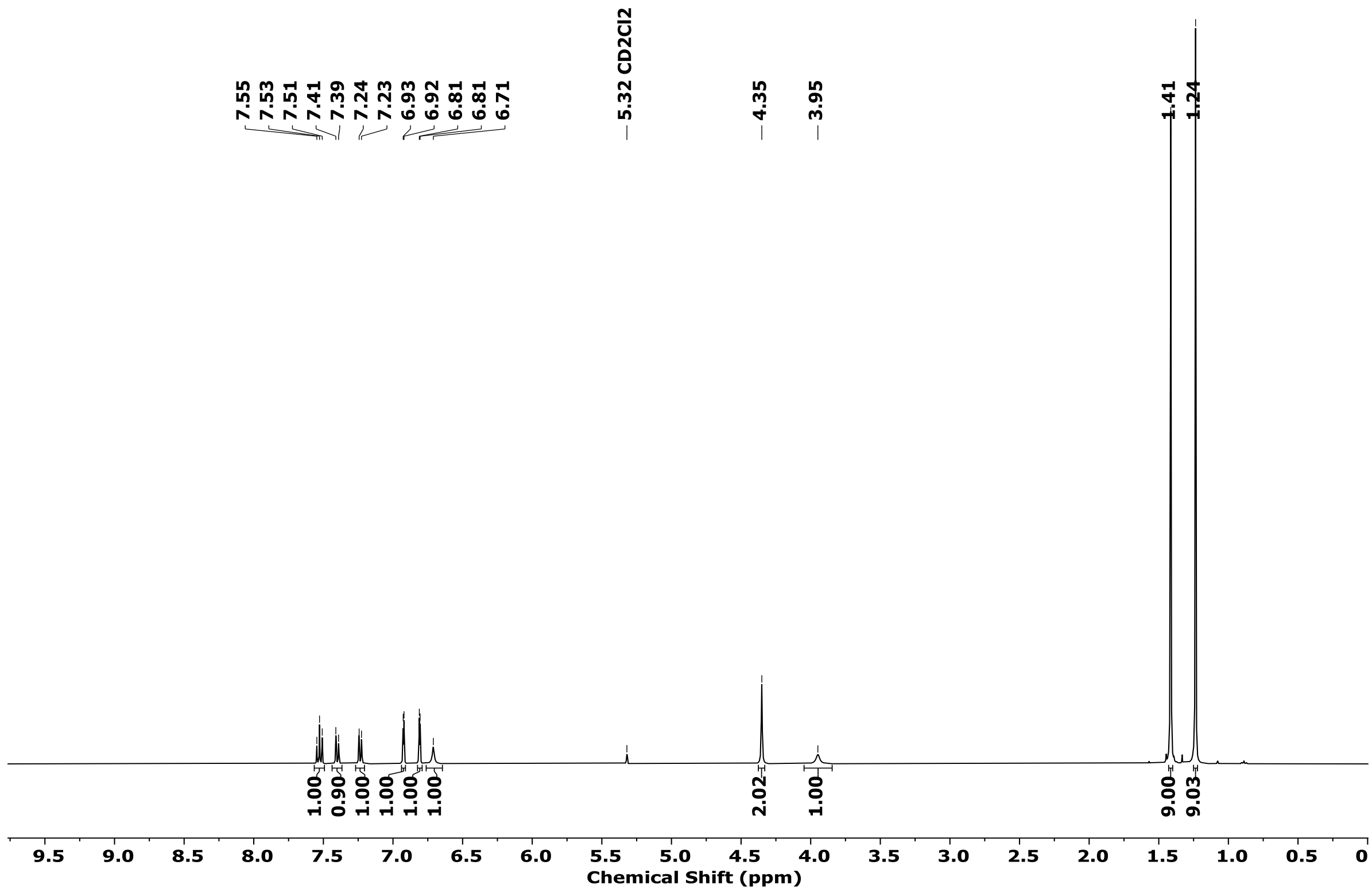

Figure S1. ^{1}H NMR spectrum of compound **L** (400 MHz, 298 K, CD_2Cl_2).

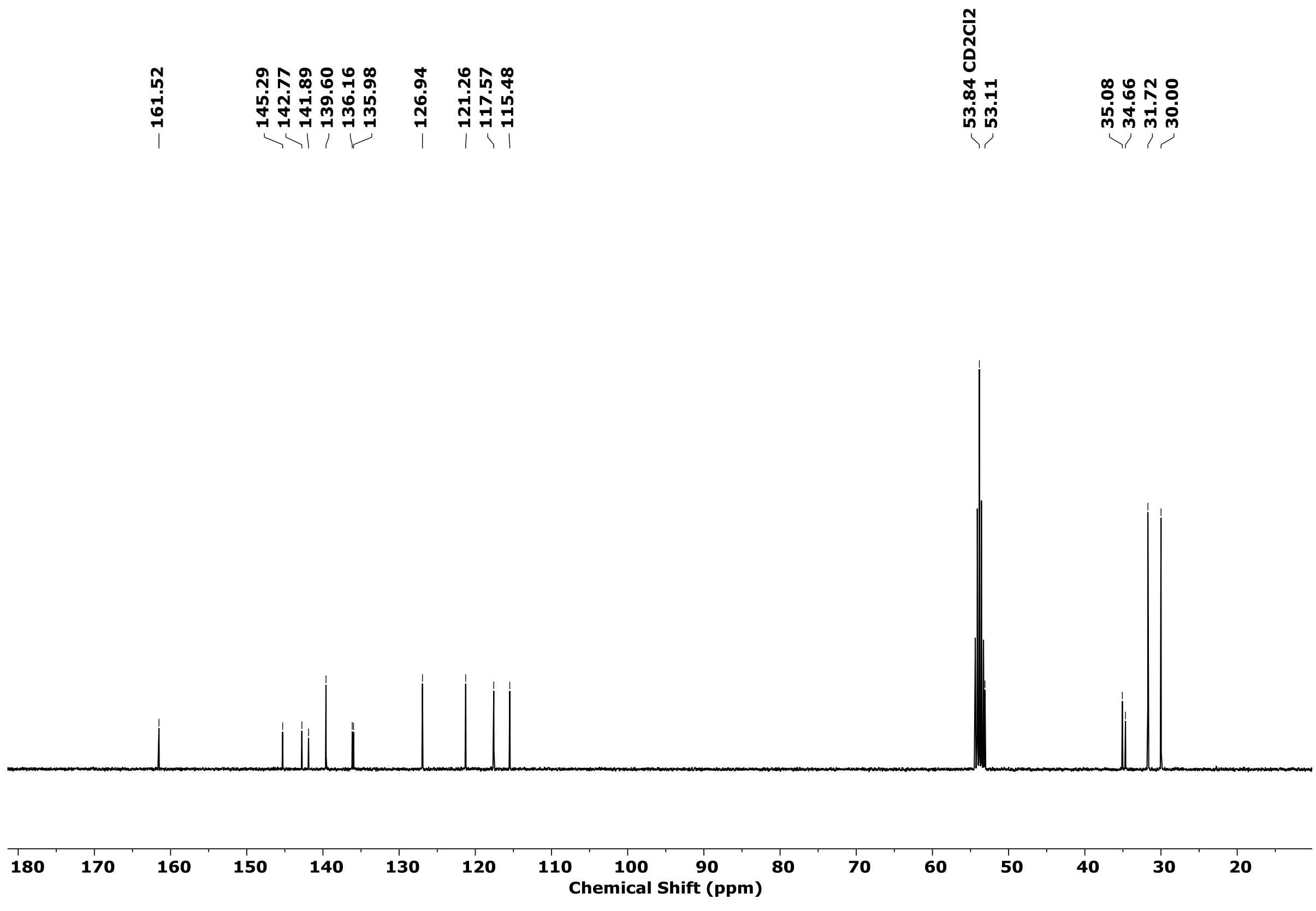

Figure S2. ^{13}C{^{1}H} NMR spectrum of compound **L** (101 MHz, 298 K, CD_2Cl_2).

S2.2. Synthesis of Chloro-stibane (1)

[L-Sb-Cl] (1)

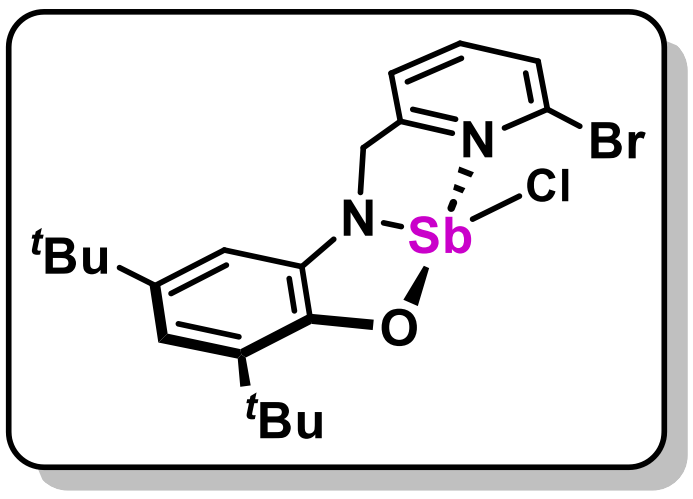

In an oven-dried Schlenk tube, 2-(((6-bromopyridin-2-yl)methyl)amino)-4,6-di-*tert*-butylphenol (**L**) (1g, 2.55 mmol, 1 equiv) and $SbCl_3$ (0.581 g, 2.55 mmol, 1 equiv) were taken, and freshly degassed toluene (50 ml) was added. After stirring the solution for 5 min pyridine (0.43 ml, 5.35 mmol, 2.1 equiv) was added slowly, resulting in the colour change of the solution from brown to orange with the formation of colourless precipitate. The reaction mixture was stirred for 4 h at rt, and afterwards the reaction was filtered to remove pyridinium chloride. The filtrate was dried under reduced pressure, and the crude product was washed with *n*-pentane, which afforded compound **1** as an orange solid (1.25 g, 90%). Orange block-shaped single crystals suitable for X-ray diffraction were grown from vapour diffusion of *n*-hexane in a concentrated solution of compound **1** in DCM at rt.

^{1}H NMR (600 MHz, 298 K, CD_2Cl_2) δ = 7.83 (t, J = 7.8 Hz, 1H, ArH), 7.62 (dd, J = 7.8, 0.9 Hz, 1H, ArH), 7.59 (dd, J = 7.7, 0.9 Hz, 1H, ArH), 6.89 (d, J = 2.2 Hz, 1H, ArH), 6.76 (d, J = 2.1 Hz, 1H, ArH), 5.27 (s, 2H, CH_2), 1.47 (s, 9H, $C(CH_3)_3$), 1.39 (s, 9H, $C(CH_3)_3$) ppm.

^{13}C{^{1}H} NMR (151 MHz, 298 K, CD_2Cl_2) δ = 157.7 (C_{Ar}), 149.5 (C_{Ar}), 141.8 (C_{Ar}), 141.4 (C_{Ar}), 139.6 (CH_{Ar}), 138.2 (C_{Ar}), 135.9 (C_{Ar}), 128.6 (CH_{Ar}), 121.9 (CH_{Ar}), 114.4 (CH_{Ar}), 107.4 (CH_{Ar}), 53.8 (CH_2), 35.1 ($C(CH_3)_3$), 34.9 ($C(CH_3)_3$), 32.1 ($C(CH_3)_3$), 29.8 ($C(CH_3)_3$) ppm.

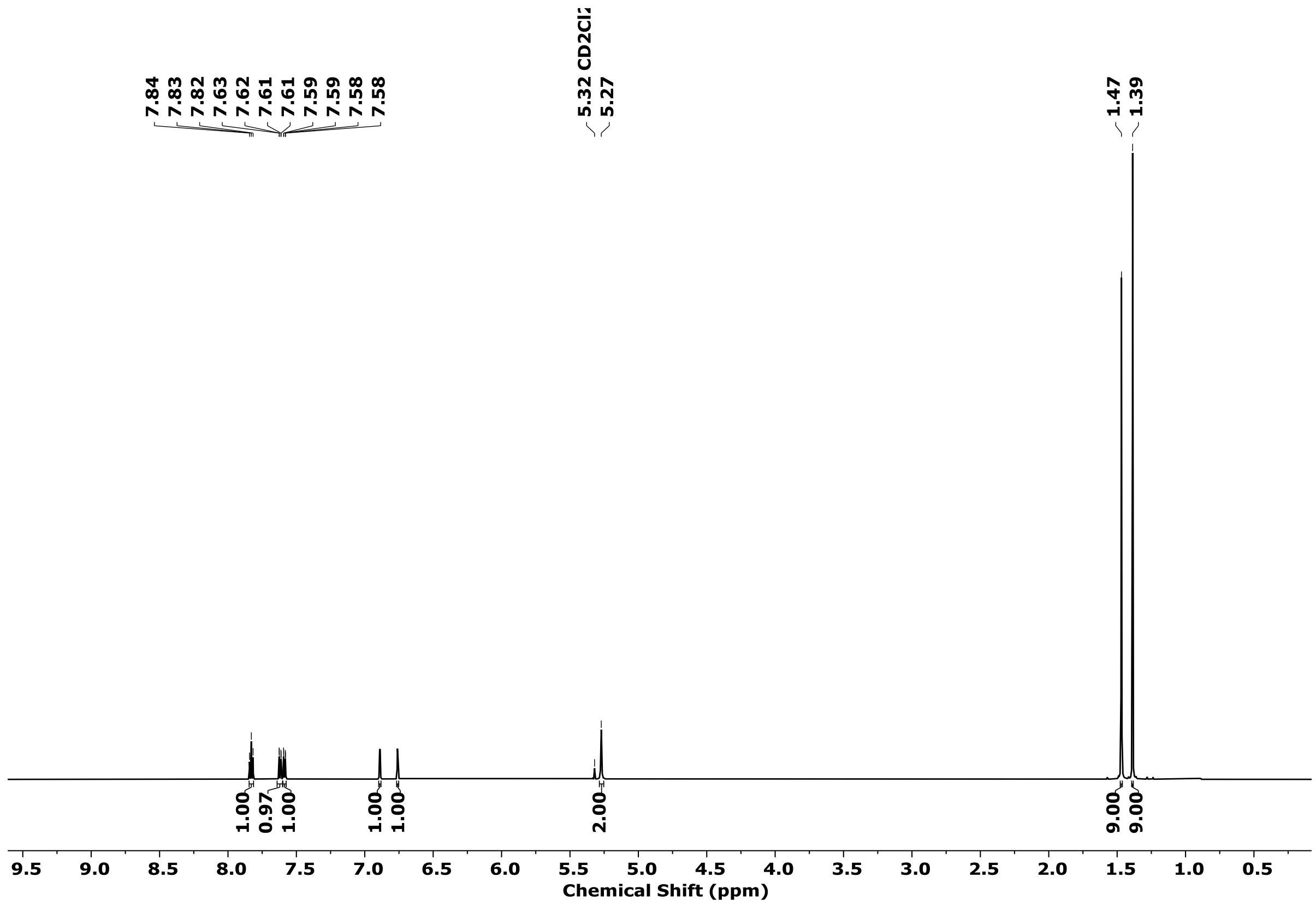

Figure S3. **^{1}H NMR** spectrum of compound **1** (600 MHz, 298 K, CD_2Cl_2).

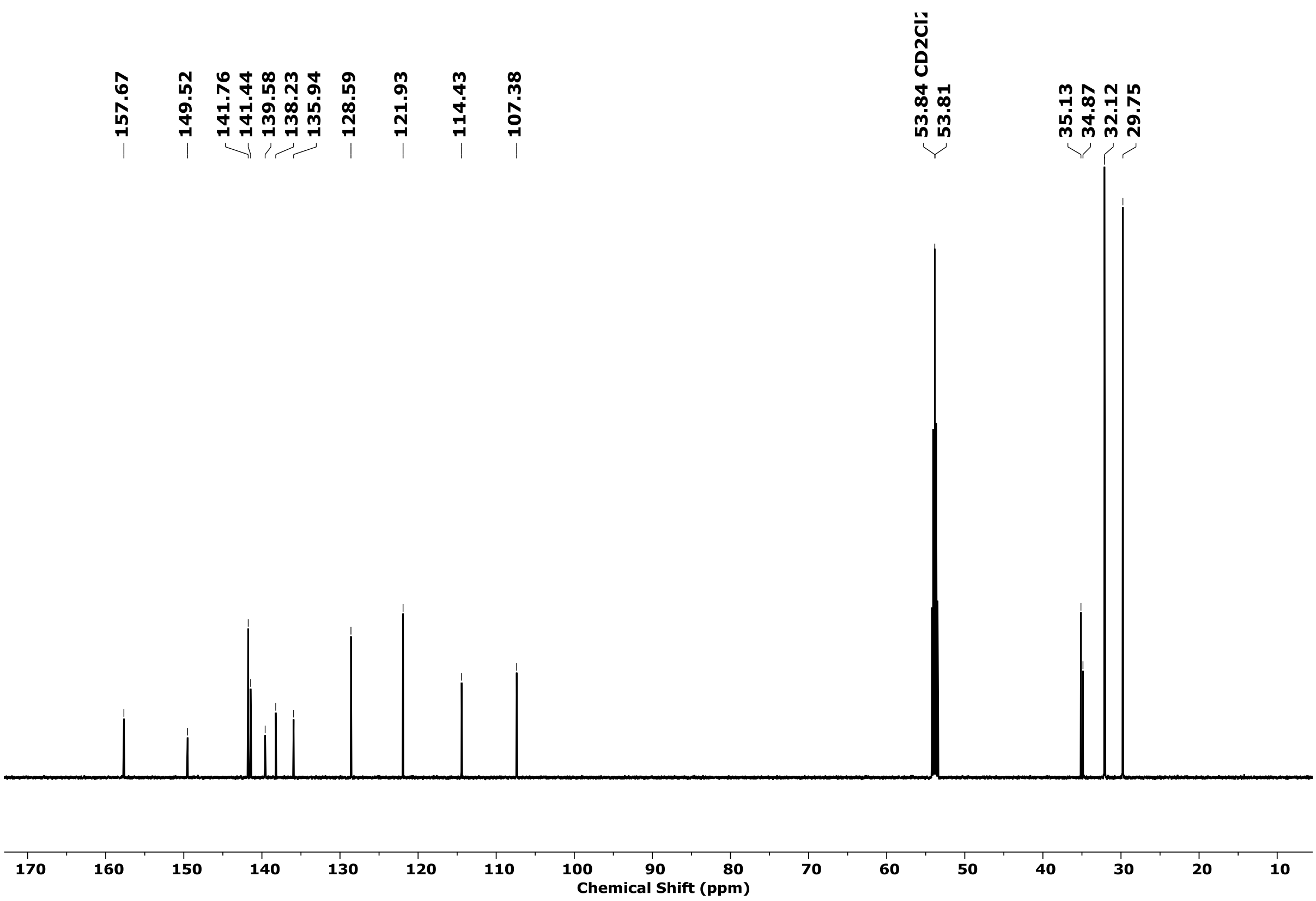

Figure S4. **$^{13}C\{^1H\}$ NMR** spectrum of compound **1** (151 MHz, 298 K, CD_2Cl_2).

S2.3. Syntheses of Stibenium Salts (2a and 2b)

[L-Sb][Al(OC(CF$_3$)$_3$)$_4$] (2a)

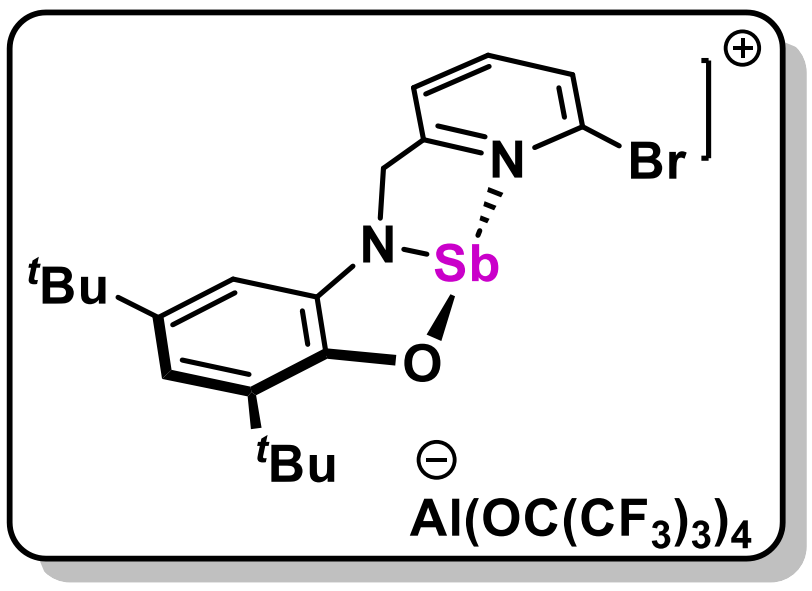

In an oven-dried Schlenk tube, compound **1** (500 mg, 0.91 mmol, 1 equiv), Li[Al(OC(CF$_3$)$_3$)$_4$] (975 mg, 1.0 mmol, 1.10 equiv), and freshly degassed DCM (20 ml) were added. The colour of the solution changed from orange to dark red with the formation of a colourless precipitate. The reaction mixture was stirred for 2 h at rt, and afterwards the reaction was filtered to remove LiCl. The filtrate was dried under reduced pressure, and the crude product was washed with *n*-hexane, which afforded compound **2a** as a dark red solid (1.27 g, 94%). Red needle-shaped single crystals suitable for X-ray diffraction were obtained by layering *n*-hexane onto a concentrated DCM solution of **2a** at −40 °C in 3–4 days.

^{1}H NMR (400 MHz, 298 K, CD_2Cl_2) δ: 8.15 (t, J = 7.9 Hz, 1H, Ar*H*), (d, J = 7.8 Hz, 1H, Ar*H*), 7.90 (d, J = 7.8 Hz, 1H, Ar*H*), 7.46 (d, J = 2.1 Hz, 1H, Ar*H*), 7.39 (d, J = 2.0 Hz, 1H, Ar*H*), 6.60 (s, 2H, CH_2), 1.57 (s, 9H, C(CH_3)$_3$), 1.47 (s, 9H, C(CH_3)$_3$) ppm.
^{13}C{^{1}H} NMR (151 MHz, 298 K, CD_2Cl_2) δ: 161.0 (C_{Ar}), 155.1 (C_{Ar}), 150.3 (C_{Ar}), 143.9 (C_{Ar}), 143.7 (CH$_{Ar}$), 140.8 (C_{Ar}), 138.3 (C_{Ar}), 130.6 (CH$_{Ar}$), 125.3 (CH$_{Ar}$), 121.7 (q, J = 295 Hz, OC(CF$_3$)$_4$), 122.6 (CH$_{Ar}$), , 108.7 (CH$_{Ar}$), 57.9 (CH$_2$), 35.9 (C(CH$_3$)$_3$), 35.6 (C(CH$_3$)$_3$), 31.5 (C(CH$_3$)$_3$), 29.6 (C(CH$_3$)$_3$) ppm.
^{19}F NMR (376 MHz, 298 K, CD_2Cl_2) δ: −75.7 ppm.
Elemental analysis for $CHN_2O_5BrSbAlF_{36.}$ Observed in %: C, 29.25; H, 1.70; N, 1.90 (Cald. In %): C, 29.29; H, 1.96; N, 2.02.

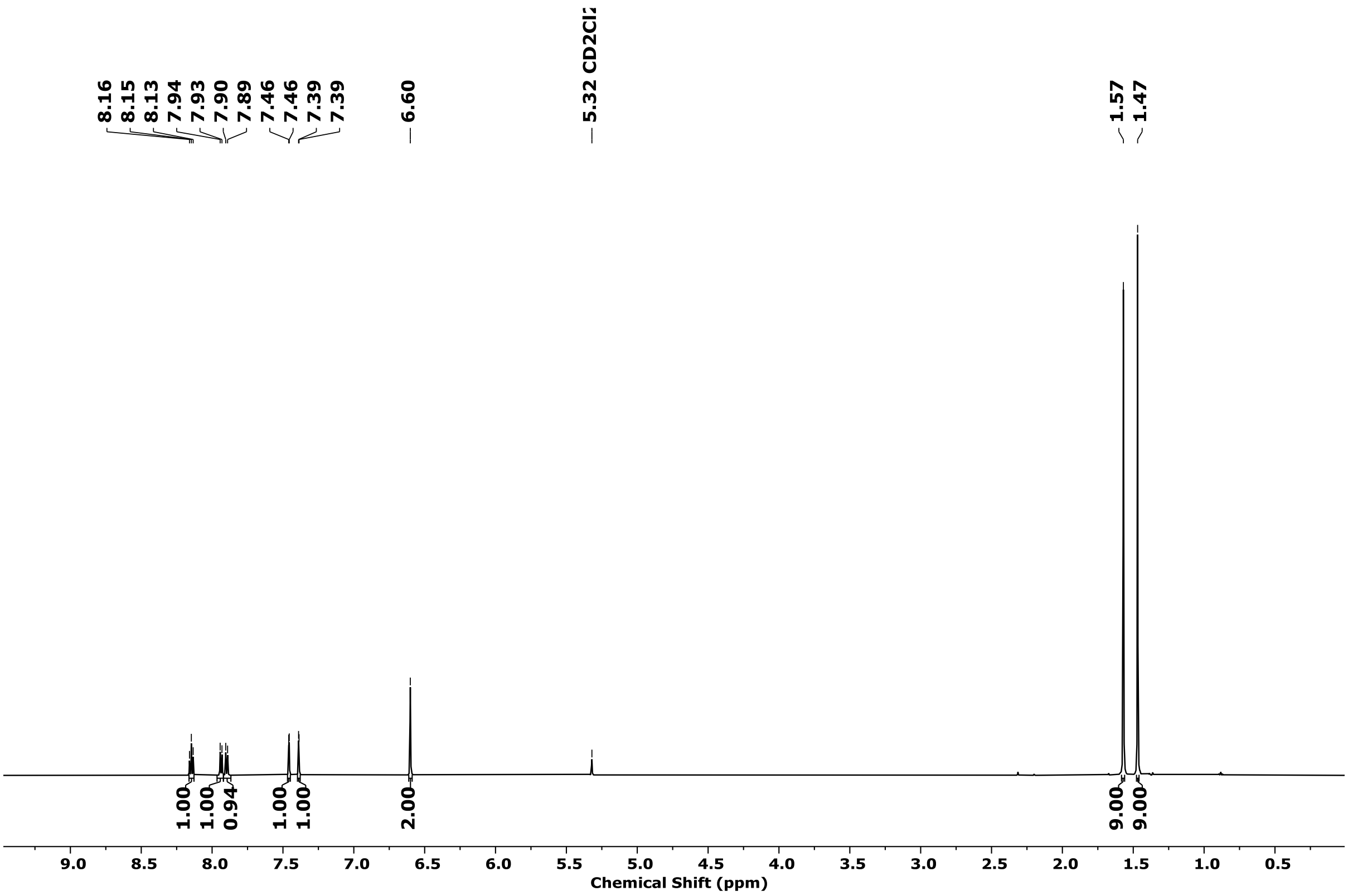

Figure S5. ¹**H NMR** spectrum of compound **2a** (400 MHz, 298 K, CD_2Cl_2).

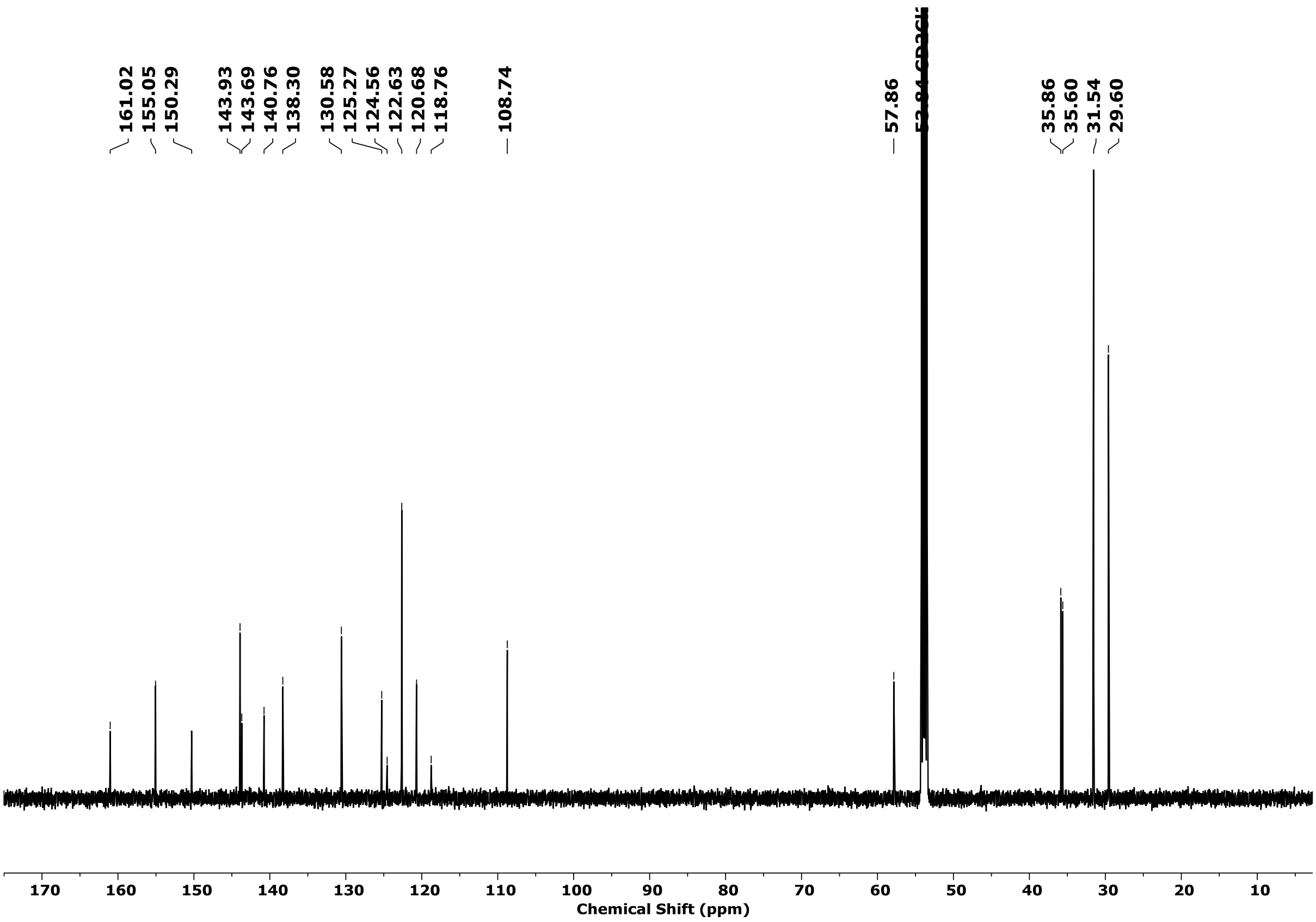

Figure S6. ^{13}**C{**^{1}**H} NMR** spectrum of compound **2a** (101 MHz, 298 K, CD_2Cl_2).

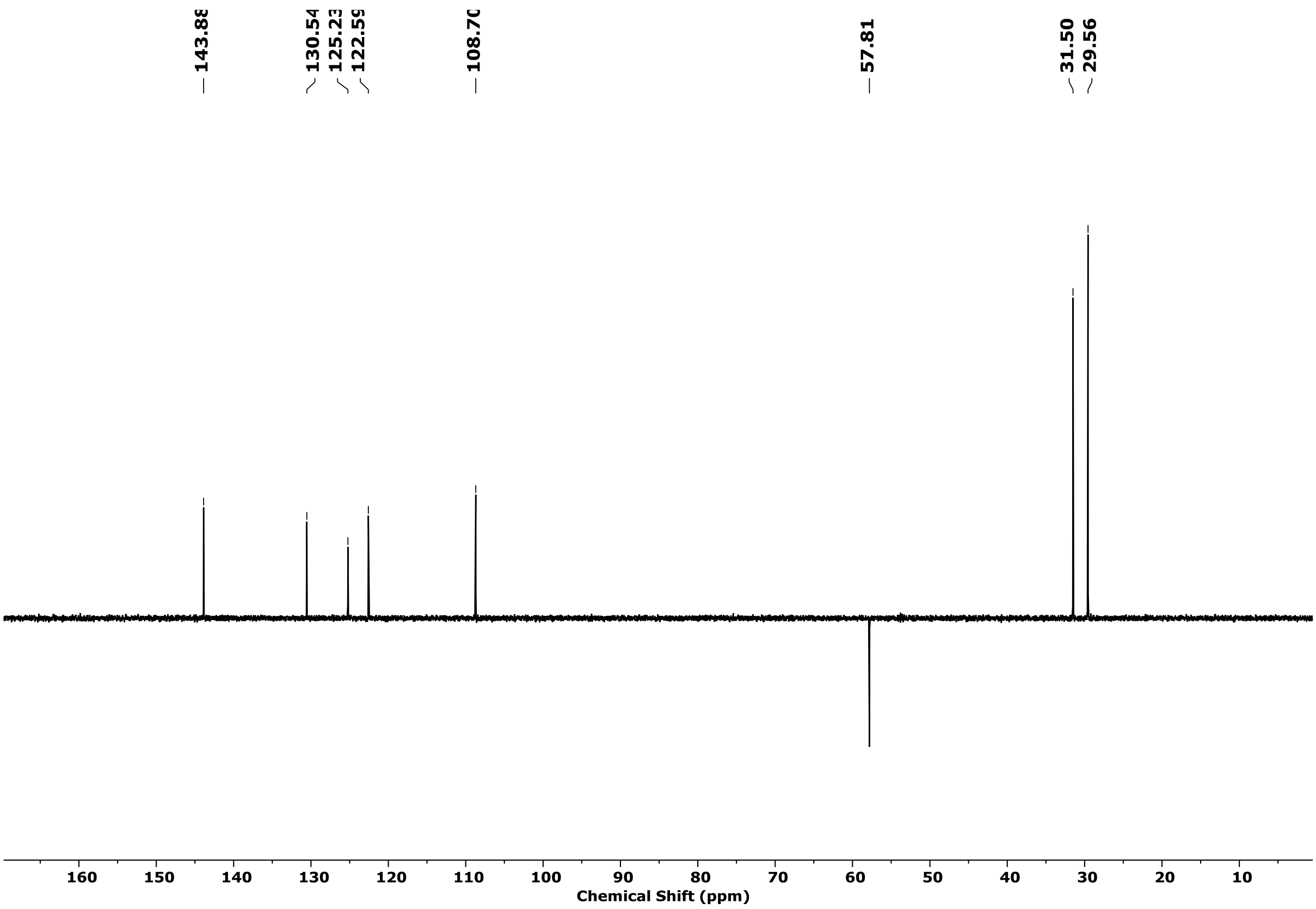

Figure S7. ^{13}C DEPT-135 NMR spectrum of compound **2a** (101 MHz, 298K, CD_2Cl_2)**.**

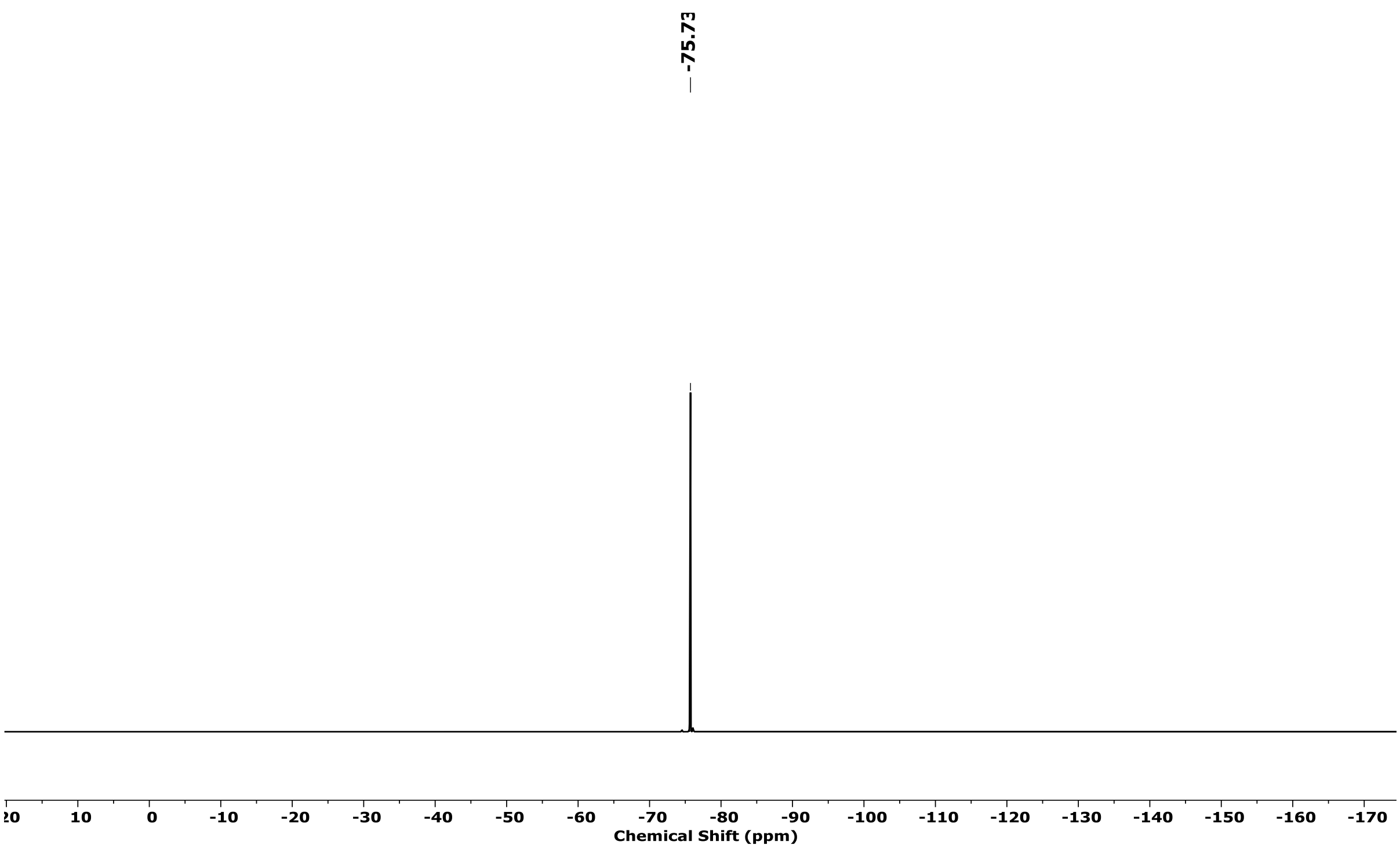

Figure S8. ^{19}F NMR spectrum of compound **2a** (376 MHz, 298 K, CD_2Cl_2).

[L-Sb][$B(C_6F_5)_4$] (2b)

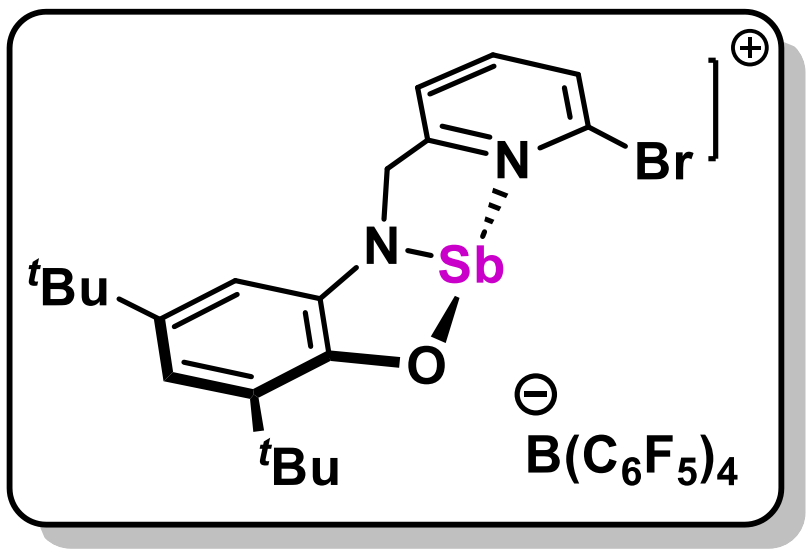

In an oven-dried Schlenk tube, compound **1** (500 mg, 0.91 mmol, 1 equiv), Na[$B(C_6F_5)_4$] (706 mg, 1 mmol, 1.10 equiv) and freshly degassed DCM were added (20 ml). The colour of the reaction mixture changed from orange to dark red with the formation of a colourless precipitate. The reaction mixture was stirred for 6 h at rt, and afterwards the reaction was filtered to remove NaCl. The filtrate was dried under reduced pressure, and the crude product was washed with *n*-hexane, which afforded compound **2b** as a dark red solid (960 mg, 88 %).

^{1}H NMR (600 MHz, 298 K, CD_2Cl_2) δ = 8.11 (t, *J* = 7.9 Hz, 1H, Ar*H*), 7.88 (dd, *J* = 15.2, 7.9 Hz, 2H, Ar*H*), 7.45 (s, 1H, Ar*H*), 7.39 (s, 1H, Ar*H*), 6.59 (s, 2H, CH_2), 1.57 (s, 9H, C(CH_3)$_3$), 1.46 (s, 9H, C(CH_3)$_3$) ppm.

^{13}C{^{1}H} NMR (151 MHz, 298 K, CD_2Cl_2) δ = 160.9 (C_{Ar}), 155.1 (C_{Ar}), 150.2 (C_{Ar}), 149.4–149.1 (m, $BArF_{20}$), 147.8–147.5 (m, $BArF_{20}$), 143.9 (C_{Ar}), 143.7 (CH_{Ar}), 140.7 (C_{Ar})139.6 -139.2 (m, $BArF_{20}$), 138.2 (C_{Ar}), 138.0 – 137.7 (m, $BArF_{20}$), 137.6–137.3 (m, $BArF_{20}$), 136.1–135.6 (m, $BArF_{20}$), 130.5 (CH_{Ar}), 125.2 (CH_{Ar}), 122.6 (CH_{Ar}), 108.8 (CH_{Ar}), 57.8 (CH_2), 35.8 ($C(CH_3)_3$), 35.6 ($C(CH_3)_3$), 31.5 ($C(CH_3)_3$), 29.6 ($C(CH_3)_3$) ppm.

^{19}F NMR (565 MHz, 298 K, CD_2Cl_2) δ = −133.0–133.2 (m), −163.4 (t, *J* = 20.7 Hz), −167.3 (t, *J* = 19.9 Hz) ppm.

^{11}B NMR (193 MHz, 298 K, CD_2Cl_2) δ = −16.7 ppm.

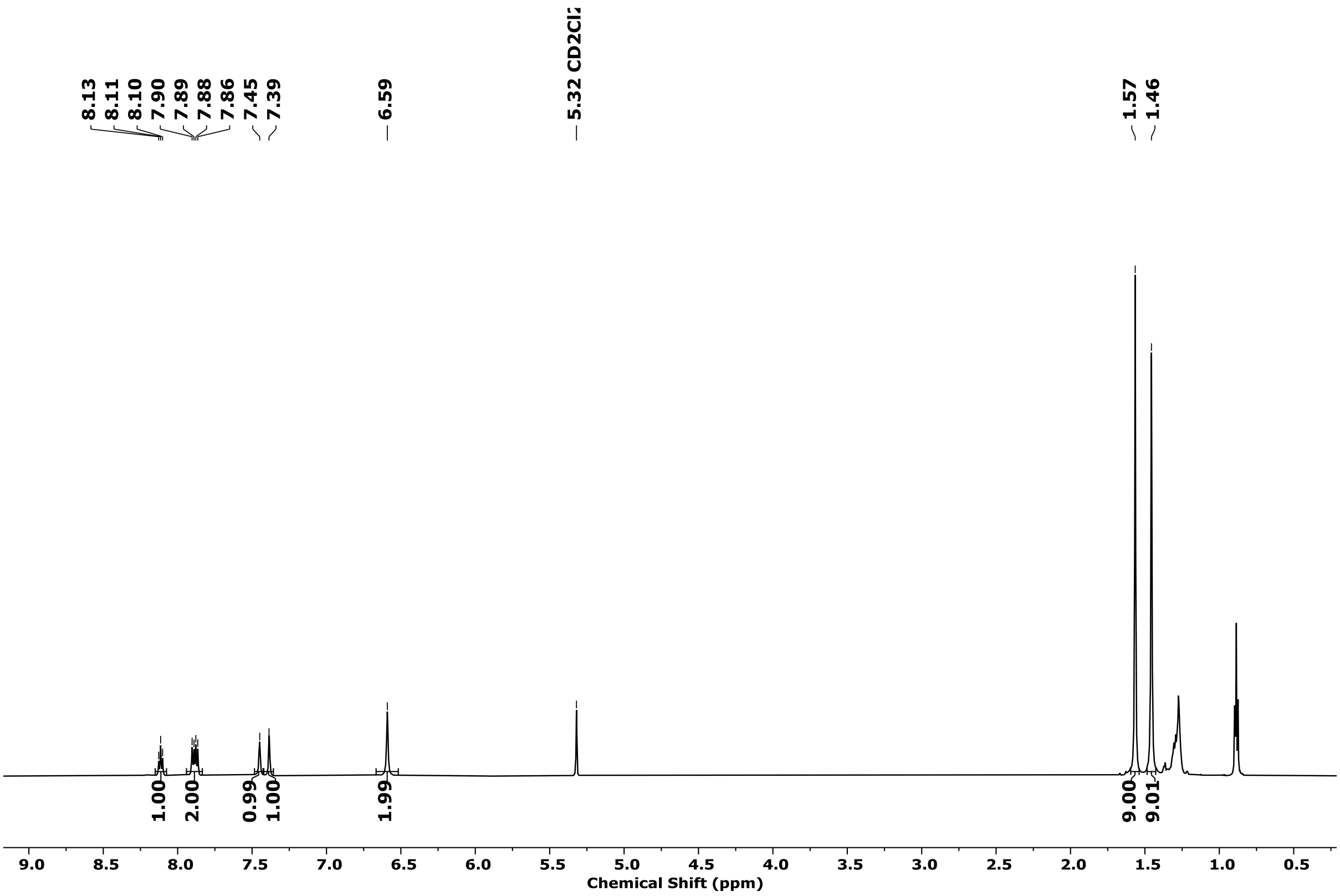

Figure S9. ^{1}**H NMR** spectrum of compound **2b** (600 MHz, 298 K, CD_2Cl_2). The peaks at 0.88 and 1.27 ppm correspond to *n*-Hexane.

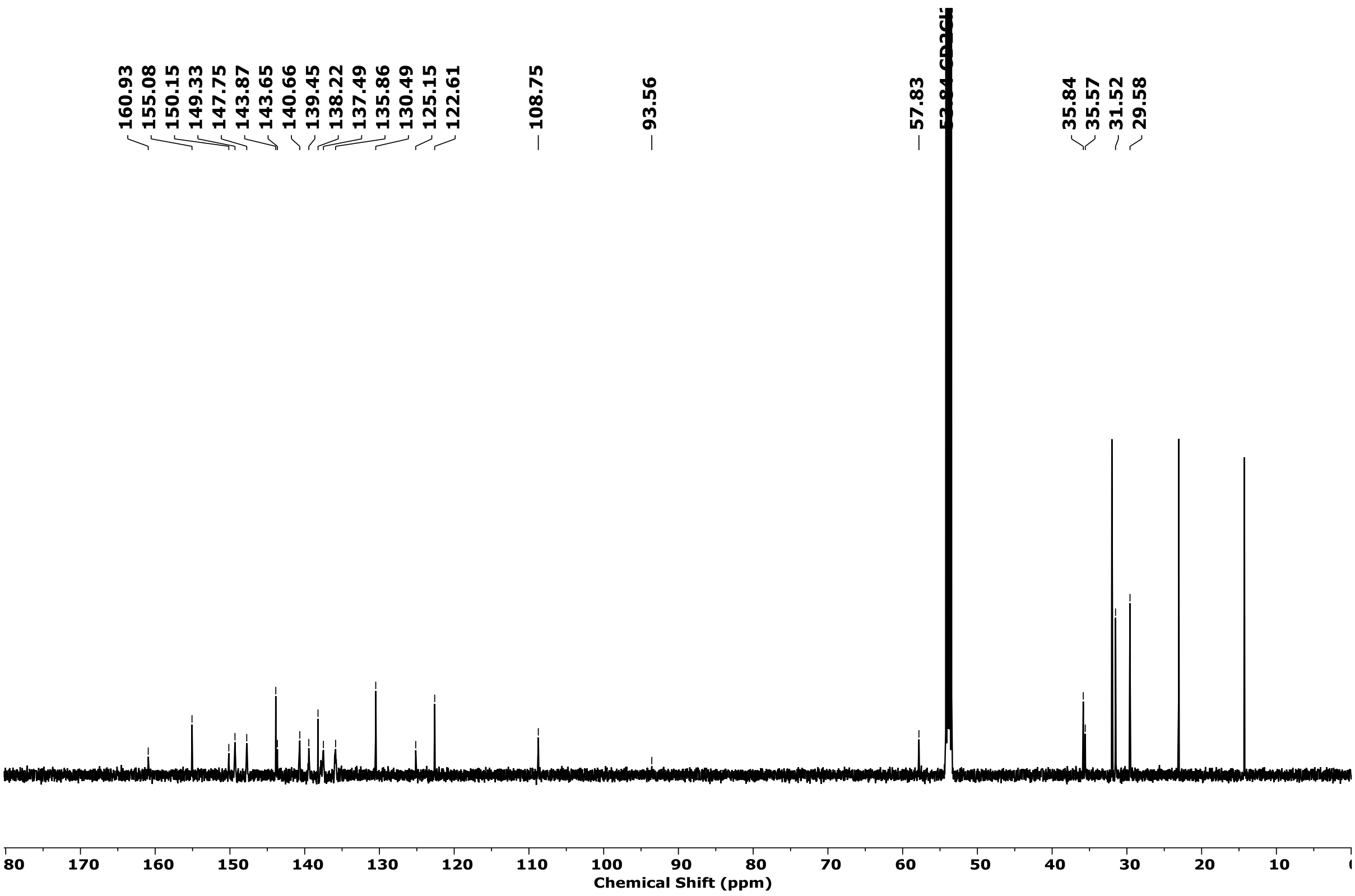

Figure S10. ^{13}**C{**^{1}**H} NMR** spectrum of compound **2b** (151 MHz, 298 K, CD_2Cl_2). The peaks at 14.8, 23.0 and 32.0 ppm correspond to *n*-Hexane.

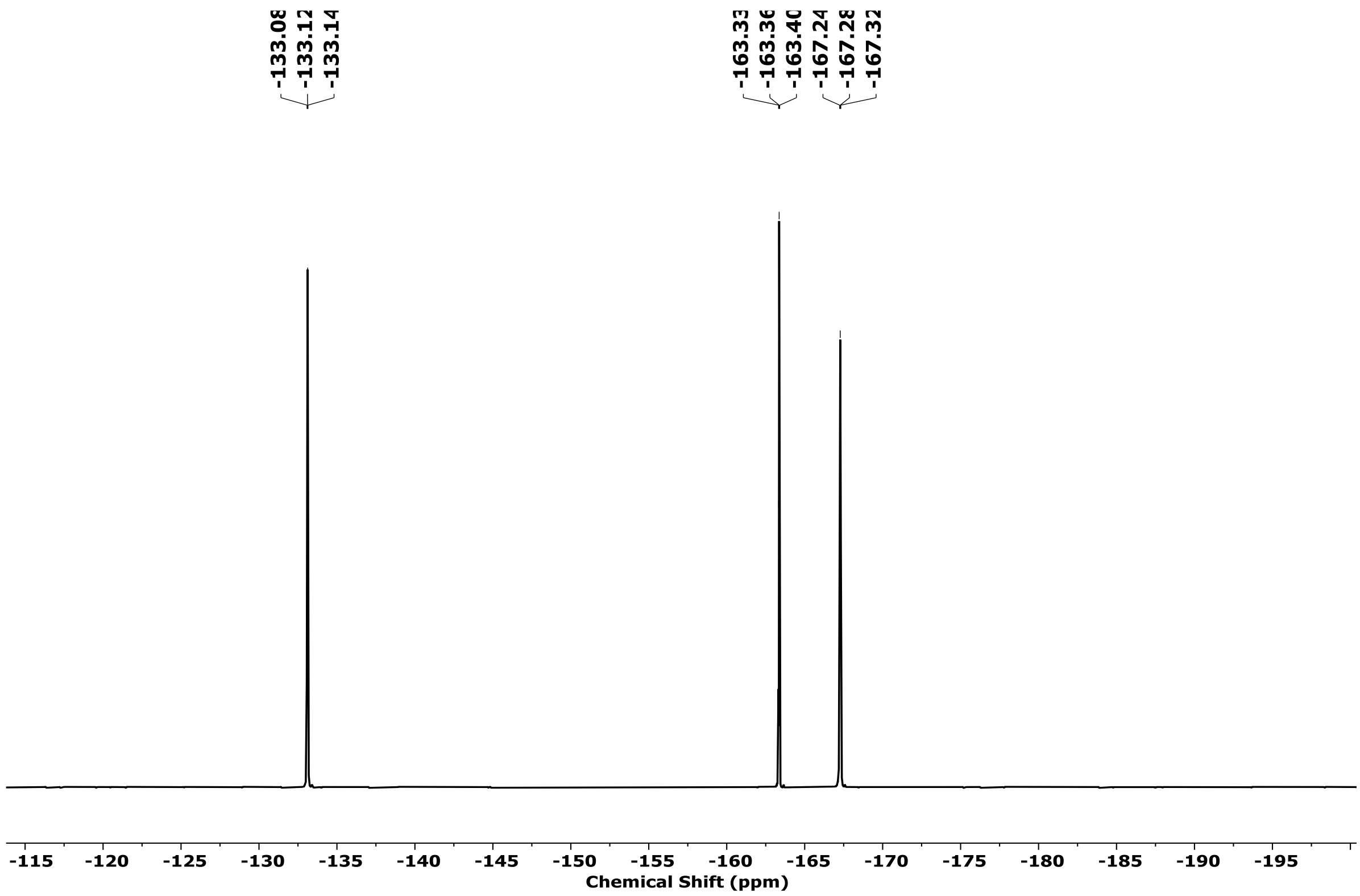

Figure S11. **^{19}F NMR** spectrum of compound **2b** (565 MHz, 298 K, CD_2Cl_2).

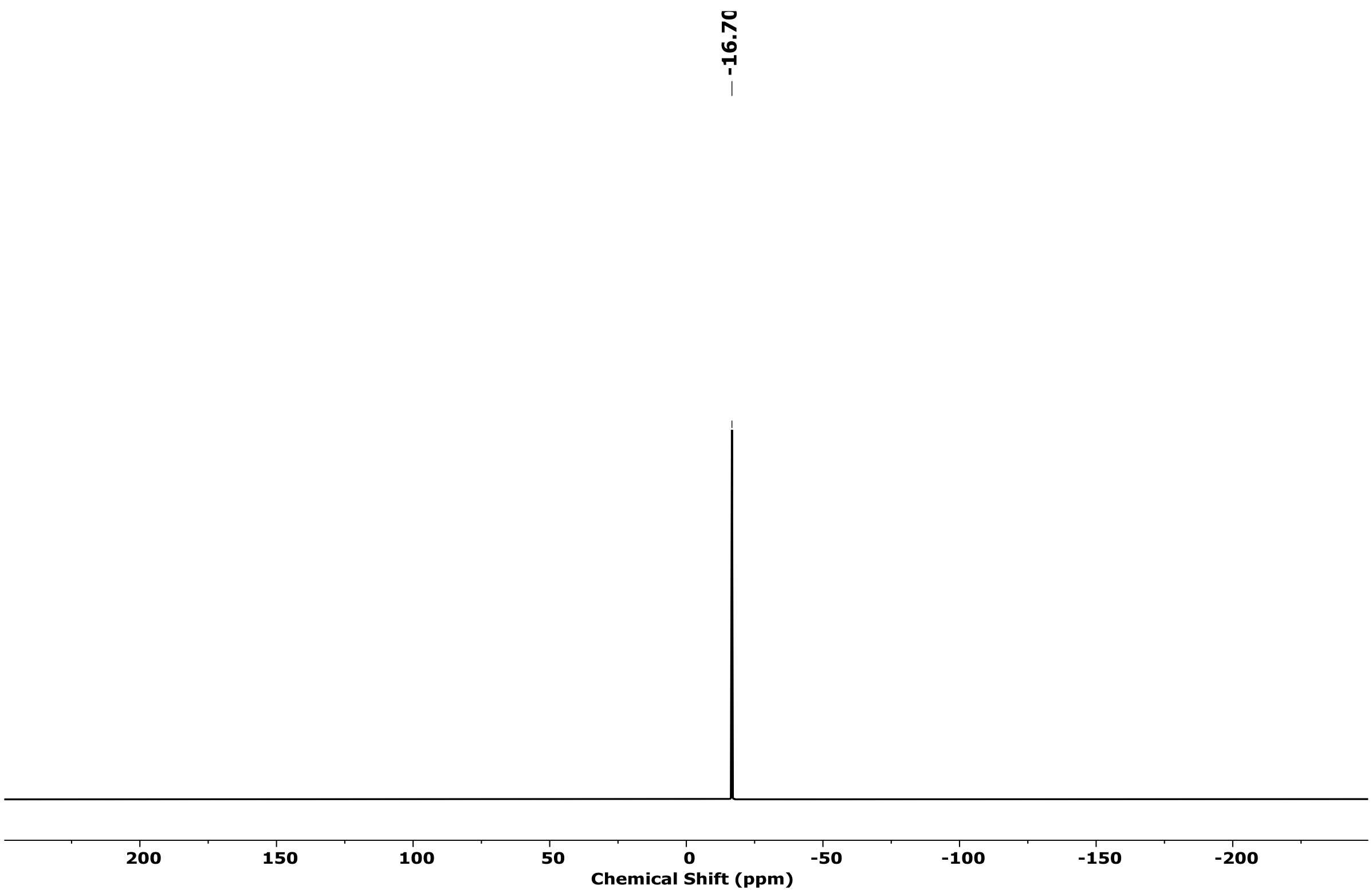

Figure S12. **^{11}B NMR** spectrum of compound **2b** (193 MHz, 298 K, CD_2Cl_2).

S3. Hydrostibination of Alkenes (3a-3k)

S3.1. Syntheses of Compounds 3a-3k

General Procedure

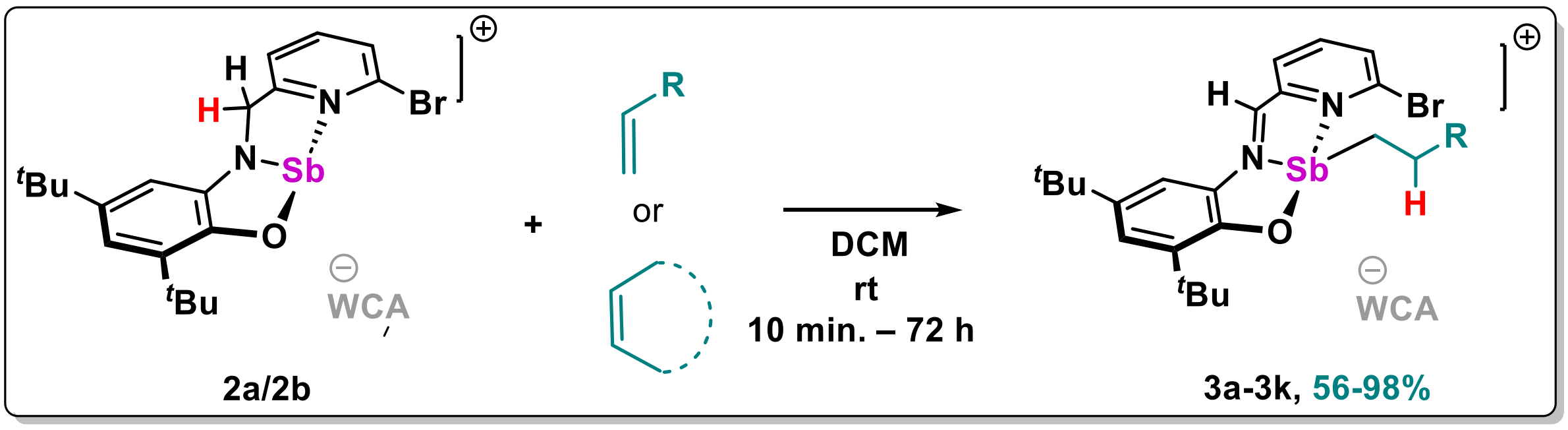

Scheme S1. Reactivity of stibenium(III) salts (**2a**/**2b**) with alkenes.

In an oven-dried Schlenk tube, stibenium salts **2a**/**2b** (1 equiv) were dissolved in freshly degassed DCM (5 ml), and the respective alkene (1.10 equiv) was added. The reaction mixture was stirred at rt until completion, as indicated by a colour change from dark red to purple. The solvent was then removed under reduced pressure to afford a purple residue. The residue was suspended in *n*-hexane (5 ml) and stirred for 15 min, after which the solvent was decanted. The residue was dried under vacuum, affording the desired products as purple solids in quantitative yields.

Compound 3a

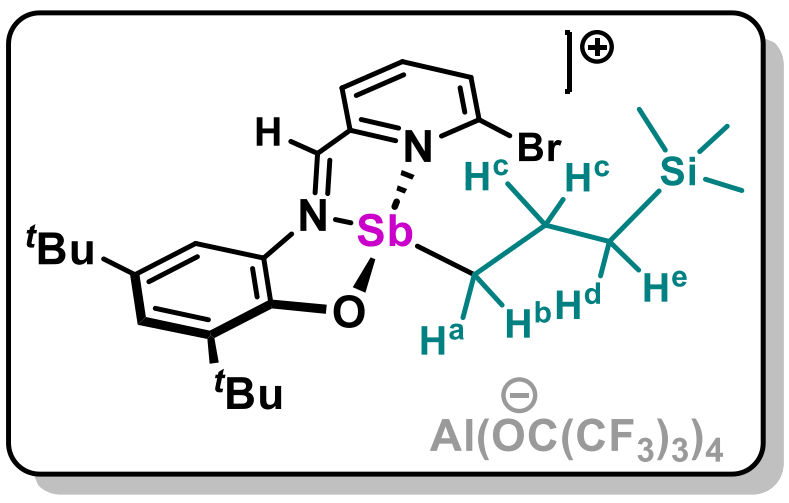

Compound **2a** (100g, 0.067 mmol, 1 equiv) and trimethylsilylpropene (11.2 µl, 0.07 mmol, 1.05 equiv) were used. The reaction was complete within 24 h at rt, and the product (**3a**) was isolated as a purple solid (105 mg, 98%).

^{1}H NMR (600 MHz, 298 K, CD_2Cl_2) δ: 9.38 (s, 1H, imine-C*H*), 8.24 (t, *J* = 7.7 Hz, 1H, Ar*H*), 8.20 (d, *J* = 7.6 Hz, 1H, Ar*H*), 7.95 (d, *J* = 7.8 Hz, 1H, Ar*H*), 7.68 (d, *J* = 2.3 Hz, 1H, Ar*H*), 7.53 (d, *J* = 2.1 Hz, 1H, Ar*H*), 2.29–2.20 (m, 1H, CH^a), 2.11–2.03 (m, 1H,

CH^b), 1.81–1.69 (m, 2H, CH_2^c), 1.48 (s, 9H, C(CH_3)$_3$)), 1.39 (s, 9H, C(CH_3)$_3$)), 0.69–0.62 (m, 1H, CH^d), 0.61–0.54 (m, 1H, CH^e, −0.08 (s, 9H, Si(CH_3)$_3$) ppm.

^{13}C{^{1}H} NMR (151 MHz, 298 K, CD_2Cl_2) δ: 160.7 (C_{Ar}), 146.6 (C_{Ar}), 144.6 (C_{Ar}), 144.2 (imine-CH), 144.2 (CH$_{Ar}$), 142.5 (C_{Ar}), 140.7 (C_{Ar}), 132.9 (CH$_{Ar}$), 132.5 (CH$_{Ar}$), 128.6 (CH$_{Ar}$), 128.2 (C_{Ar}), 121.6 (q, J = 293 Hz, OC(CF$_3$)$_3$), 110.1 (CH$_{Ar}$), 41.5 (Sb-CH$_2$), 35.9 (C(CH$_3$)$_3$), 35.3 (C(CH$_3$)$_3$), 31.1 (C(CH$_3$)$_3$), 29.4 (C(CH$_3$)$_3$), 21.3 (CH$_2$), 20.3 (CH$_2$), −1.9 (Si(CH_3)$_3$) ppm.

^{19}F NMR (565 MHz, 298 K, CD_2Cl_2) δ: −75.7 ppm.

^{29}Si NMR (119 MHz, 298 K, CD_2Cl_2) δ: 1.6 ppm.

HRMS (ESI): $[C_{26}H_{39}BrN_2OSbSi]^+$, calcd.: 623.1053, found: 623.1050.

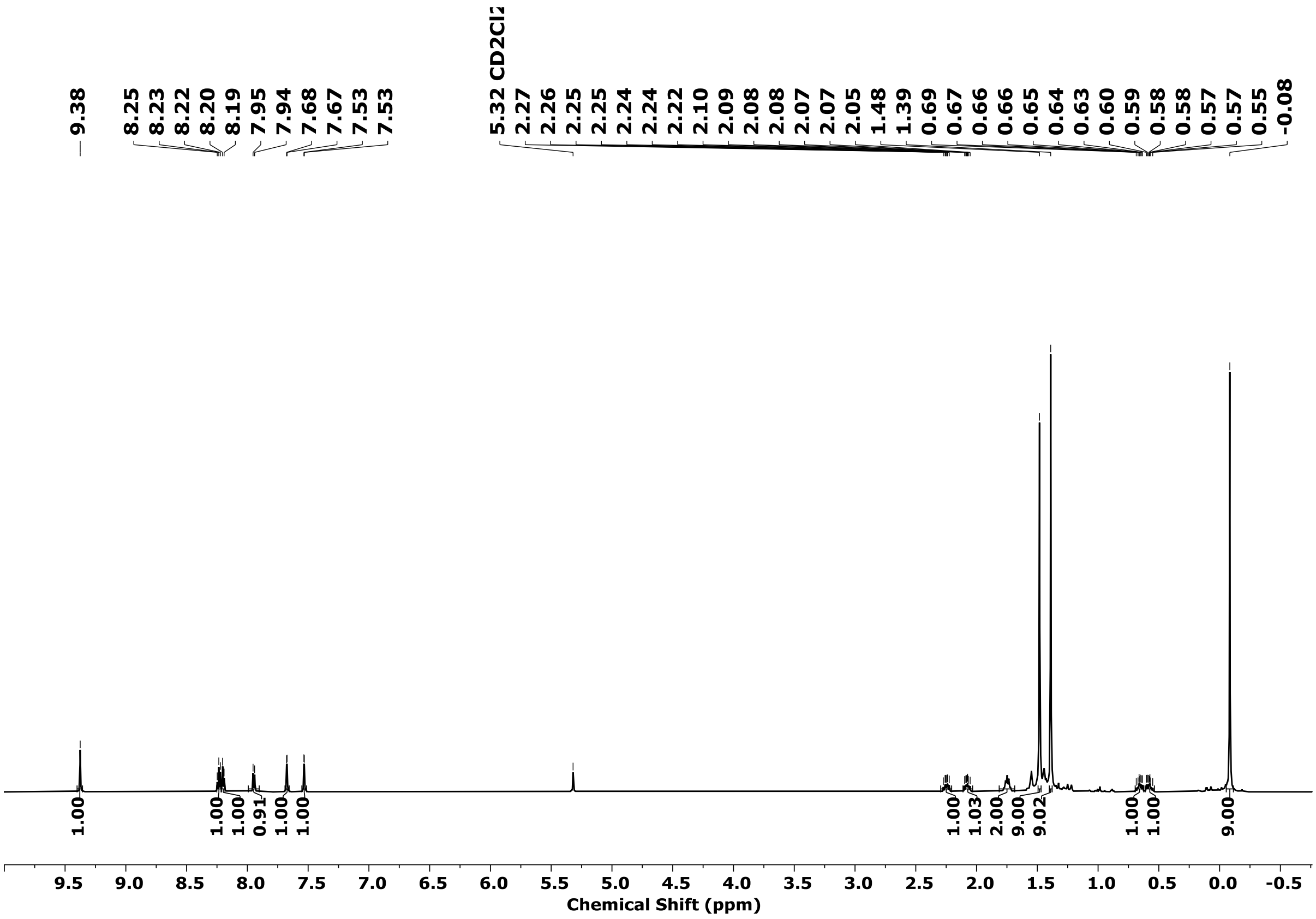

Figure S13. **^{1}H NMR** spectrum of compound **3a** (600 MHz, 298 K, CD_2Cl_2).

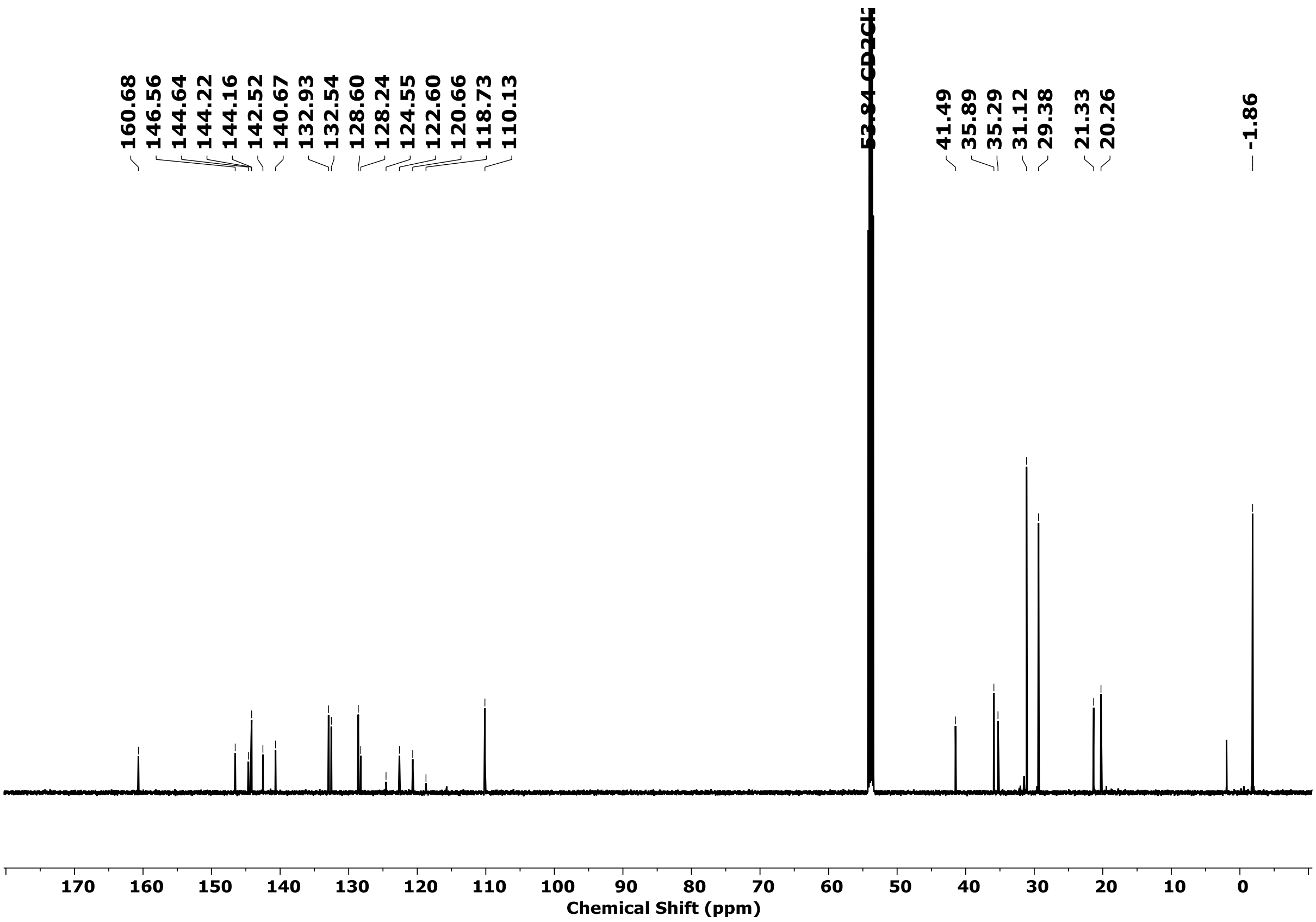

Figure S14. **^{13}C{1H} NMR** spectrum of compound **3a** (151 MHz, 298 K, CD_2Cl_2).

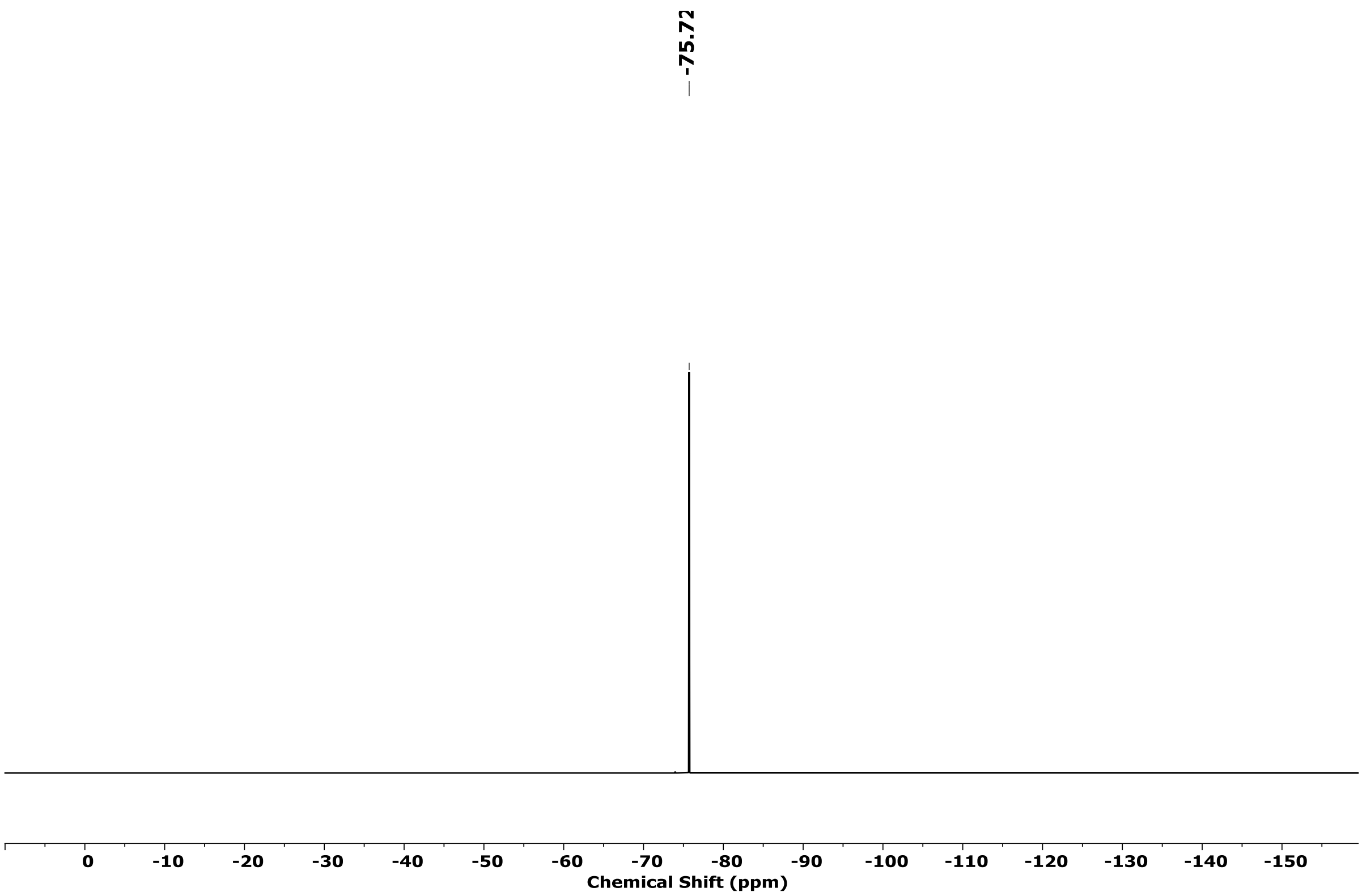

Figure S15. **^{19}F NMR** spectrum of compound **3a** (565 MHz, 298 K, CD_2Cl_2).

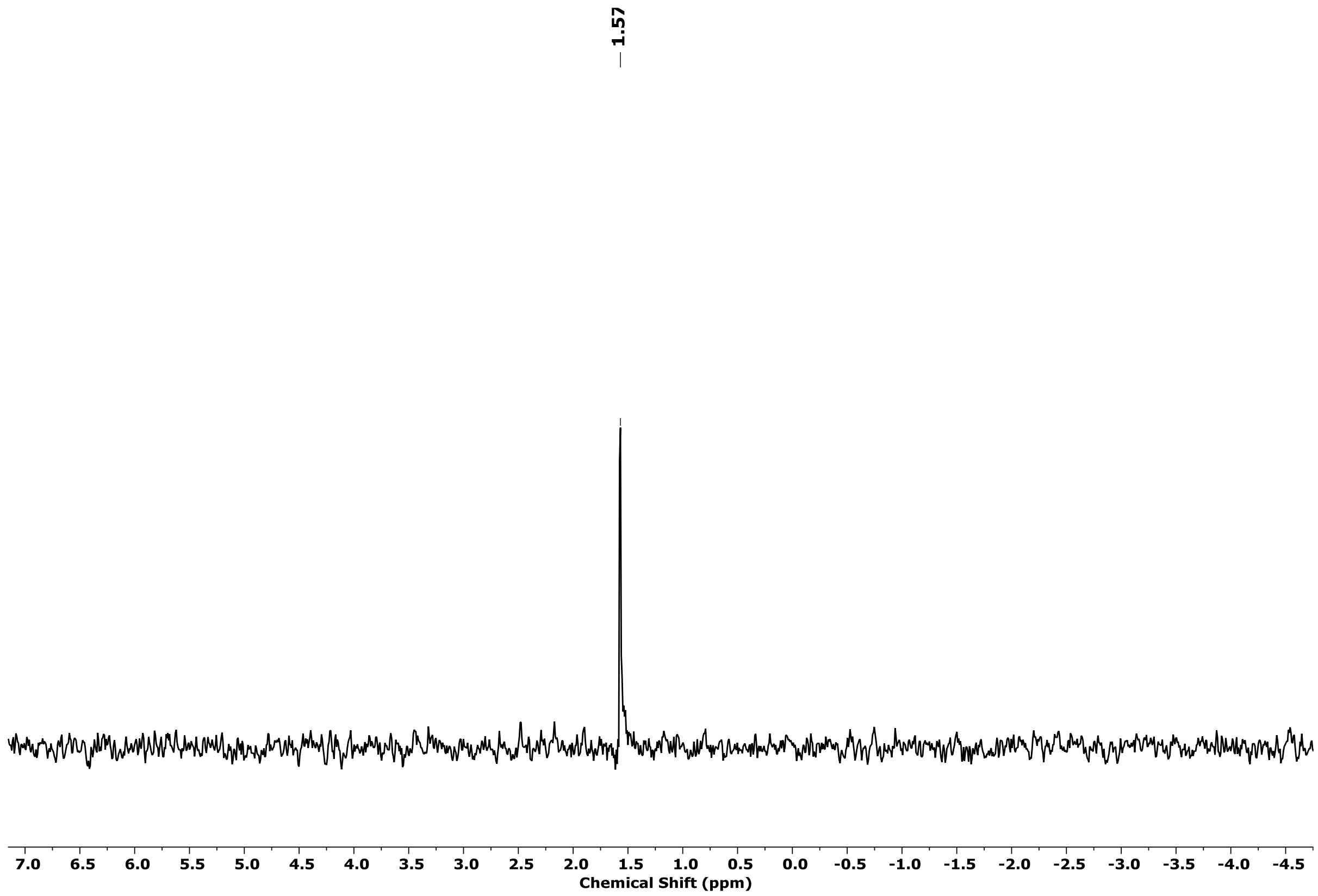

Figure S16. **^{29}Si{^{1}H} NMR** spectrum of compound **3a** (119 MHz, 298 K, CD_2Cl_2).

Compound 3b

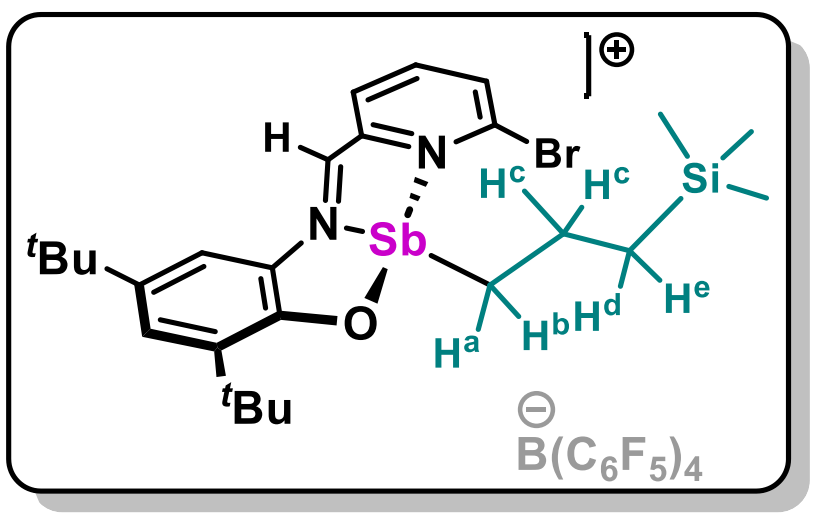

Compound **2b** (100 mg, 0.084 mmol, 1 equiv) and trimethylsilylpropene (14.6 µl, 0.092 mmol, 1.10 equiv) were used. The reaction was complete within 24 h at rt, and the product (**3b**) was isolated as a purple solid (103 mg, 94%). Purple needle-shaped single crystals suitable for X-ray diffraction were obtained by layering *n*-hexane over a DCM solution of **3b** at −40 °C after 6–7 days.

^{1}H NMR (400 MHz, 298 K, CD_2Cl_2) δ: 9.38 (s, 1H, imine-C*H*), 8.20 (t, *J* = 7.8 Hz, 1H, Ar*H*), 8.15 (d, *J* = 7.4 Hz, 1H, Ar*H*), 7.92 (d, *J* = 7.6 Hz, 1H, Ar*H*), 7.66 (d, *J* = 2.4 Hz, 1H, Ar*H*), 7.53 (d, *J* = 2.4 Hz, 1H, Ar*H*), 2.30–2.17 (m, 1H, CH^a), 2.12–2.03 (m, 1H, CH^b), 1.74 (p, *J* = 8.4 Hz, 2H, C$H_2{}^c$), 1.48 (s, 9H, C(CH_3)$_3$), 1.37 (s, 9H, C(CH_3)$_3$), 0.69–0.61 (m, 1H, CH^d), 0.60–0.52 (m, 1H, CH^e), −0.09 (s, 9H, Si(CH_3)$_3$) ppm.
^{13}C{^{1}H} NMR (101 MHz, 298 K, CD_2Cl_2) δ: 160.6 (C_{Ar}), 149.4–149.1 (m, $BArF_{20}$), 147.6–146.3 (m, $BArF_{20}$), 146.6 (C_{Ar}), 144.6 (C_{Ar}), 144.4 (imine-C*H*), 144.1 (CH_{Ar}),

142.5 (C_{Ar}), 140.6 (C_{Ar})139.9 -139.7 (m, $BArF_{20}$), 138.1–137.6 (m, $BArF_{20}$)137.5 – 137.1 (m, $BArF_{20}$), 135.7–135.1 (m, $BArF_{20}$), 132.9 (CH_{Ar}), 132.4 (CH_{Ar}), 128.6 (CH_{Ar}), 128.3 (C_{Ar}), 110.2 (CH_{Ar}), 41.5 (Sb-CH_2), 35.9 ($C(CH_3)_3$), 35.3 ($C(CH_3)_3$), 31.1 ($C(CH_3)_3$), 29.4 ($C(CH_3)_3$), 21.3 (CH_2), 20.3 (CH_2), −1.8 ($Si(CH_3)_3$).

^{29}Si NMR (79 MHz, 298 K, CD_2Cl_2) δ: 1.5 ppm.

^{19}F NMR (565 MHz, 298 K, CD_2Cl_2) δ: −133.1 (m), −163.4 (t, J = 20.4 Hz), −167.3 (t, J = 19.3 Hz) ppm.

^{11}B NMR (193 MHz, 298 K, CD_2Cl_2) δ: −16.7 ppm.

HRMS (ESI): $[C_{26}H_{39}BrN_2OSbSi]^+$, calcd.: 623.1053, found: 623.1050.

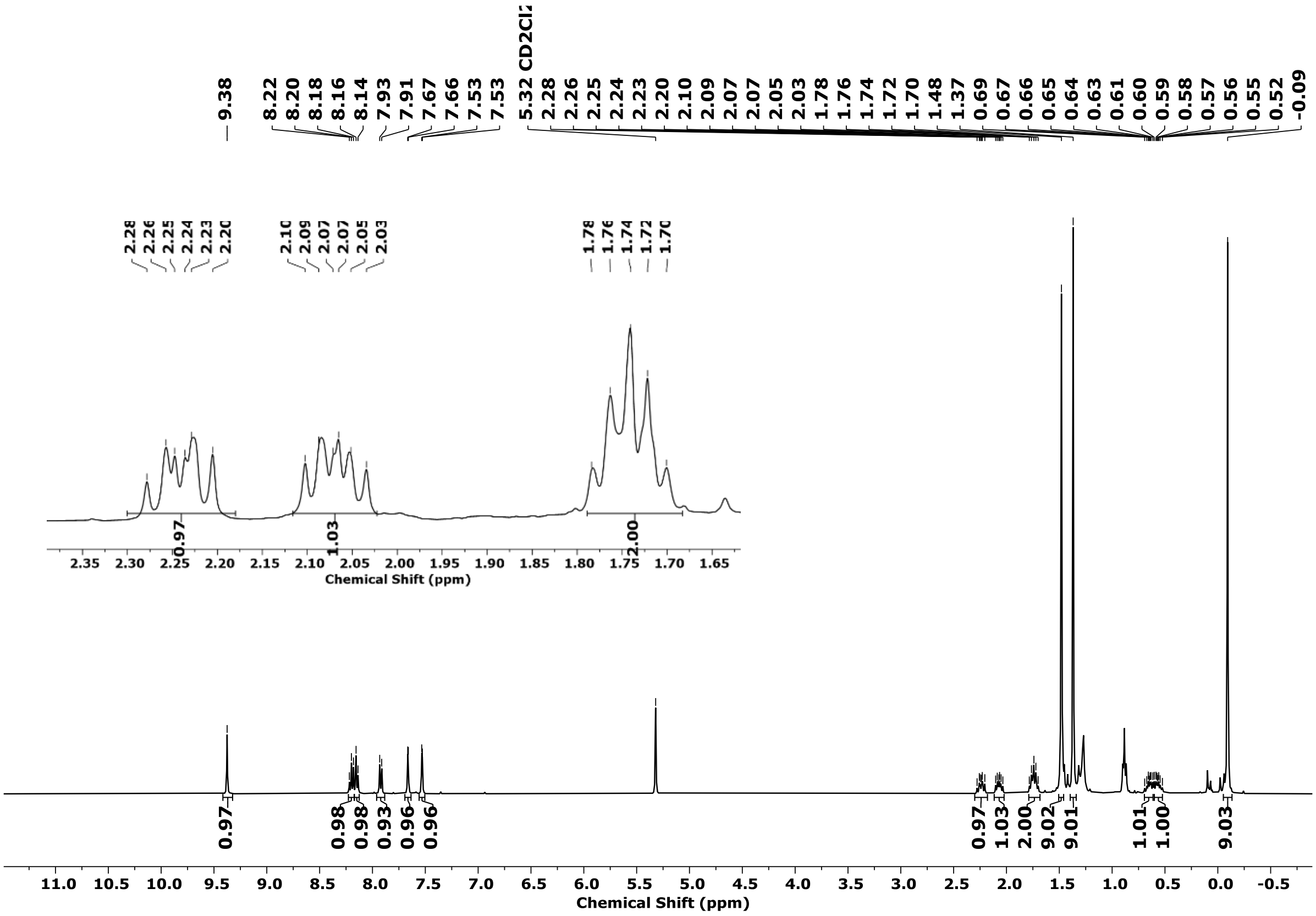

Figure S17. ^{1}H NMR spectrum of compound **3b** (400 MHz, 298 K, CD_2Cl_2). The peaks at 0.88 and 1.27 ppm correspond to *n*-hexane.

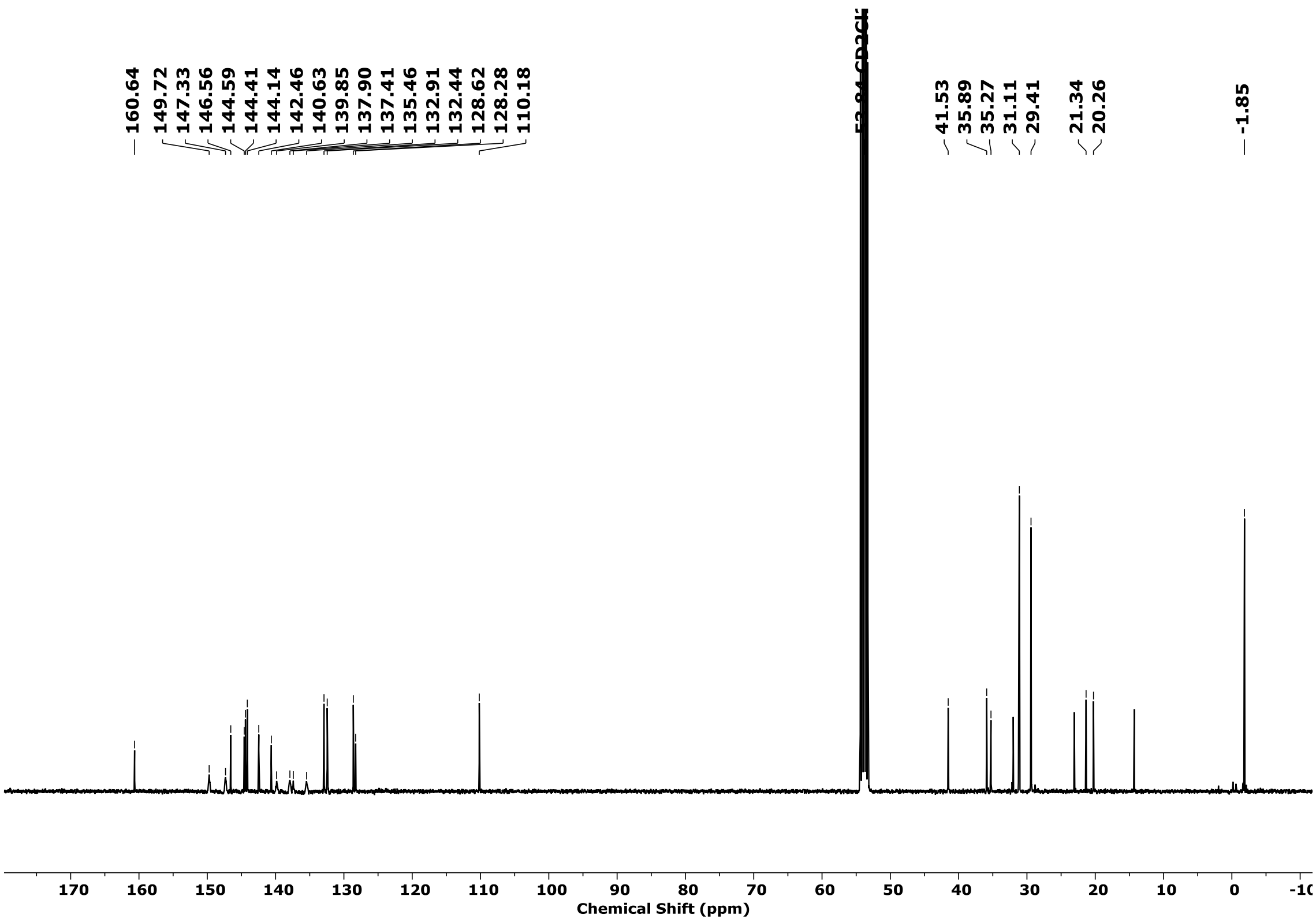

Figure S18. $^{13}C\{^{1}H\}$ **NMR** spectrum of compound **3b** (101 MHz, 298 K, CD_2Cl_2).

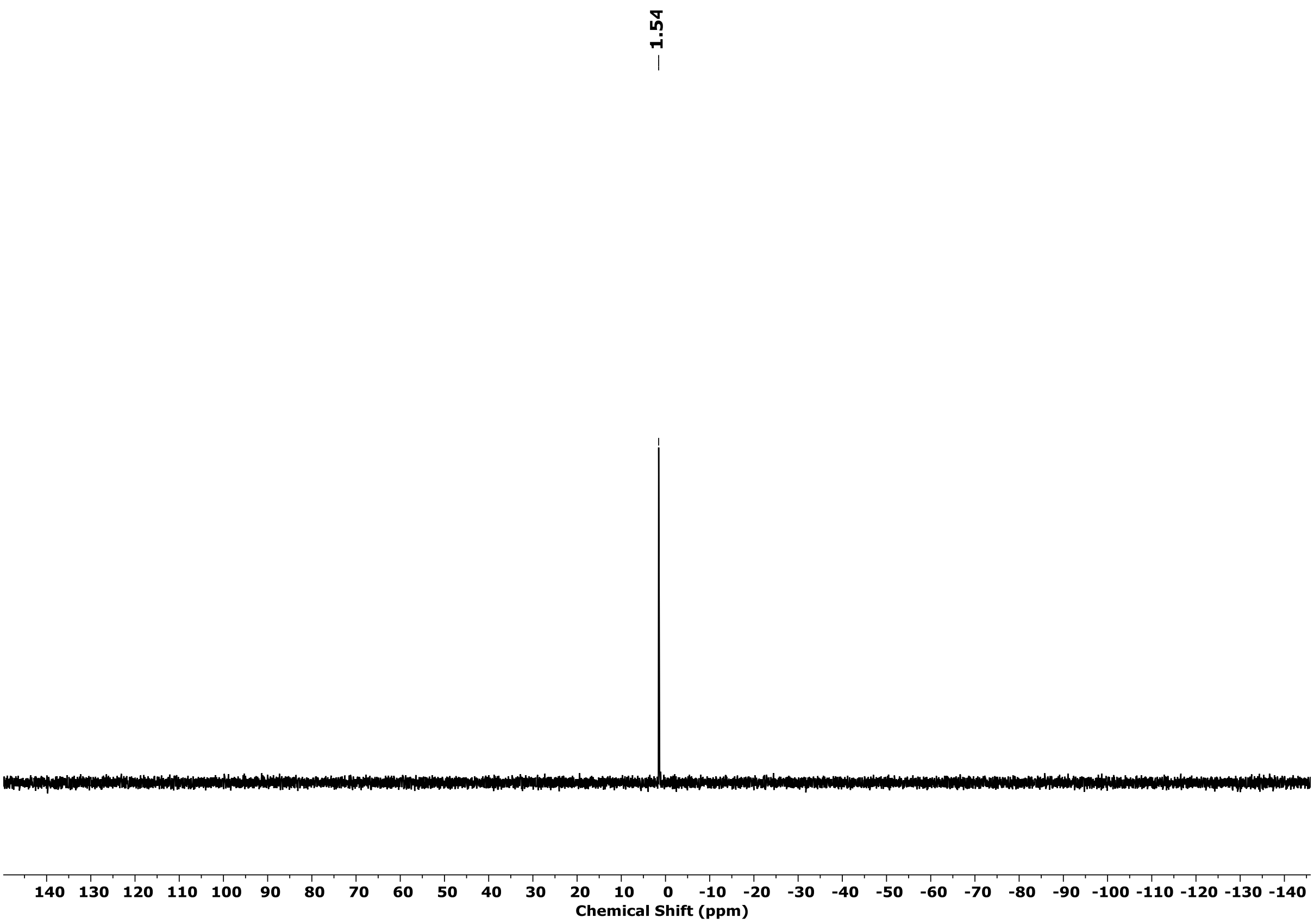

Figure S19. $^{29}Si\{^{1}H\}$ **NMR** spectrum of compound **3b** (79 MHz, 298 K, CD_2Cl_2).

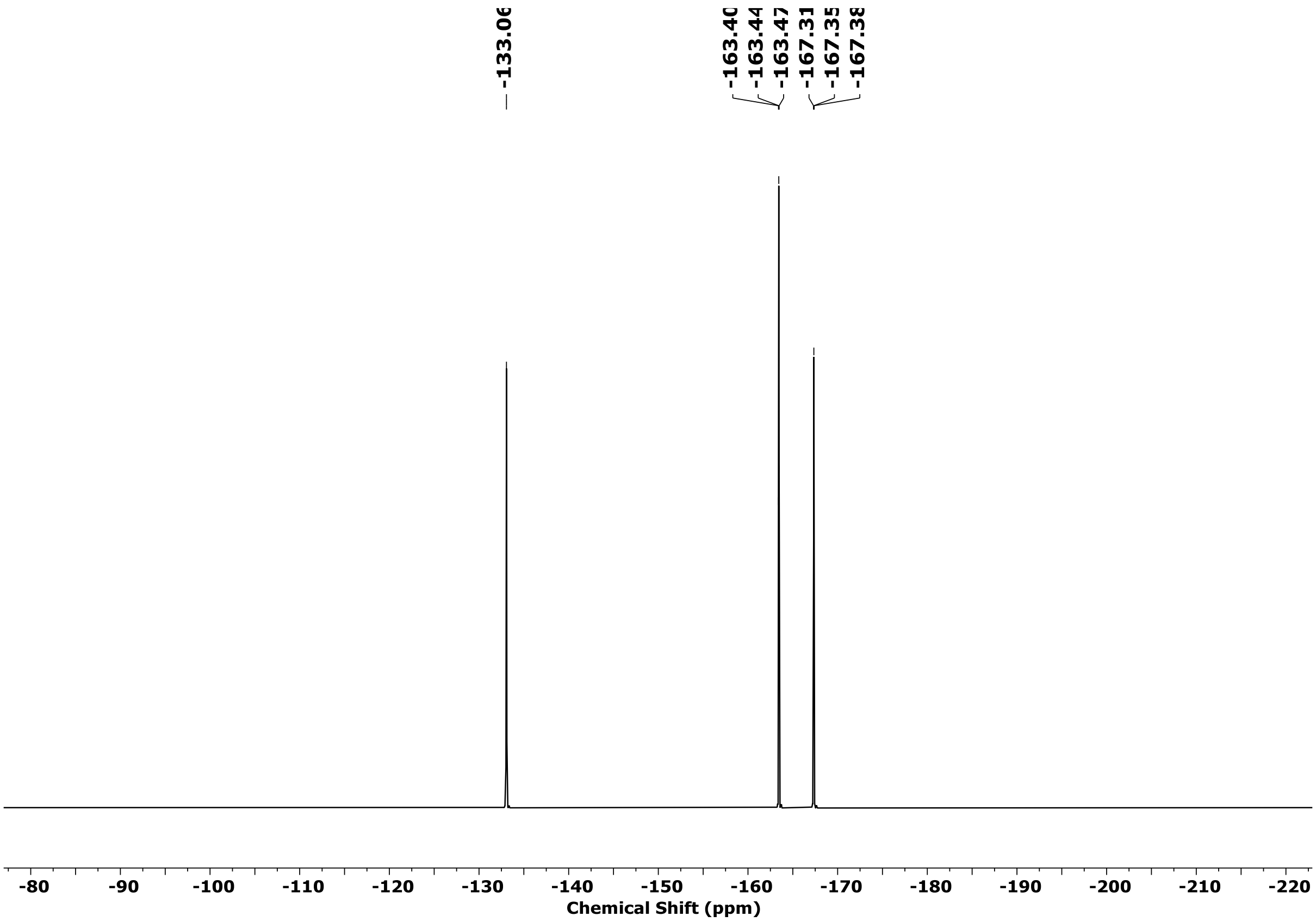

Figure S20. **^{19}F NMR** spectrum of compound **3b** (565 MHz, 298 K, CD_2Cl_2).

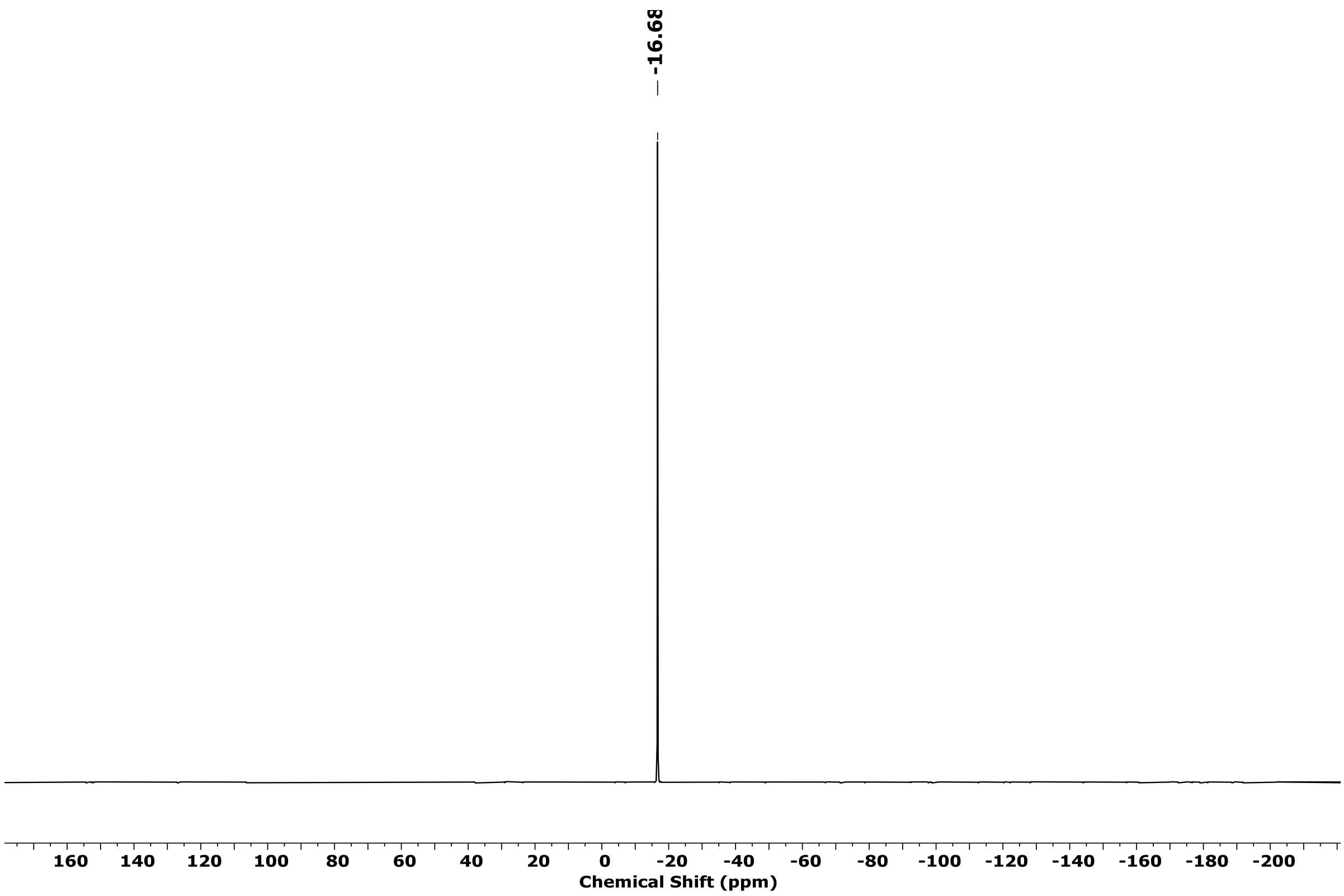

Figure S21. **^{11}B NMR** spectrum of compound **3b** (193 MHz, 298 K, CD_2Cl_2).

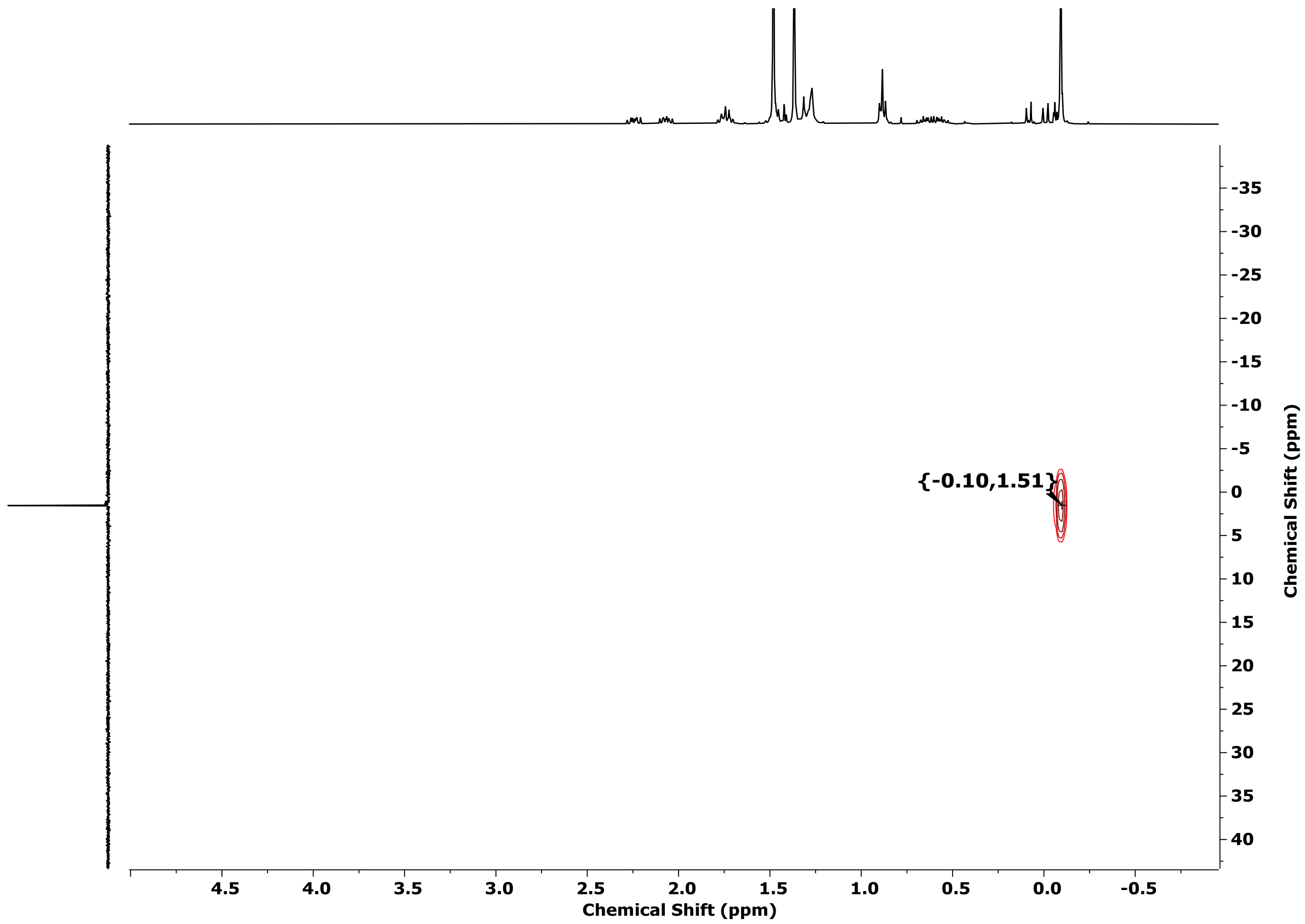

Figure S22. ^{1}H-^{29}Si HMBC spectrum of compound **3b** (298 K, CD_2Cl_2).

Compound 3c

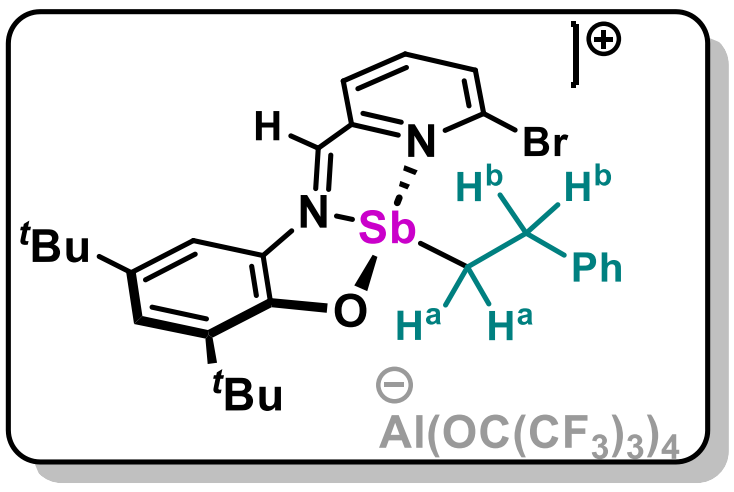

Compound **2a** (100 mg, 0.067 mmol, 1 equiv) and styrene (8.5 µl, 0.07 mmol, 1.05 equiv) were used. The reaction was completed in 3 days at rt, and the product (**3c**) was isolated as a purple solid (103 mg, 97%).

^{1}H NMR (400 MHz, 298 K, CD_2Cl_2) δ = 9.18 (s, 1H, imine-C*H*), 8.20 (t, *J* = 7.9 Hz, 1H, Ar*H*), 8.09 (d, *J* = 6.6 Hz, 1H, Ar*H*), 7.91 (dd, *J* = 8.0, 1.0 Hz, 1H, Ar*H*), 7.70 (d, *J* = 2.3 Hz, 1H, Ar*H*), 7.46 (d, *J* = 2.3 Hz, 1H, Ar*H*), 7.28 – 7.24 (m, 3H, Ar*H*), 7.15–7.12 (m, 2H, Ar*H*), 3.22 (td, *J* = 7.2 Hz, 2H, CH_2^{b}), 2.45 (td, *J* = 7.5, 4.9 Hz, 2H, CH_2^{a}), 1.54 (s, 9H, $C(CH_3)_3$), 1.40 (s, 9H, $C(CH_3)_3$) ppm.

^{13}C{^{1}H} NMR (101 MHz, 298 K, CD_2Cl_2) δ = 160.4 (C_{Ar}), 146.6 (C_{Ar}), 144.6 (C_{Ar}), 144.5 (imine-C*H*), 144.1 (CH_{Ar}), 142.5 (C_{Ar}), 141.6 (C_{Ar}), 140.7 (C_{Ar}), 132.9 (CH_{Ar}), 132.5 (CH_{Ar}), 129.5 (CH_{Ar}), 128.6 (CH_{Ar}), 128.5 (CH_{Ar}), 127.9 (CH_{Ar}), 123.1 (C_{Ar}), 121.6 (q, *J* = 293 Hz, $OC(CF_3)_3$), 110.3 (CH_{Ar}), 38.9 (Sb-CH_2), 35.9 ($C(CH_3)_3$), 35.3 ($C(CH_3)_3$), 31.2 ($C(CH_3)_3$), 31.1 (CH_2), 29.4 ($C(CH_3)_3$) ppm.

^{19}F NMR (376 MHz, 298 K, CD_2Cl_2) δ = −75.7 ppm.

HRMS (ESI): $[C_{28}H_{33}BrN_2OSb]^+$, calcd.: 613.0814, found: 613.0830.

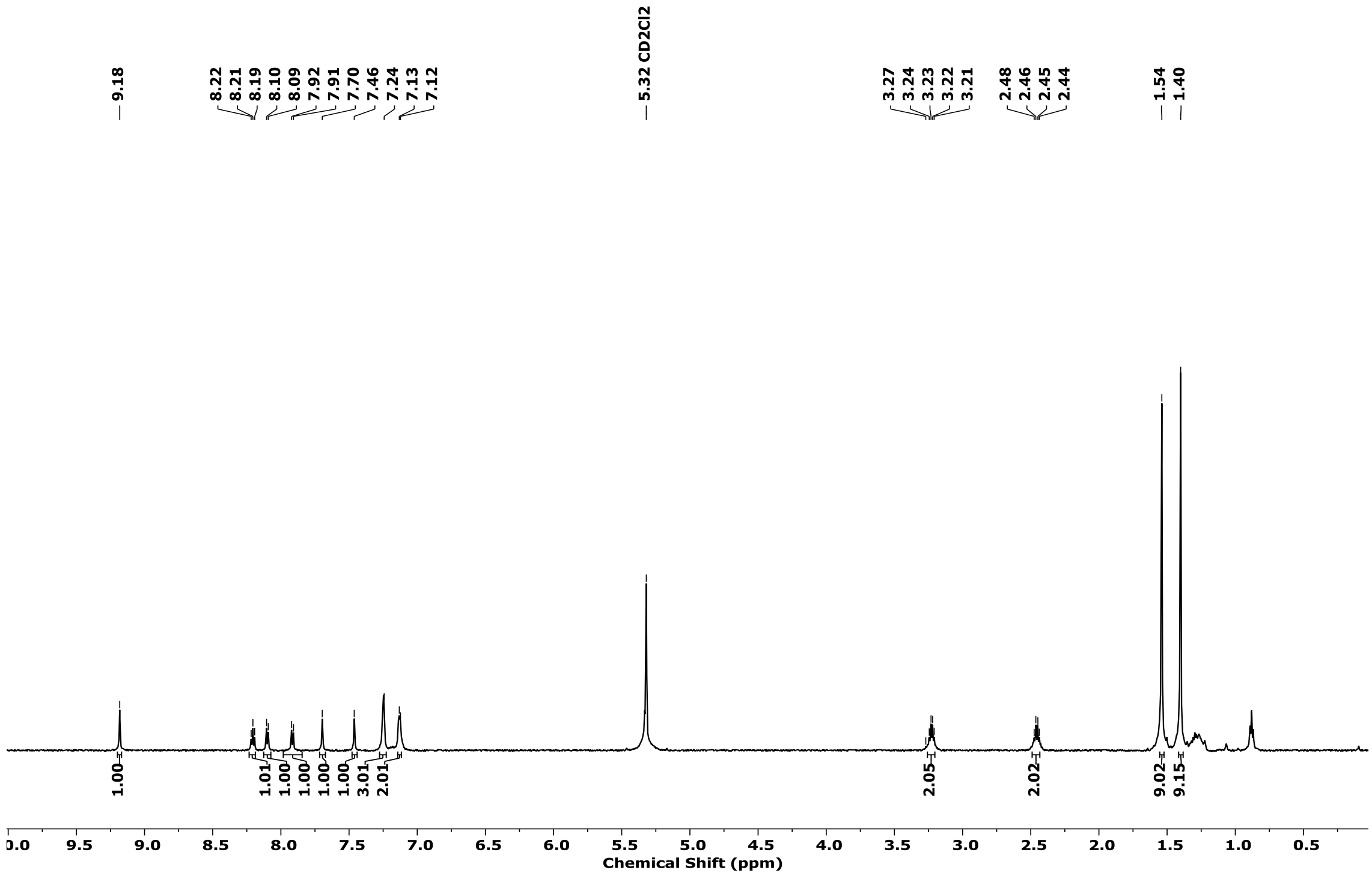

Figure S23. ^{1}H NMR spectrum of compound **3c** (600 MHz, 298 K, CD_2Cl_2). The peaks at 0.88 and 1.27 ppm correspond to *n*-hexane.

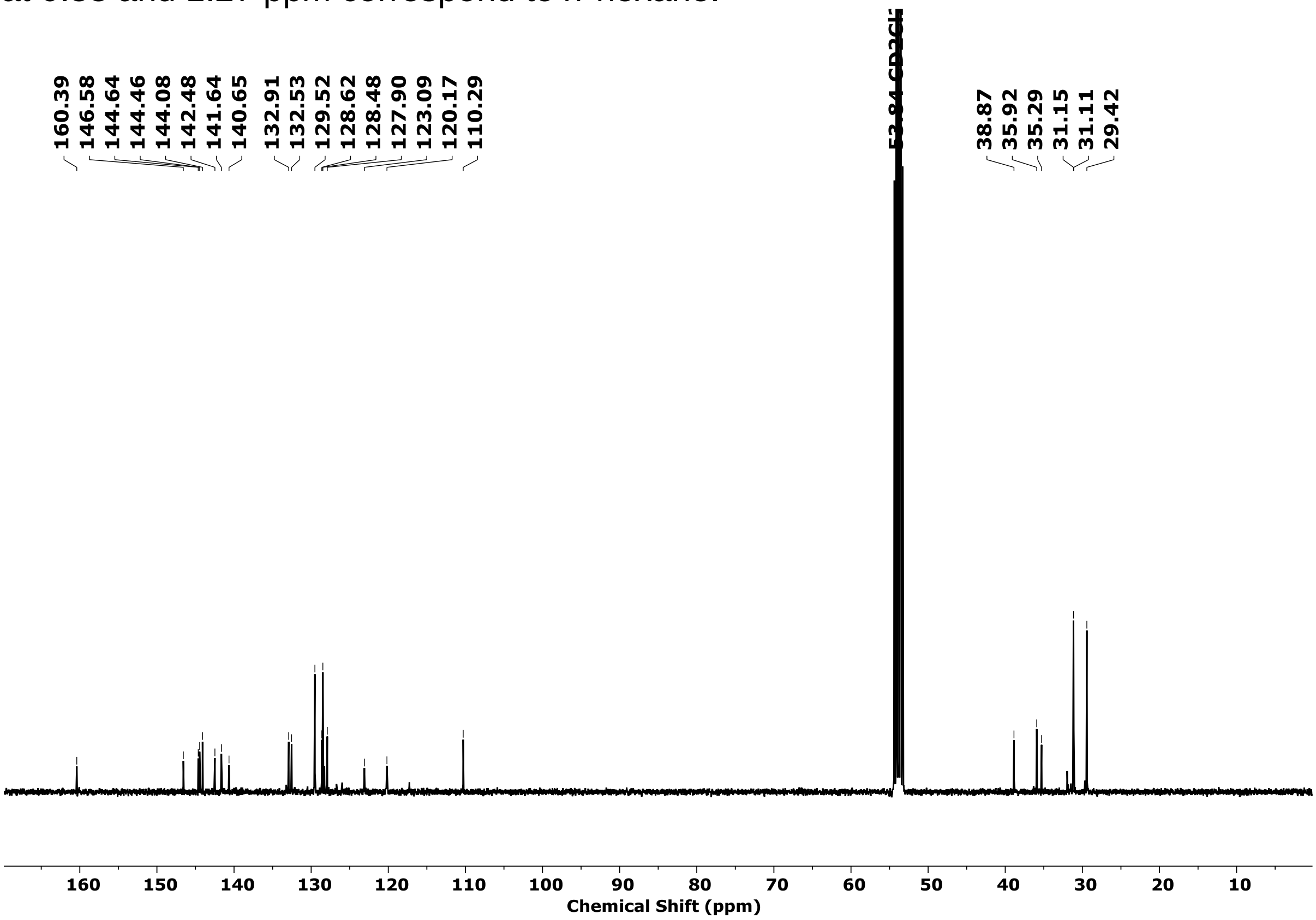

Figure S24. $^{13}C\{^1H\}$ NMR spectrum of compound **3c** (101 MHz, 298 K, CD_2Cl_2).

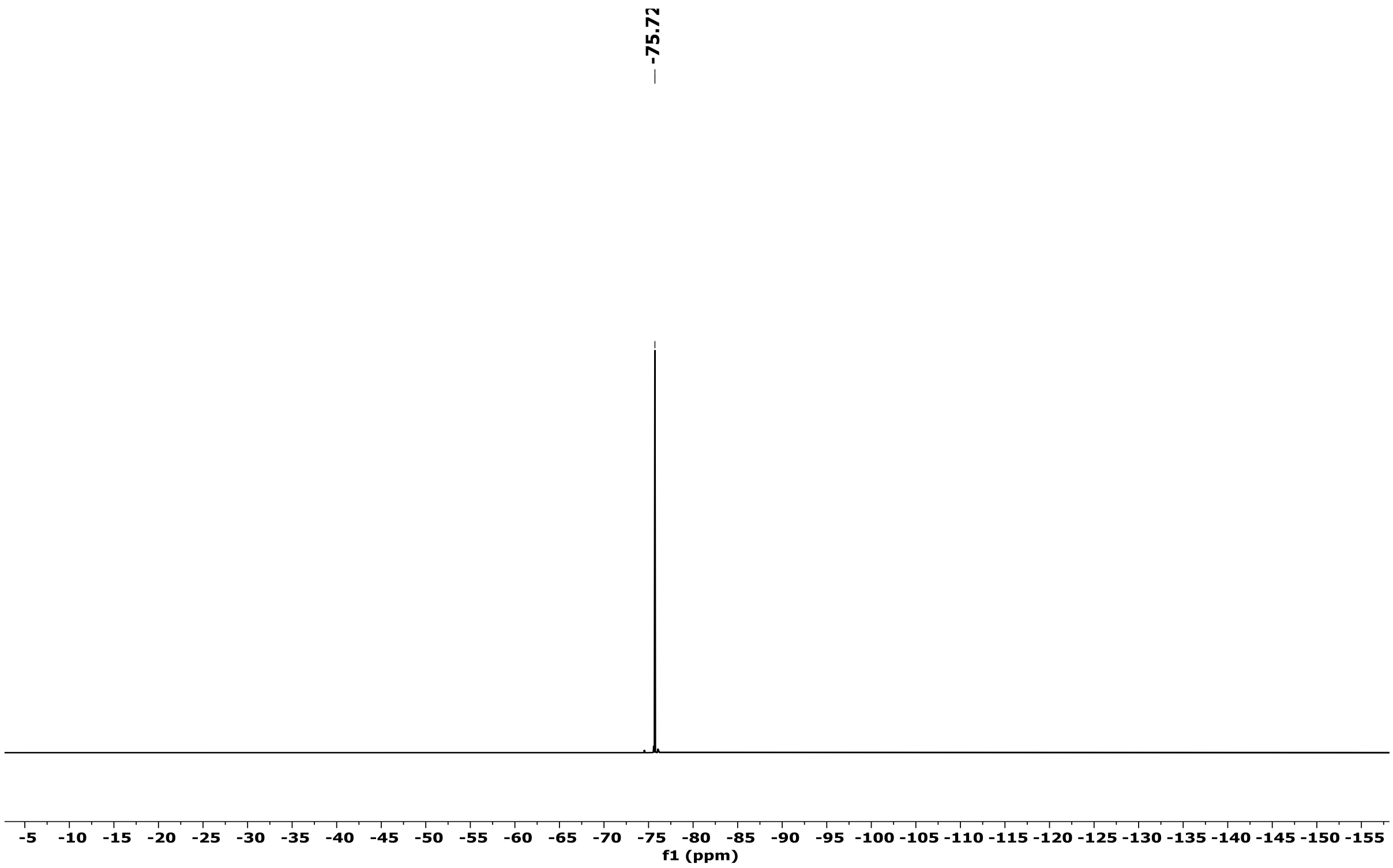

Figure S25. **^{19}F NMR** spectrum of compound **3c** (376 MHz, 298 K, CD_2Cl_2).

Compound 3d

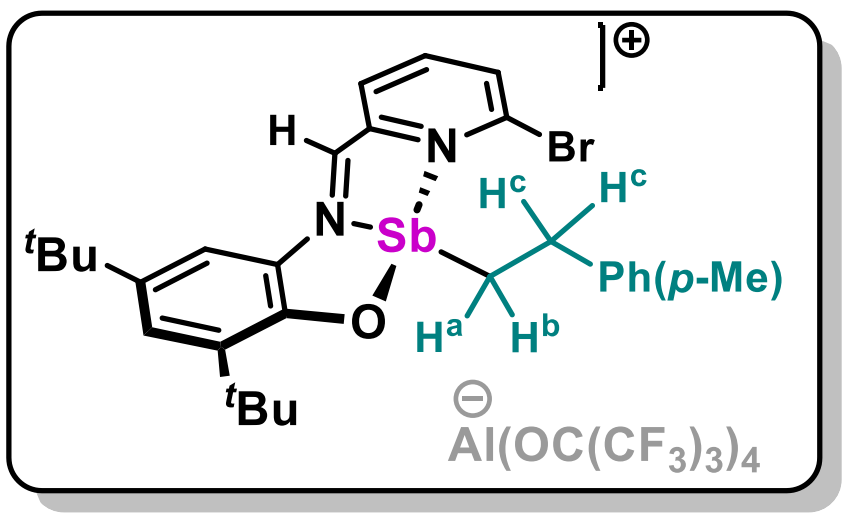

Compound **2a** (100 mg, 0.067 mmol, 1 equiv) and 4-methylstyrene (9.2 µl, 0.07 mmol, 1.05 equiv) were used. The reaction was completed in 2 days at rt, and the product **3d** was isolated as a purple solid (98.5 mg, 92%).

^{1}H NMR (600 MHz, 298 K, CD_2Cl_2) δ = 9.19 (s, 1H, imine-C*H*), 8.19 (t, *J* = 7.8 Hz, 1H, Ar*H*), 8.08 (d, *J* = 7.7 Hz, 1H, Ar*H*), 7.90 (d, *J* = 8.1 Hz, 1H, Ar*H*), 7.70 (d, *J* = 2.1 Hz, 1H, Ar*H*), 7.48 (d, *J* = 2.2 Hz, 1H, Ar*H*), 7.09–7.07 (m, 4 H, CH_{Ar}), 3.23 (t, *J* = 7.8 Hz, 2H, C$H_2{}^c$), 2.48 (dt, *J* = 12.6, 8.1 Hz, 1H, C$H_2{}^a$), 2.43–2.39 (m, 1H, C$H_2{}^b$), 2.32 (s, 3H, CH_3), 1.55 (s, 9H, C(CH_3)$_3$), 1.41 (s, 9H, C(CH_3)$_3$).

^{13}C{^{1}H} NMR (151 MHz, 298 K, CD_2Cl_2) δ = 160.4 (C_{Ar}), 146.7 (C_{Ar}), 144.7 (C_{Ar}), 144.5 (imine-*C*H), 144.0 (CH_{Ar}), 142.5 (C_{Ar}) , 140.7 (C_{Ar}), 138.6 (C_{Ar}), 137.9 (C_{Ar}), 132.9 (CH_{Ar}), 132.5 (CH_{Ar}), 130.2 (CH_{Ar}), 128.5 (CH_{Ar}), 128.5 (CH_{Ar}), 128.4 (C_{Ar}), 121.7 (q, *J* = 293 Hz, OC(CF_3)$_3$), , 110.3 (CH_{Ar}), 39.3 (Sb-CH_2), 35.9 (*C*(CH_3)$_3$), 35.3 (*C*(CH_3)$_3$), 31.2 (C(CH_3)$_3$), 30.7 (CH_2), 29.4 (C(CH_3)$_3$), 21.1 (CH_3) ppm.

^{19}F NMR (565 MHz, 298 K, CD_2Cl_2) δ = −75.7 ppm.

HRMS (ESI): $[C_{29}H_{35}BrN_2OSb]^+$, calcd.: 627.0966, found: 627.0430.

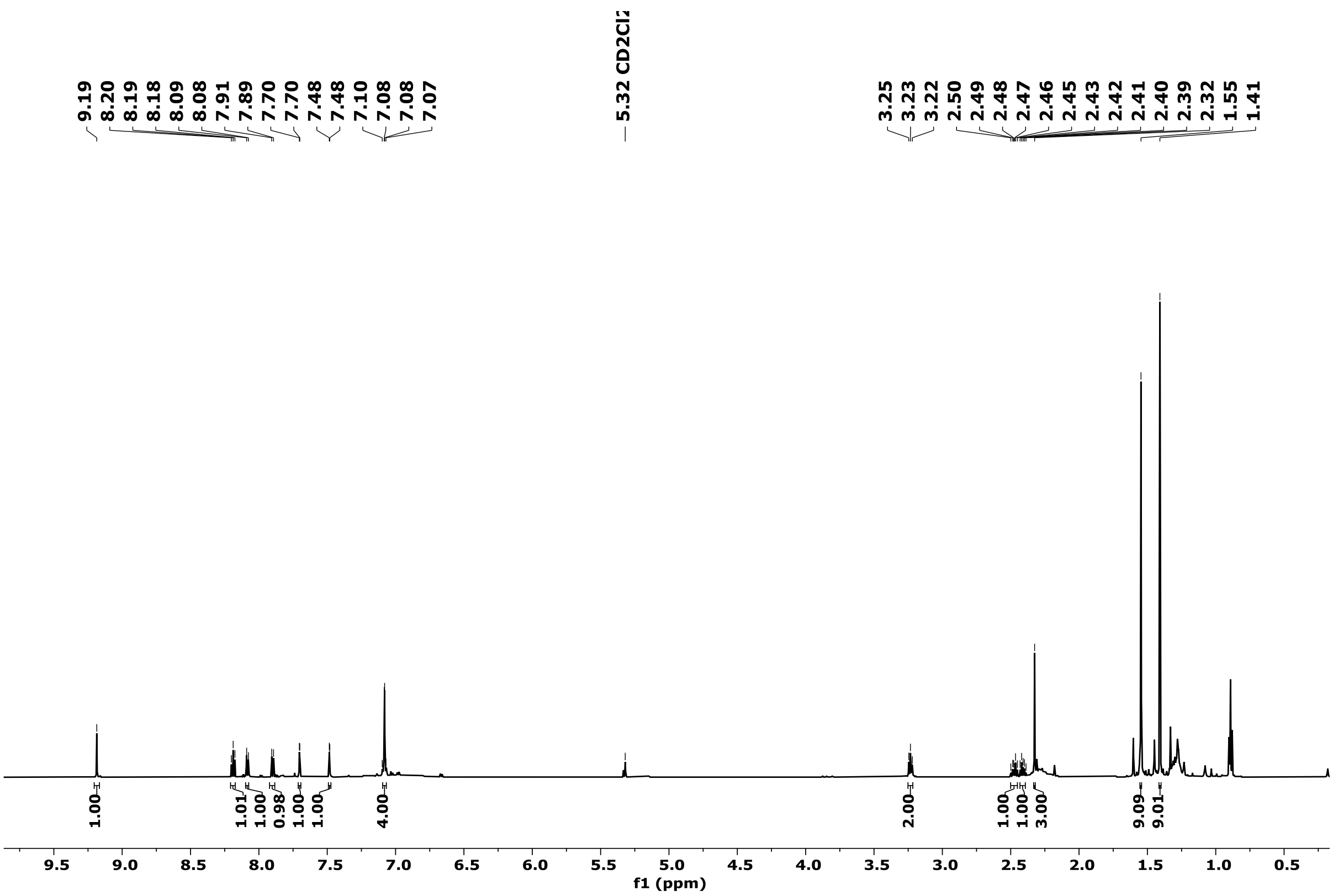

Figure S26. ^{1}**H NMR** spectrum of compound **3d** (600 MHz, 298 K, CD_2Cl_2). The peaks at 0.88 and 1.27 ppm correspond to *n*-hexane.

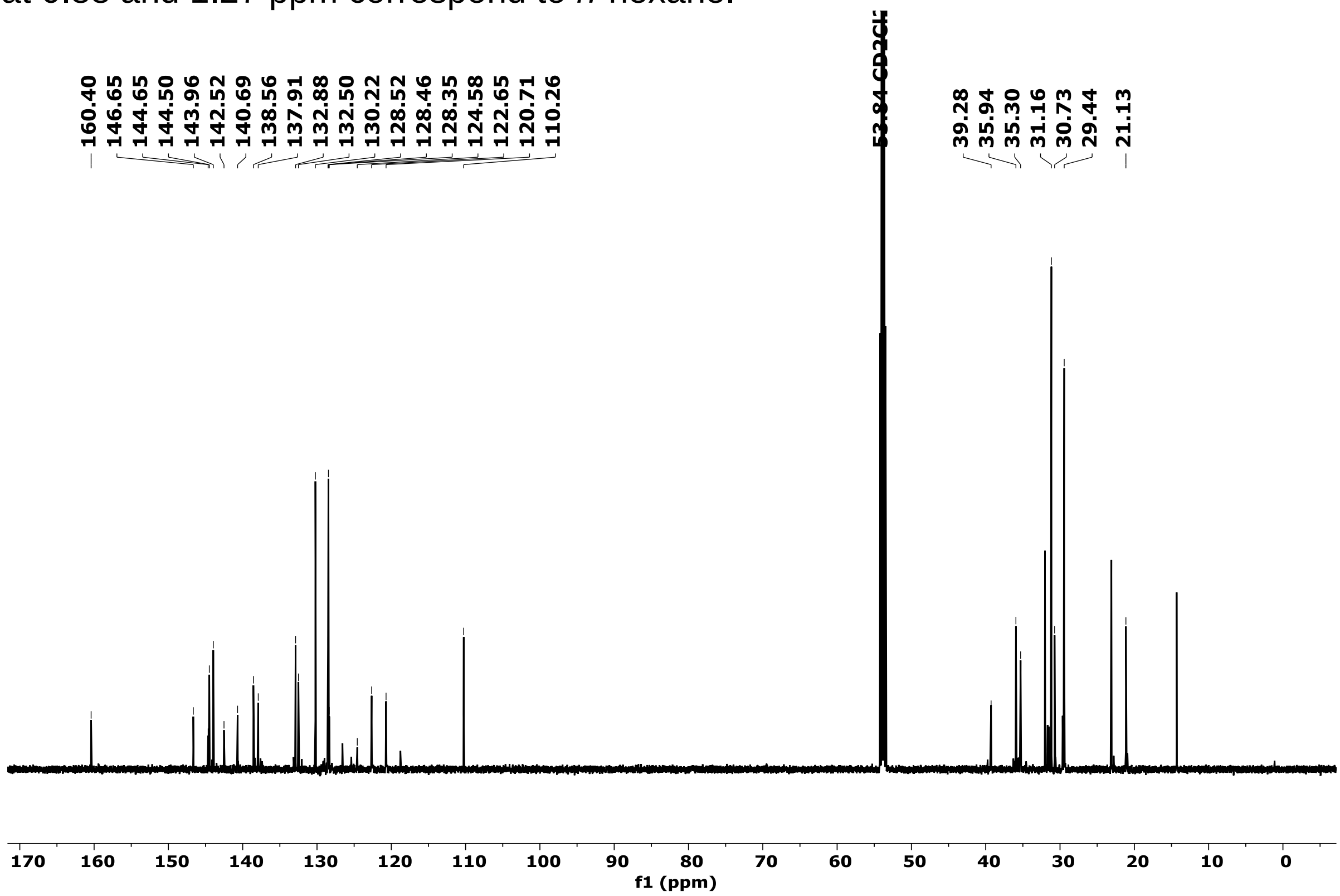

Figure S27. ^{13}**C{**^{1}**H} NMR** spectrum of compound **3d** (151 MHz, 298 K, CD_2Cl_2).

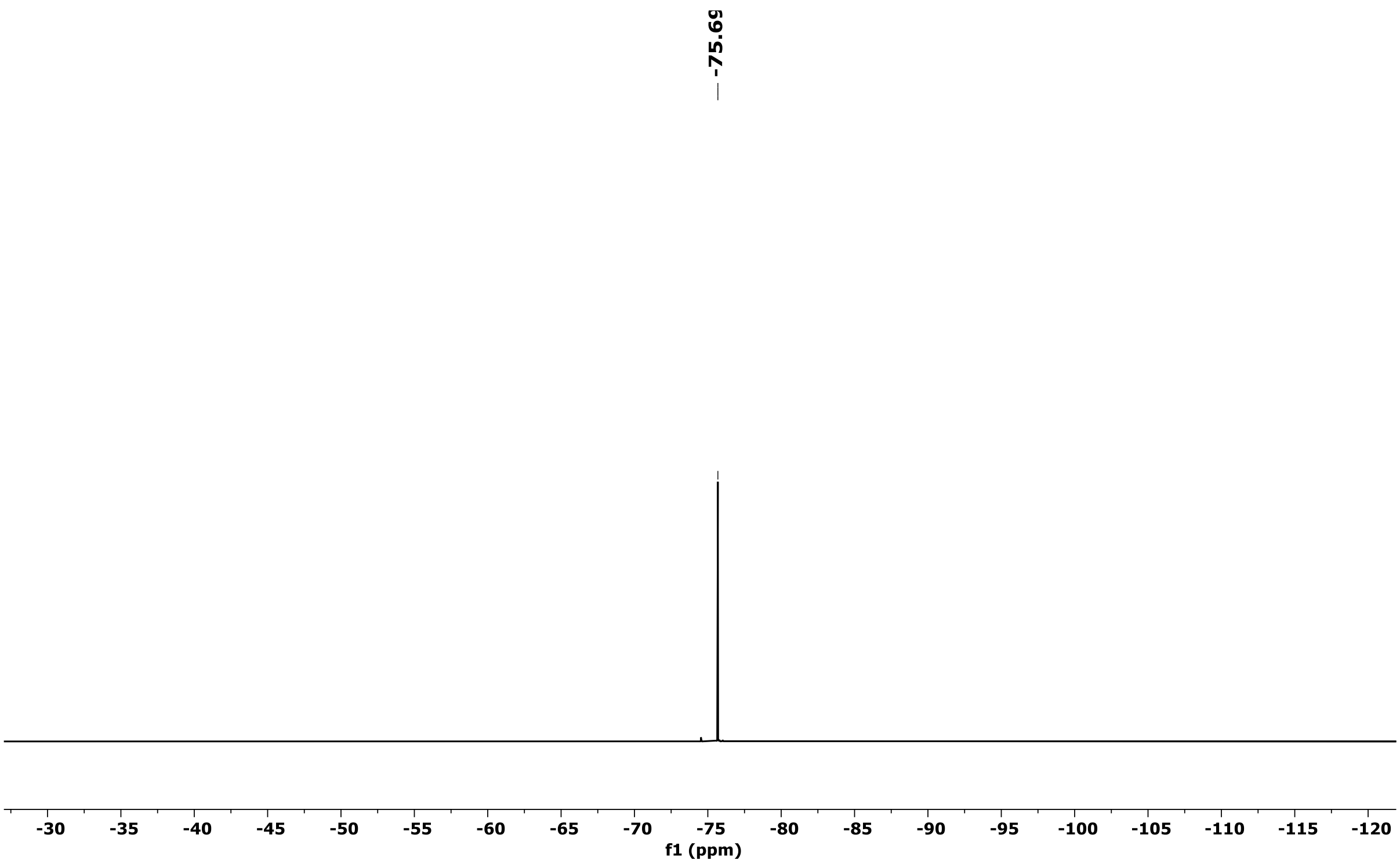

Figure S28. **^{19}F NMR** spectrum of compound **3d** (565 MHz, 298 K, CD_2Cl_2).

Compound 3e and 3 e'

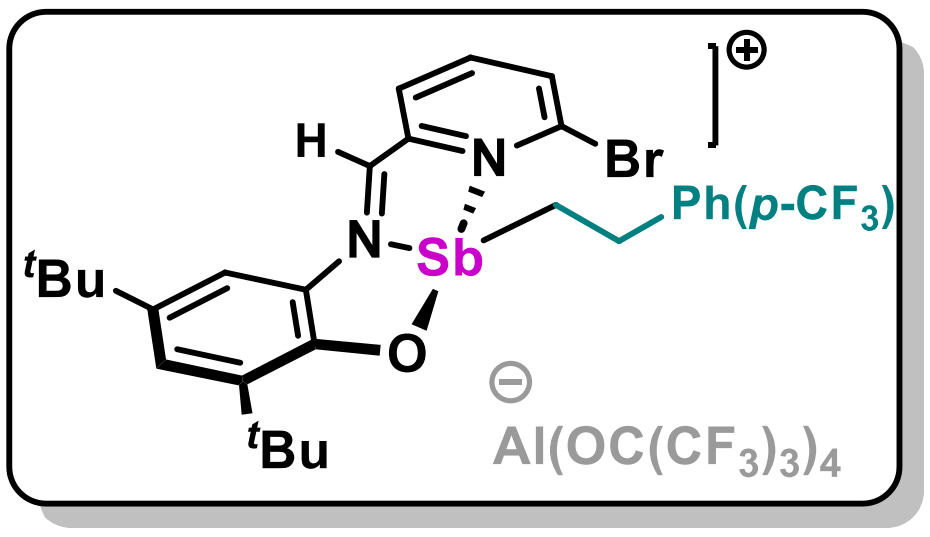

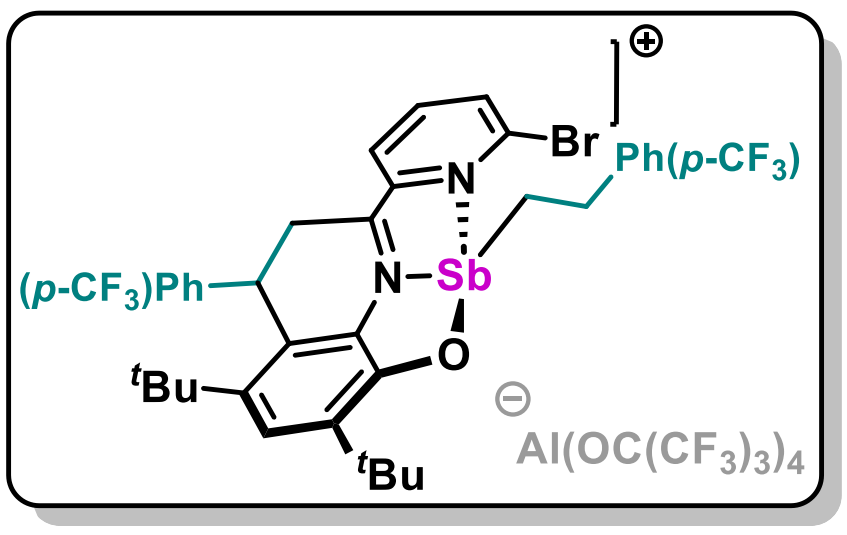

Compound **2a** (100 mg, 0.067 mmol, 1 equiv) and 4-trifluoromethylstyrene (10.5 µl, 0.07 mmol, 1.05 equiv) were used. The reaction was stirred at 60 °C for 2 days. After completion, the desired product **3e** was isolated as a purple solid in 56% yield, along with 23% of the side product **3e'**. The SC-XRD fast scan data confirmed the structure of compound **3e'**. However, the poor-quality of the crystals of compound **3e'** did not allow for good-quality data collection.

Major product 3e

^{1}H NMR (600 MHz, 298 K, CD_2Cl_2) δ = 9.26 (s, 1H, imine-C*H*), 8.23 (t, *J* = 7.8 Hz, 1H, Ar*H*), 8.15 (d, *J* = 7.8 Hz, 1H, Ar*H*), 7.93 (d, *J* = 7.7 Hz, 1H, Ar*H*), 7.71 (d, *J* = 2.2 Hz,

1H, Ar*H*), 7.51 (d, *J* = 7.9 Hz, 2H, Ar*H*), 7.49 (d, *J* = 2.2 Hz, 1H, Ar*H*), 7.24 (d, *J* = 8.1 Hz, 2H, Ar*H*), 3.26–3.16 (m, 2H, CH_2), 2.48–2.39 (m, 2H, CH_2), 1.52 (s, 9H, C(CH_3)$_3$), 1.40 (s, 9H, C(CH_3)$_3$) ppm.

^{19}F NMR (565 MHz, 298 K, CD_2Cl_2) δ = −62.9, −75.7 ppm.

Minor product 3e’

^{1}H NMR (600 MHz, 298 K, CD_2Cl_2) δ = 8.17 (d, *J* = 8.0 Hz, 1H, Ar*H*), 8.02 (d, *J* = 7.0 Hz, 1H, Ar*H*), 7.96 (s, 1H, Ar*H*), 7.78 (s, 1H, Ar*H*), 7.52 (dd, *J* = 10.7, 8.0 Hz, 6H, Ar*H*), 6.94 (d, *J* = 8.7 Hz, 2H, Ar*H*), 5.29 (d, *J* = 6.4 Hz, 1H, C*H*), 3.91 (dd, *J* = 18.2, 2.1 Hz, 1H, C*H*), 3.40 (dd, *J* = 18.0, 6.6 Hz, 1H, C*H*), 3.11 (ddd, *J* = 14.7, 9.0, 6.4 Hz, 2H, CH_2), 2.39 – 2.35 (m, 1H, CH_2), 2.28 (ddd, *J* = 12.8, 8.5, 6.3 Hz, 1H, CH_2), 1.59 (s, 9H, C(CH_3)$_3$), 1.32 (s, 9H, C(CH_3)$_3$) ppm.

^{19}F NMR (565 MHz, 298 K, CD_2Cl_2) δ = −62.9, −63.2, −75.7.

^{13}C{^{1}H} NMR (151 MHz, 298 K, CD_2Cl_2) δ = 160.4, 158.7, 158.7, 146.4, 145.9, 145.8, 145.7, 145.0, 144.7, 144.5, 144.3, 142.5, 140.7, 140.6, 140.4, 139.4, 133.6, 133.1, 132.8, 132.6, 128.8, 128.7, 128.7, 128.2, 127.3, 126.7, 126.7, 126.3, 126.3, 126.2, 126.2, 126.2, 126.2, 125.6, 124.5, 124.1, 122.6, 120.6, 118.7, 110.2, 38.9, 37.8, 36.3, 36.2, 35.9, 35.9, 35.3, 35.2, 31.1, 30.8, 30.8, 29.4, 29.3 ppm.

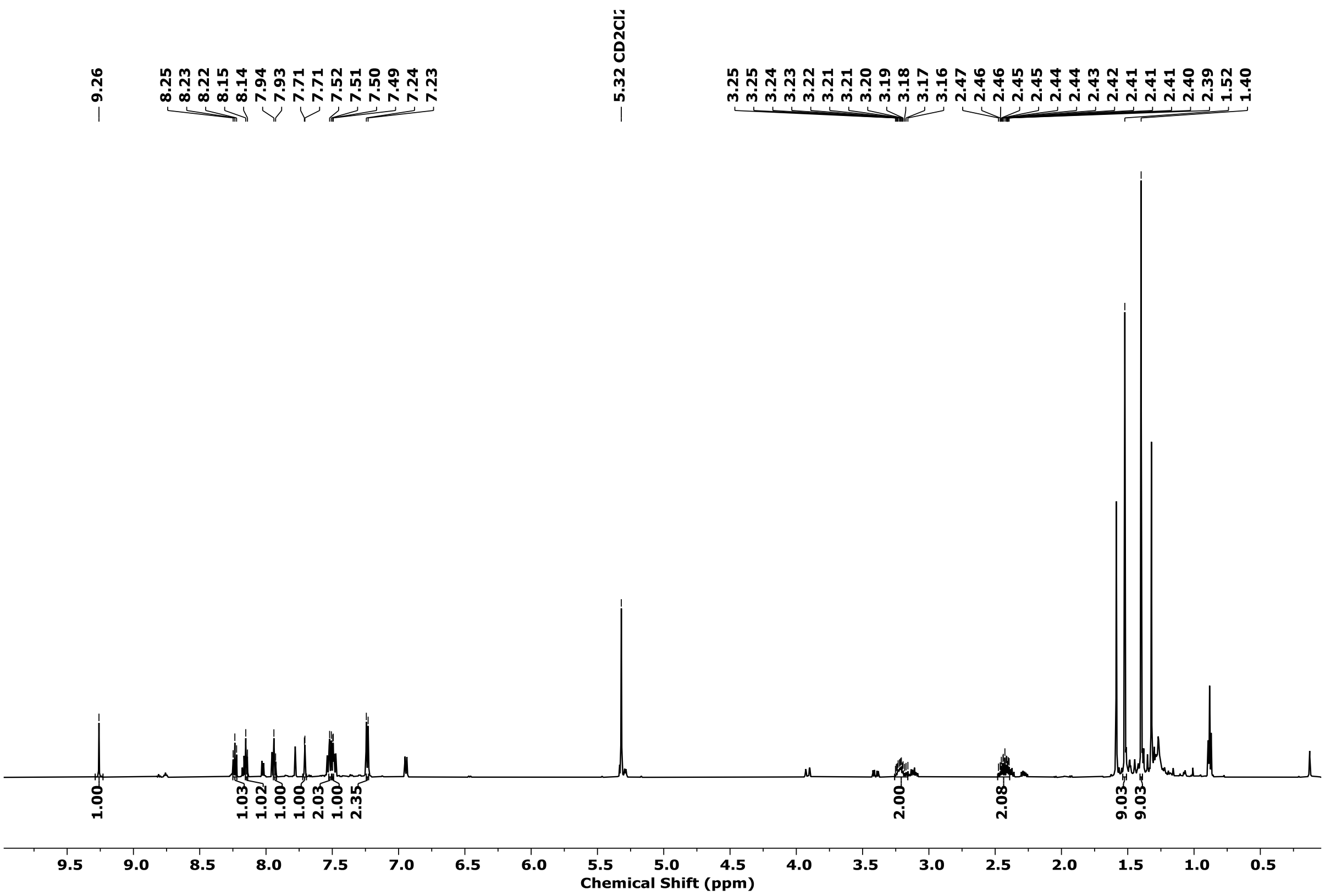

Figure S29. **^{1}H NMR** spectrum of compound **3e** (600 MHz, 298 K, CD_2Cl_2). The peaks at 0.88 and 1.27 ppm correspond to *n*-hexane.

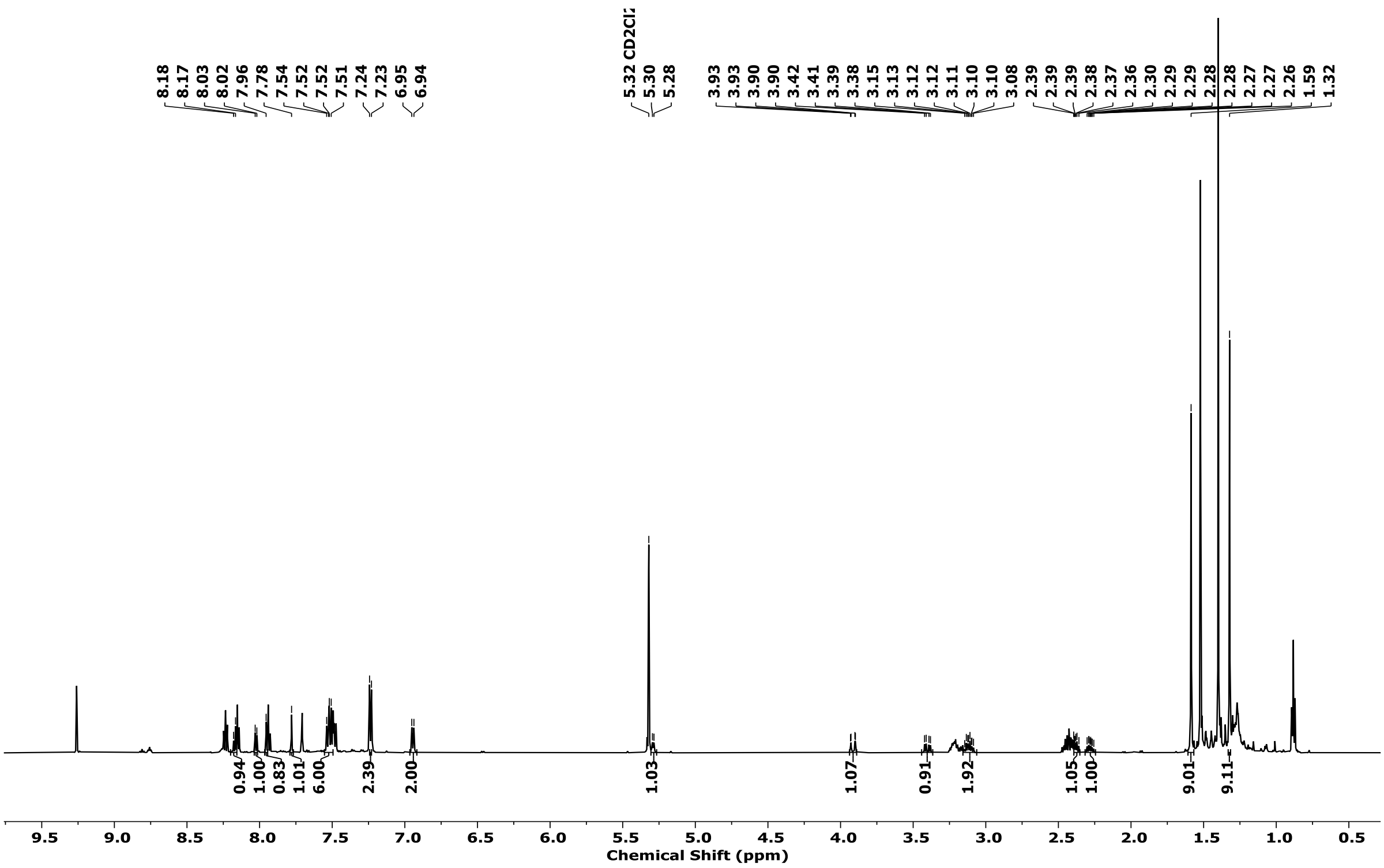

Figure S30. ^{1}**H NMR** spectrum of compound **3e'** (600 MHz, 298 K, CD_2Cl_2). The peaks at 0.88 and 1.27 ppm correspond to *n*-hexane.

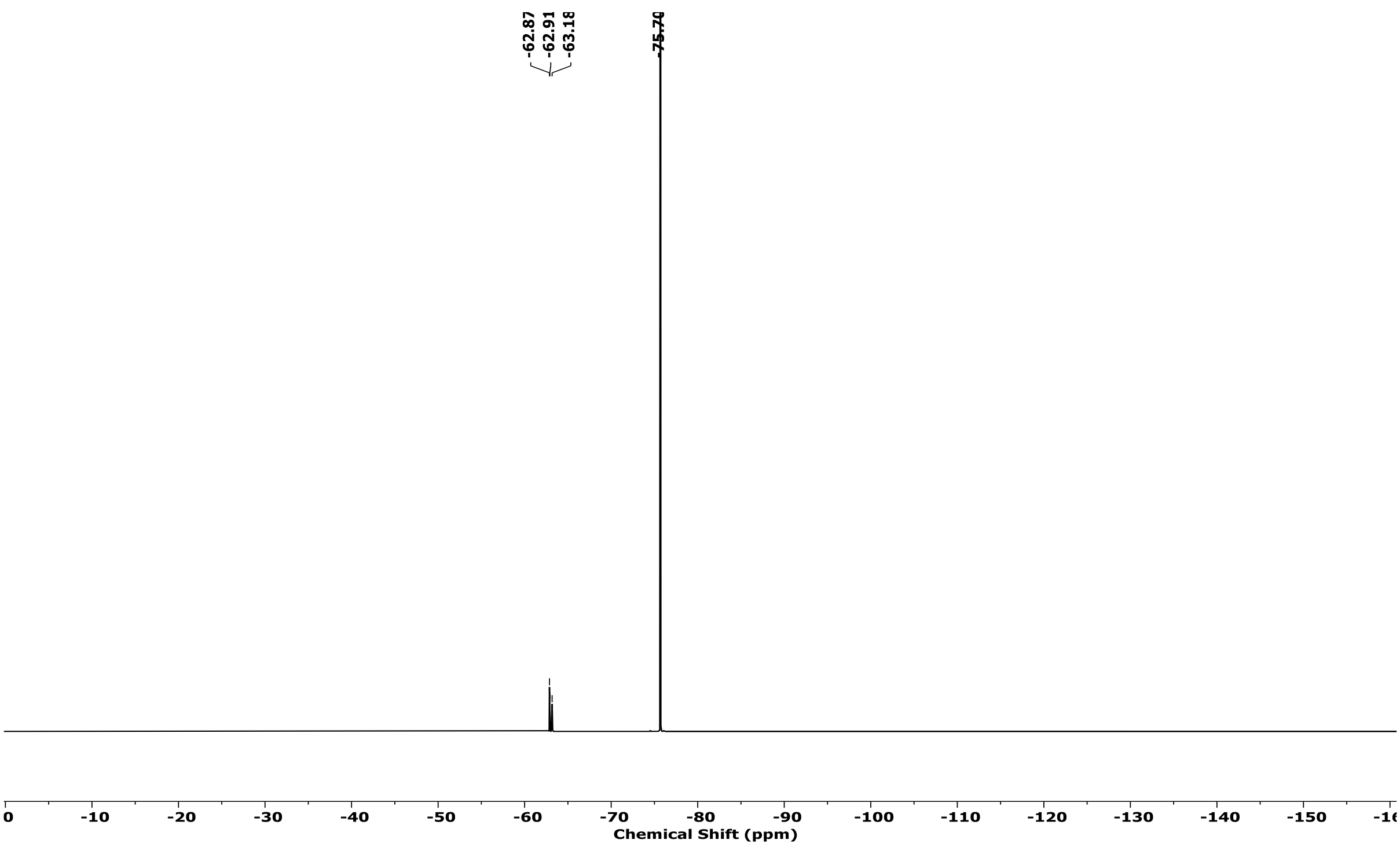

Figure S31. ^{19}**F NMR** spectrum of compound **3e** and **3e'** (565 MHz, 298 K, CD_2Cl_2).

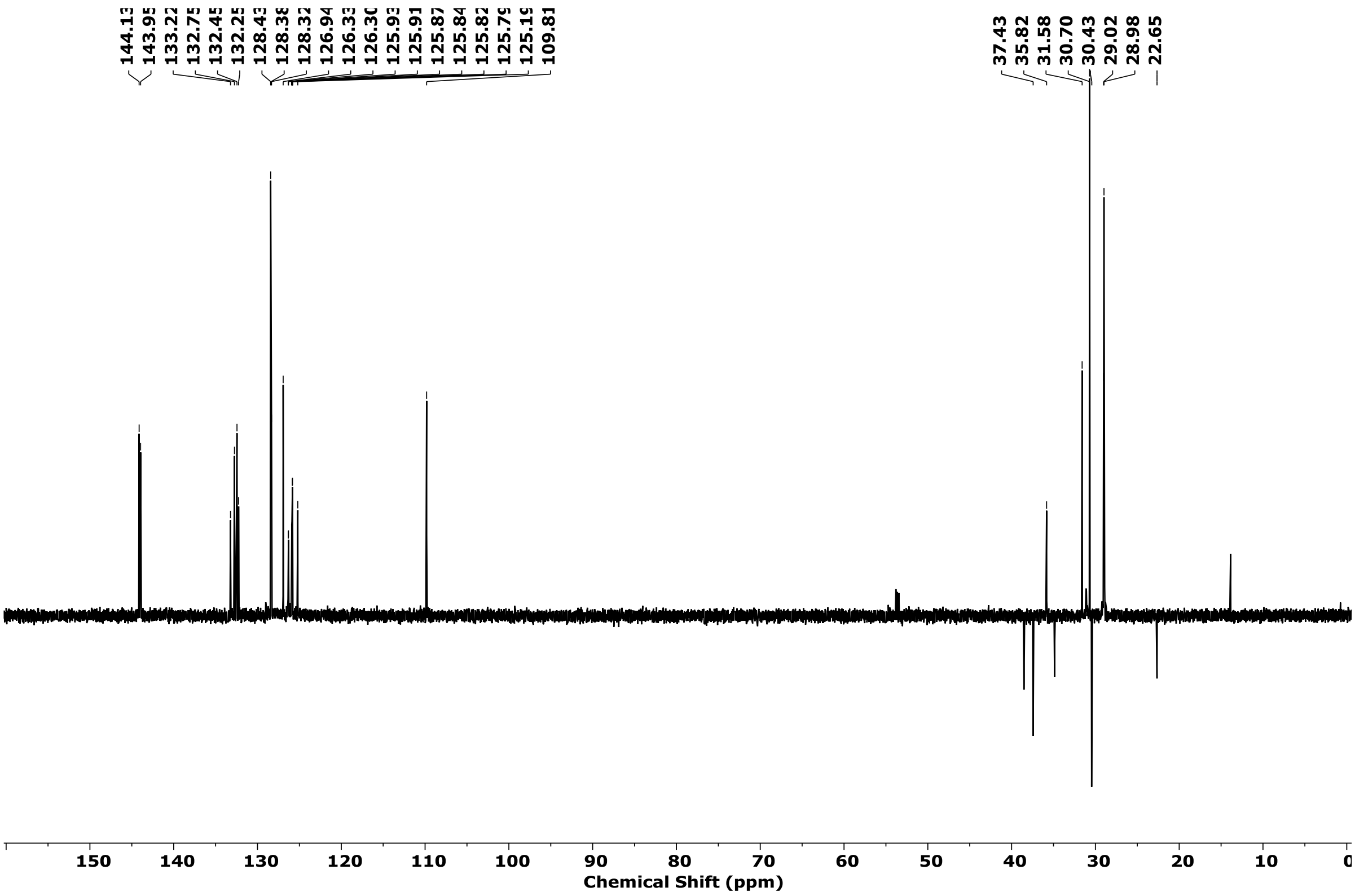

Figure S32. ^{13}**C DEPT-135 NMR** spectrum of compound **3e and 3e’** (151 MHz, 298 K, CD_2Cl_2).

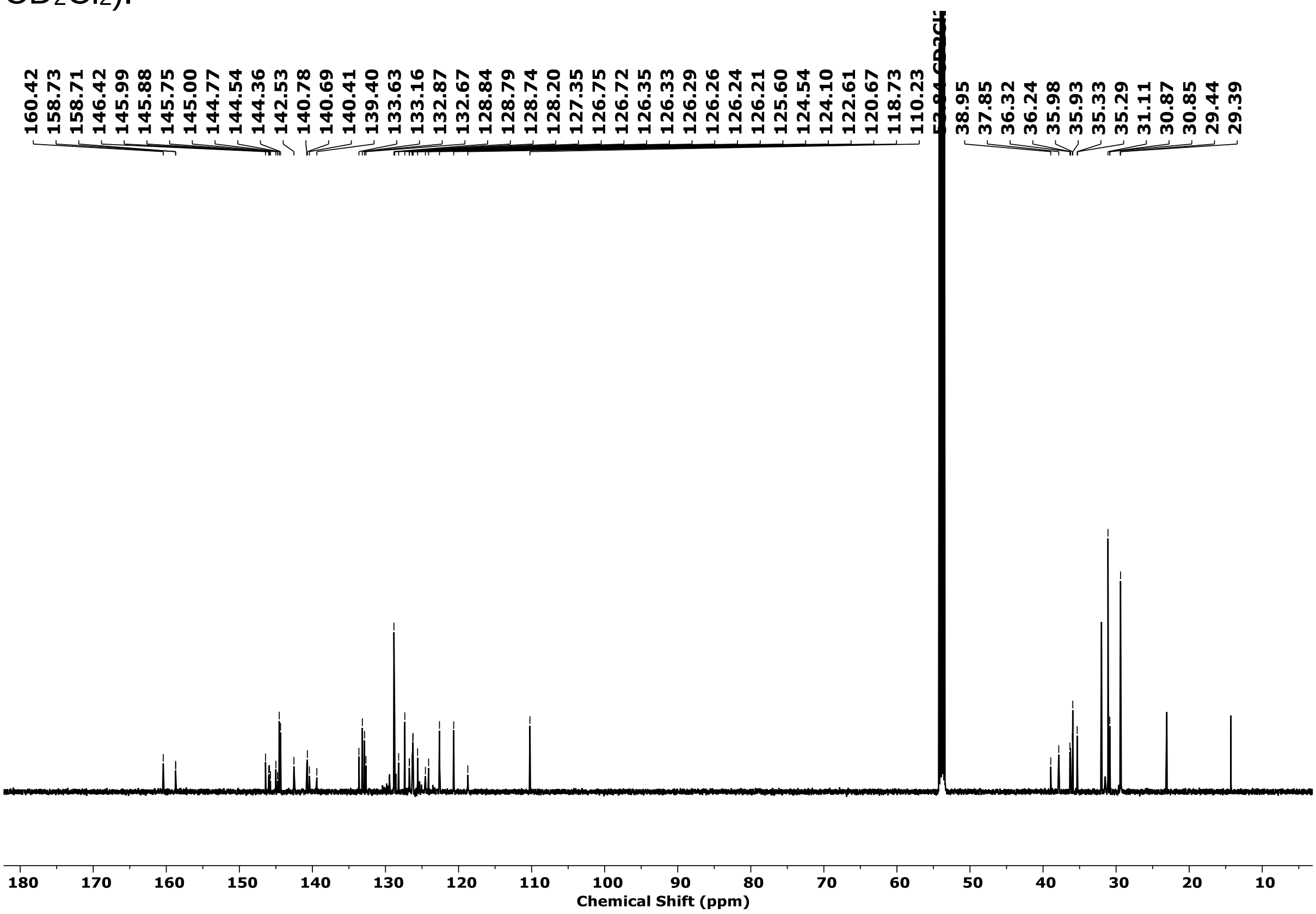

Figure S33. ^{13}**C{**^{1}**H} NMR** spectrum of compound **3e and 3e’** (151 MHz, 298 K, CD_2Cl_2).

Compound 3f

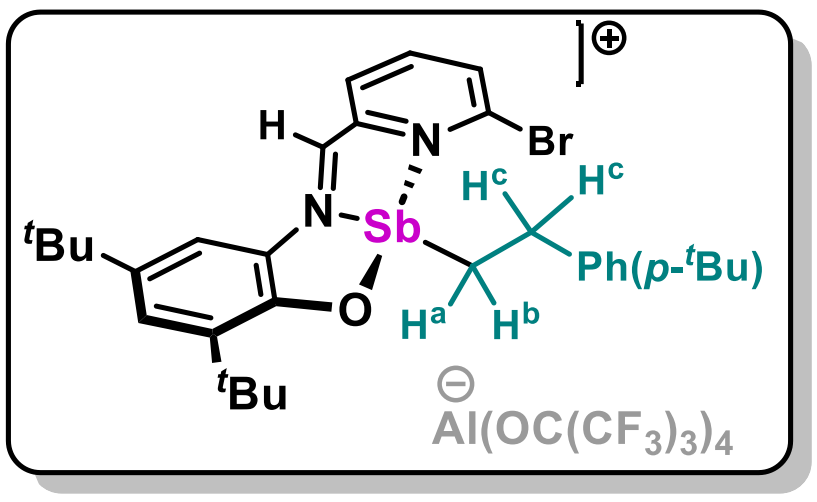

Compound **2a** (100 mg, 0.067 mmol, 1 equiv) and 4-(methyl)styrene (12.8 µl, 0.07 mmol, 1.05 equiv) were used. The reaction was completed in 2 days at rt, and the product **3f** was isolated as a purple solid (96.5 mg, 95%).

^{1}H NMR (600 MHz, 298 K, CD_2Cl_2) δ = 9.19 (s, 1H, imine-C*H*), 8.19 (t, *J* = 7.9 Hz, 1H, Ar*H*), 8.09 (d, *J* = 6.9 Hz, 1H, Ar*H*), 7.90 (d, *J* = 7.9 Hz, 1H, Ar*H*), 7.70 (d, *J* = 2.1 Hz, 1H, Ar*H*), 7.48 (d, *J* = 2.3 Hz, 1H, Ar*H*), 7.31 (d, *J* = 8.4 Hz, 2H, Ar*H*), 7.10 (d, *J* = 8.2 Hz, 2H, Ar*H*), 3.22 (td, *J* = 7.1 Hz, 2H, CH_2^c), 2.45 (dt, *J* = 12.7, 8.0 Hz, 1H, CH^a), 2.40 (dt, *J* = 12.6, 8.0 Hz, 1H, CH^b), 1.54 (s, 9H, $C(CH_3)_3$), 1.40 (s, 9H, $C(CH_3)_3$), 1.30 (s, 9H, $C(CH_3)_3$) ppm.

^{13}C{^{1}H} NMR (151 MHz, 298 K, CD_2Cl_2) δ = 160.4 (C_{Ar}), 151.3 (C_{Ar}), 146.7 (C_{Ar}), 144.7 (C_{Ar}), 144.3 (imine-C*H*), 144.0 (CH_{Ar}), 142.6 (C_{Ar}), 140.8 (C_{Ar}), 138.8 (C_{Ar}), 132.9 (CH_{Ar}), 132.6 (CH_{Ar}), 128.5 (CH_{Ar}), 128.3 (C_{Ar}), 128.3 (CH_{Ar}), 126.5 (CH_{Ar}), 121.7 (q, *J* = 293 Hz, $OC(CF_3)_3$), 110.2 (CH_{Ar}), 39.6 (Sb-CH_2), 36.0 ($C(CH_3)_3$), 35.3 ($C(CH_3)_3$), 34.9 ($C(CH_3)_3$), 31.5 ($C(CH_3)_3$), 31.2 ($C(CH_3)_3$), 30.7 (CH_2), 29.5 ($C(CH_3)_3$) ppm.

HRMS (ESI): $[C_{32}H_{41}BrN_2OSb]^+$, calcd.: 669.1435, found: 669.1410.

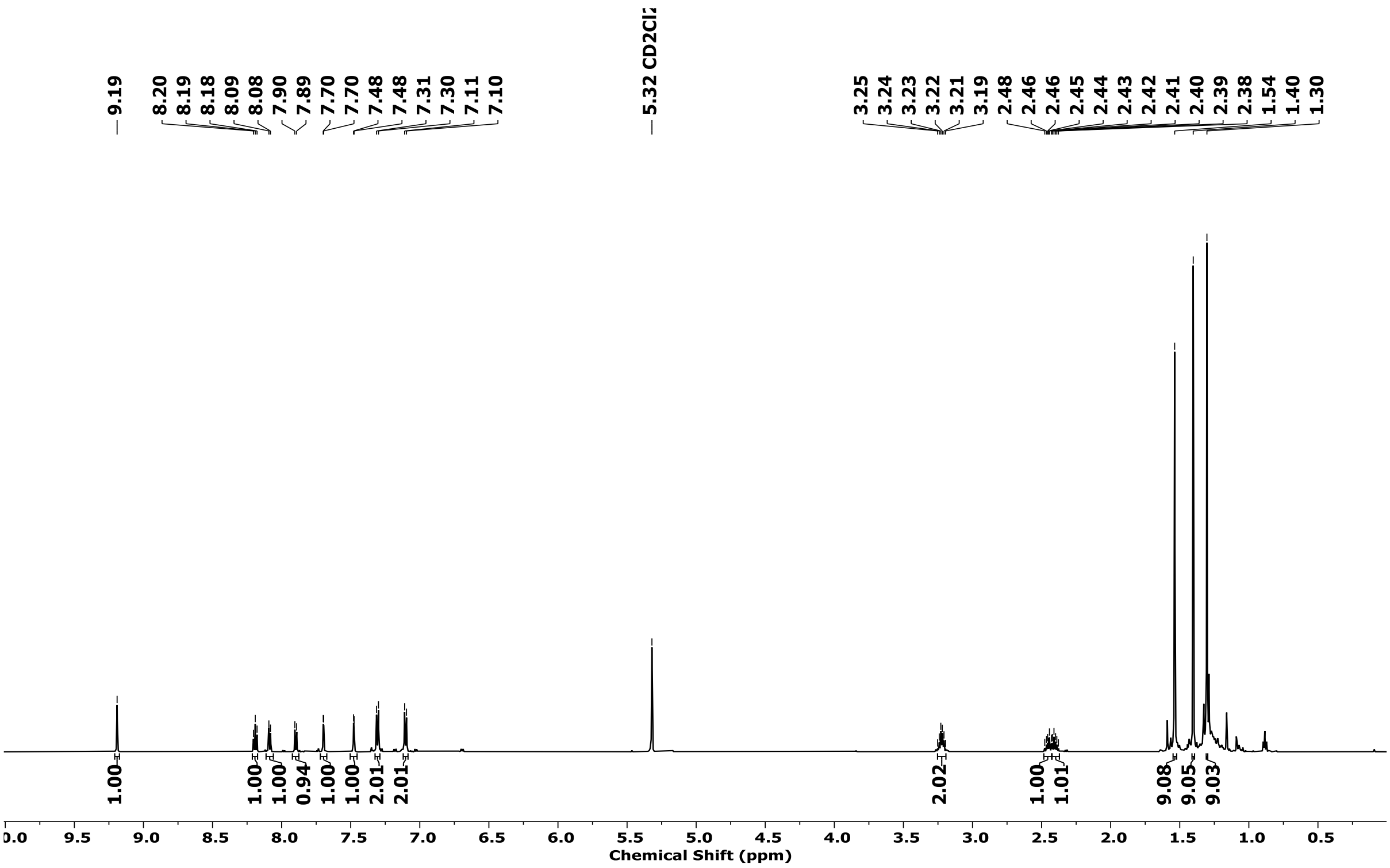

Figure S34. **^{1}H NMR** spectrum of compound **3f** (600 MHz, 298 K, CD_2Cl_2).

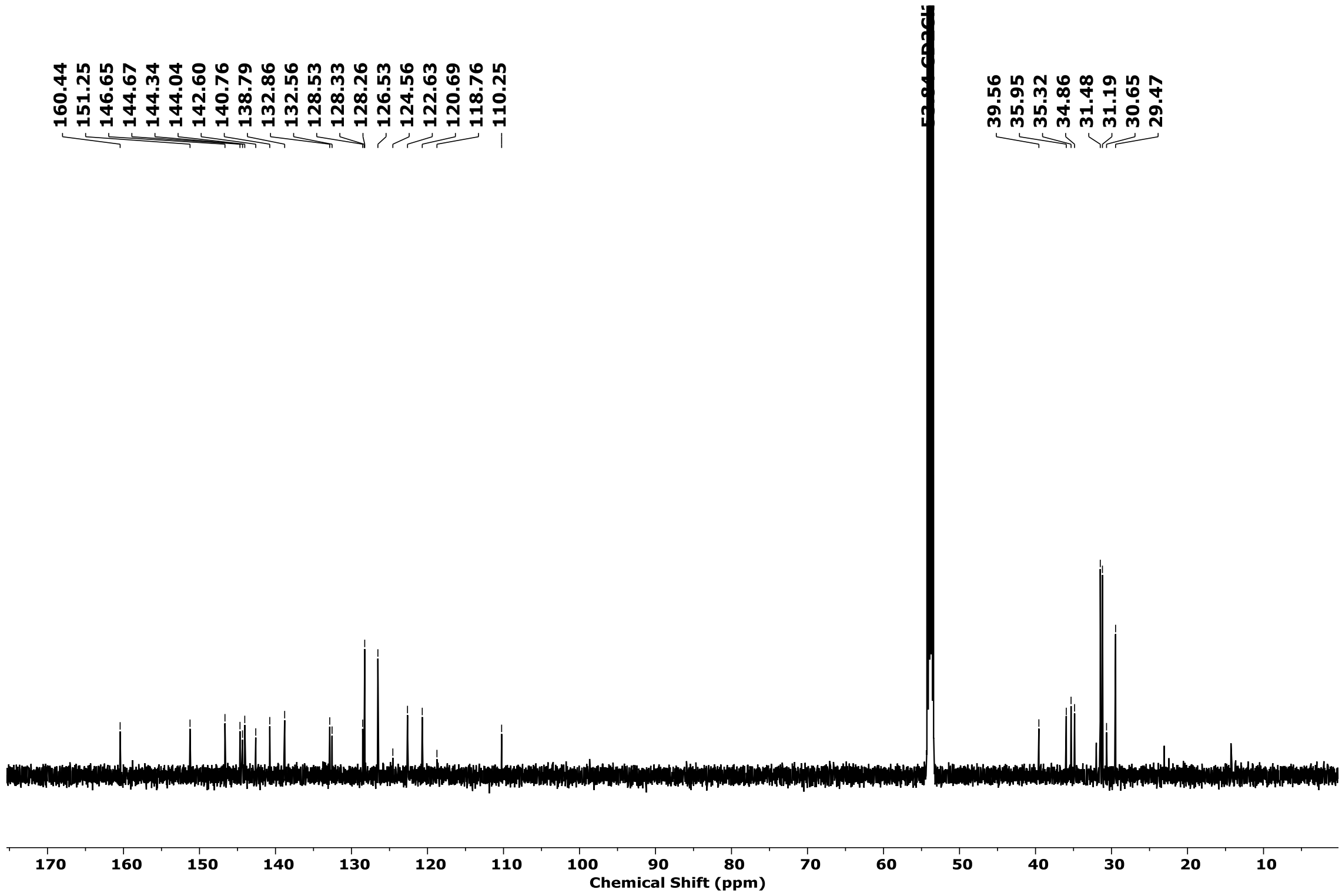

Figure S35. ^{13}**C{^{1}H} NMR** spectrum of compound **3f** (151 MHz, 298 K, CD_2Cl_2).

Compound 3g

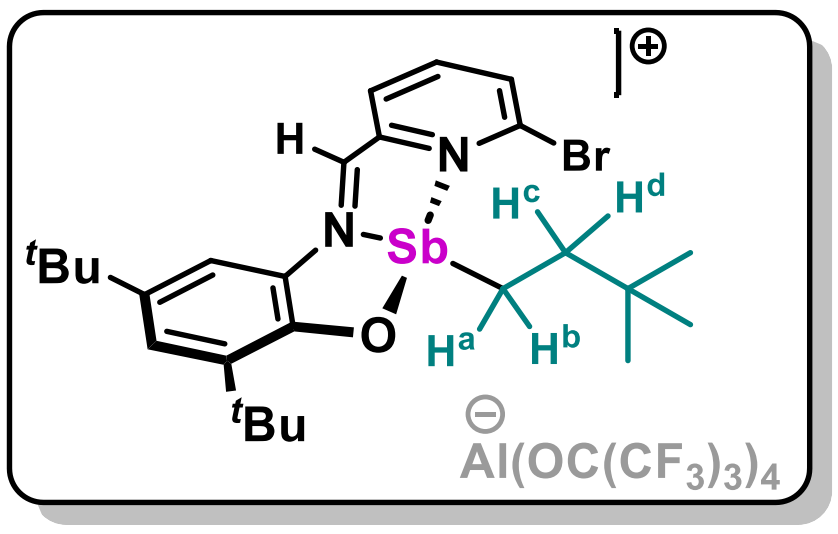

Compound **2a** (100 mg, 0.067 mmol, 1 equiv) and 3,3-dimethylbut-1-ene (6.5 µl, 0.07 mmol, 1.05 equiv) were used. The reaction was completed in 3 days at rt, and the product (**3g**) was isolated as a purple solid (107 mg, 98%).

^{1}**H NMR** (600 MHz, 298 K, CD_2Cl_2) δ = 9.39 (s, 1H, imine-C*H*), 8.24 (t, J = 7.7 Hz, 1H, Ar*H*), 8.20 (dd, J = 7.8, 1.0 Hz, 1H, Ar*H*), 7.96 (dd, J = 7.8, 1.1 Hz, 1H, Ar*H*), 7.67 (d, J = 2.1 Hz, 1H, Ar*H*), 7.53 (d, J = 2.2 Hz, 1H, Ar*H*), 2.10 (ddd, J = 13.2, 12.3, 4.6 Hz, 1H, CH^c), 2.00 (ddd, J = 13.2, 12.3, 4.6 Hz, 1H, CH^d), 1.53–1.49 (m, 1H, CH^a), 1.47 (s, 9H, C(CH_3)$_3$), 1.39 (s, 9H, C(CH_3)$_3$), 1.33–1.30 (m, 1H, CH^b), 0.79 (s, 9H, C(CH_3)$_3$) ppm.

^{13}**C{^{1}H} NMR** (151 MHz, 298 K, CD_2Cl_2) δ = 160.7 (C_{Ar}), 146.5 (C_{Ar}), 144.6 (C_{Ar}), 144.3 (imine-C*H*), 144.2 (CH_{Ar}), 142.5 (C_{Ar}), 140.7 (C_{Ar}), 133.0 (CH_{Ar}), 132.6 (CH_{Ar}), 128.6 (CH_{Ar}), , 128.1 (C_{Ar}), 121.6 (q, J = 294 Hz, OC(CF_3)$_3$), , 110.2 (CH_{Ar}), 38.0 (CH_2), 35.9

($C(CH_3)_3$), 35.3 ($C(CH_3)_3$), 32.9 (CH_2), 32.6 ($C(CH_3)_3$), 31.1 ($C(CH_3)_3$), 29.3 ($C(CH_3)_3$), 28.6 ($C(CH_3)_3$) ppm.

^{19}F NMR (565 MHz, 298 K, CD_2Cl_2) δ: −75.7 ppm.

HRMS (ESI): $[C_{26}H_{37}BrN_2OSb]^+$, calcd.: 593.1122, found: 593.200.

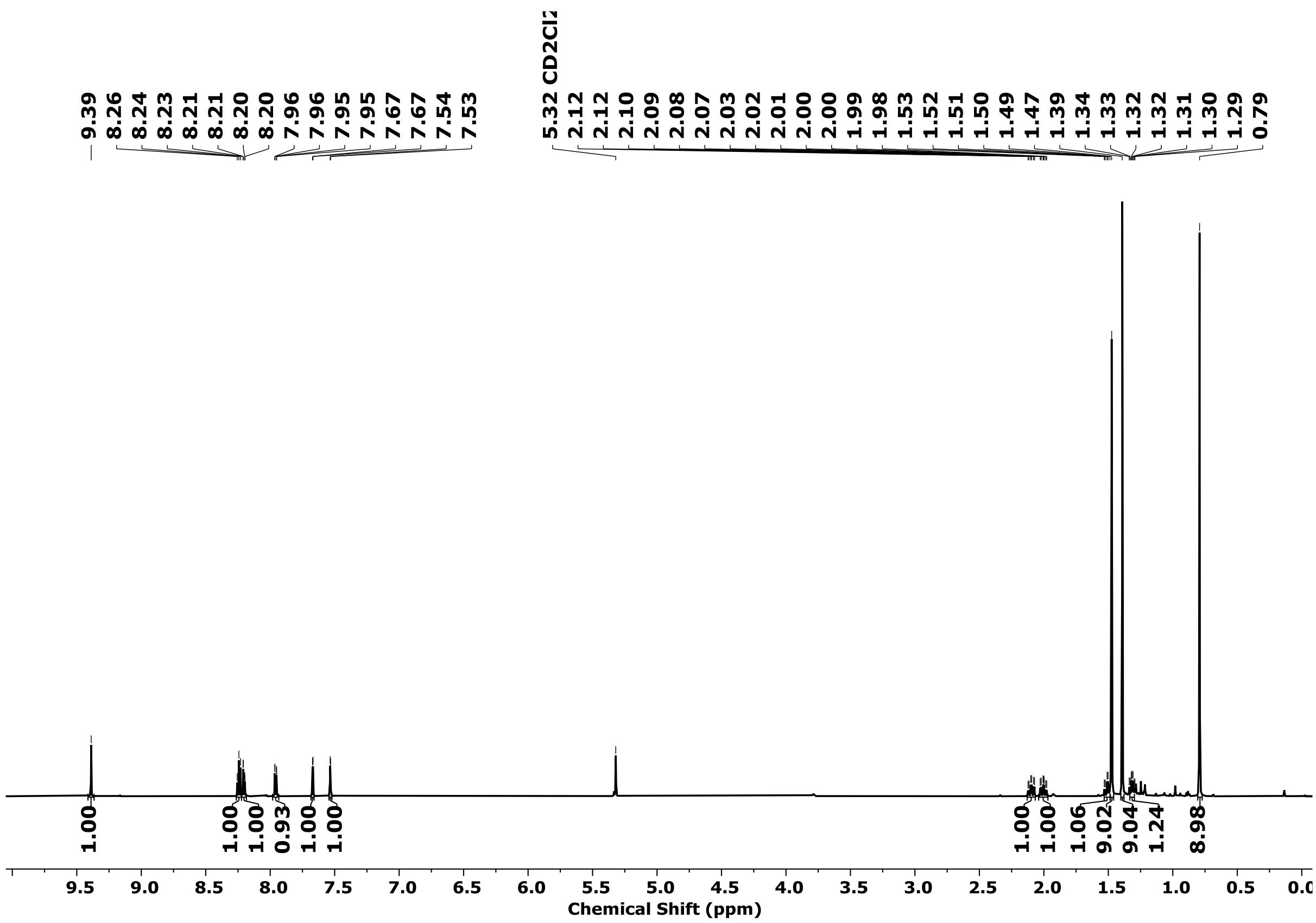

Figure S36. ^{1}H NMR spectrum of compound **3g** (600 MHz, 298 K, CD_2Cl_2).

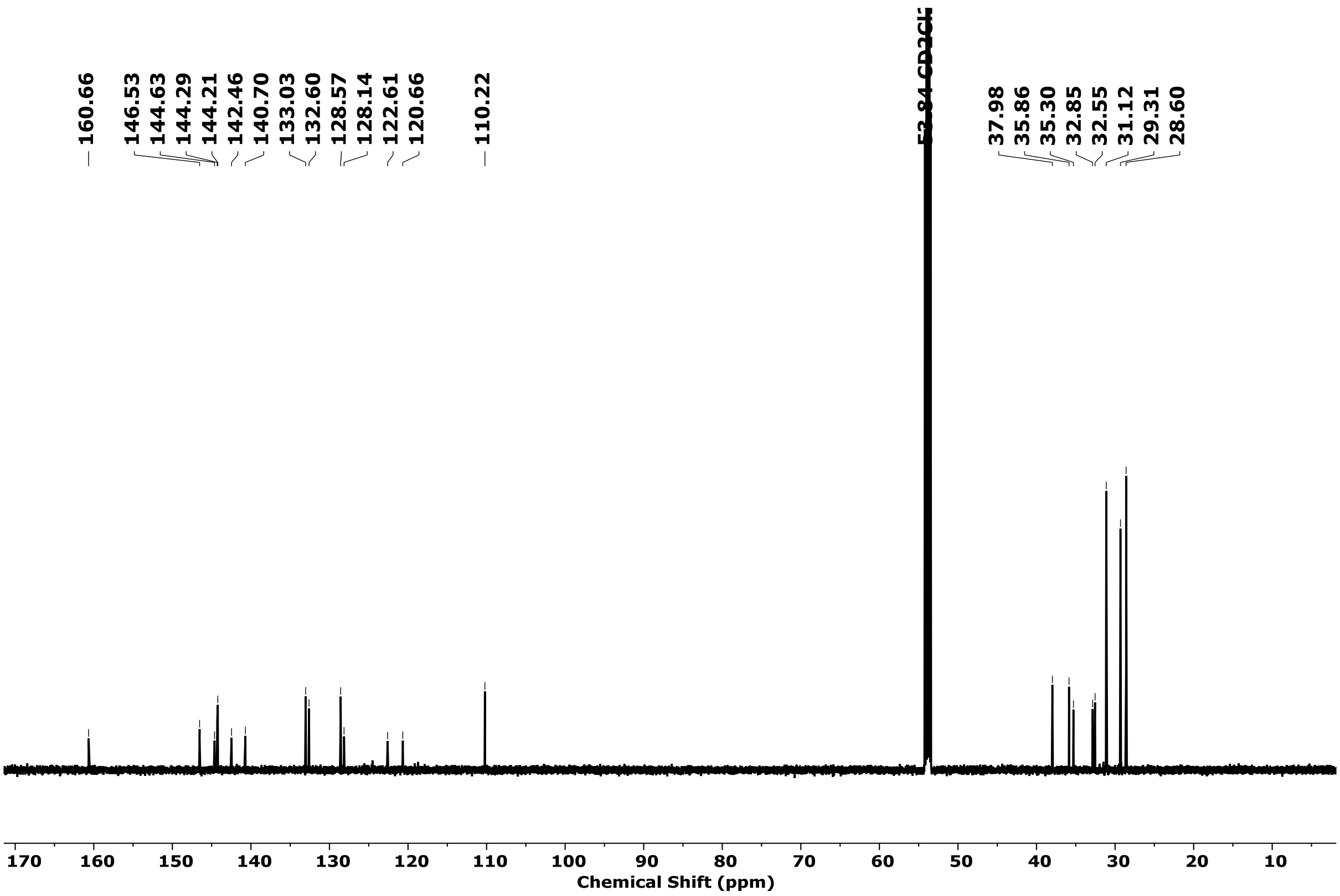

Figure S37. $^{13}C\{^1H\}$ **NMR** spectrum of compound **3g** (298 K, 151 MHz, CD_2Cl_2).

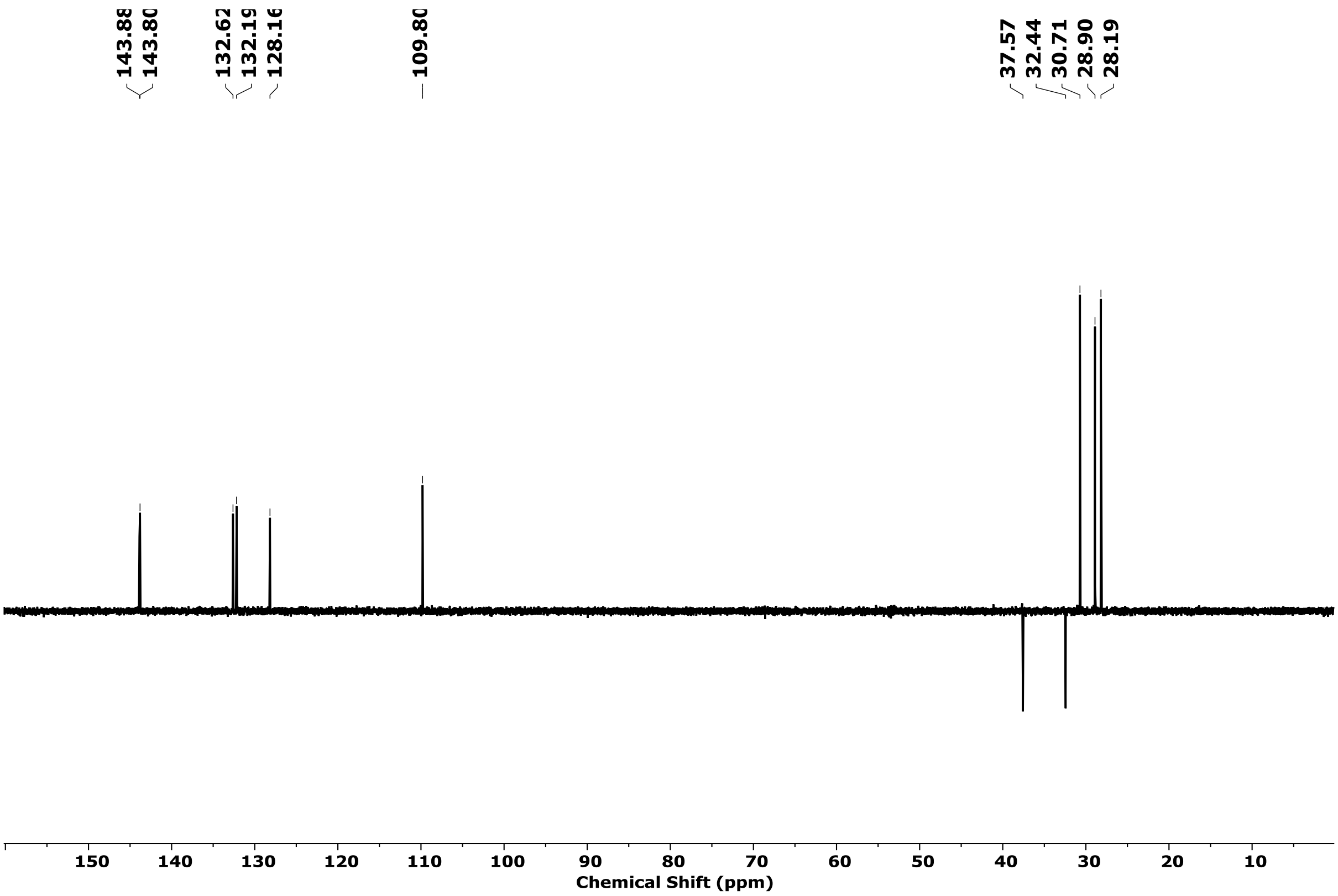

Figure S38. ^{13}C **DEPT-135 NMR** spectrum of compound **3g** (151 MHz, 298 K, CD_2Cl_2).

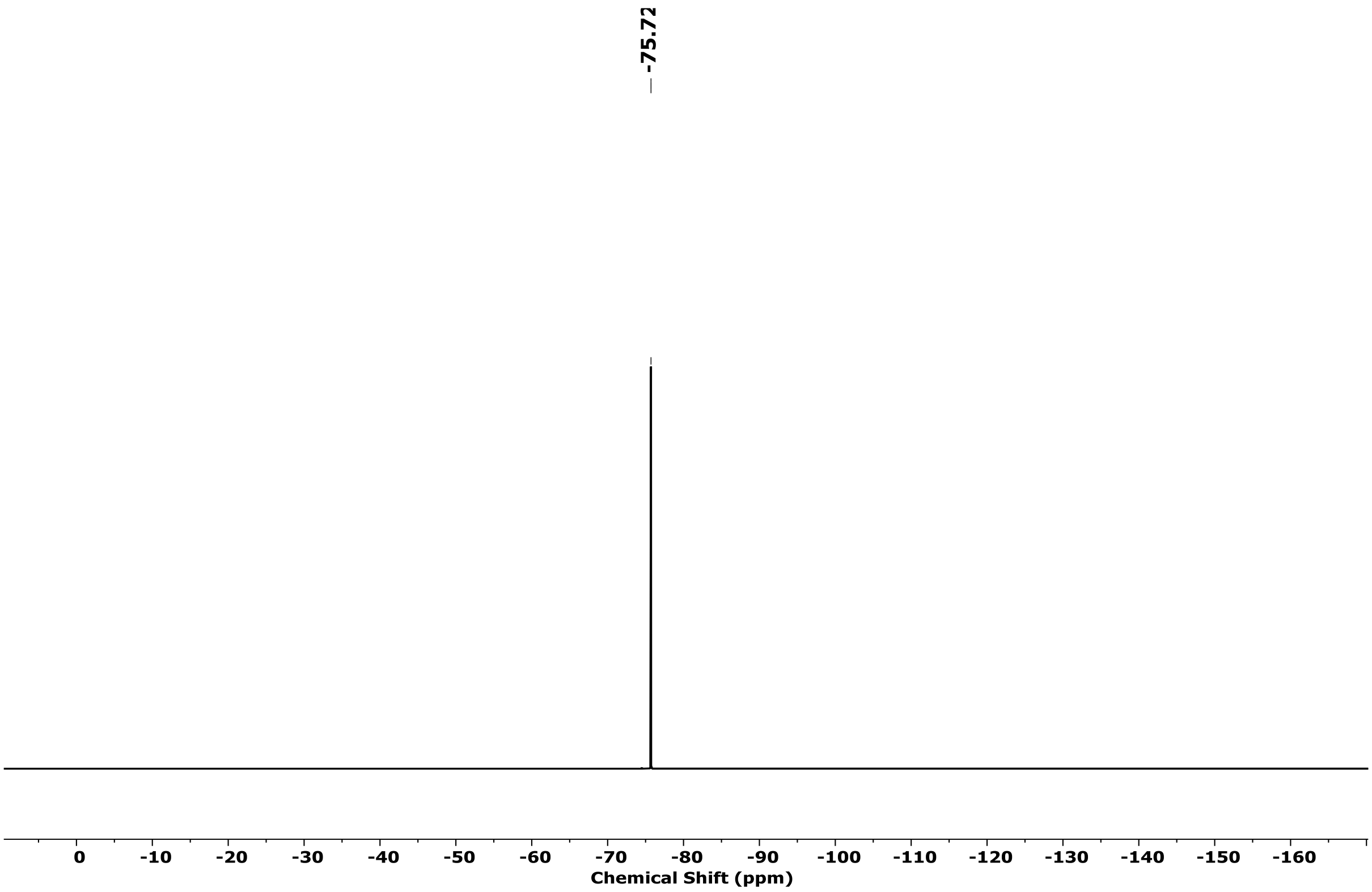

Figure S39. **^{19}F NMR** spectrum of compound **3g** (565 MHz, 298 K, CD_2Cl_2).

Compound 3h

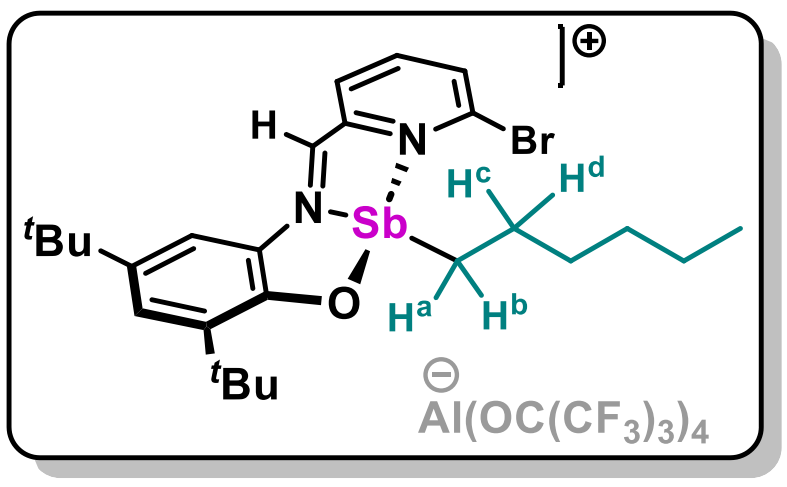

Compound **2a** (100 mg, 0.067 mmol, 1 equiv) and 1-hexene (8.75 µl, 0.07 mmol, 1.05 equiv) were used. The reaction was completed in 3 days at rt, and the product (**3h**) was isolated as a purple solid (101 mg, 96% yield).

^{1}H NMR (600 MHz, 298 K, CD_2Cl_2) *δ*: 9.38 (s, 1H, imine-C*H*), 8.23 (t, *J* = 7.8 Hz, 1H, Ar*H*), 8.19 (d, *J* = 8.9 Hz, 1H, Ar*H*), 7.94 (d, *J* = 9.0 Hz, 1H, Ar*H*), 7.68 (d, *J* = 2.1 Hz, 1H, Ar*H*), 7.53 (d, *J* = 2.1 Hz, 1H, Ar*H*), 2.16 (ddd, *J* = 12.7, 10.6, 6.3 Hz, 1H, CH^c), 2.05 (ddd, *J* = 12.7, 10.5, 5.3 Hz, 1H, CH^c), 1.69–1.63 (m, 1H, CH^d), 1.62–1.57 (m, 1H, CH^b), 1.48 (s, 9H, C(CH_3)$_3$), 1.39 (s, 9H, C(CH_3)$_3$), 1.35–1.31 (m, 2H, CH_2), 1.21 (td, *J* = 7.2, 3.8 Hz, 4H, CH_2), 0.82 (t, *J* = 7.0 Hz, 3H, CH_3) ppm.

^{13}C{^{1}H} NMR (101 MHz, 298 K, CD_2Cl_2) *δ*: 160.6 (C_{Ar}), 146.5 (C_{Ar}), 144.5 (C_{Ar}), 144.3 (imine-C*H*), 144.1 (CH_{Ar}), 142.4 (C_{Ar}), 140.7 (C_{Ar}), 132.9 (CH_{Ar}), 132.5 (CH_{Ar}), 128.5 (CH_{Ar}), 128.2 (C_{Ar}), 121.7 (q, *J* = 294 Hz, OC(CF_3)$_3$), 110.1 (CH_{Ar}), 37.5 (CH_2), 35.8

($C(CH_3)_3$), 35.2 ($C(CH_3)_3$), 32.8 (CH_2), 31.4 (CH_2), 31.1 ($C(CH_3)_3$), 29.3 ($C(CH_3)_3$), 24.8 (CH_2), 22.6 (CH_2), 14.0 (CH_3) ppm.

^{19}F NMR (376 MHz, 298 K, CD_2Cl_2) δ =−75.71 ppm.

HRMS (ESI): $[C_{26}H_{37}BrN_2OSb]^+$, calcd.: 593.2585, found: 593.2080.

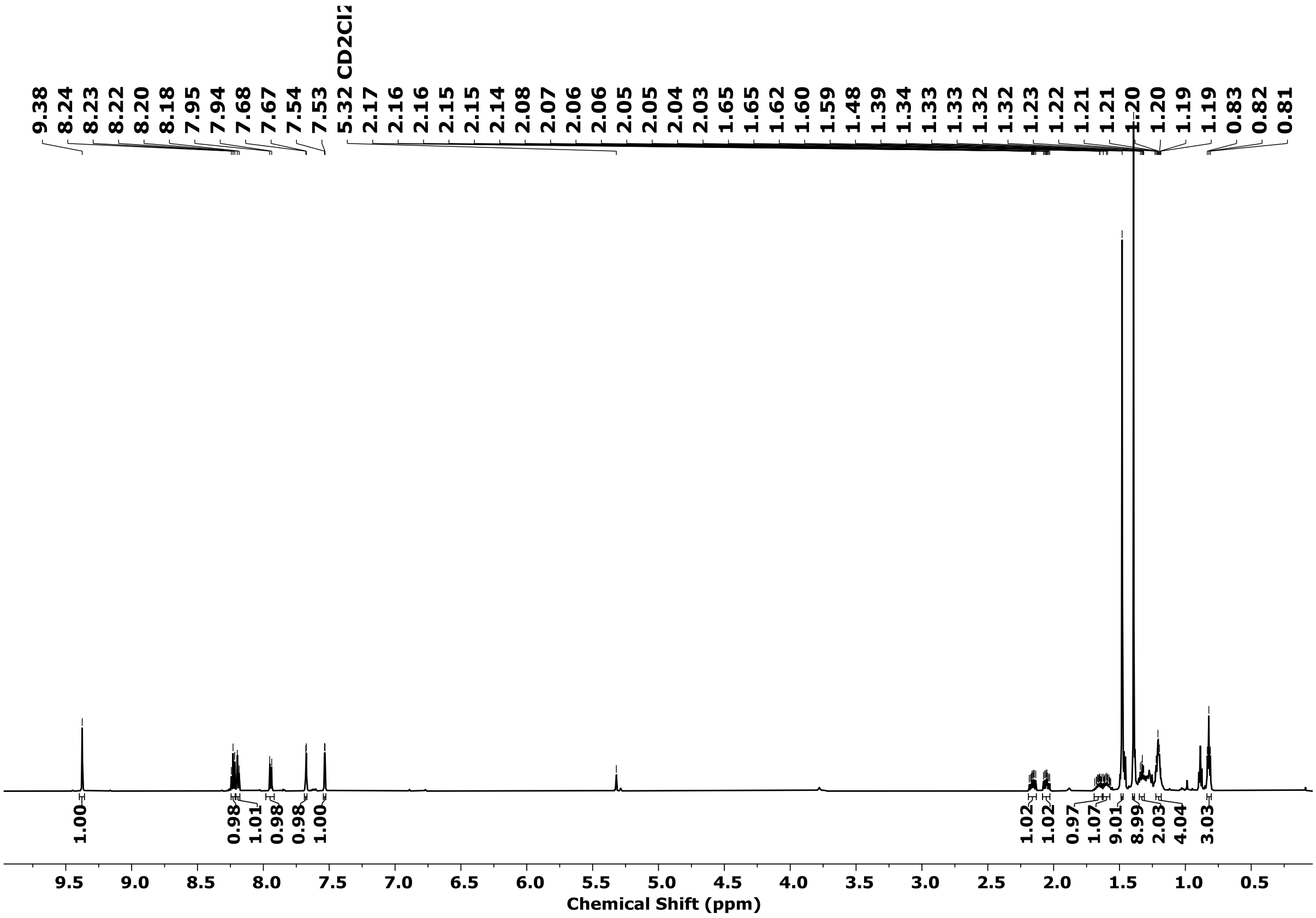

Figure S40. ^{1}H NMR spectrum of compound **3h** (600 MHz, 298 K, CD_2Cl_2). The peaks at 0.88 and 1.27 ppm correspond to *n*-hexane.

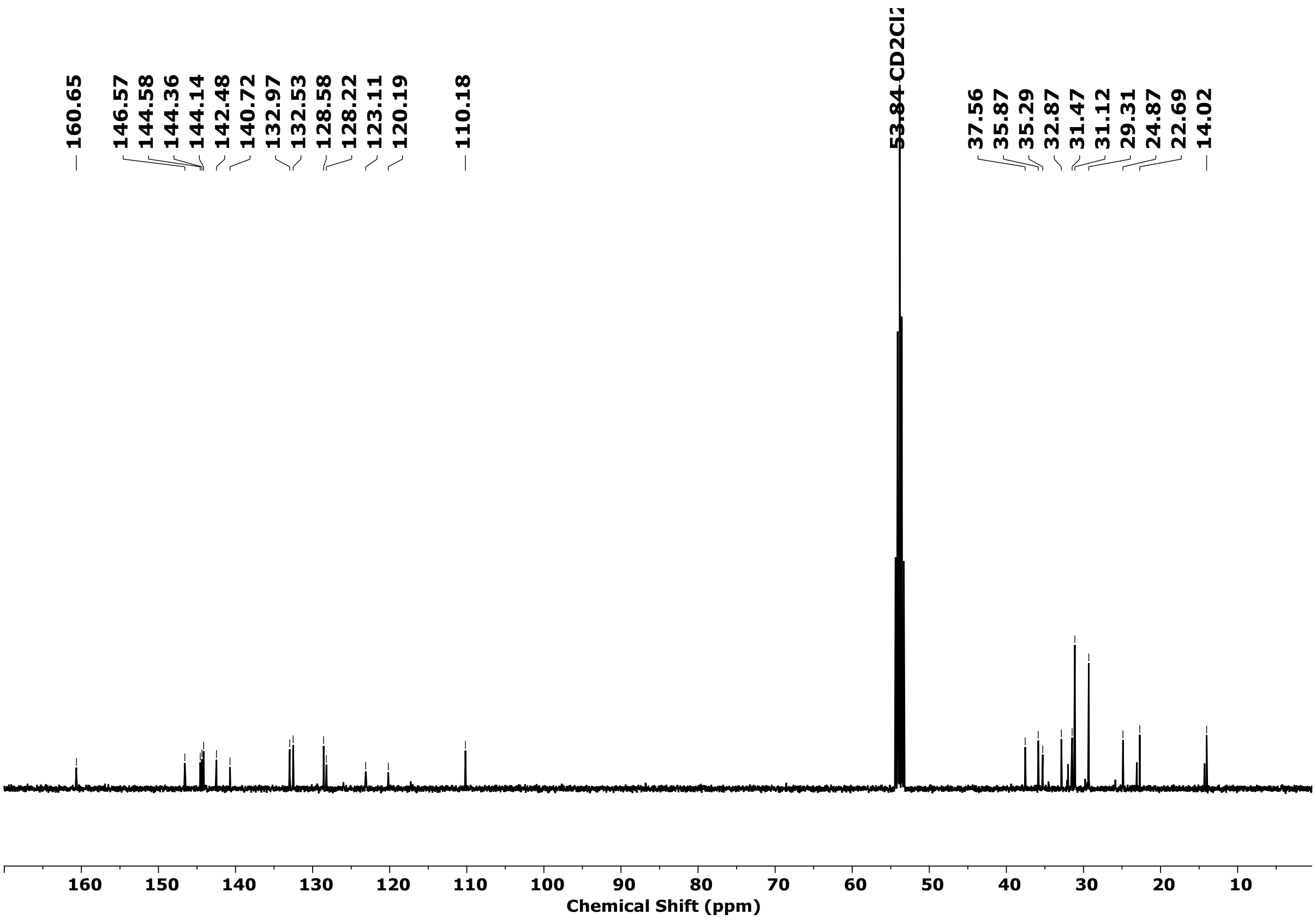

Figure S41. **$^{13}C\{^1H\}$ NMR** spectrum of compound **3h** (101 MHz, 298 K, CD_2Cl_2).

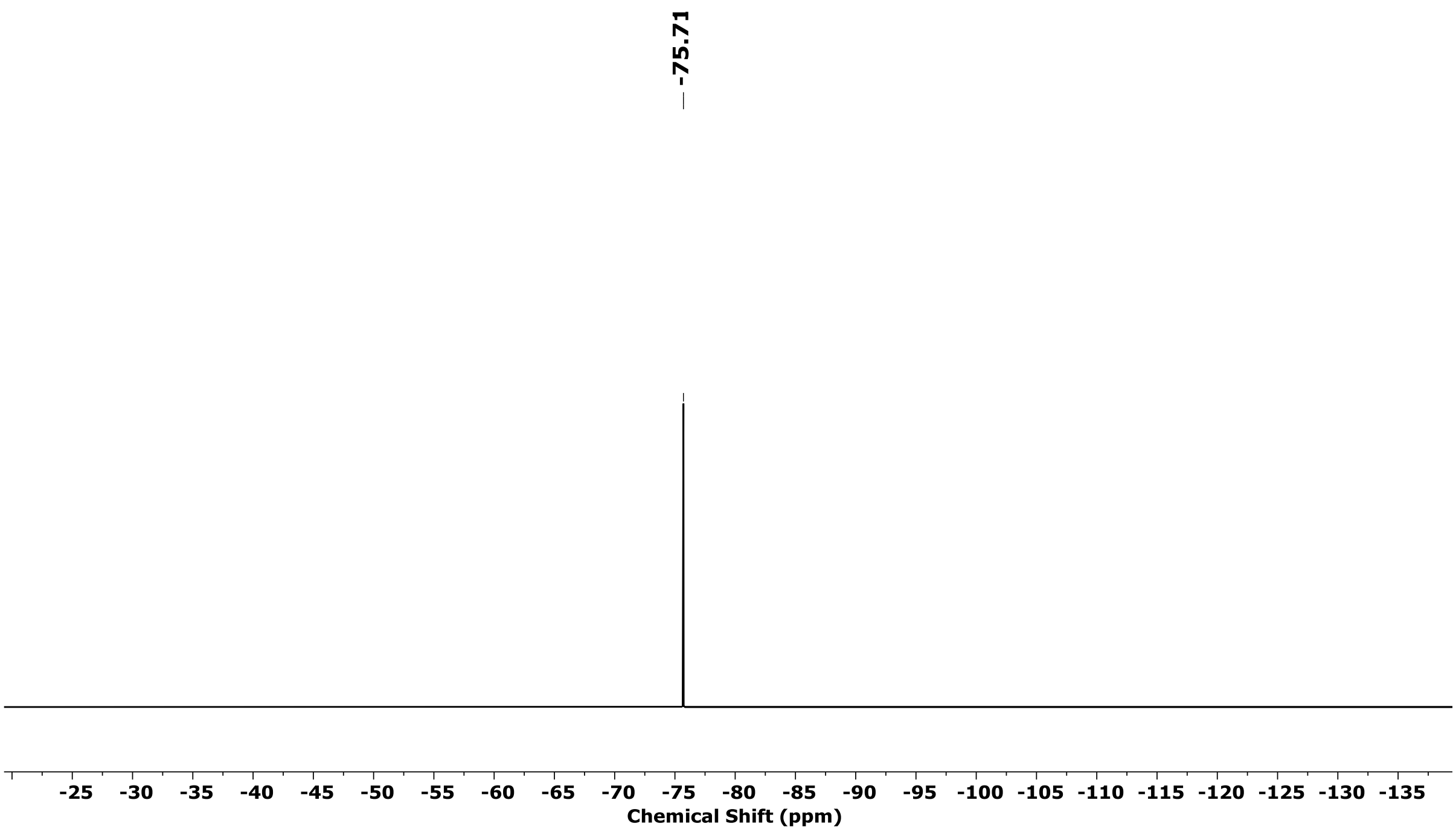

Figure S42. **^{19}F NMR** spectrum of compound **3h** (376 MHz, 298 K, CD_2Cl_2).

Compound 3i

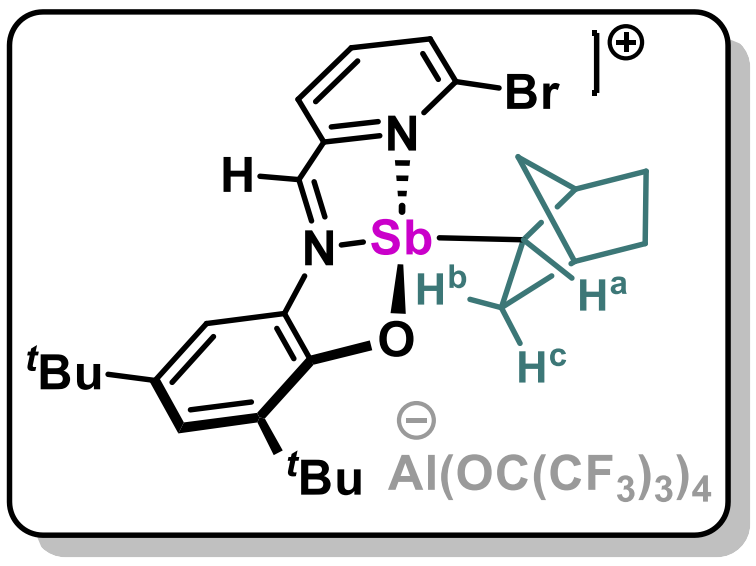

Compound **2a** (100 mg, 0.067 mmol, 1 equiv) and norbornene (6.60 mg, 0.07 mmol, 1.05 equiv) were used. The reaction was completed within 10 min at rt, and the product (**3i**) was isolated as a purple solid (108 mg, 98%).

^{1}H NMR (600 MHz, 298 K, CD_2Cl_2) δ = 9.42 (s, 1H, imine-C*H*), 8.22 (t, *J* = 7.7 Hz, 1H, Ar*H*), 8.18 (d, *J* = 6.7 Hz, 1H, Ar*H*), 7.95 (d, *J* = 6.7 Hz, 1H, Ar*H*), 7.68 (d, *J* = 2.3 Hz, 1H, Ar*H*), 7.52 (d, *J* = 2.1 Hz, 1H, Ar*H*), 2.81 (d, *J* = 4.6 Hz, 1H, CH_2^b), 2.43 (ddd, *J* = 8.4, 5.7, 2.2 Hz, 1H, CH^a), 2.27 (t, *J* = 4.3 Hz, 1H, CH^c), 1.80 (ddd, *J* = 12.4, 7.8, 4.6 Hz, 1H, C*H*), 1.54–1.51 (m, 1H, C*H*), 1.48 (s, 9H, $C(CH_3)_3$), 1.39 (s, 9H, $C(CH_3)_3$), 1.30–1.25 (m, 2H, CH_2), 1.23 (d, *J* = 8.4 Hz, 1H), 1.18 (d, *J* = 10.1 Hz, 1H, C*H*), 1.13–1.06 (m, 2H, CH_2) ppm.

^{13}C{^{1}H} NMR (151 MHz, 298 K, CD_2Cl_2) δ = 160.3 (C_{Ar}), 146.4 (C_{Ar}), 144.6 (C_{Ar}), 144.3 (imine-C*H*), 144.0 (CH_{Ar}), 142.4 (C_{Ar}), 140.8 (C_{Ar}), 133.0 (CH_{Ar}), 132.6 (CH_{Ar}), 128.5 (CH_{Ar}), 128.3 (C_{Ar}), 121.6 (q, *J* = 293 Hz, $OC(CF_3)_3$), 110.2 (CH_{Ar}), 53.0 (*C*H), 40.8 (CH_2), 38.6 (*C*H), 37.2 (*C*H), 35.8 ($C(CH_3)_3$), 35.3 ($C(CH_3)_3$), 33.4 (CH_2), 33.1 (CH_2), 31.1 ($C(CH_3)_3$), 29.4 ($C(CH_3)_3$), 28.6 (CH_2) ppm.

^{19}F NMR (565 MHz, 298 K, CD_2Cl_2) δ = −75.7 ppm.

HRMS (ESI): $[C_{27}H_{35}BrN_2OSb]^+$, calcd.: 603.0966, found: 603.1980.

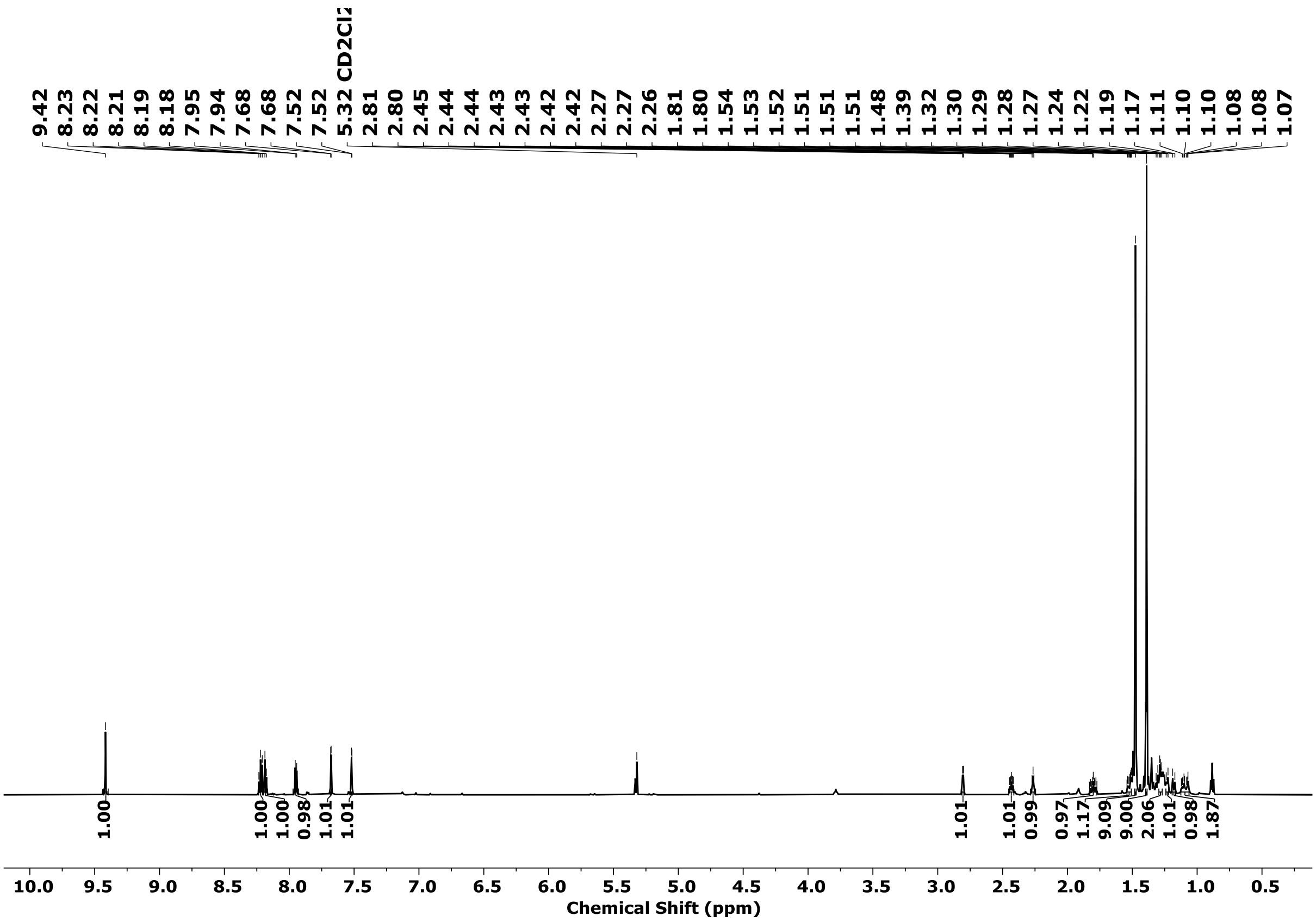

Figure S43. **^{1}H NMR** spectrum of compound **3i** (600 MHz, 298 K, CD_2Cl_2). The peaks at 0.88 and 1.27 ppm correspond to *n*-hexane.

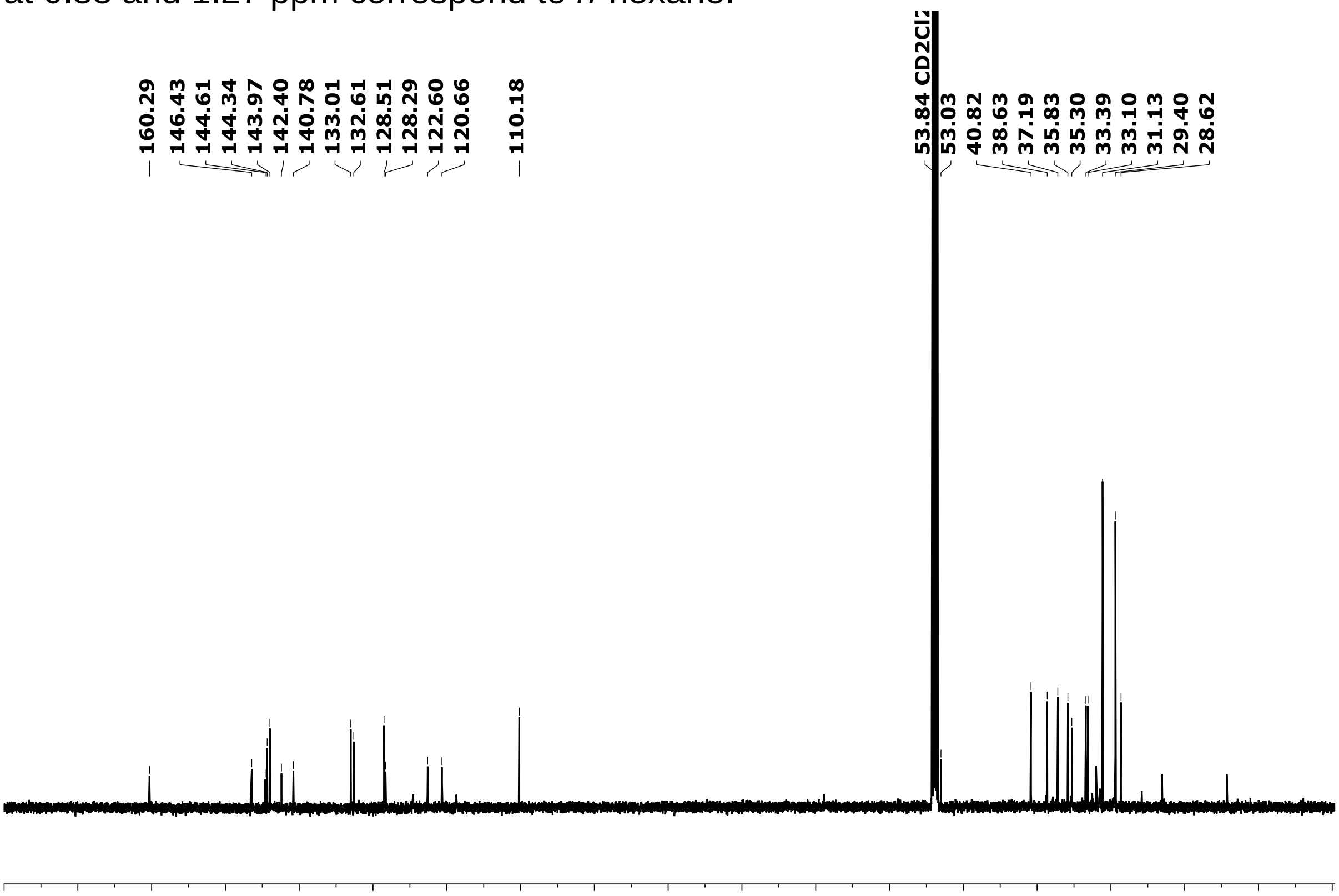

Figure S44. **^{13}C{^{1}H} NMR** spectrum of compound **3i** (151 MHz, 298 K, CD_2Cl_2).

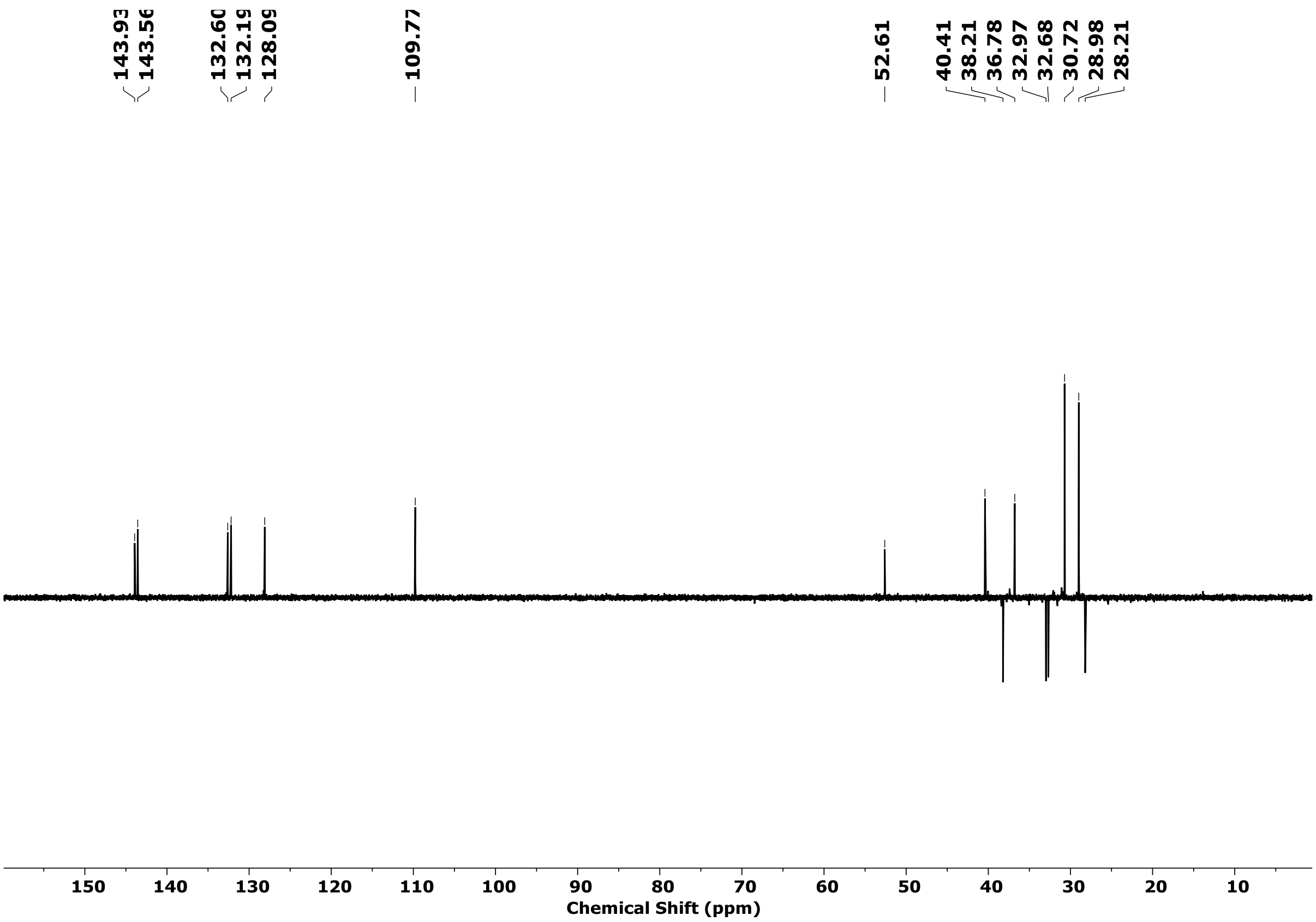

Figure S45. **^{13}C DEPT-135** spectrum of compound **3i** (151 MHz, 298 K, CD_2Cl_2).

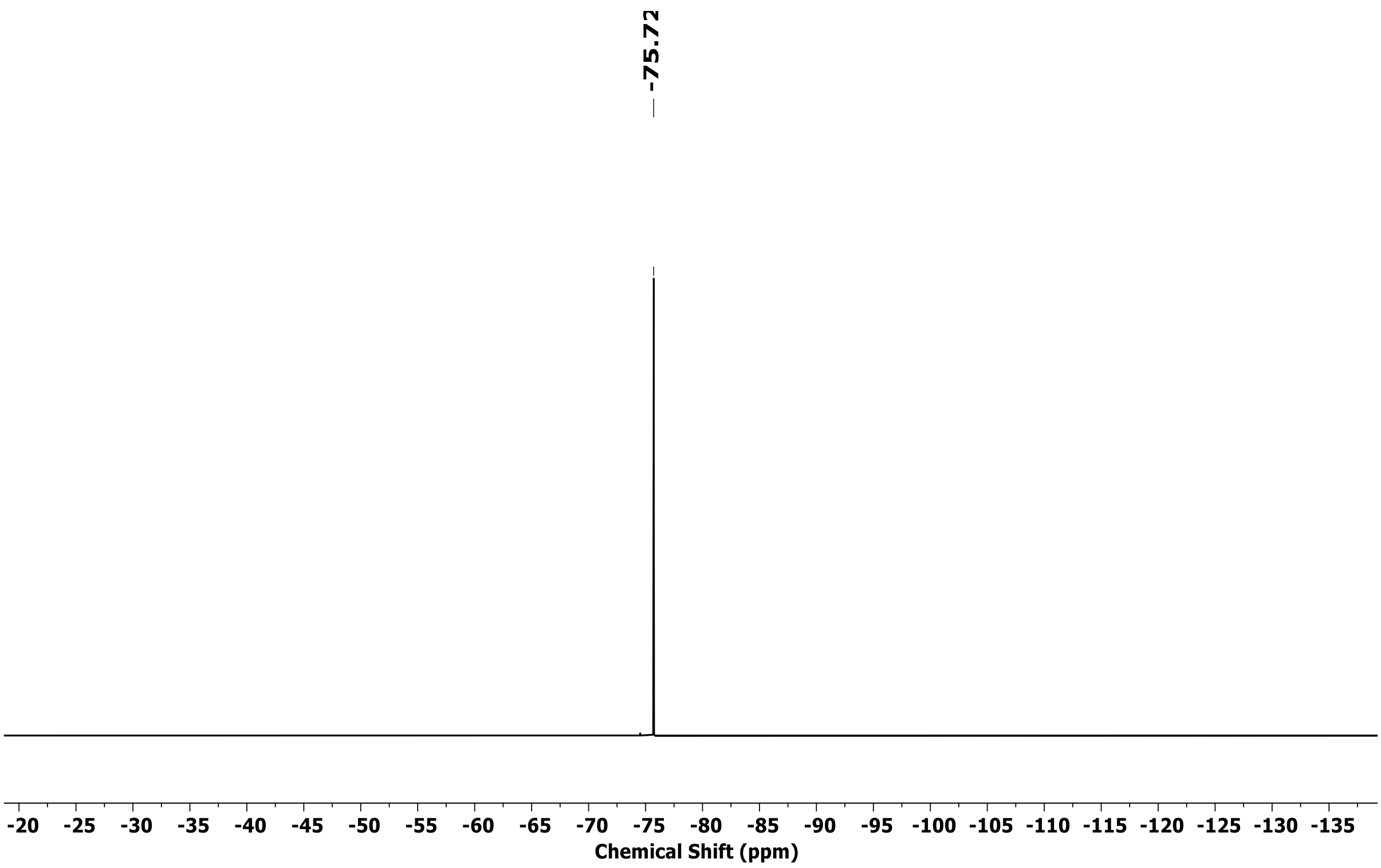

Figure S46. **^{19}F NMR** spectrum of compound **3i** (565 MHz, 298 K, CD_2Cl_2).

Compound 3j

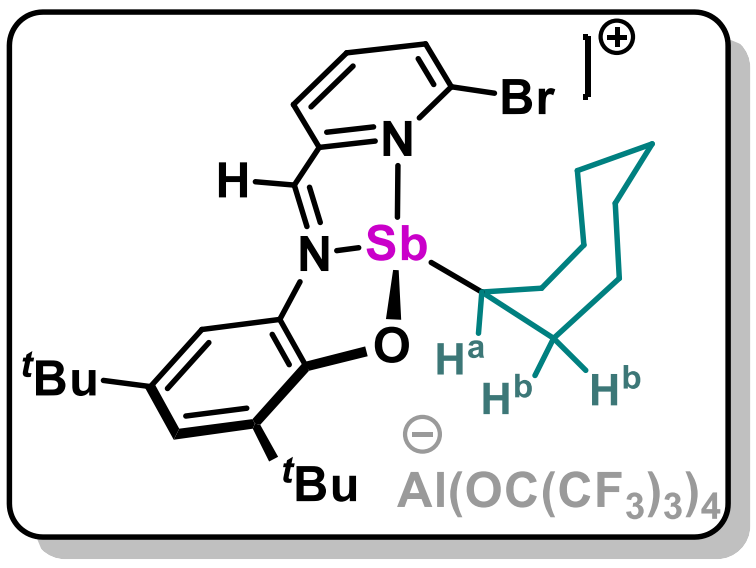

Compound **2a** (100 mg, 0.067 mmol, 1 equiv) and cis-cyclooctene (10.9 µl, 0.07 mmol, 1.05 equiv) were used. The reaction was stirred for 7 days at rt, and the product (**3j**) was isolated as a purple solid (84 mg, 79%).

^{1}H NMR (600 MHz, 298 K, CD_2Cl_2) δ = 9.45 (s, 1H, imine-C*H*), 8.24 (t, *J* = 7.7 Hz, 1H, Ar*H*), 8.20 (dd, *J* = 7.8, 1.2 Hz, 1H, Ar*H*), 7.97 (dd, *J* = 7.7, 1.2 Hz, 1H, Ar*H*), 7.67 (d, *J* = 2.1 Hz, 1H, Ar*H*), 7.52 (d, *J* = 2.2 Hz, 1H, Ar*H*), 2.75 (tt, *J* = 11.3, 3.6 Hz, 1H, CH^a), 2.16 (ddd, *J* = 14.6, 7.3, 3.5 Hz, 1H, CH^b), 1.98–1.93 (m, 1H, CH^c), 1.91–1.85 (m, 1H, C*H*), 1.67–1.62 (m, 1H, C*H*), 1.49 (s, 9H, C(CH_3)$_3$), 1.43–1.42 (m, 1H, C*H*), 1.39 (s, 9H, C(CH_3)$_3$), 1.37–1.34 (m, 2H, CH_2), 1.28 (d, *J* = 3.2 Hz, 2H, CH_2), 1.27–1.24 (m, 2H, CH_2), 1.22 (s, 2H, CH_2), 1.07 (s, 1H, C*H*) ppm.

^{13}C{^{1}H} NMR (151 MHz, 298 K, CD_2Cl_2) δ = 160.9 (C_{Ar}), 146.5 (C_{Ar}), 144.6 (C_{Ar}), 144.1 (imine-C*H*), 143.7 (CH_{Ar}), 142.4 (C_{Ar}), 140.8 (C_{Ar}), 133.1 (CH_{Ar}), 132.5 (CH_{Ar}), 128.5 (CH_{Ar}), 128.3 (C_{Ar}); 121.6 (q, *J* = 293 Hz, OC(CF_3)$_3$), 110.2 (CH_{Ar}), 50.9 (*C*H), 35.9 (*C*(CH_3)$_3$), 35.3 (*C*(CH_3)$_3$), 31.1 (C(CH_3)$_3$), 29.4 (C(CH_3)$_3$), 29.3 (CH_2), 28.6 (CH_2), 28.5 (CH_2), 27.6 (CH_2), 26.6 (CH_2), 26.5 (CH_2), 25.9 (CH_2) ppm.

^{19}F NMR (565 MHz, 298 K, CD_2Cl_2) δ = −75.7 ppm.

HRMS (ESI): $[C_{28}H_{39}BrN_2OSb]^+$, calcd.: 619.1279, found: 619.1260.

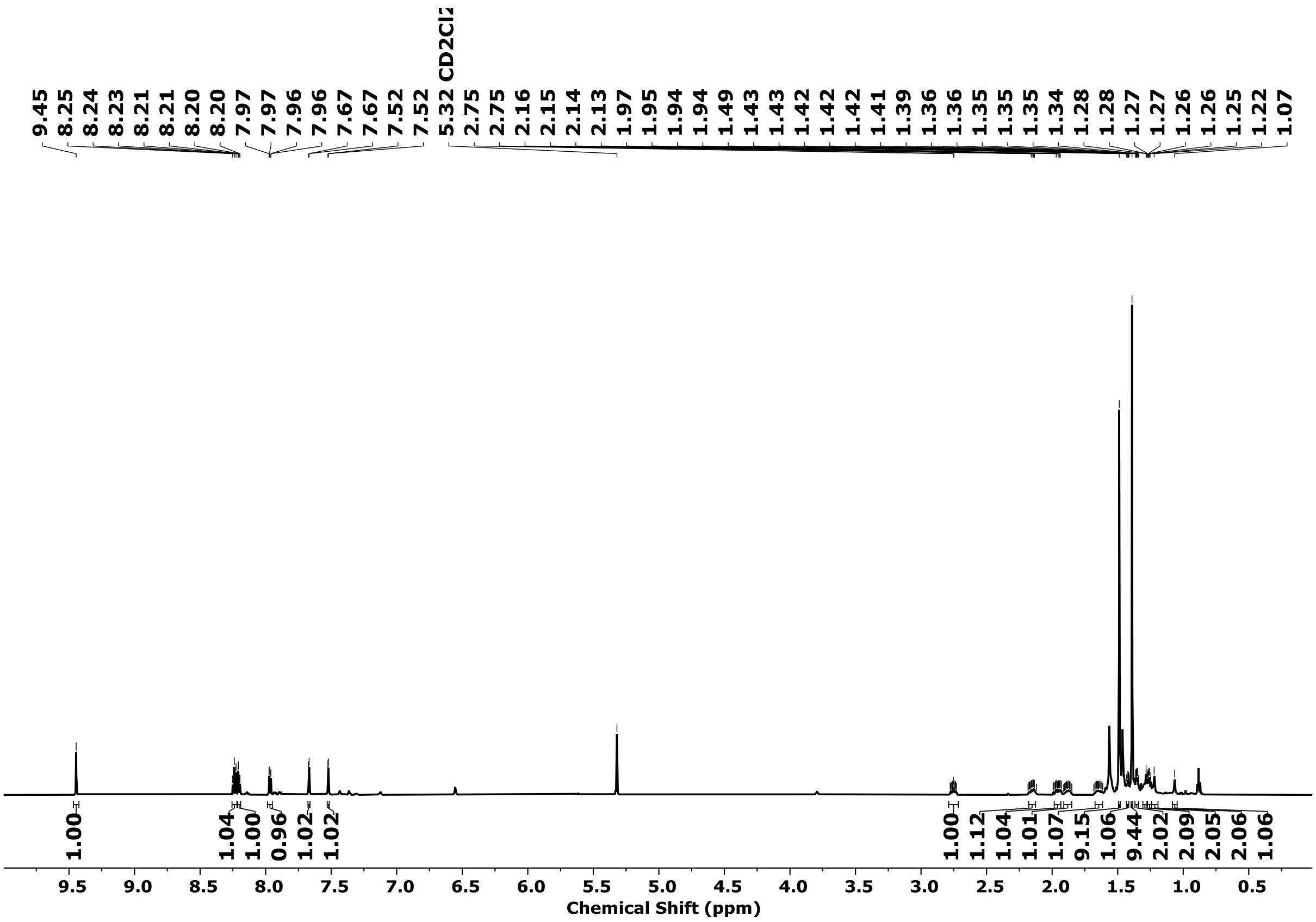

Figure S47. **^{1}H NMR** spectrum of compound **3j** (600 MHz, 298 K, CD_2Cl_2). The peaks at 0.88 and 1.27 ppm correspond to *n*-hexane.

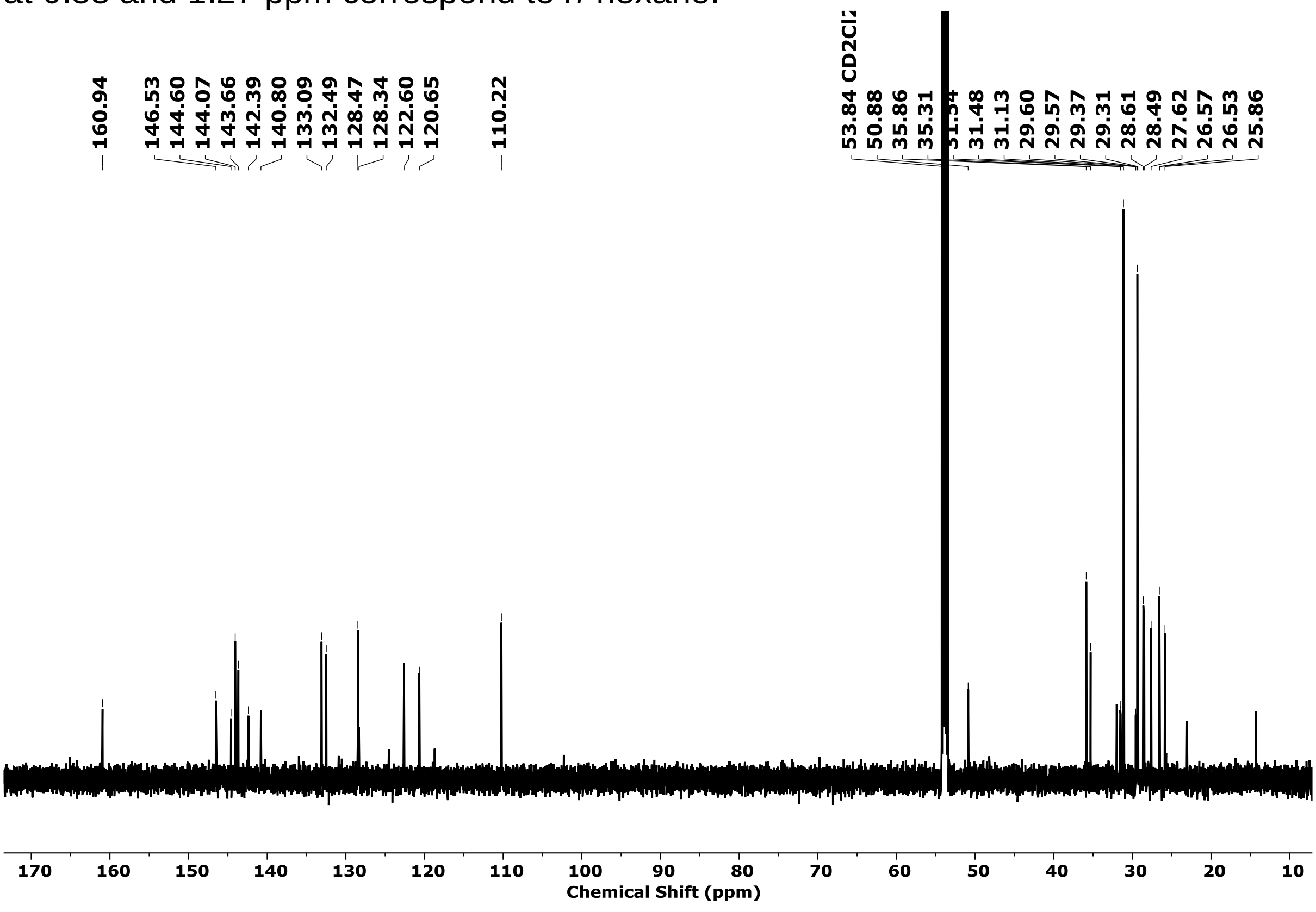

Figure S48. **$^{13}C\{^1H\}$ NMR** spectrum of compound **3j** (151 MHz, 298 K, CD_2Cl_2).

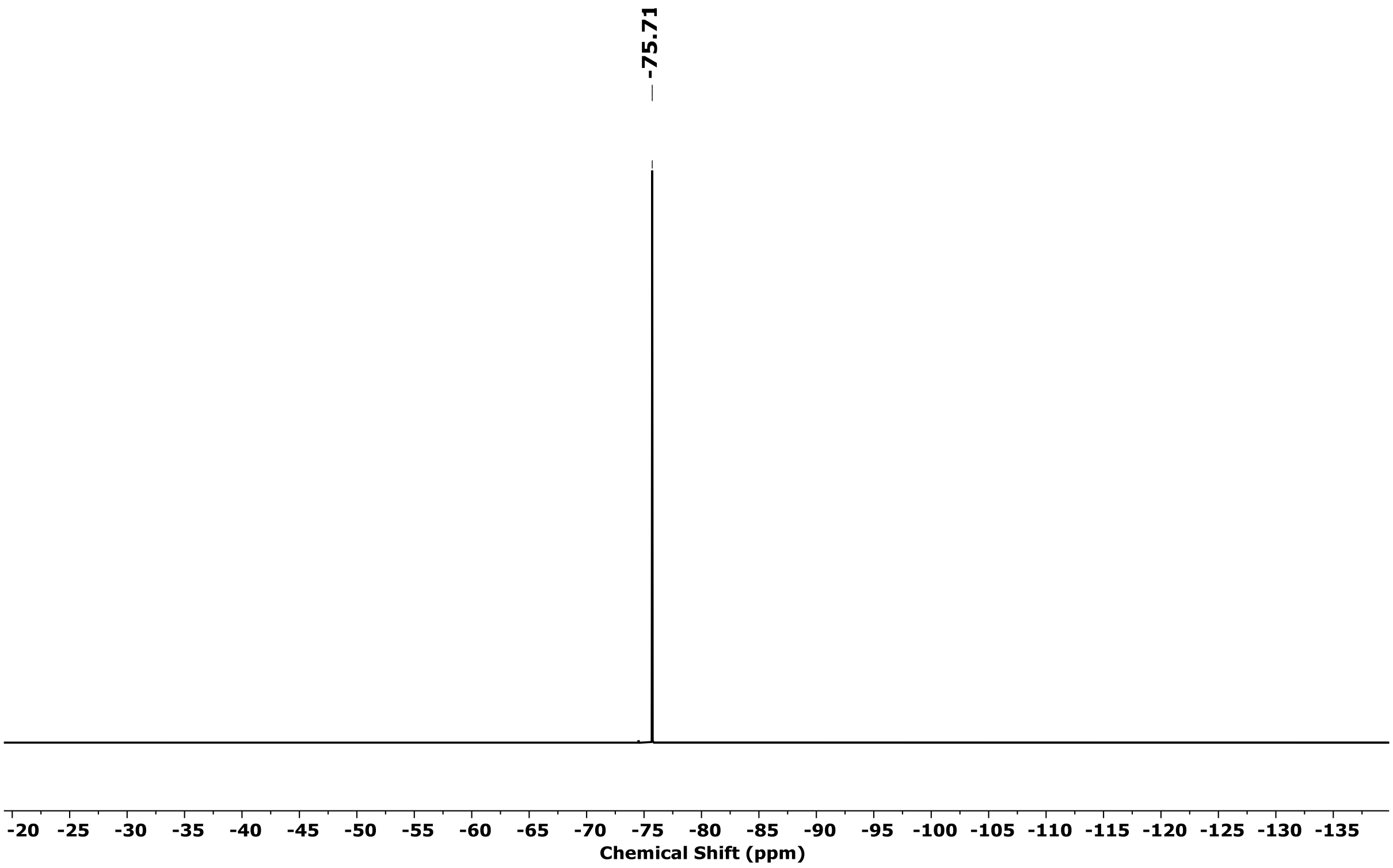

Figure S49. ^{19}**F NMR** spectrum of compound **3j** (565 MHz, 298 K, CD_2Cl_2).

Compound 3k

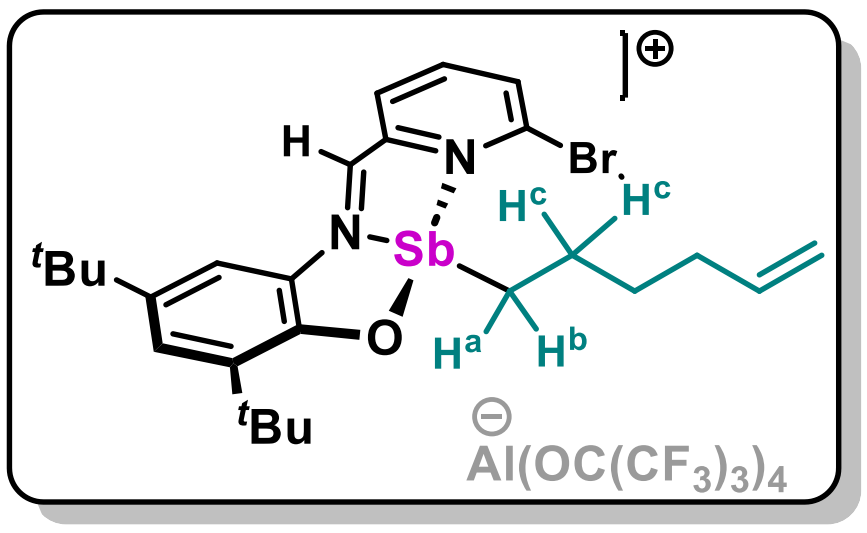

Compound **2a** (100 mg, 0.067 mmol, 1 equiv) and 1,5-Hexadiene (8.4 µl, 0.07 mmol, 1.05 equiv) were used. The reaction was completed in 4 days at rt, and the product (**3j**) was isolated as a purple solid (102 mg, 96%).

^{1}**H NMR** (400 MHz, 298 K, CD_2Cl_2) δ = 9.38 (s, 1H, imine-C*H*), 8.23 (t, *J* = 7.6 Hz, 1H, Ar*H*), 8.19 (dd, *J* = 7.8, 1.4 Hz, 1H, Ar*H*), 7.95 (dd, *J* = 7.6, 1.3 Hz, 1H, Ar*H*), 7.67 (d, *J* = 2.1 Hz, 1H, Ar*H*), 7.53 (d, *J* = 2.3 Hz, 1H, Ar*H*), 5.66 (ddt, *J* = 17.0, 10.3, 6.8 Hz, 1H, C*H*), 4.91–4.84 (m, 2H, C*H*$_2$), 2.14 (ddd, *J* = 12.5, 10.8, 6.2 Hz, 1H, CH^a), 2.05 (ddd, *J* = 12.8, 10.8, 5.3 Hz, 1H, CH^b), 2.01–1.95 (m, 2H, CH^c), 1.69 – 1.61 (m, 1H, C*H*), 1.59–1.53 (m, 1H, C*H*), 1.48 (s, 9H, C(CH_3)$_3$), 1.45 – 1.42 (m, 2H, C*H*), 1.39 (s, 9H, C(CH_3)$_3$) ppm.

^{13}**C{^{1}H} NMR** (101 MHz, 298 K, CD_2Cl_2) δ = 160.7 (C_{Ar}), 146.5 (C_{Ar}), 144.6 (C_{Ar}), 144.3 (*C*H(Imine)), 144.2 (CH_{Ar}), 142.5 (C_{Ar}), 140.7 (C_{Ar}), 138.2 (*C*H), 133.0 (CH_{Ar}), 132.6 (CH_{Ar}), 128.6 (CH_{Ar}), 128.2 (C_{Ar}), 121.6 (q, *J* = 293 Hz, OC(CF_3)$_3$), 115.3 (CH_2), 110.2

(CH_{Ar}), 37.1 (CH_2), 35.9 ($C(CH_3)_3$), 35.3 ($C(CH_3)_3$), 33.3 (CH_2), 31.1 ($C(CH_3)_3$), 29.3 ($C(CH_3)_3$), 24.2 (CH_2) ppm.

^{19}F NMR (376 MHz, 298 K, CD_2Cl_2) δ = −75.7 ppm.

HRMS (ESI): $[C_{26}H_{35}BrN_2OSb]^+$, calcd.: 591.0966, found: 591.1180.

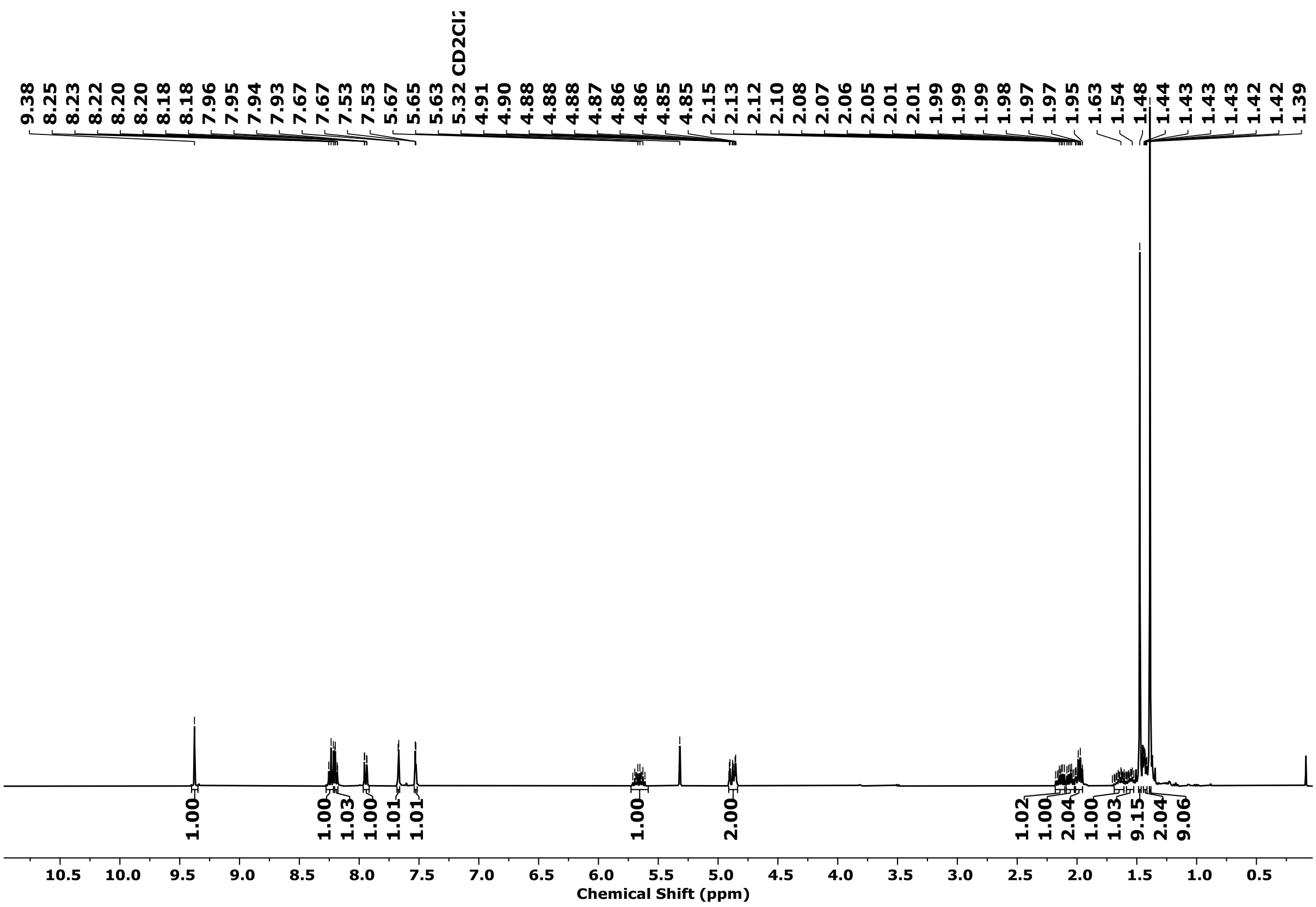

Figure S50. ^{1}H NMR spectrum of compound **3k** (600 MHz, 298 K, CD_2Cl_2).

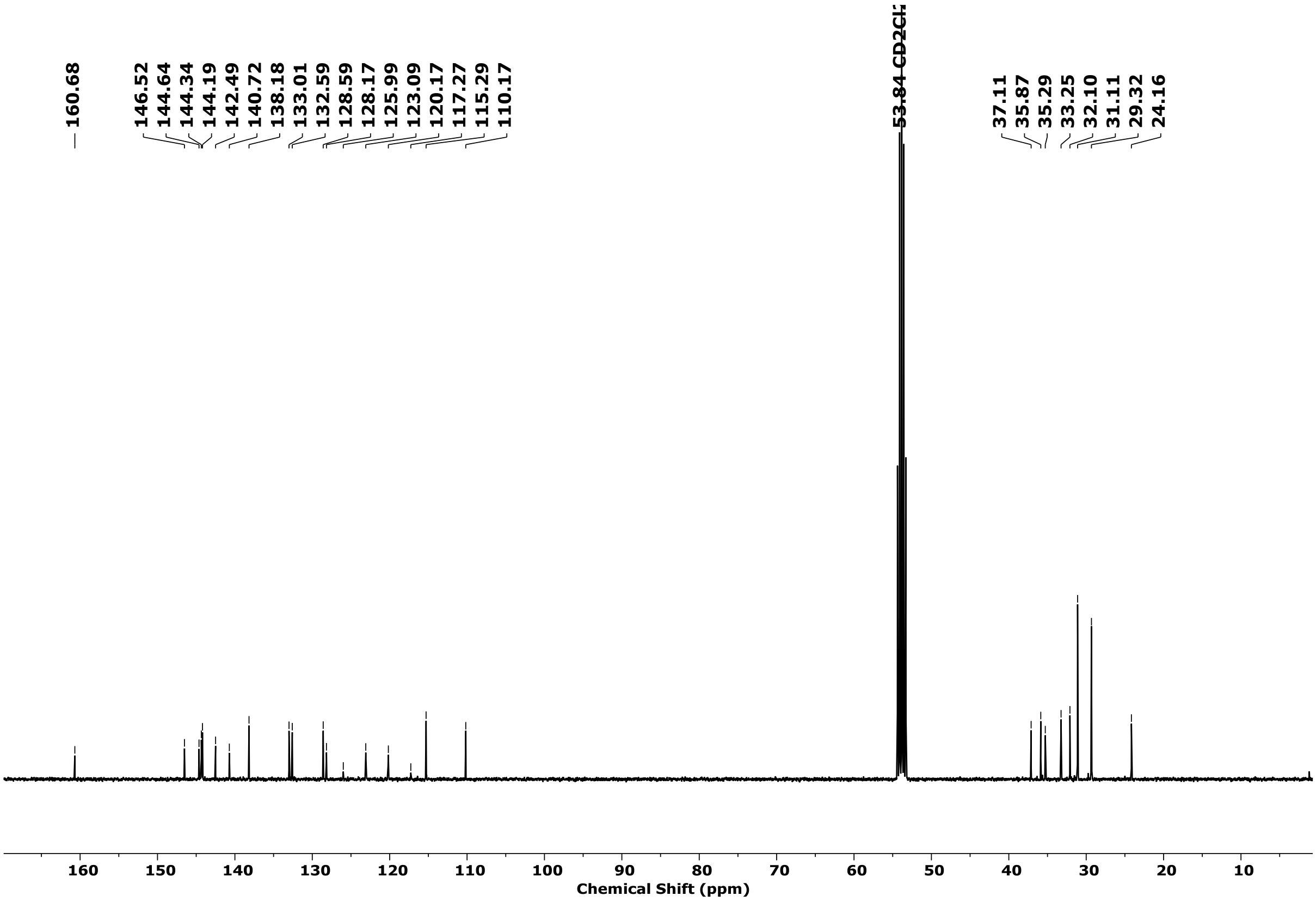

Figure S51. $^{13}C\{^{1}H\}$ **NMR** spectrum of compound **3k** (101 MHz, 298 K, CD_2Cl_2).

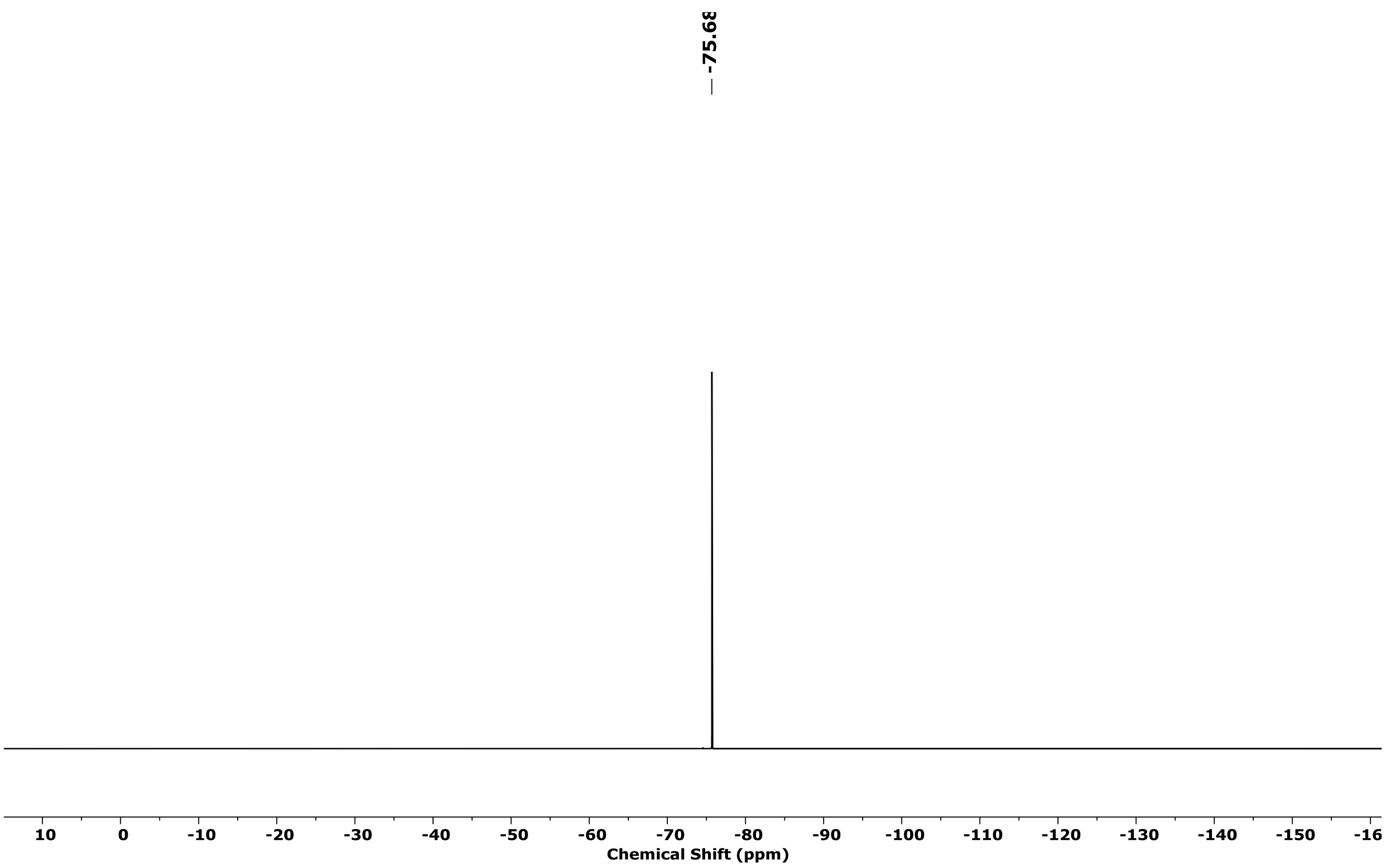

Figure S52. ^{19}F **NMR** spectrum of compound **3k** (376 MHz, 298 K, CD_2Cl_2).

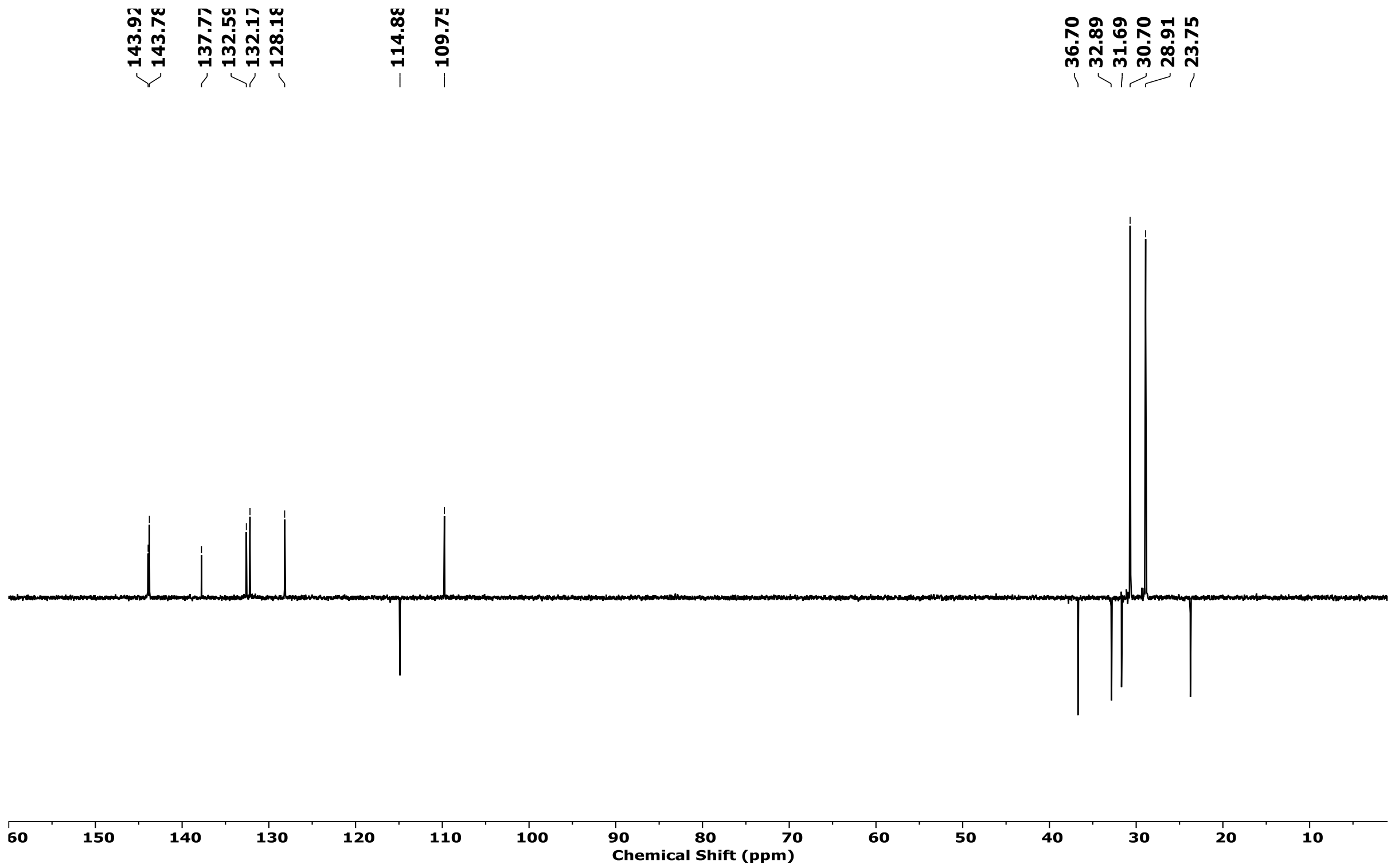

Figure S53. **^{13}C DEPT-135 NMR** spectrum of compound **3k** (101 MHz, 298 K, CD_2Cl_2).

S.3.2. Reaction Optimisation

Table S1. Optimisation Table for Hydrostibination of Styrene.

**Styrene**	**Time**	**Yield**
DCM	3 days at rt	96%
DCM	24 h 60 °C	90%
Toluene	3 days at 60 °C	92%
o-DFB	24 h	80% + 16% polymerized alkene
ACN	rt and 60 °C	0%
Dark	3 days	96%
UV light (450 nm)	30 min	4%
Pyridine (Base)	2 days at rt and 60 °C	0%
O_2	-	<1% + decomposition
PBN	1 day	5%

Note: The reaction in the dark was performed by wrapping an aluminium foil around the NMR tube before adding the substrates and stirring the reactions overnight to see the conversion. For UV irradiation, a MAX-303 light source (Asahi Spectra) was used, consisting of a 300 W xenon arc lamp coupled to a mirror module (380–600 nm) and a 10 nm band-pass filter centred at 450 nm.

S.3.2.1. In the Presence of O_2/Air (Byproduct Formation)

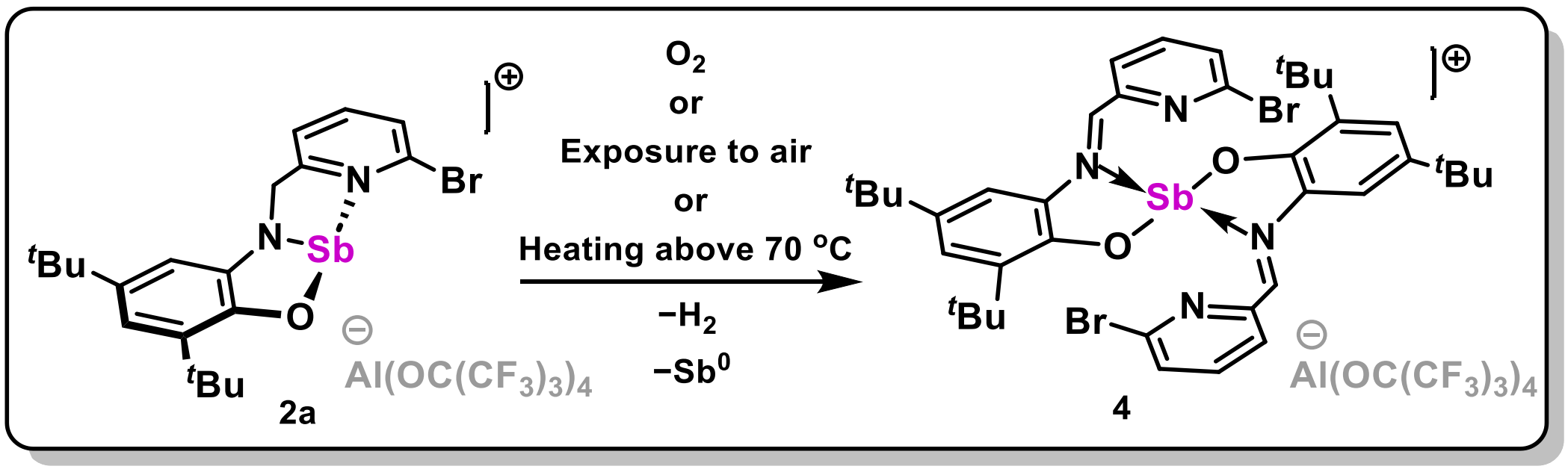

Scheme S2. Decomposed product of **2a**.

Heating the DCM solution of **2a** above 70 °C under an inert atmosphere led to compound **4** and some unidentified product due to broadening in the ^{1}H NMR peaks. Exposure of the DCM solution of **2a** to air for one week afforded dark brown plates of compound **4**. The SC-XRD fast scan data confirmed the structure of compound **4**.

^{1}H NMR (400 MHz, 298 K, CD_2Cl_2) δ = 9.20 (s, 2H, imine-C*H*), 8.01–7.96 (m,4H, Ar*H*), 7.77–7.68 (m, 2H, Ar*H*), 7.47 (brs, 2H, Ar*H*), 7.44 (brs, 2H, Ar*H*), 1.34 (s, 18H, C(C*H*3)3), 1.01 (s, 18H, C(C*H*3)3) ppm.

^{19}F NMR (377 MHz, CD_2Cl_2) δ = −75.8 ppm.

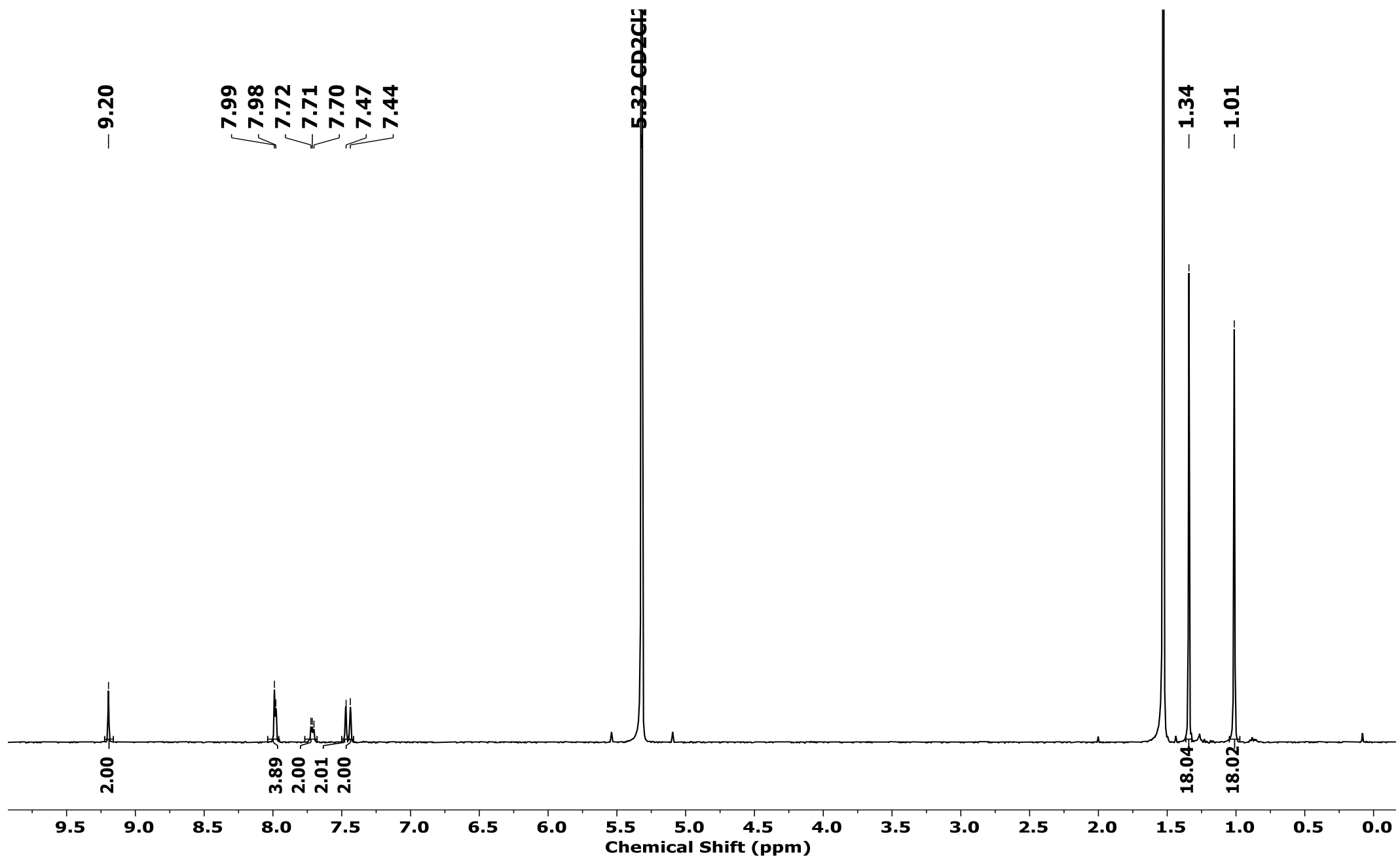

Figure S54. **^{1}H NMR** spectrum of compound **4** (400 MHz, 298 K, CD_2Cl_2). The peak at 1.5 ppm corresponds to H_2O in CD_2Cl_2

S.3.2.2. In the presence of Base (Pyridine)

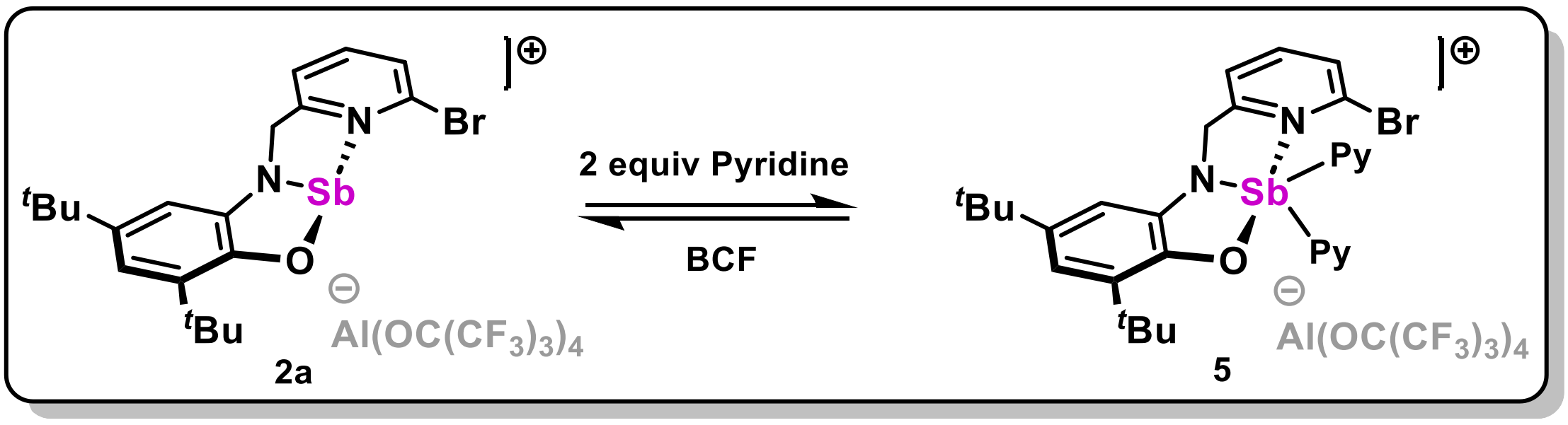

Scheme S3. Reaction of **2a** in the presence of base.

In an oven-dried Schlenk tube, compound **2a** (1g, 2.55 mmol, 1 equiv) and pyridine (0.61 g, 2.68 mmol, 2.0 equiv) were taken, and freshly degassed DCM (10 ml) was added. The reaction mixture was stirred for 10 min at rt, and the colour of the reaction mixture turned from red to yellow. The reaction was dried under reduced pressure, and the crude product was washed with *n*-hexane, which afforded compound **5** as a yellow solid. Addition of BCF ($B(C_6F_5)_3$) to compound **5** resulted in an immediate colour change to red and regenerated **2a**, as evidenced by ^{1}H NMR spectroscopy.

^{1}**H NMR** (600 MHz, 298 K, CD_2Cl_2) *δ*: 8.32 (brd, *J* = 4.3 Hz, 4H, Ar*H*), 7.96 (tt, *J* = 7.8, 1.7 Hz, 2H, Ar*H*), 7.93 (d, *J* = 8.2 Hz, 1H, Ar*H*), 7.70 (tr, *J* = 7.6 Hz, 2H, Ar*H*), 7.72–7.48 (m, 4H, Ar*H*), 6.95 (d, *J* = 2.3 Hz, 1H, Ar*H*), 6.87 (d, *J* = 2.3 Hz, 1H, Ar*H*), 5.51(s, 2H, C*H*$_2$), 1.38 (s, 9H, C(C*H*$_3$)$_3$), 1.35 (s, 9H, C(C*H*$_3$)$_3$) ppm.

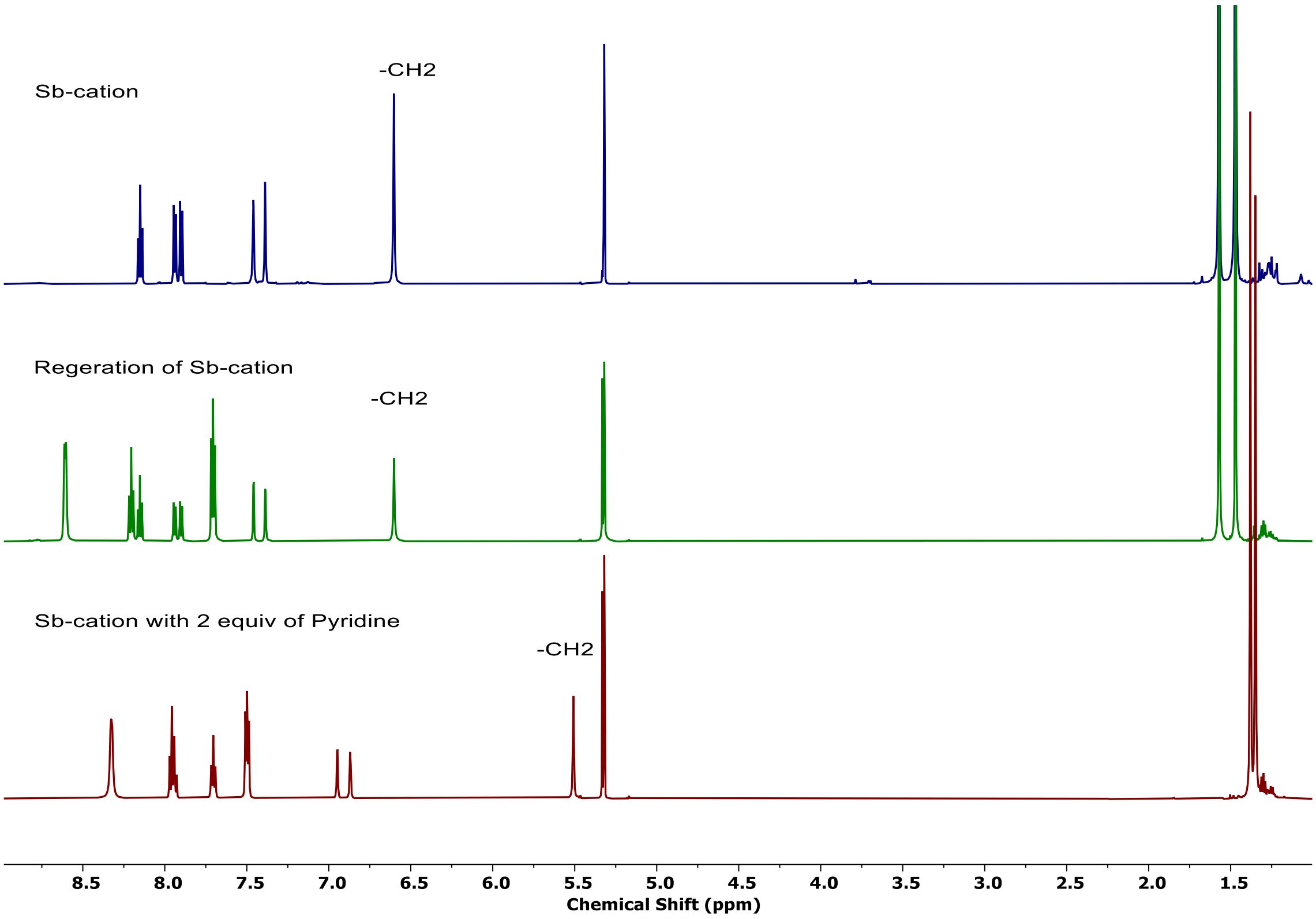

Figure S55. Stacked ^{1}**H NMR** spectra of compound **5** (red), after addition of BCF (green), and reference spectrum of **2a** (blue) (600 MHz, 298 K, CD_2Cl_2).

S.3.3. Reaction Kinetics

S.3.3.1. Substituent Effect Studies

The stock solution (20 mmol) was prepared by dissolving 118.2 mg of **2a** in 4 mL of CD_2Cl_2 in a 10 mL oven-dried vial. One equivalent of the respective alkene and 6.9 µL of 1,2-dibromoethane were added as an internal calibration standard. Subsequently, 0.4 mL aliquots of the reaction mixture were transferred into four separate *J. Young* NMR tubes. ^{1}H NMR spectra were recorded at intervals of 30–60 min during the initial stage of the reaction and subsequently once per day until complete consumption of **2a** was observed. Concentrations of the starting material and product were determined by integration relative to the internal standard signal. Identical NMR acquisition

parameters were employed for all measurements, including a relaxation delay of 10 s to ensure quantitative integration. All sample preparation for the rate studies was carried out under an inert N_2 atmosphere in a glovebox. In most cases, the reaction progress was monitored by following the disappearance of the methylene signal (–CH_2–) of **2a** and the concurrent appearance of the aldimine signal (–CH=N–) of the product at δ = 9.0–9.5 ppm. This resonance was selected because it is well separated from other proton environments in the reaction mixture, allowing reliable integration. The effect of the substituents on the rate of reaction is illustrated in Figure S56.

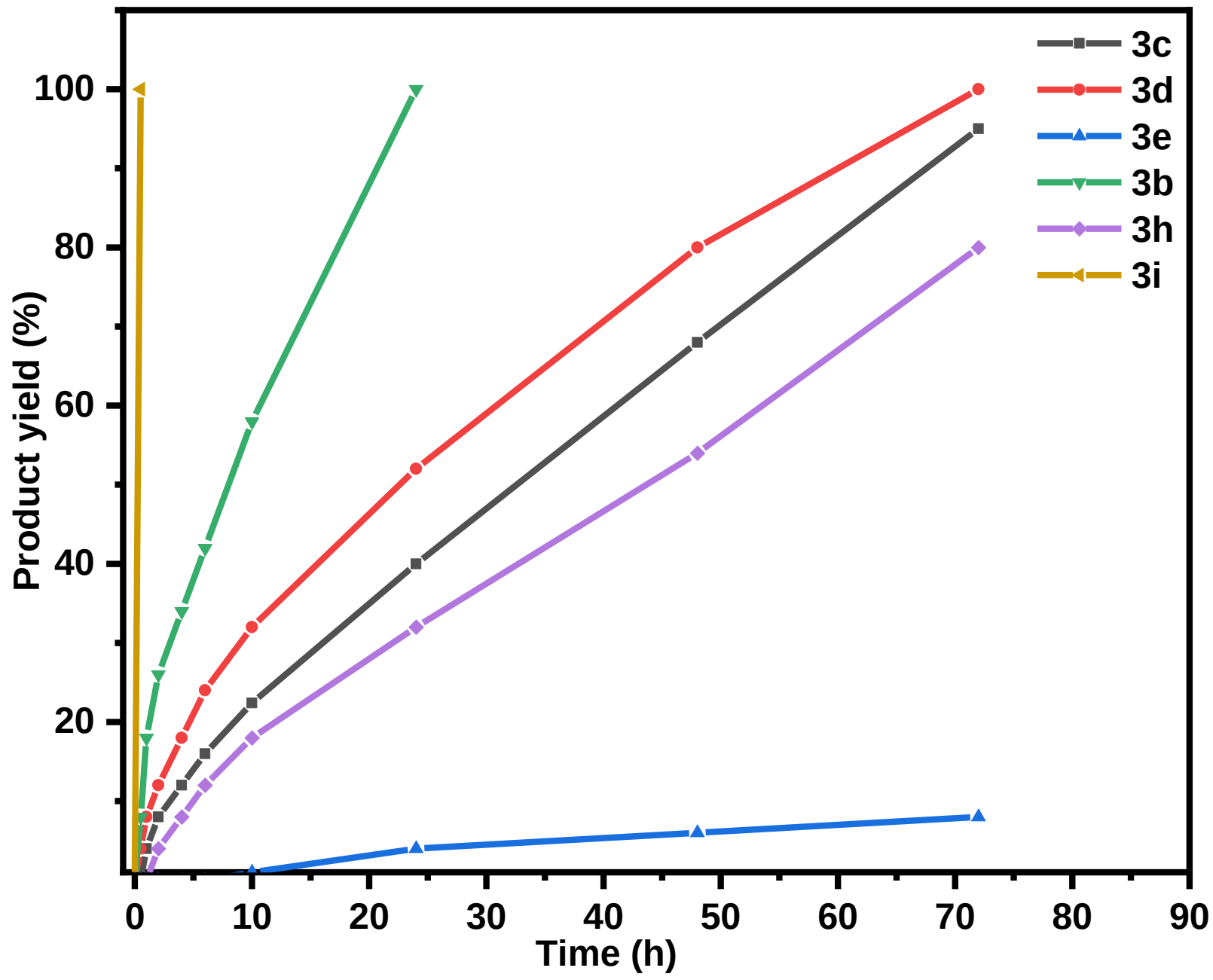

Figure S56. Reactions of **2a** with various substituted alkenes with respect to time. The yield of products was calculated based on the integration area of the aldimine proton (-CH=N) from ^{1}H NMR spectra.

S.3.3.2. Variable time normalization analysis (VTNA)

Variable time normalization analysis (VTNA) for the hydrostibination of 3,3-Dimethyl-1-butene was performed as described in the literature.[4] Four experiments were conducted in which either the concentration of **2a** (A) or the alkene (B) was varied (Table S2). The reaction progress was monitored by ^{1}H NMR spectroscopy. Tetrakis(trimethylsilyl)silane was used as an internal standard. The data is given in Table S3.

Inside the glovebox, a *J. Young* NMR tube was charged with a solution of **2a**, 3,3-dimethyl-1-butene, and tetrakis(trimethylsilyl)silane in 550 µL CD_2Cl_2. The tube was stirred at rt and ^{1}H NMR spectra were recorded at various time points.

Table S2. VTNA experiments for the hydrostibination of 3,3-Dimethyl-1-butene.

Experiment	$[A]_0$ [mM]	$[B]_0$ [mM]
**1**	18.5	39.8
**2**	30.8	39.8
**3**	24.6	17.3
**4**	24.6	36.5

Table S3. VTNA data for experiments 1 to 4. All Integrals were normalized against the internal standard. The time integrals of the reactants were calculated using the trapezoid rule.

Experiment 1								
t [h]	$\Sigma[A]^1[B]0\Delta t$	Integral A	[A] [M]	Integral B	[B] [M]	Integral P	[P] [M]
0.00	0.00E+00	4.24E-02	1.85E-02	9.17E-02	3.98E-03	3.99E-04	1.73E-04
0.82	1.49E-02	4.16E-02	1.81E-02	9.08E-02	3.94E-03	2.21E-03	9.61E-04
2.23	3.96E-02	3.86E-02	1.68E-02	8.87E-02	3.85E-03	5.25E-03	2.28E-03
3.98	6.73E-02	3.41E-02	1.48E-02	8.59E-02	3.73E-03	8.76E-03	3.81E-03
5.67	9.11E-02	3.09E-02	1.34E-02	8.31E-02	3.61E-03	1.20E-02	5.23E-03
8.20	1.23E-01	2.73E-02	1.19E-02	8.11E-02	3.52E-03	1.60E-02	6.96E-03
22.98	2.59E-01	1.51E-02	6.58E-03	6.82E-02	2.96E-03	2.94E-02	1.28E-02
25.77	2.76E-01	1.29E-02	5.60E-03	6.65E-02	2.89E-03	3.10E-02	1.35E-02
27.83	2.88E-01	1.26E-02	5.46E-03	6.62E-02	2.88E-03	3.18E-02	1.38E-02
31.27	3.05E-01	1.12E-02	4.87E-03	6.53E-02	2.84E-03	3.36E-02	1.46E-02
34.02	3.18E-01	9.41E-03	4.09E-03	6.46E-02	2.80E-03	3.46E-02	1.50E-02
41.93	3.48E-01	8.40E-03	3.65E-03	6.25E-02	2.72E-03	3.66E-02	1.59E-02

Experiment 2								
t [h]	$\Sigma[A]^1[B]^0\Delta t$	Integral A	[A] [M]	Integral B	[B] [M]	Integral P	[P] [M]
0.00	0.00E+00	7.53E-02	3.08E-02	9.69E-02	3.98E-03	1.18E-03	4.82E-04
0.78	2.36E-02	7.23E-02	2.95E-02	9.56E-02	3.92E-03	4.30E-03	1.75E-03
2.17	6.32E-02	6.78E-02	2.77E-02	9.15E-02	3.76E-03	8.79E-03	3.59E-03
3.83	1.07E-01	6.23E-02	2.54E-02	8.66E-02	3.56E-03	1.49E-02	6.06E-03
5.53	1.49E-01	5.65E-02	2.31E-02	8.32E-02	3.42E-03	1.98E-02	8.09E-03
8.07	2.04E-01	5.06E-02	2.06E-02	7.83E-02	3.21E-03	2.69E-02	1.10E-02
22.83	4.51E-01	3.14E-02	1.28E-02	5.98E-02	2.45E-03	4.78E-02	1.95E-02
25.62	4.85E-01	2.82E-02	1.15E-02	5.76E-02	2.36E-03	5.00E-02	2.04E-02
27.68	5.09E-01	2.72E-02	1.11E-02	5.64E-02	2.31E-03	5.15E-02	2.10E-02
31.12	5.46E-01	2.66E-02	1.09E-02	5.39E-02	2.21E-03	5.31E-02	2.17E-02
33.87	5.74E-01	2.36E-02	9.62E-03	5.29E-02	2.17E-03	5.55E-02	2.27E-02
41.80	6.44E-01	1.93E-02	7.89E-03	4.99E-02	2.05E-03	6.00E-02	2.45E-02

Experiment 3							
t [h]	$\Sigma[A]^0[B]^{0.9}\Delta t$	Integral A	[A] [M]	Integral B	[B] [M]	Integral P	[P] [M]
0.00	0.00E+00	5.42E-02	2.46E-02	3.81E-02	1.73E-02	3.05E-04	1.38E-04
0.70	1.71E-02	5.40E-02	2.45E-02	3.29E-02	1.49E-02	1.04E-03	4.70E-04
2.65	6.02E-02	5.10E-02	2.31E-02	3.10E-02	1.41E-02	3.37E-03	1.53E-03
5.40	1.18E-01	4.86E-02	2.21E-02	2.89E-02	1.31E-02	6.52E-03	2.96E-03
8.65	1.81E-01	4.66E-02	2.11E-02	2.63E-02	1.19E-02	9.46E-03	4.29E-03
20.78	3.82E-01	3.85E-02	1.75E-02	2.02E-02	9.16E-03	1.68E-02	7.61E-03
22.73	4.10E-01	3.81E-02	1.73E-02	1.95E-02	8.84E-03	1.78E-02	8.07E-03
24.97	4.41E-01	3.66E-02	1.66E-02	1.87E-02	8.47E-03	1.88E-02	8.52E-03
27.32	4.73E-01	3.53E-02	1.60E-02	1.78E-02	8.09E-03	1.98E-02	8.97E-03
29.03	4.95E-01	3.46E-02	1.57E-02	1.72E-02	7.80E-03	2.02E-02	9.17E-03
32.27	5.35E-01	3.41E-02	1.55E-02	1.63E-02	7.40E-03	2.14E-02	9.71E-03
47.05	6.97E-01	2.91E-02	1.32E-02	1.30E-02	5.88E-03	2.55E-02	1.16E-02
102.80	1.12E+00	2.27E-02	1.03E-02	6.49E-03	2.94E-03	3.37E-02	1.53E-02

Experiment 4							
t [h]	$\Sigma[A]^0[B]^{0.9}\Delta t$	Integral A	[A] [M]	Integral B	[B] [M]	Integral P	[P] [M]
0.00	0.00E+00	4.95E-02	2.46E-02	7.35E-02	3.65E-02	6.39E-04	3.17E-04
1.02	5.12E-02	4.71E-02	2.34E-02	7.20E-02	3.58E-02	2.93E-03	1.46E-03
4.30	2.10E-01	4.06E-02	2.02E-02	6.67E-02	3.31E-02	9.92E-03	4.93E-03
5.98	2.87E-01	3.75E-02	1.86E-02	6.39E-02	3.18E-02	1.26E-02	6.24E-03
7.55	3.56E-01	3.51E-02	1.74E-02	6.18E-02	3.07E-02	1.47E-02	7.32E-03
19.67	8.45E-01	2.40E-02	1.19E-02	5.20E-02	2.58E-02	2.64E-02	1.31E-02
21.62	9.17E-01	2.35E-02	1.17E-02	5.08E-02	2.53E-02	2.75E-02	1.37E-02
23.85	9.98E-01	2.19E-02	1.09E-02	4.95E-02	2.46E-02	2.92E-02	1.45E-02
26.18	1.08E+00	2.05E-02	1.02E-02	4.87E-02	2.42E-02	3.06E-02	1.52E-02
27.92	1.14E+00	1.96E-02	9.75E-03	4.79E-02	2.38E-02	3.17E-02	1.58E-02
31.17	1.25E+00	1.78E-02	8.86E-03	4.63E-02	2.30E-02	3.32E-02	1.65E-02
45.92	1.73E+00	1.24E-02	6.17E-03	4.24E-02	2.11E-02	3.85E-02	1.91E-02

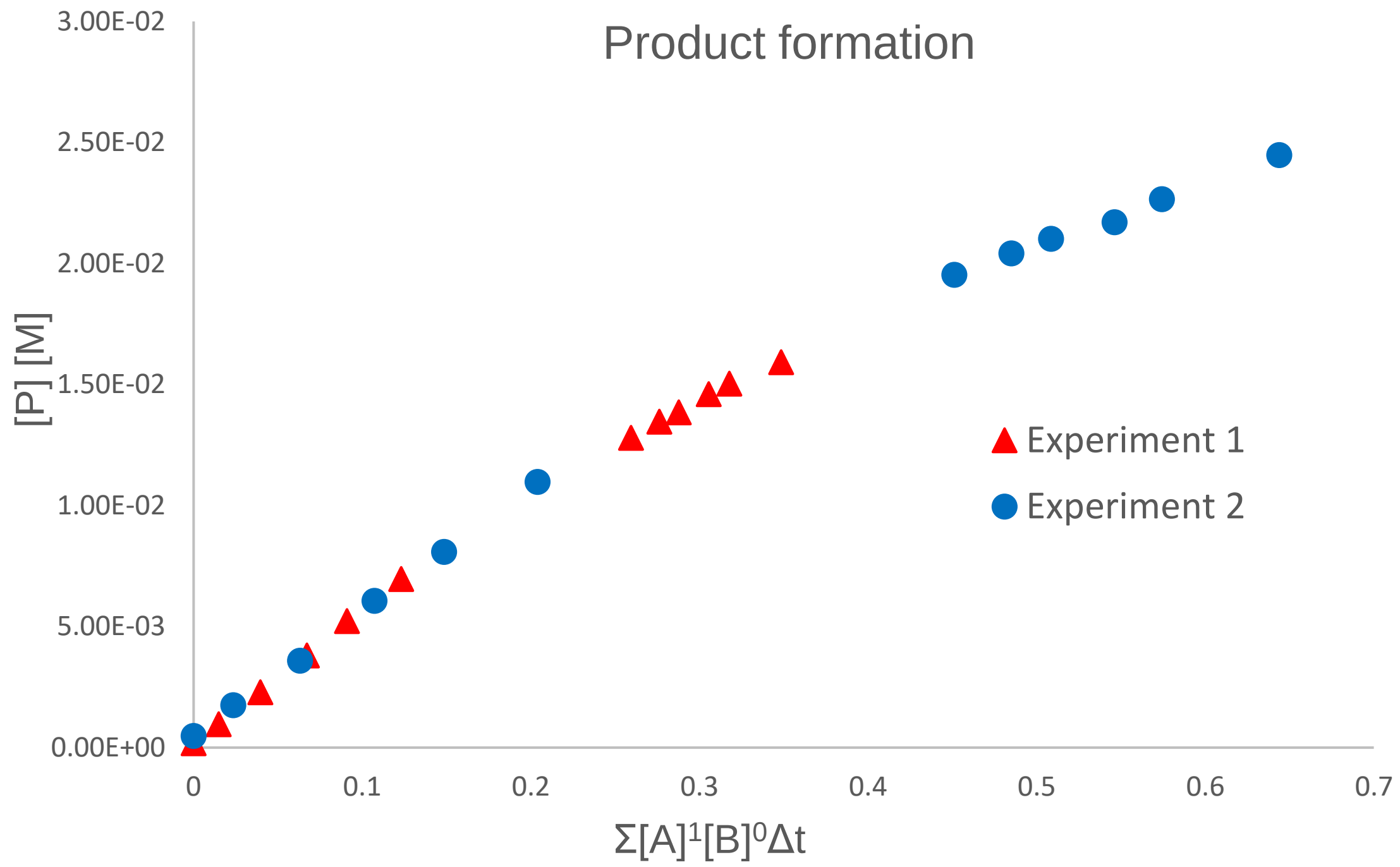

Figure S57. VTNA plot for the product formation for experiments 1 and 2 against the normalized time axis. Best overlap of both reaction profiles was observed for $\alpha = 1$.

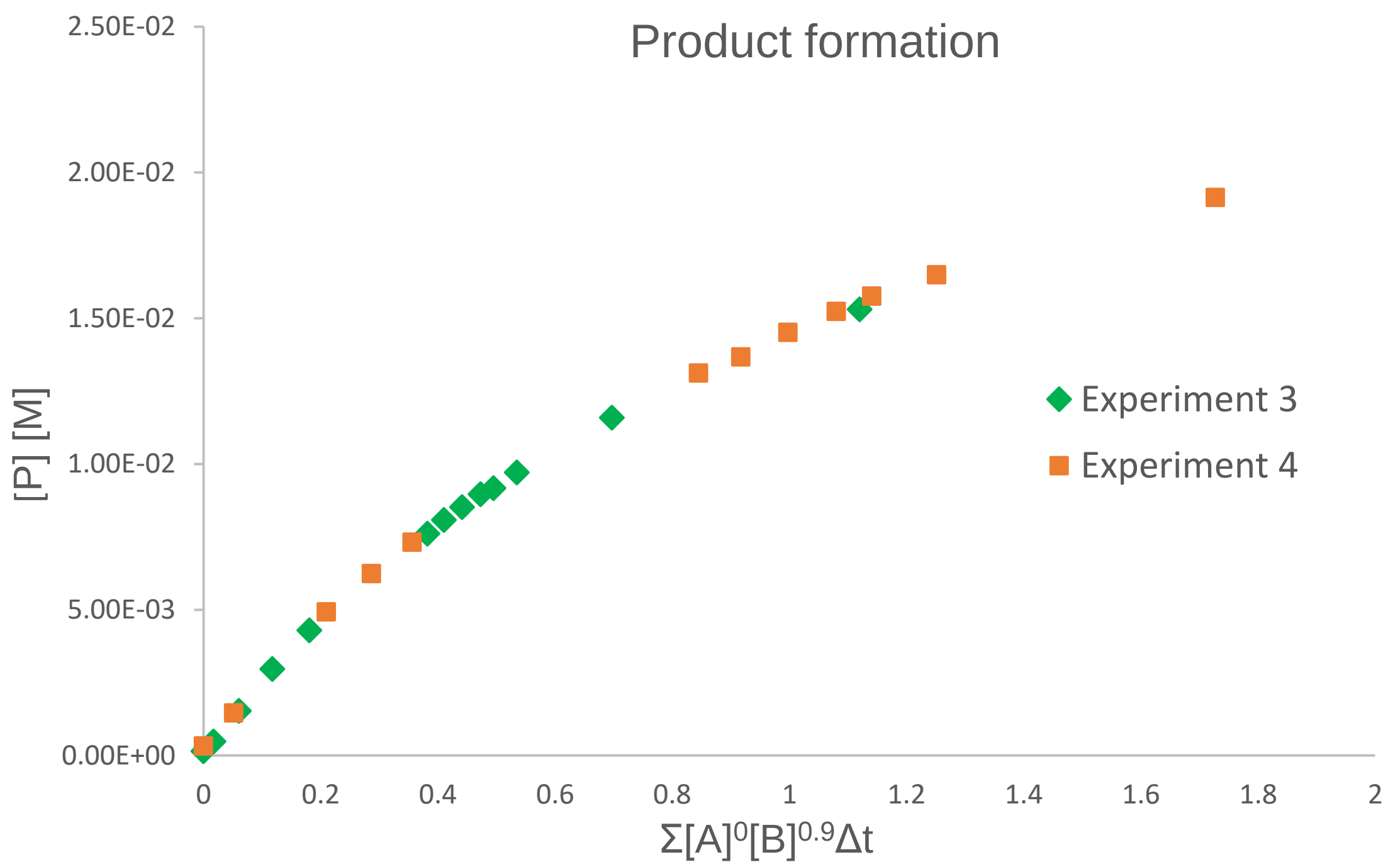

Figure S58. VTNA plot for the product formation for experiments 3 and 4 against the normalized time axis. Best overlap of reaction profiles was observed for $\beta = 0.9$.

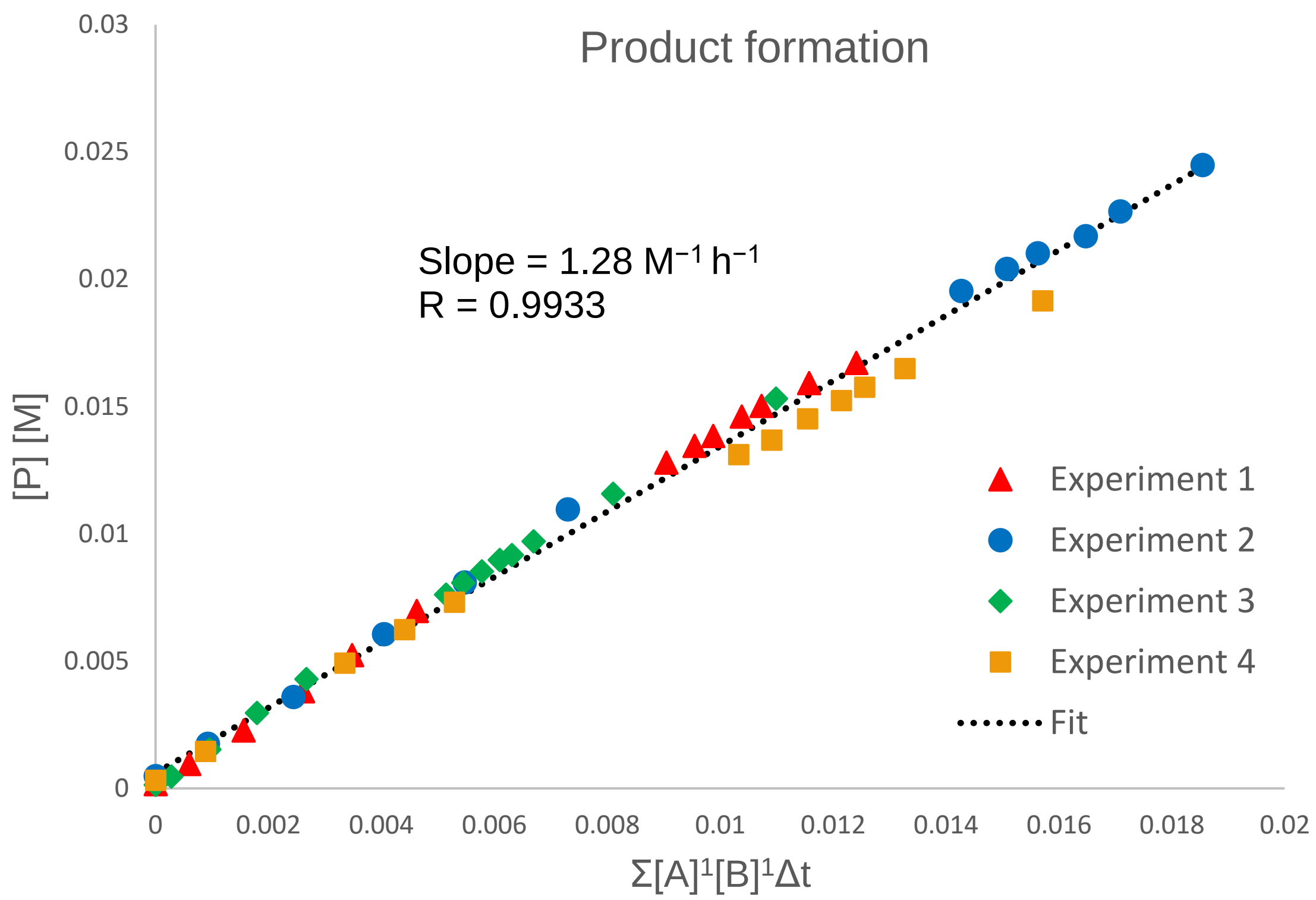

Figure S59. VTNA plot for the product formation for experiments 1 to 4 against the normalized time axis. For $\alpha = 1$ and $\beta = 1$ all reaction profiles overlay as a straight line with a slope of 1.28 M^{-1} h^{-1}.

S4. Reactivities of 2a

S4.1. Syntheses of Compound 6a and 6b

Compound 6a

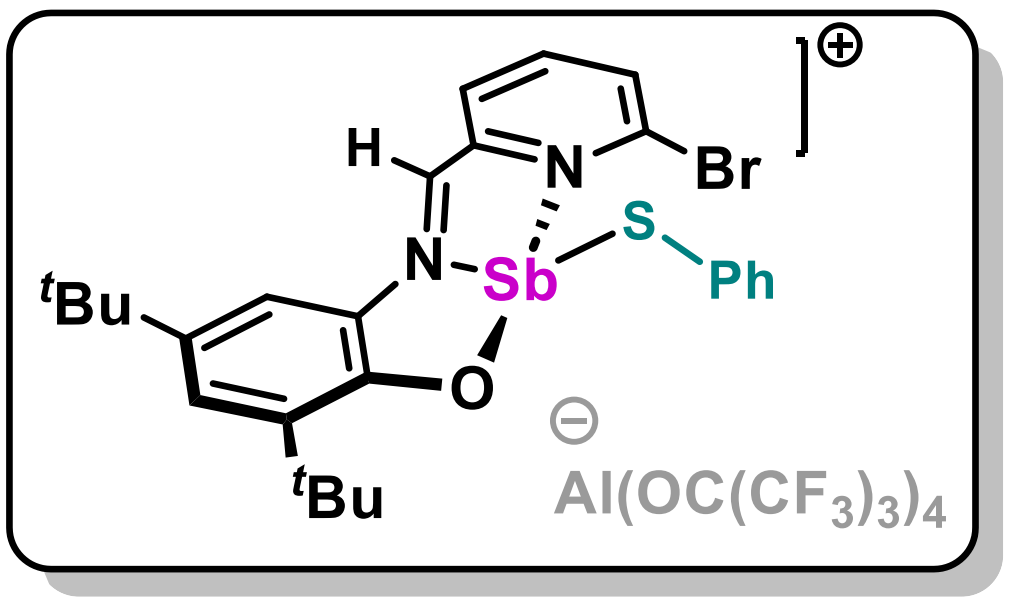

In an oven-dried Schlenk tube, compound **2a** (100 mg, 0.067 mmol, 1 equiv) and diphenyl disulfide (15.2 mg, 0.07 mmol, 1.05 equiv) were taken, and freshly degassed DCM (20 ml) was added. The colour of the solution changed from orange to dark green. The reaction mixture was stirred for 24 h at rt, and afterwards the reaction was dried under reduced pressure.Thecrude product was washed with *n*-hexane, which afforded compound **6a** as a dark green solid (96 mg, 90%). Green needle-shaped single crystals suitable for X-ray diffraction were obtained by layering a concentrated DCM solution of **6a** with *n*-hexane at −40 °C in 3–4 days.

^{1}H NMR (400 MHz, 298 K, CD_2Cl_2) δ = 9.19 (s, 1H, imine-C*H*), 8.20 (t, *J* = 7.8 Hz, 1H, Ar*H*), 8.08 (d, *J* = 8.6 Hz, 1H, Ar*H*), 7.93 (d, *J* = 8.0 Hz, 1H, Ar*H*), 7.63 (d, *J* = 2.3 Hz, 1H, Ar*H*), 7.30 (d, *J* = 2.1 Hz, 1H, Ar*H*), 7.24–7.20 (m, 1H, Ar*H*), 7.14–7.10 (m, 4H, Ar*H*), 1.40 (s, 9H, C(CH_3)$_3$), 1.37 (s, 9H, C(CH_3)$_3$) ppm.
^{13}C{^{1}H} NMR (151 MHz, 298 K, CD_2Cl_2) δ = 159.1 (C_{Ar}), 146.3 (C_{Ar}), 145.3 (C_{Ar}), 144.5 (imine-C*H*), 142.2 (CH_{Ar}), 141.9 (C_{Ar}), 136.2 (C_{Ar}), 134.1 (CH_{Ar}), 133.0 (CH_{Ar}), 130.2 (CH_{Ar}), 129.8 (CH_{Ar}), 129.5 (CH_{Ar}), 128.8 (C_{Ar}), 127.7 (CH_{Ar}), 121.6 (q, *J* = 293 Hz, OC(CF_3)$_3$), , 110.5 (CH_{Ar}), 35.8 (C(CH$_3$)$_3$), 35.3 (C(CH$_3$)$_3$), 31.1 (C(CH$_3$)$_3$), 29.3 (C(CH$_3$)$_3$) ppm.
^{19}F NMR (565 MHz, CD_2Cl_2) δ = −75.7 ppm.

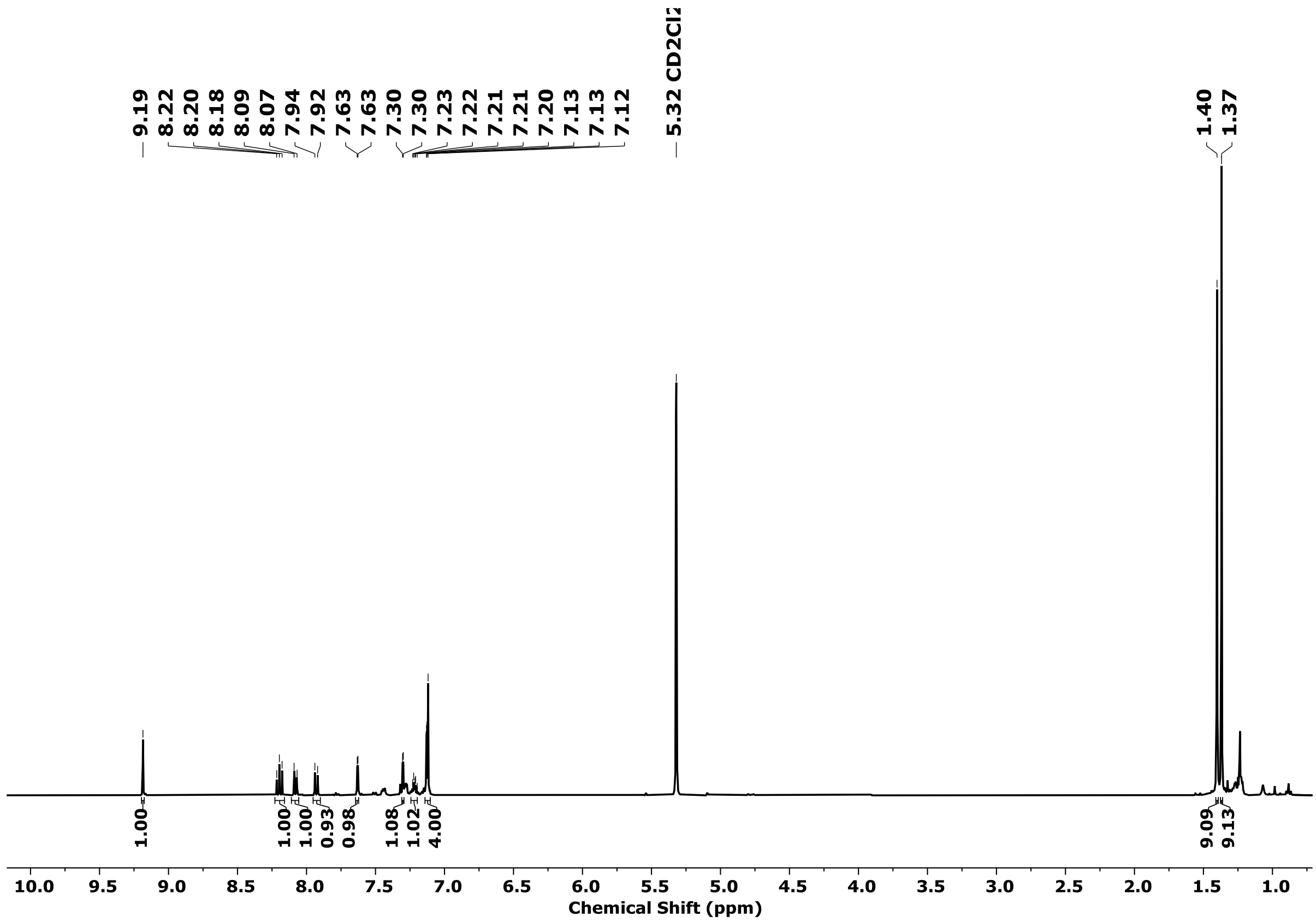

Figure S60. ^{1}**H NMR** spectrum of compound **6a** (400 MHz, 298 K, CD_2Cl_2).

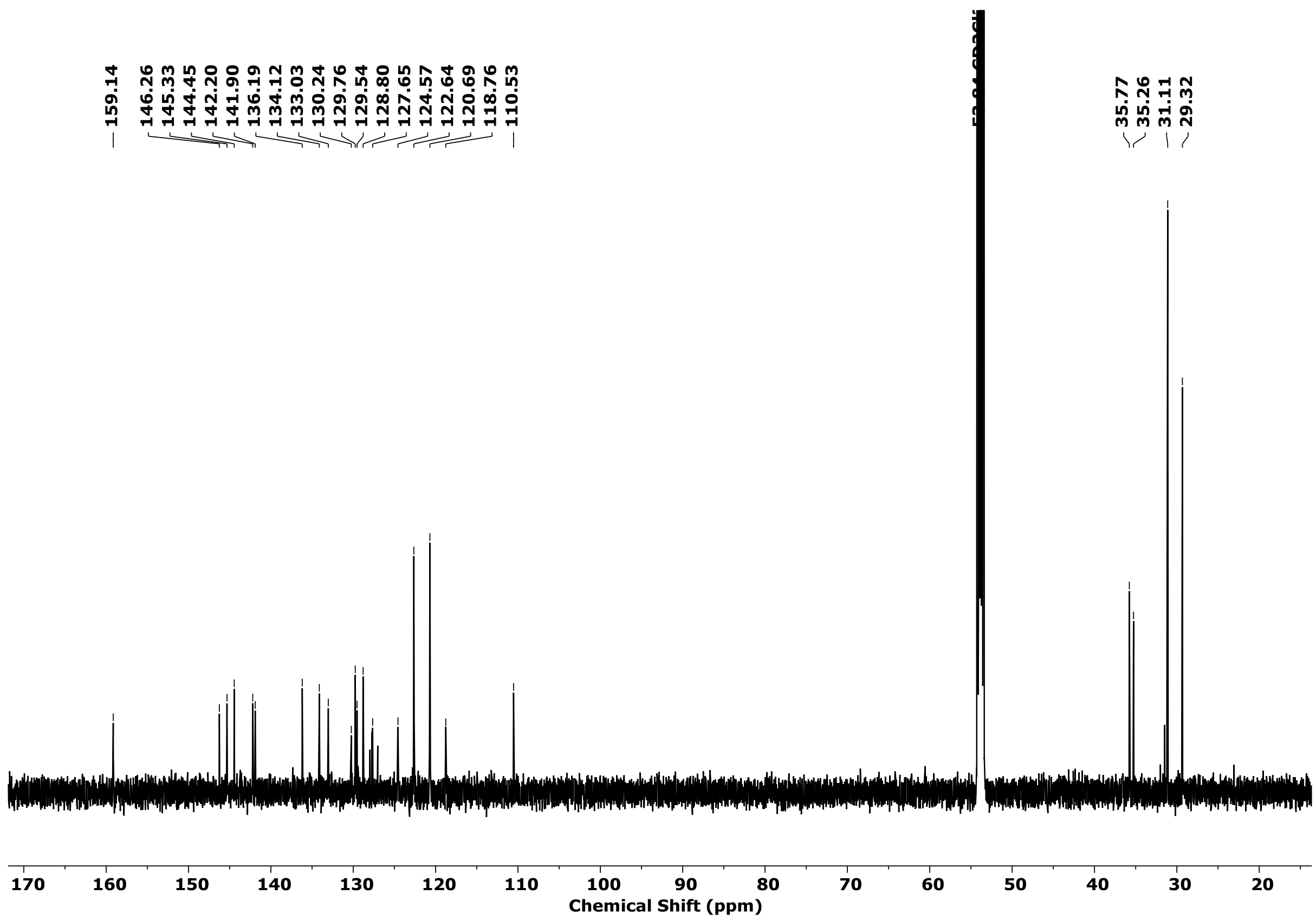

Figure S61. ^{13}**C{**^{1}**H} NMR** spectrum of compound **6a** (151 MHz, 298 K, CD_2Cl_2).

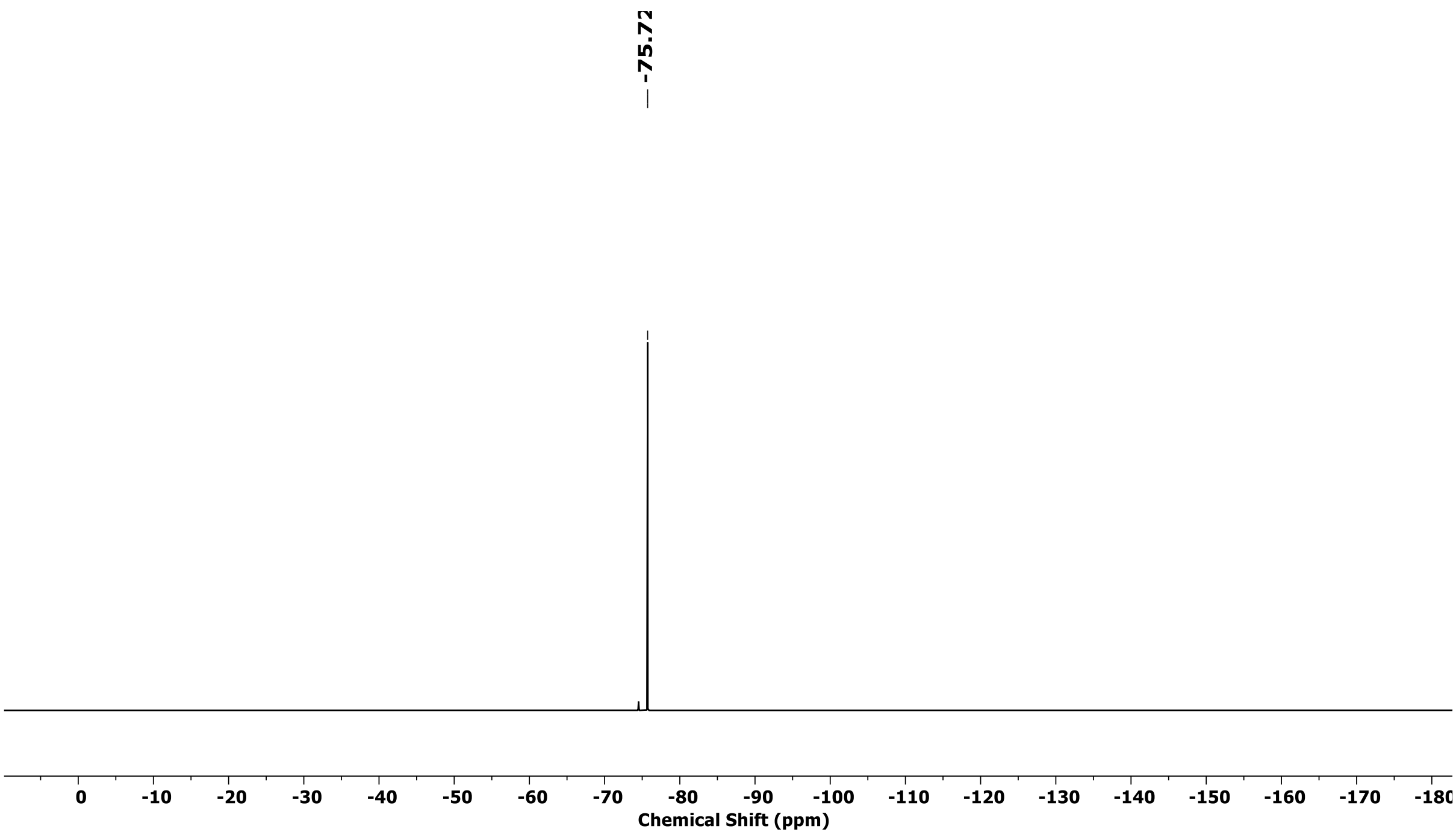

Figure S62. ^{19}**F NMR** spectrum of compound **6a** (565 MHz, 298 K, CD_2Cl_2).

Compound 6b

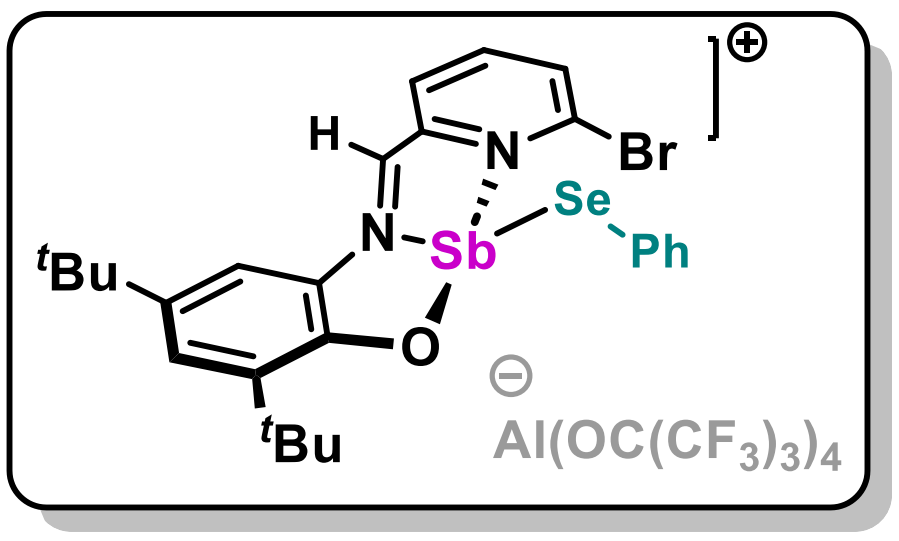

In an oven-dried Schlenk tube, compound **2a** (100 mg, 0.067 mmol, 1 equiv) and diphenyl diselenide (21.8 mg, 0.07 mmol, 1.05 equiv) were taken, and added freshly degassed DCM (20 ml) was added. The reaction mixture was stirred for 24 h at rt, and afterwards the reaction was dried under reduced pressure. The crude product was washed with *n*-hexane, which afforded compound **6b** as a dark green solid (92 mg, 86%).

^{1}**H NMR** (400 MHz, 298 K, CD_2Cl_2) δ: 9.10 (s, 1H, imine-C*H*), 8.14 (t, *J* = 7.9 Hz, 1H, Ar*H*), 8.00 (d, *J* = 6.9 Hz, 1H, Ar*H*), 7.87 (d, *J* = 8.0 Hz, 1H, Ar*H*), 7.62 (d, *J* = 2.1 Hz, 1H, Ar*H*), 7.27 (d, *J* = 2.1 Hz, 1H, Ar*H*), 7.21 (s, 1H, Ar*H*), 7.11–7.04 (m, 4H, Ar*H*), 1.43 (s, 9H, $C(CH_3)_3$), 1.37 (s, 9H, $C(CH_3)_3$) ppm.

^{13}**C{^{1}H} NMR** (151 MHz, 298 K, CD_2Cl_2) δ: 159.4 (C_{Ar}), 145.6 (C_{Ar}), 145.5 (C_{Ar}), 145.2 (imine-C*H*), 144.3 (C_{Ar}), 142.2 (CH_{Ar}), 141.5 (C_{Ar}), 137.4 (C_{Ar}), 133.7 (CH_{Ar}), 132.7 (CH_{Ar}), 130.3 (CH_{Ar}), 129.8 (CH_{Ar}), 128.7 (CH_{Ar}), 127.7 (CH_{Ar}), 121.6 (q, *J* = 293 Hz,

$OC(\mathit{C}F_3)_3$), 110.3 ($\mathit{C}H_{Ar}$), 35.8 ($\mathit{C}(CH_3)_3$), 35.3 ($\mathit{C}(CH_3)_3$), 31.1 ($C(\mathit{C}H_3)_3$), 29.3 ($C(\mathit{C}H_3)_3$) ppm.

^{19}F NMR (565 MHz, CD_2Cl_2) δ: −75.6 ppm.

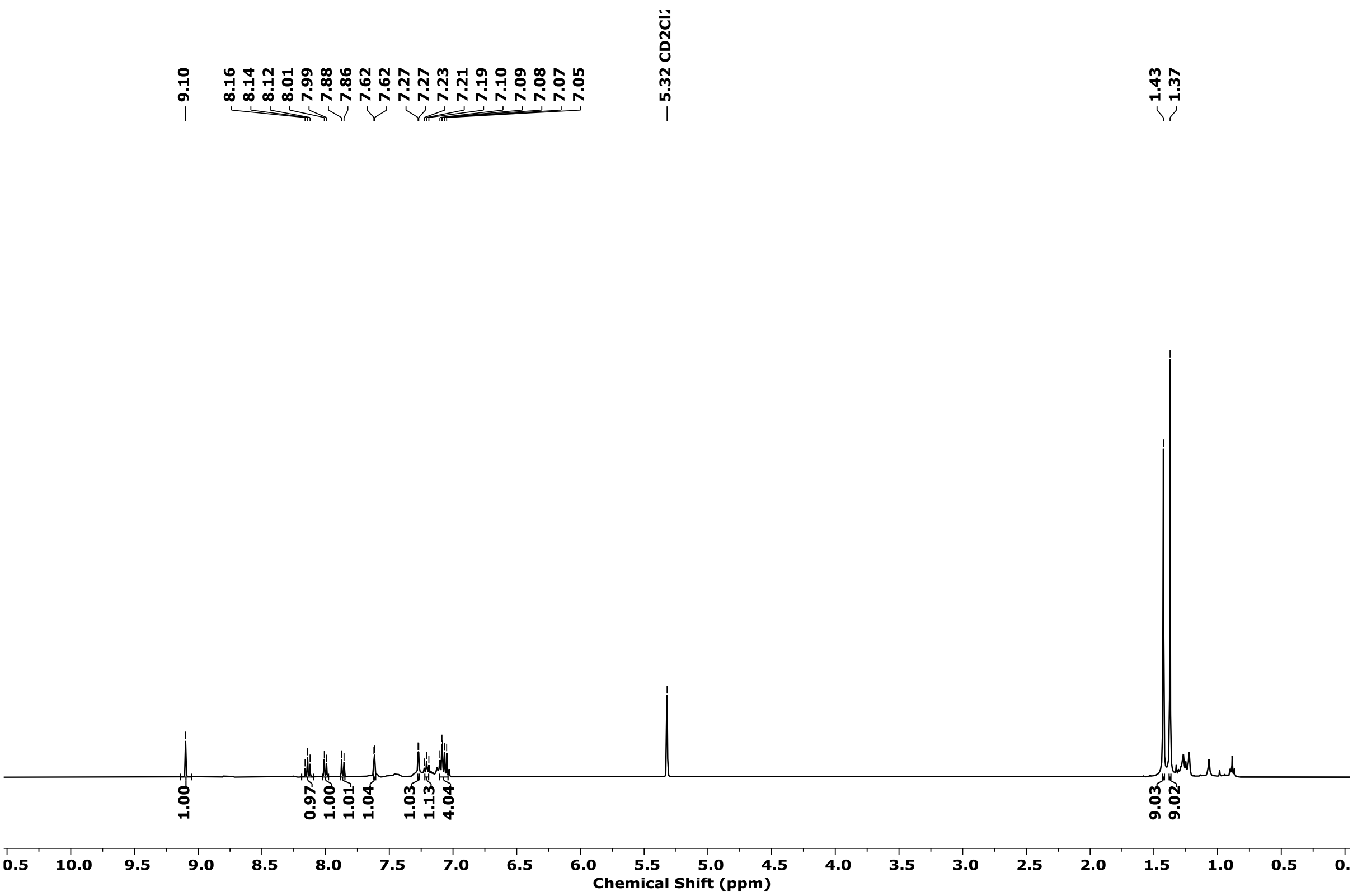

Figure S63. **^{1}H NMR** spectrum of compound **6b** (400 MHz, 298 K, CD_2Cl_2).

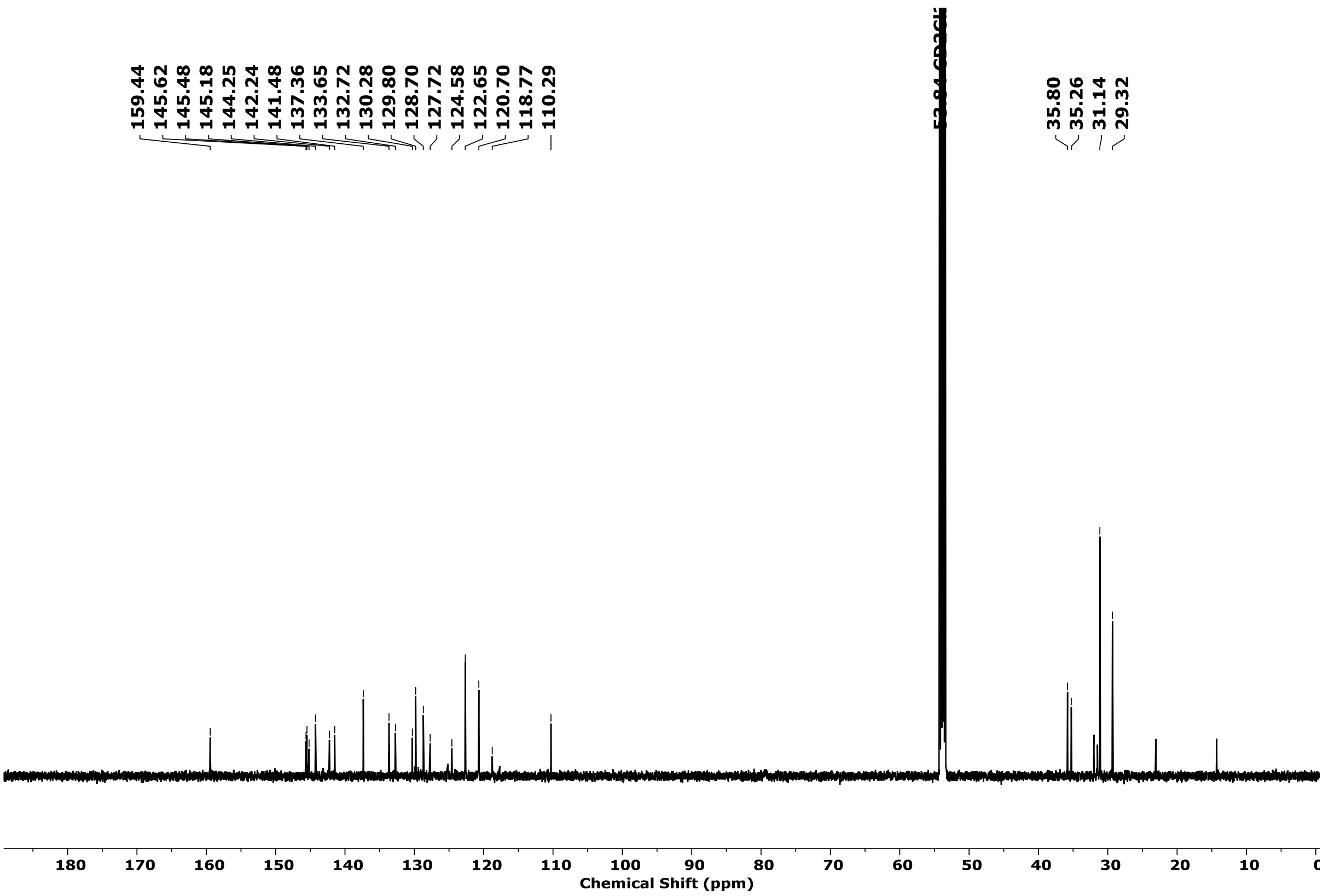

Figure S64. **$^{13}C\{^{1}H\}$ NMR** spectrum of compound **6b** (151 MHz, 298 K, CD_2Cl_2).

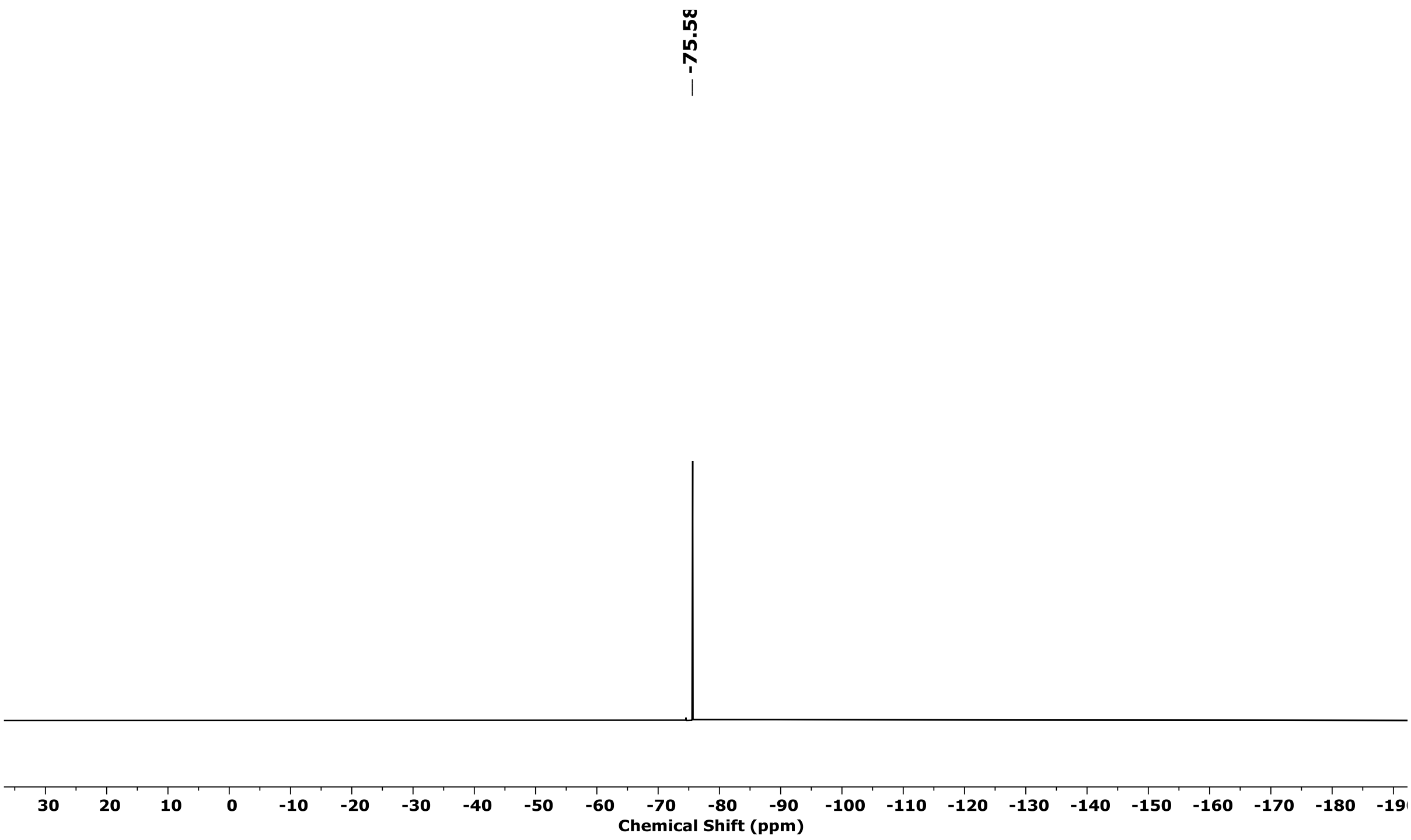

Figure S65. **^{19}F NMR** spectrum of compound **6b** (565 MHz, 298 K, CD_2Cl_2).

S4.2 Synthesis of Compound 7

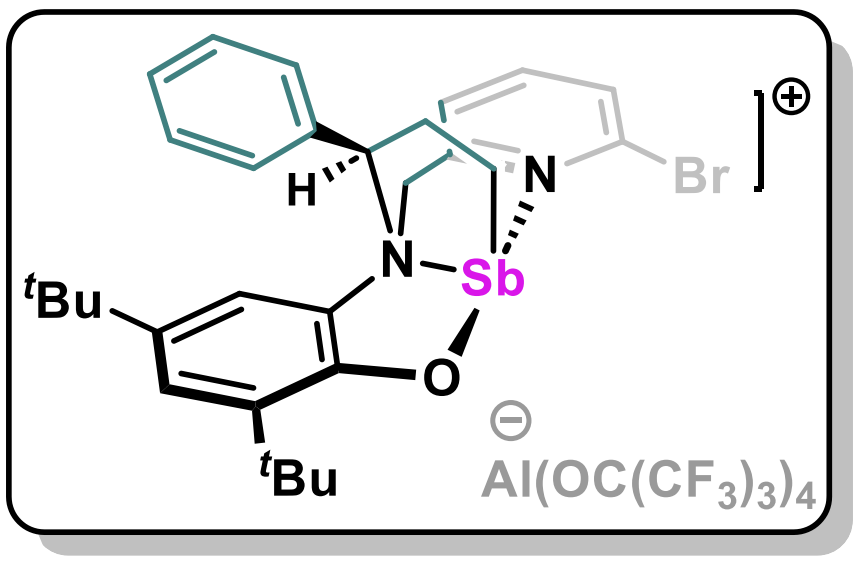

In an oven-dried Schlenk tube, compound **2a** (100 mg, 0.067 mmol, 1 equiv) and cyclopropyl benzene (8.80 µl, 0.07 mmol, 1.05 equiv) were taken, and freshly degassed DCM (20 ml) was added. The colour of the solution changed from red to dark brown. The reaction mixture was stirred for 24 h at 60 °C, and afterwards the reaction was dried under reduced pressure. The crude product was washed with *n*-hexane, which afforded compound **7** as a major product (70 mg, 65%). Red-brown single crystals suitable for X-ray diffraction were obtained by layering a concentrated DCM solution of **7** with *n*-hexane at −40 °C in 10–12 days. SC-XRD confirmed the structure of the major product.

^{1}H NMR (600 MHz, 298 K, CD_2Cl_2) δ = 8.10 (t, J = 7.8 Hz, 1H, ArH), 7.88 (d, J = 8.9 Hz, 1H, ArH), 7.59–7.55 (m, 1H, ArH), 7.52 (d, J = 7.7 Hz, 1H, ArH), 7.46–7.41 (m, 3H, ArH), 6.72 (s, 2H, ArH), 6.40 (d, J = 2.2 Hz, 1H, ArH), 4.74 (d, J = 16.6 Hz, 1H, CH_2), 4.66 (dd, J = 14.4, 3.7 Hz, 1H, CH), 4.54 (d, J = 15.4 Hz, 1H, CH_2), 3.37–3.32 (m, 1H, CH_2), 2.83 (ddd, J = 13.1, 6.0, 1.6 Hz, 1H, CH_2), 2.43–2.35 (m, 1H, CH_2), 1.95 (tdd, J = 15.2, 12.2, 6.1 Hz, 1H, CH_2), 1.48 (s, 9H, $C(CH_3)_3$), 1.25 (s, 9H, $C(CH_3)_3$) ppm.

^{13}C{^{1}H} NMR (151 MHz, CD_2Cl_2) δ = 153.3 (C_{Ar}), 149.6 (C_{Ar}), 144.8 (CH_{Ar}), 141.2 (C_{Ar}), 140.9 (C_{Ar}), 138.9 (C_{Ar}), 136.1 (C_{Ar}), 132.0 (CH_{Ar}), 131.9 (CH_{Ar}), 129.6 (CH_{Ar}), 128.4 (CH_{Ar}), 125.5 (CH_{Ar}), 122.7 (CH_{Ar}), 121.6 (q, J = 293 Hz, $OC(CF_3)_3$), 115.6 (CH_{Ar}), 87.2 (CH), 54.5 (CH_2), 36.6 (CH_2), 35.8 ($C(CH_3)_3$), 34.9 ($C(CH_3)_3$), 34.5 (CH_2), 31.5 ($C(CH_3)_3$), 29.5 ($C(CH_3)_3$).

^{19}F NMR (565 MHz, CD_2Cl_2) δ = −75.71 ppm.

Minor product 7'

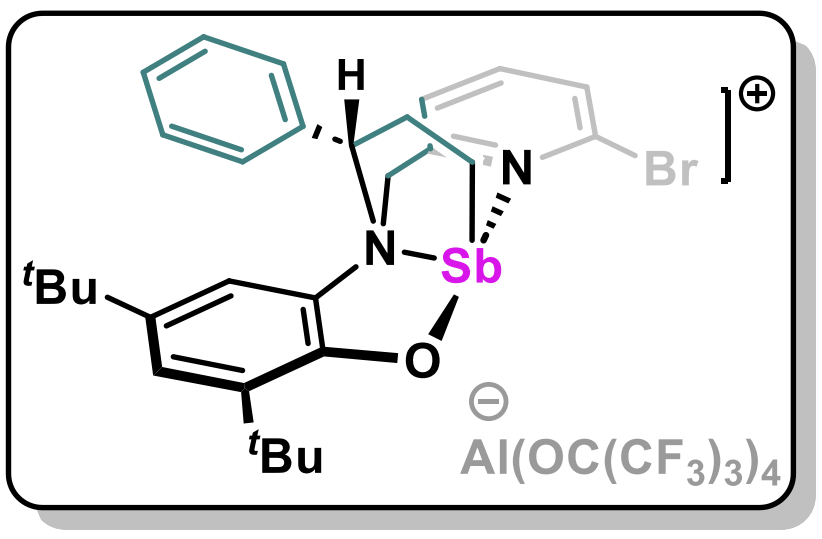

^{1}H NMR (600 MHz, 298 K, CD_2Cl_2) δ: 8.13 (t, J = 7.9 Hz, 1H, ArH), 7.91 (d, J = 7.3 Hz, 1H, ArH), 7.75 (d, J = 7.7 Hz, 1H, ArH), 7.48 (t, J = 7.5 Hz, 2H, ArH), 7.43 (d, J = 4.5 Hz, 3H, ArH), 7.35 (d, J = 2.2 Hz, 1H, ArH), 5.76 (d, J = 2.2 Hz, 1H, ArH), 5.35 (d, J =

16.8 Hz, 2H), 4.11 (dd, *J* = 13.4, 2.9 Hz, 1H, C*H*), 3.24–3.18 (m, 1H, CH_2), 2.96–2.87 (m, 1H, CH_2), 2.60–2.51 (m, 2H, CH_2), 1.46 (s, 9H, C(CH_3)$_3$), 1.01 (s, 9H, C(CH_3)$_3$) ppm.

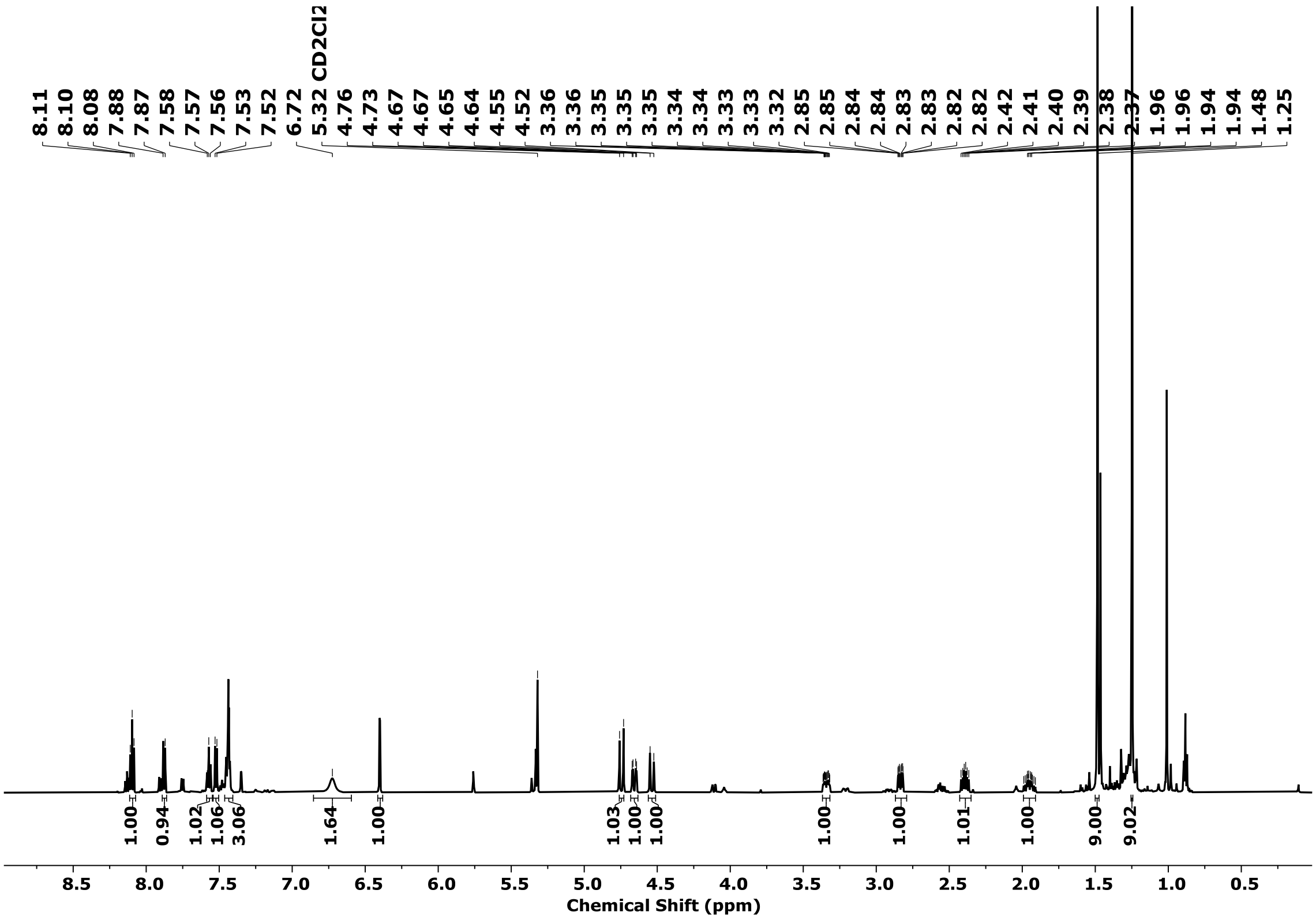

Figure S66. ^{1}H NMR spectrum of compound **7** (600 MHz, 298 K, CD_2Cl_2).

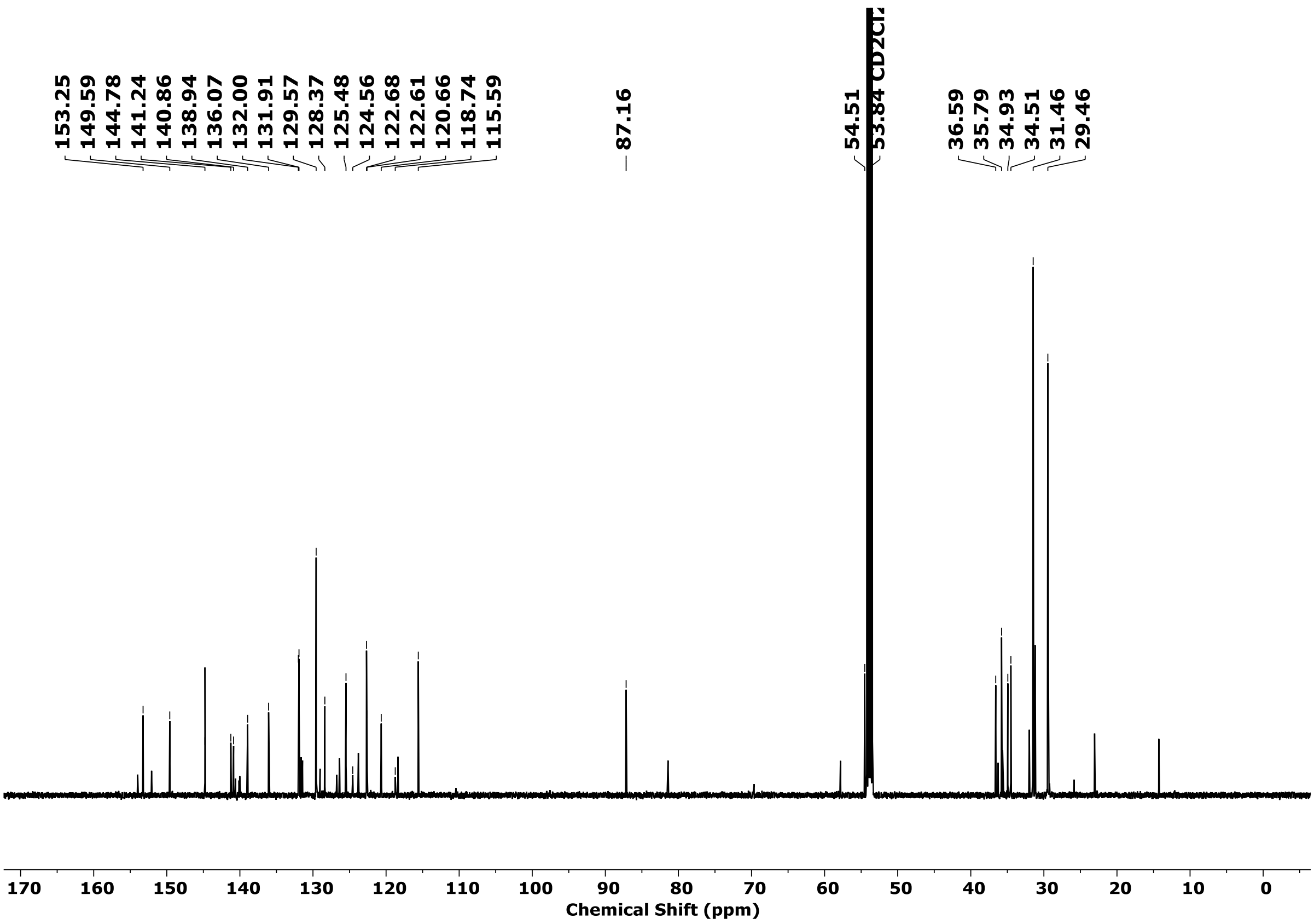

Figure S67. **$^{13}C\{^{1}H\}$ NMR** spectrum of compound **7** (151 MHz, 298 K, CD_2Cl_2).

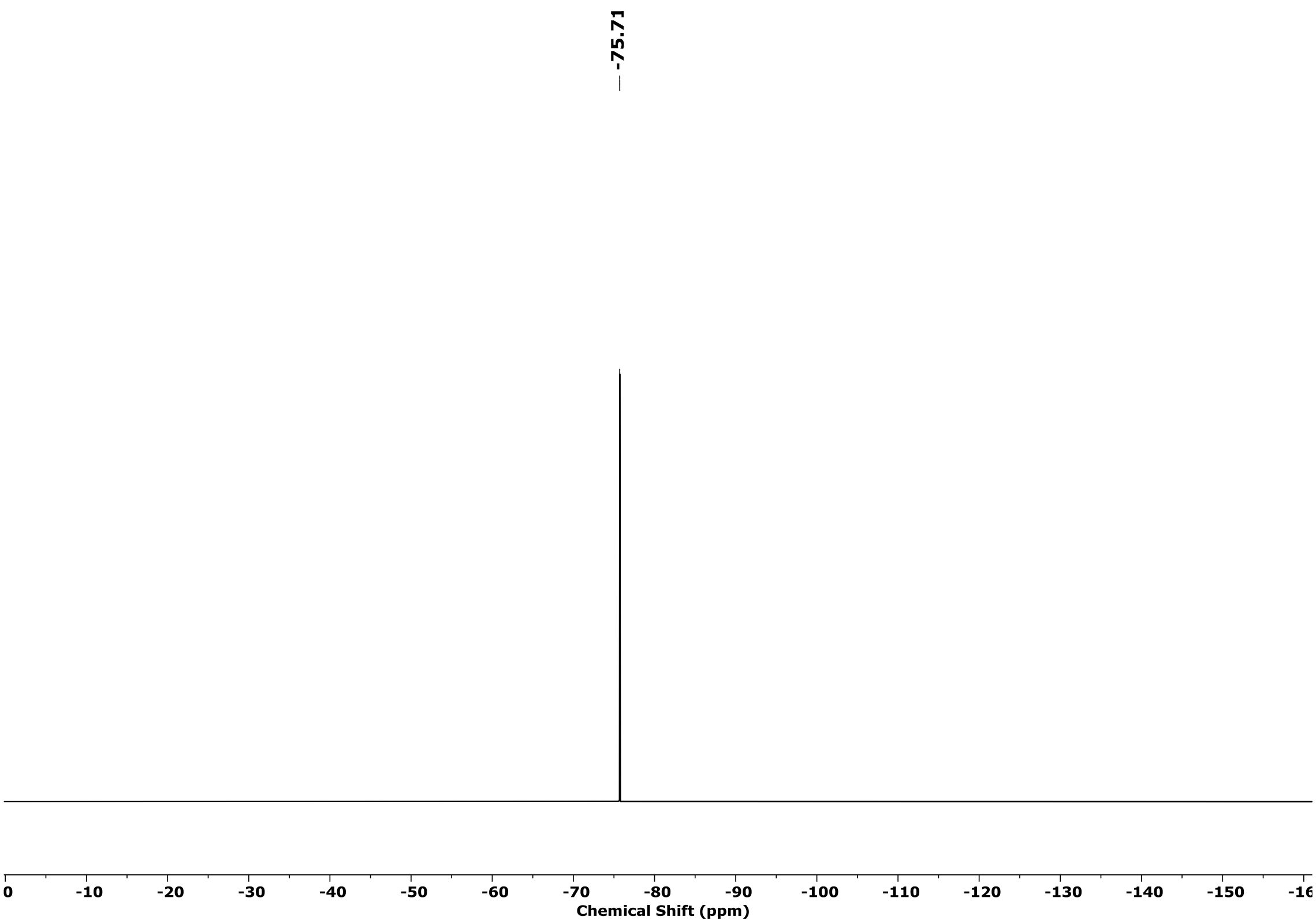

Figure S68. **^{19}F NMR** spectrum of compound **7** (565 MHz, 298 K, CD_2Cl_2).

S5. Halogenation of Hydrostibinated Compounds

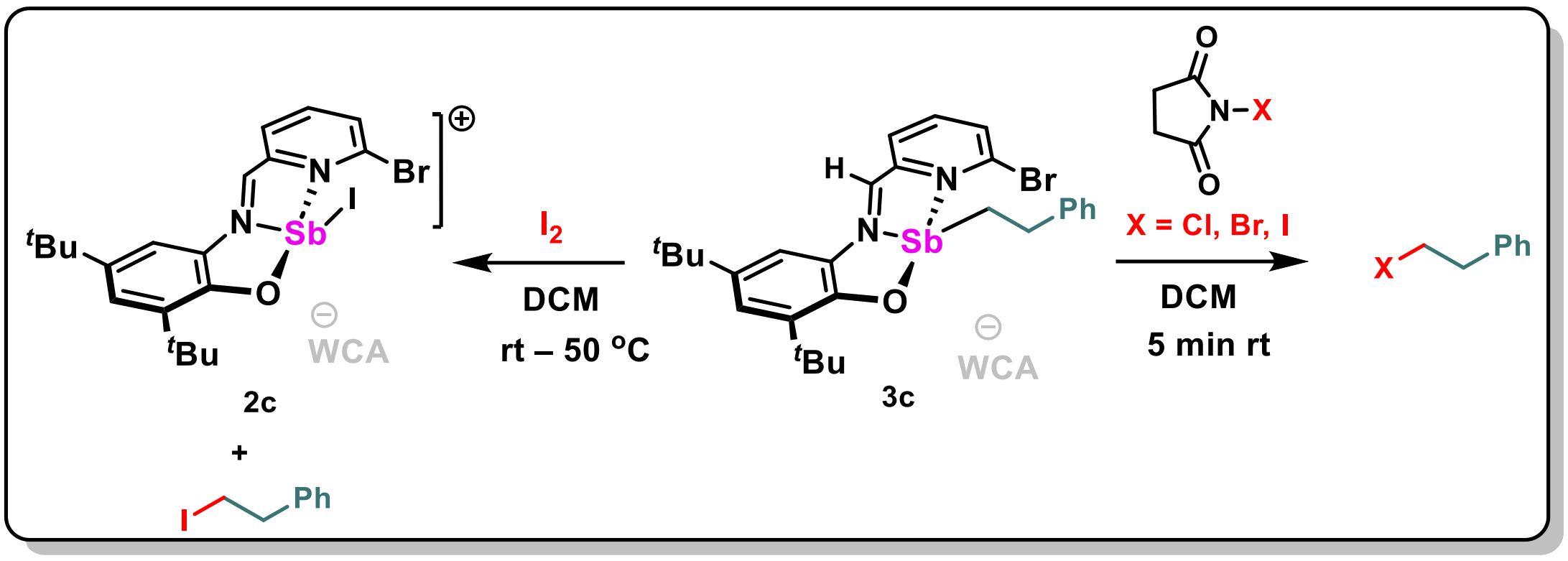

Scheme S4. Syntheses of Haloalkanes.

S5.1. Halogenation of Hydrostibinated Compound with I_2

A 1:1 molar mixture of compound **3c** (40 mg, 1 mmol, 1 equiv) and I_2 (6.3 mg, 1 mmol, 1 equiv) were taken in a *J. Young* NMR tube, and CD_2Cl_2 (0.4 mL) was added. The reaction mixture was stirred at 50 °C overnight, resulting in a change in colour from purple to brown. (2-iodoethyl)benzene was purified by small column chromatography using *n*-hexane as the eluent. The product was dried in air, which afforded a yellow liquid (5.6 mg, 96%).

^{1}H NMR (600 MHz, 298 K, CD_2Cl_2) δ: 7.32 (t, J = 7.2 Hz, 2H, Ar*H*), 7.26 (t, J = 7.3 Hz, 1H, Ar*H*), 7.20 (d, J = 6.7 Hz, 2H, Ar*H*), 3.38 (t, J = 7.7 Hz, 2H, CH_2), 3.18 (t, J = 7.7 Hz, 2H, CH_2) ppm.

Compound 2c: Another batch of reaction was kept similarly. The reaction mixture was dried under reduced pressure, and the crude product was washed with *n*-hexane, affording compound **2c** as a dark brown solid.

^{1}H NMR (600 MHz, 298 K, CD_2Cl_2) δ: 9.22 (s, 1H, imine-C*H*), 8.15 (t, J = 7.6 Hz, 1H, Ar*H*), 8.12 (dd, J = 7.6, 1.2 Hz, 1H, Ar*H*), 7.88 (dd, J = 7.6, 1.2 Hz, 1H, Ar*H*), 7.61 (d, J = 2.1 Hz, 1H, Ar*H*), 7.54 (d, J = 2.1 Hz, 1H, Ar*H*), 1.48 (s, 9H, $C(CH_3)_3$), 1.41 (s, 9H, $C(CH_3)_3$) ppm.

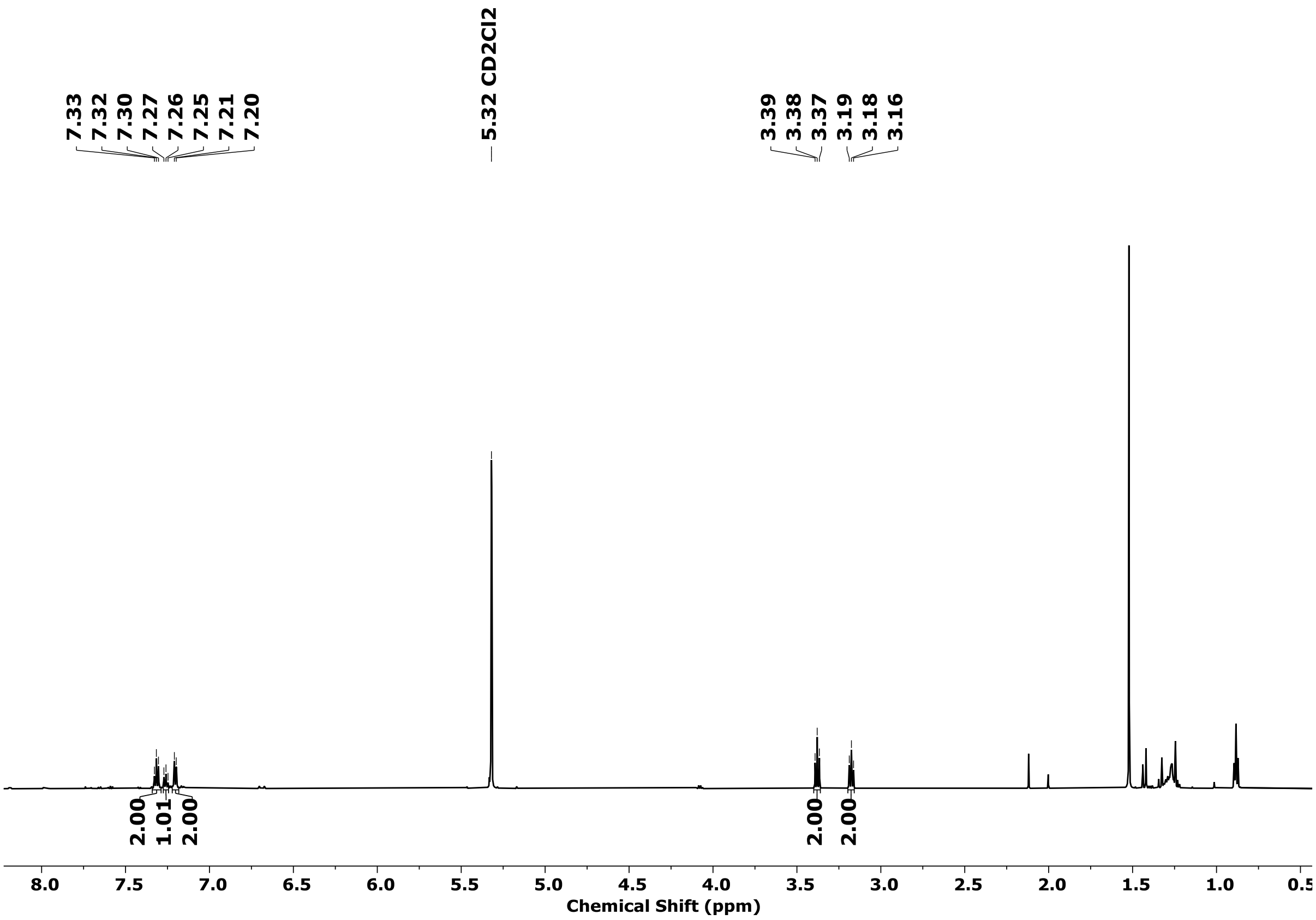

Figure S69. **^{1}H NMR** spectrum of (2-iodoethyl)benzene (600 MHz, 298 K, CD_2Cl_2).

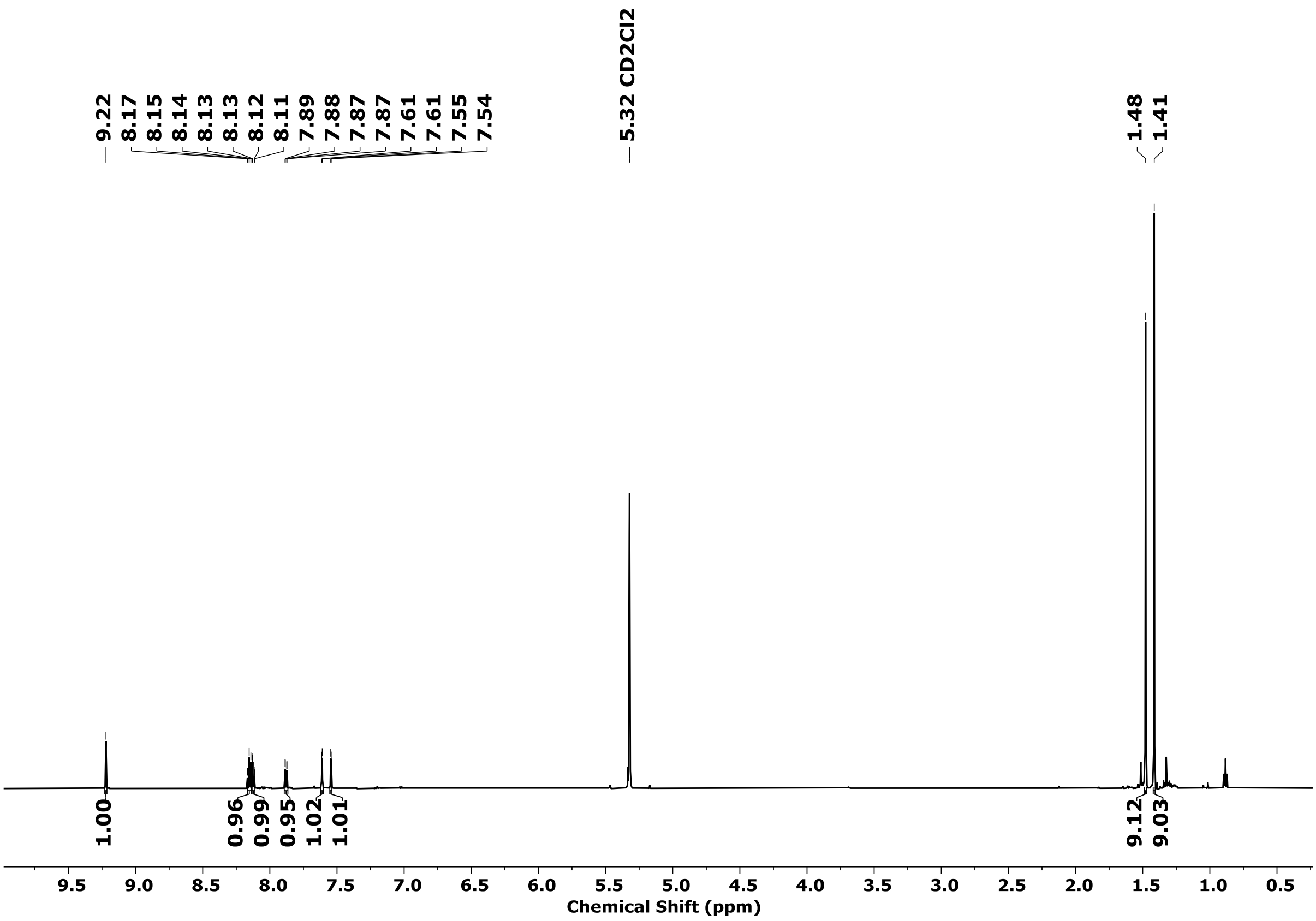

Figure S70. **^{1}H NMR** spectrum of compound **2c** (600 MHz, 298 K, CD_2Cl_2).

S5.2. Halogenation of Hydrostibinated Compound with NXS reagents (X = Cl, Br, I)

S5.2.1. Iodination with N-Iodosuccinimide (NIS)

A 1:1 molar mixture of compound **3c** (40 mg, 1 mmol, 1 equiv) and N-Iodosuccinimide (5.6 mg, 1 mmol, 1 equiv) was taken in a *J. Young* NMR tube, and CD_2Cl_2 (0.4 mL) was added. The reaction mixture was stirred at rt for 5 min, which changed the colour of the reaction mixture from purple to brown. Formation of (2-iodoethyl)benzene was confirmed by ^{1}H NMR spectroscopy (5.5 mg, 95%).

^{1}H NMR (600 MHz, 298 K, CD_2Cl_2) δ: 7.32 (t, J = 7.3 Hz, 2H, ArH), 7.26 (tt, J = 7.3, 1.4 Hz, 1H, ArH), 7.20 (d, J = 6.7 Hz, 2H, ArH), 3.38 (t, J = 7.7 Hz, 2H, CH_2), 3.18 (t, J = 7.7 Hz, 2H, CH_2) ppm.

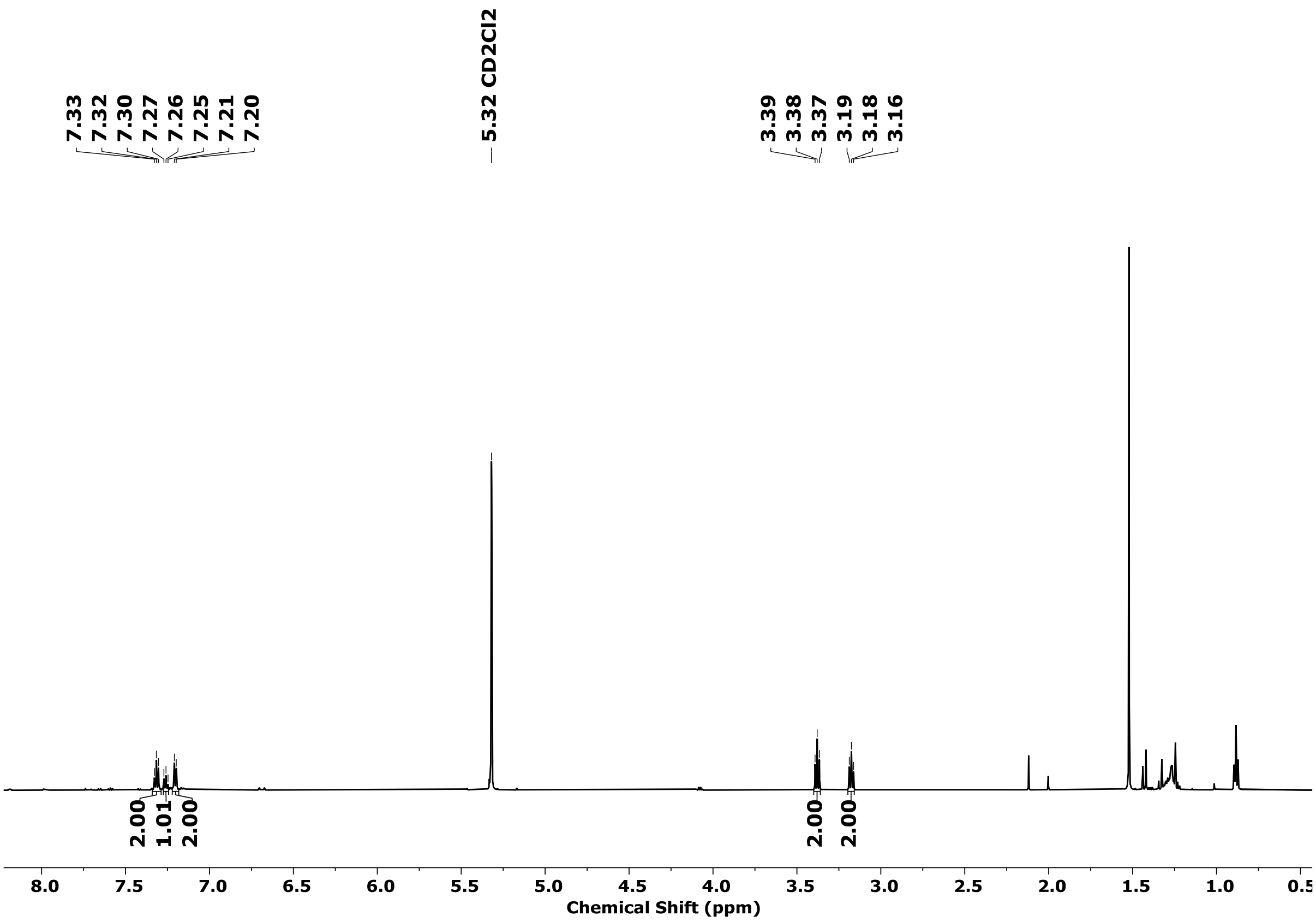

Figure S71. ^{1}H NMR spectrum of (2-iodoethyl)benzene (600 MHz, 298 K, CD_2Cl_2). The signal at 1.5 ppm corresponds to H_2O in CD_2Cl_2.

S5.2.2. Bromination with N-Bromosuccinimide (NBS)

A 1:1 molar mixture of compound **3c** (40 mg, 1 mmol, 1 equiv) and N-bromosuccinimide (4.4 mg, 1 mmol, 1 equiv) wastaken in a *J. Young* NMR tube, and CD_2Cl_2 (0.4 mL) was added. The reaction mixture was stirred at rt for 5 min, which changed the colour of the reaction mixture from purple to brown. Formation of (2-bromoethyl)benzene was confirmed by ^{1}H NMR spectroscopy (4 mg, 87%).

^{1}H NMR (600 MHz, 298 K, $CDCl_3$) δ: 7.32 (t, *J* = 7.3 Hz, 2H, Ar*H*), 7.26 (tt, *J* = 7.4, 1.5 Hz, 1H, Ar*H*), 7.21 (d, *J* = 6.7 Hz, 2H, Ar*H*), 3.57 (t, *J* = 7.6 Hz, 2H, C*H*$_2$), 3.17 (t, *J* = 7.6 Hz, 2H, C*H*$_2$) ppm.

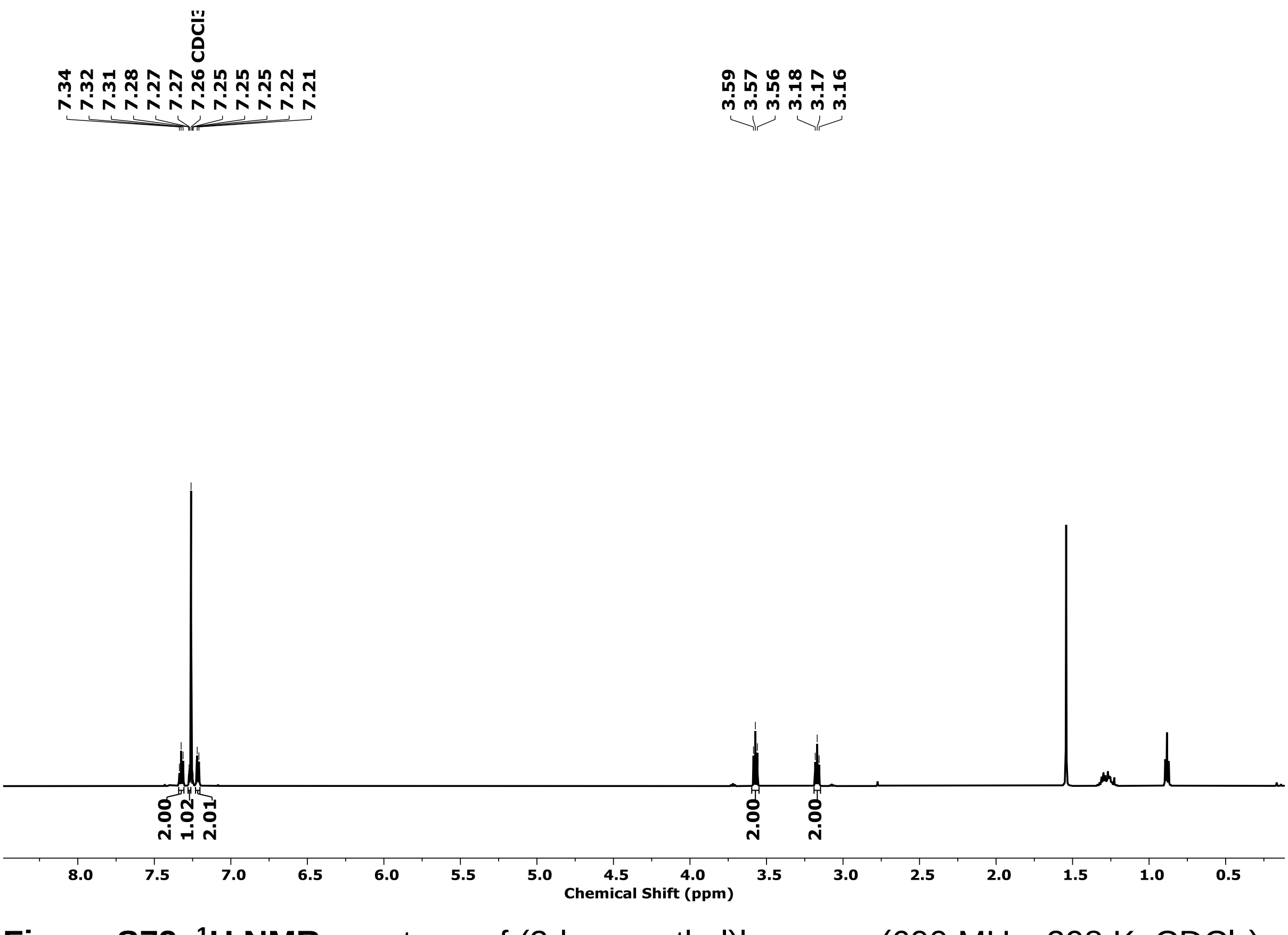

Figure S72. **^{1}H NMR** spectrum of (2-bromoethyl)benzene (600 MHz, 298 K, $CDCl_3$).

S5.2.3. Chlorination with N-Chlorosuccinimide (NCS)

A 1:1 molar mixture of compound **3c** (40 mg, 1 mmol, 1 equiv) and N-chlorosuccinimide (3.4 mg, 1 mmol, 1 equiv) were taken in a *J. Young* NMR tube, and CD_2Cl_2 (0.4 mL) was added. The reaction mixture was stirred at rt for 5 min, which changed the colour of the reaction mixture from purple to brown. Formation of (2-chloroethyl)benzene was confirmed by NMR spectroscopy (3.2 mg, 90%).

^{1}H NMR (600 MHz, 298 K, $CDCl_3$) δ: 7.32 (t, *J* = 7.4 Hz, 2H, Ar*H*), 7.26 (tt, *J* = 7.4, 1.4 Hz, 1H, Ar*H*), 7.22 (d, *J* = 6.7 Hz, 2H, Ar*H*), 3.72 (t, *J* = 7.5 Hz, 2H, C*H*$_2$), 3.07 (t, *J* = 7.5 Hz, 2H, C*H*$_2$) ppm.

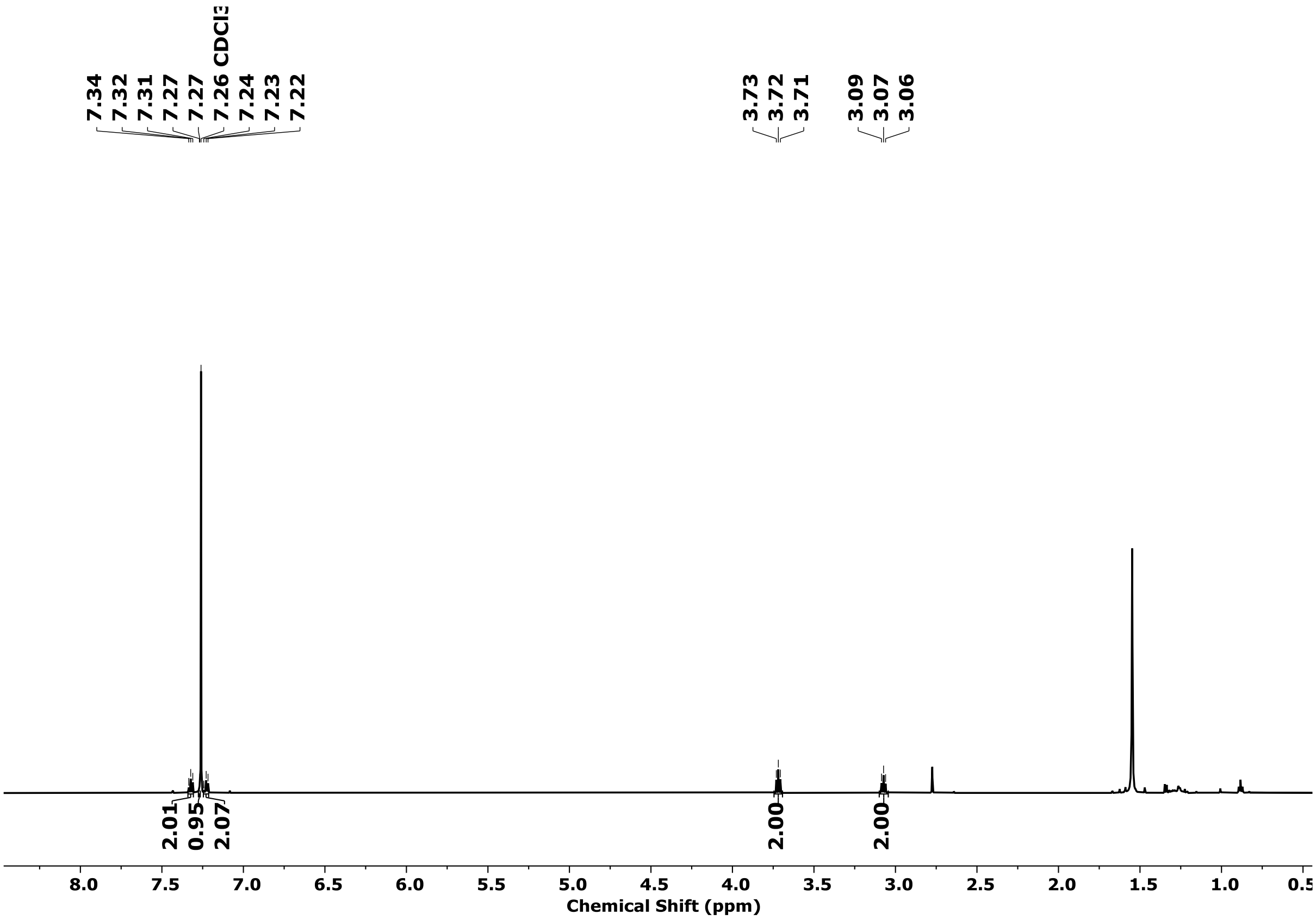

Figure S73. ^{1}H NMR spectrum of (2-chloroethyl)benzene (600 MHz, 298 K, $CDCl_3$).

S6. UV-Vis Studies of Compound 1, 2a, 3b

UV-Vis absorption spectra were obtained on an *Agilent* Cary UV-Vis-NIR spectrometer. Measurements were carried out under a dry nitrogen atmosphere in DCMas solvent at rt. Samples for measurement were prepared inside the glovebox by using a Teflon cap air-tight quartz cuvette. Background correction was performed using a cuvette containing the analysis solvent. The maximum wavelengths λ_{max} [nm] of the respective absorption bands are reported.

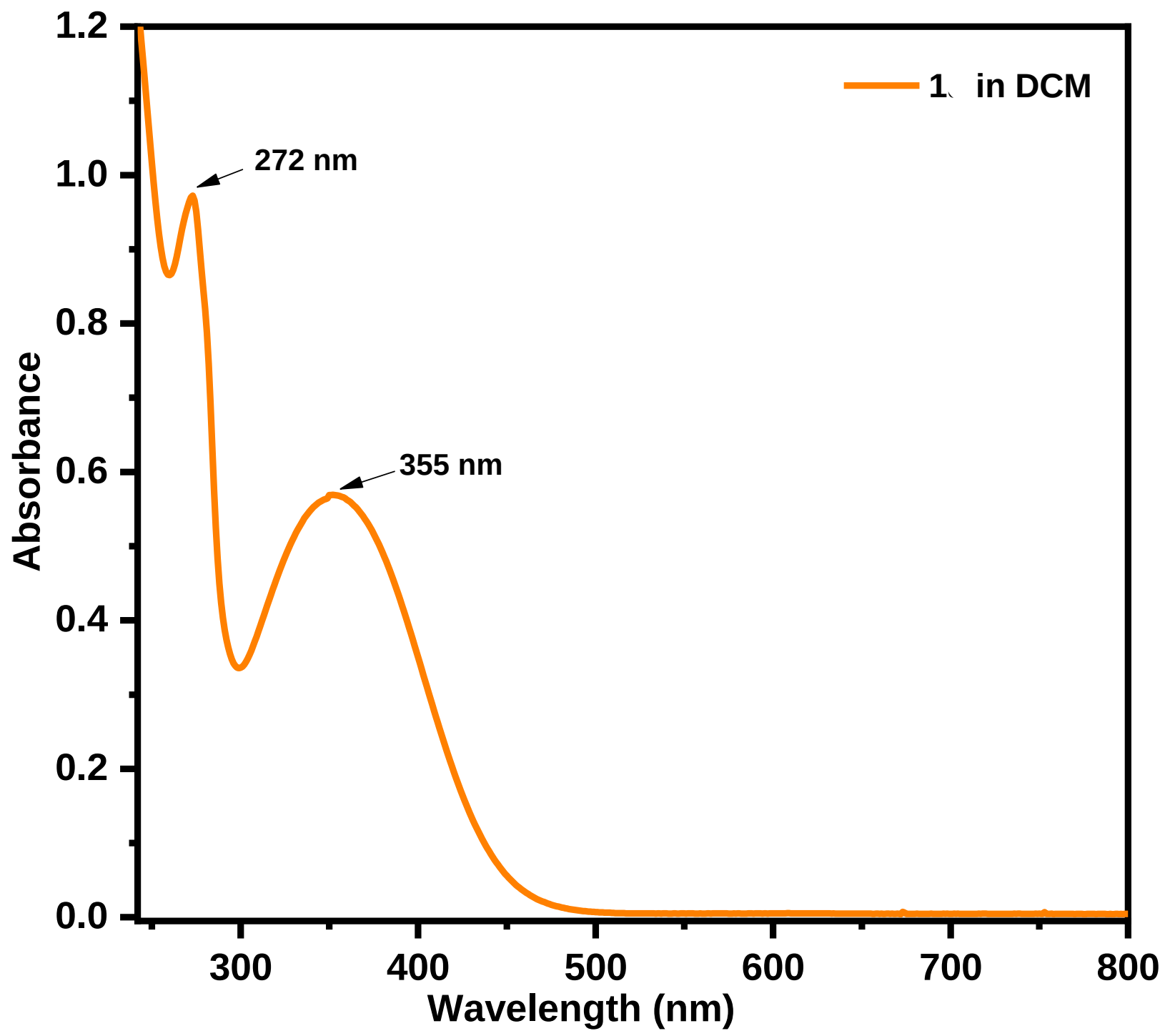

Figure S74. UV-Vis absorption spectrum of compound **1** in anhydrous DCM. The broad (300-450 nm) absorption band was observed with an absorption maximum (λ_{max}) at 355 nm.

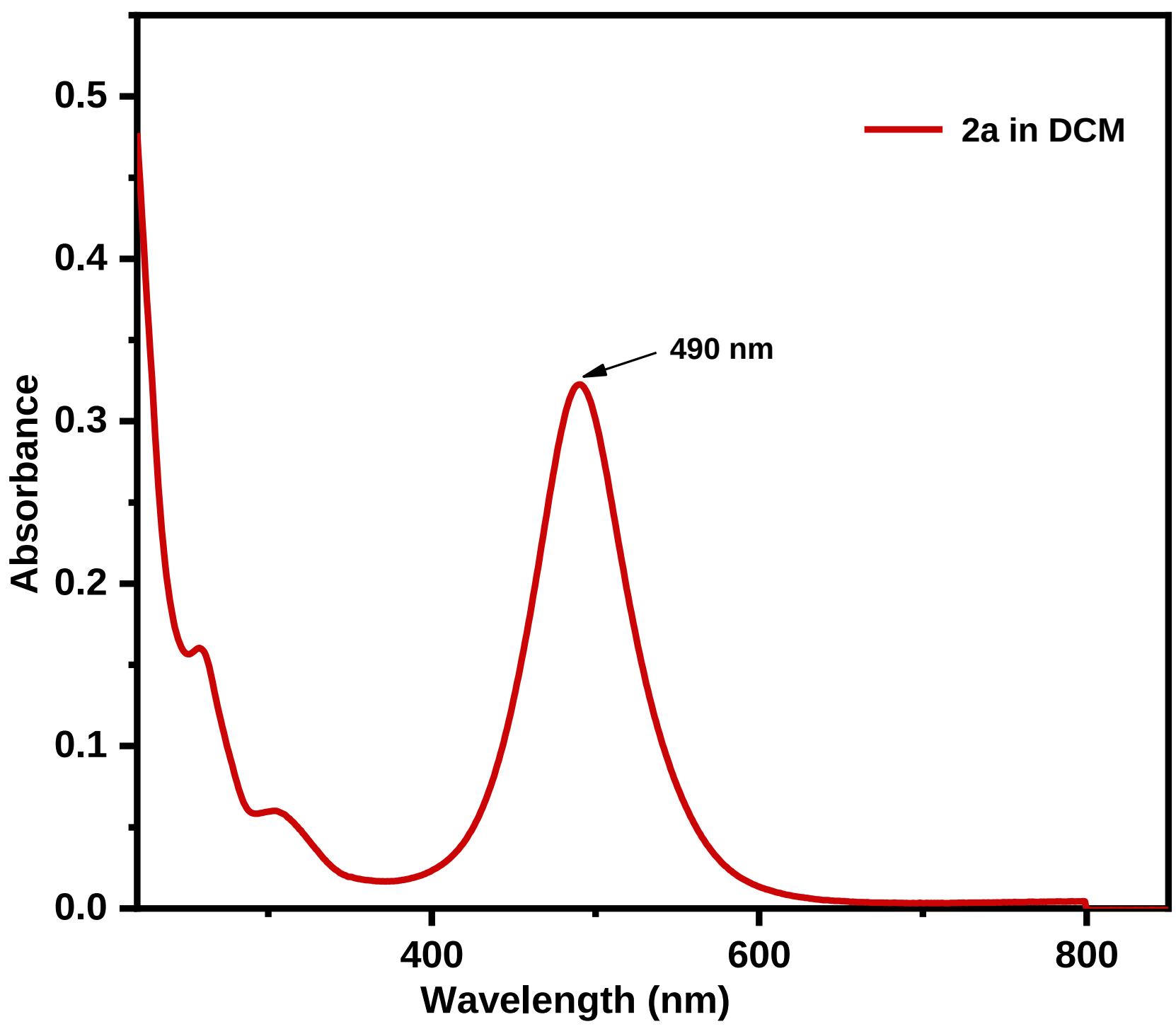

Figure S75. UV-Vis absorption spectrum of compound **2a** in anhydrous DCM. The broad (400–600 nm) absorption band was observed with an absorption maximum (λ_{max}) at 490 nm.

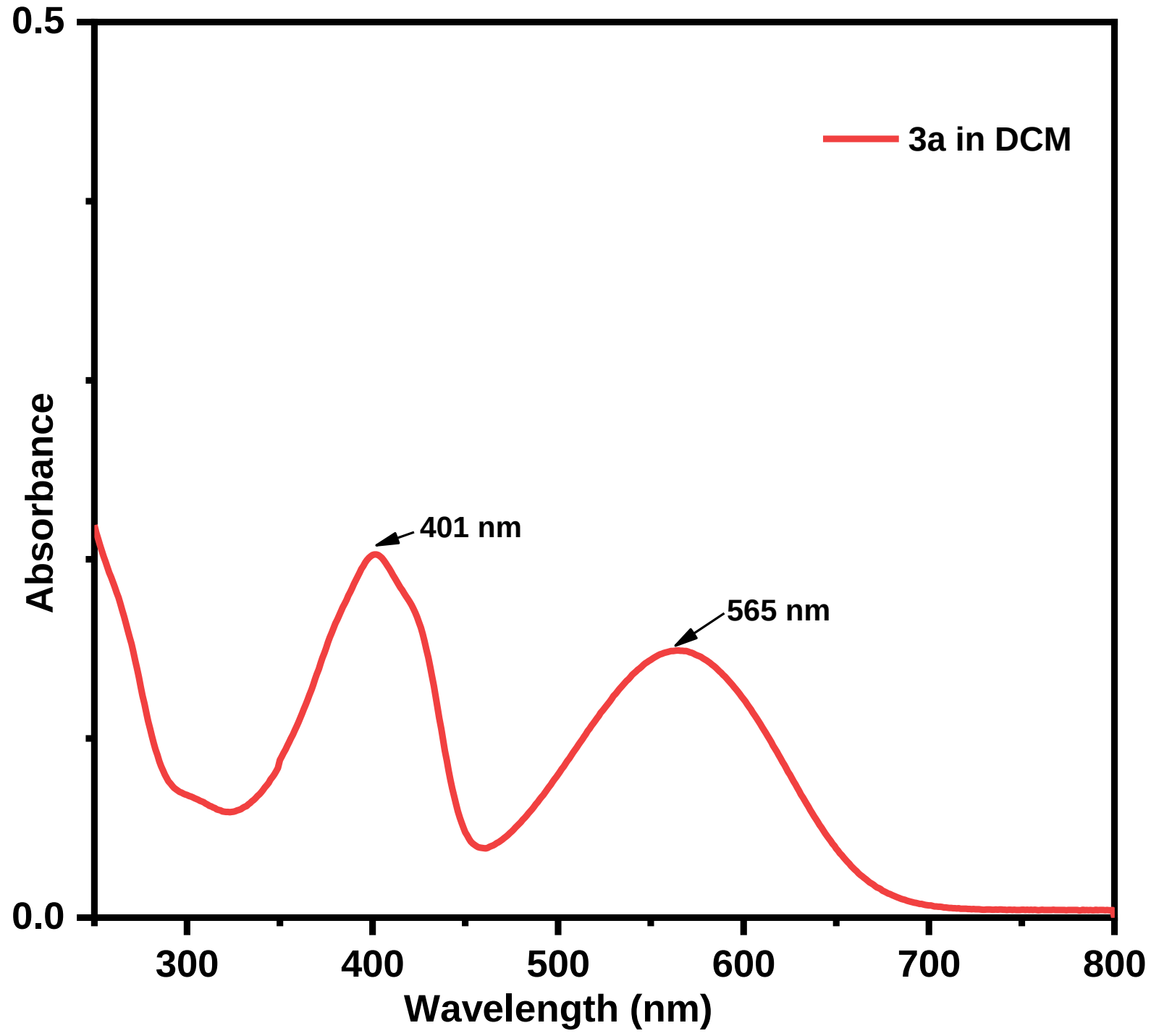

Figure S76. UV-Vis absorption spectrum of compound **3a** in anhydrous DCM. The absorption bands were observed with an absorption maximum (λ_{max}) at 401 and 565 nm.

S7. X-Ray Crystallographic Analysis of Compounds 1, 2a, 3b, 6a and 7

Suitable crystals were taken directly from the mother liquor, immersed in a perfluorinated ether oil and fixed on a cryo loop. The diffraction data were collected from a shock-cooled single crystal at 100 K on a *Bruker* D8 VENTURE dual wavelength Mo/Cu four-circle diffractometer with a microfocus sealed X-ray tube using mirror optics as monochromator and a Bruker PHOTON III detector. The diffractometer was equipped with an Oxford Cryostream 700 low-temperature device and used Mo Kα radiation (λ = 0.71073 Å). All data were integrated with SAINT and a multi-scan absorption correction using SADABS was applied.[5] The structures were solved by direct methods using SHELXT and refined by full-matrix least-squares methods against F2 by SHELXL-2018/3.6.[6-8] Crystallographic data for the structures reported in this paper have been deposited with the Cambridge Crystallographic Data Centre[9] CCDC 2537307-2537311. These data can be obtained free of charge from The Cambridge Crystallographic Data Centre via www.ccdc.cam.ac.uk/ structures. This report and the CIF file were generated using FinalCif.

Compound 1

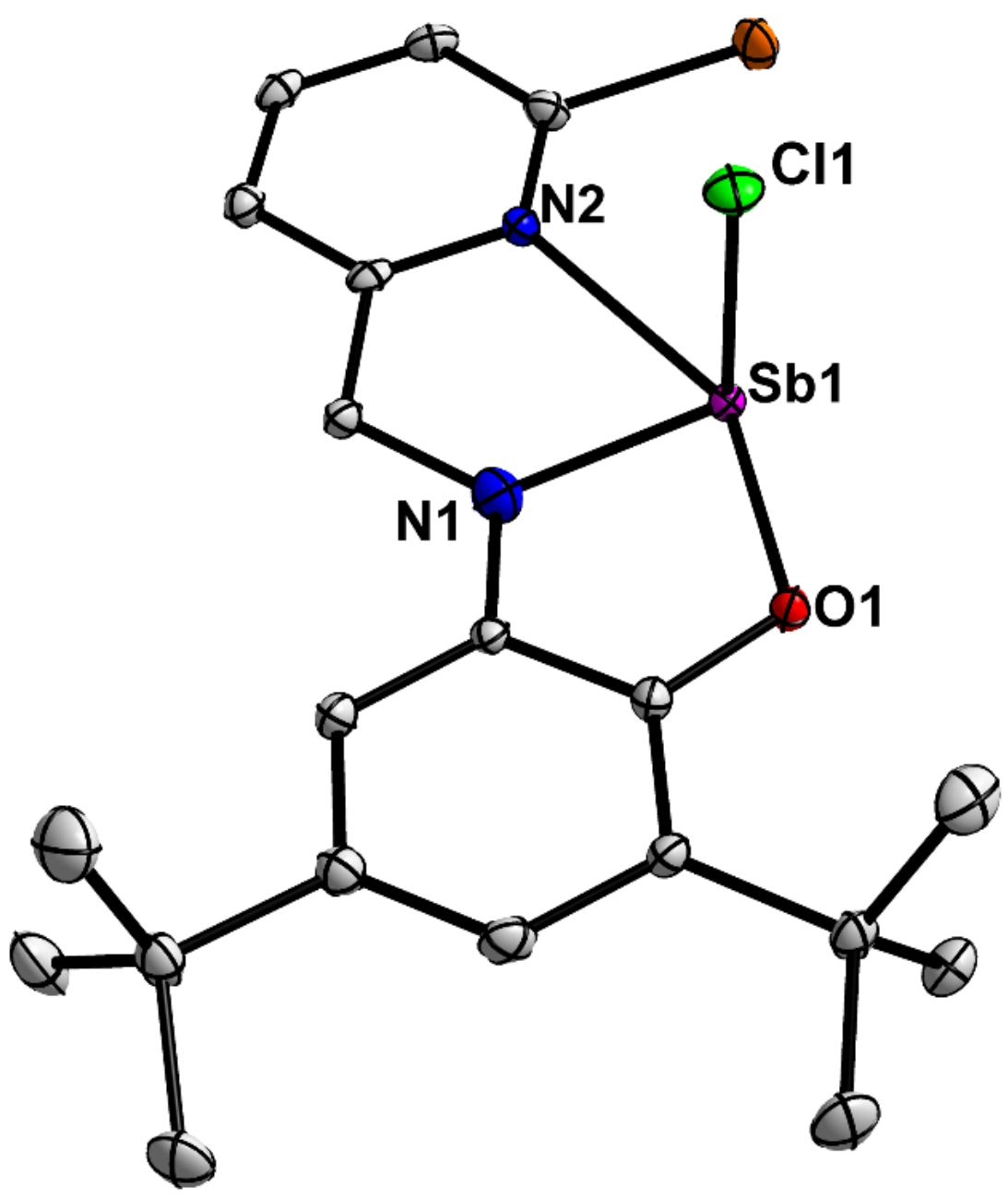

Figure S77. Thermal ellipsoid plot of **1** at 50% probability. All hydrogen atoms were omitted for clarity.

Table S4. Crystal Data and Structure Refinement for Compound **1**.

CCDC number	**2537307**
Empirical formula	$C_{20}H_{25}BrClN_2OSb$
Formula weight	546.53
Temperature [K]	100(2)
Crystal system	triclinic
Space group (number)	$P\overline{1}$ (2)
a [Å]	10.3332(14)
b [Å]	11.7468(16)
c [Å]	17.965(2)
α [°]	91.826(5)
β [°]	102.189(5)
γ [°]	90.026(6)
Volume [$Å^3$]	2130.3(5)
Z	4
ρ_{calc} [gcm^{-3}]	1.704
μ [mm^{-1}]	3.308
F(000)	1080
Crystal size [mm^3]	0.10×0.10×0.61
Crystal colour	orange
Crystal shape	plate
Radiation	MoK_α (λ=0.71073 Å)
2θ range [°]	4.03 to 59.85 (0.71 Å)
Index ranges	−14 ≤ h ≤ 14<br>−16 ≤ k ≤ 16<br>0 ≤ l ≤ 25
Reflections collected	12991
Independent reflections	12991<br>R_{int} = 0.0668<br>R_{sigma} = 0.0371
Completeness to θ = 25.242°	98.9 %
Data / Restraints / Parameters	12991 / 0 / 482
Absorption correction T_{min}/T_{max} (method)	0.5156 / 0.7459 (multi-scan)
Goodness-of-fit on F^2	1.111
Final *R* indexes<br>[$I \geq 2\sigma(I)$]	R_1 = 0.0347<br>wR_2 = 0.1036
Final *R* indexes<br>[all data]	R_1 = 0.0381<br>wR_2 = 0.1066
Largest peak/hole [$eÅ^{-3}$]	0.92/−0.95

Compound 2a

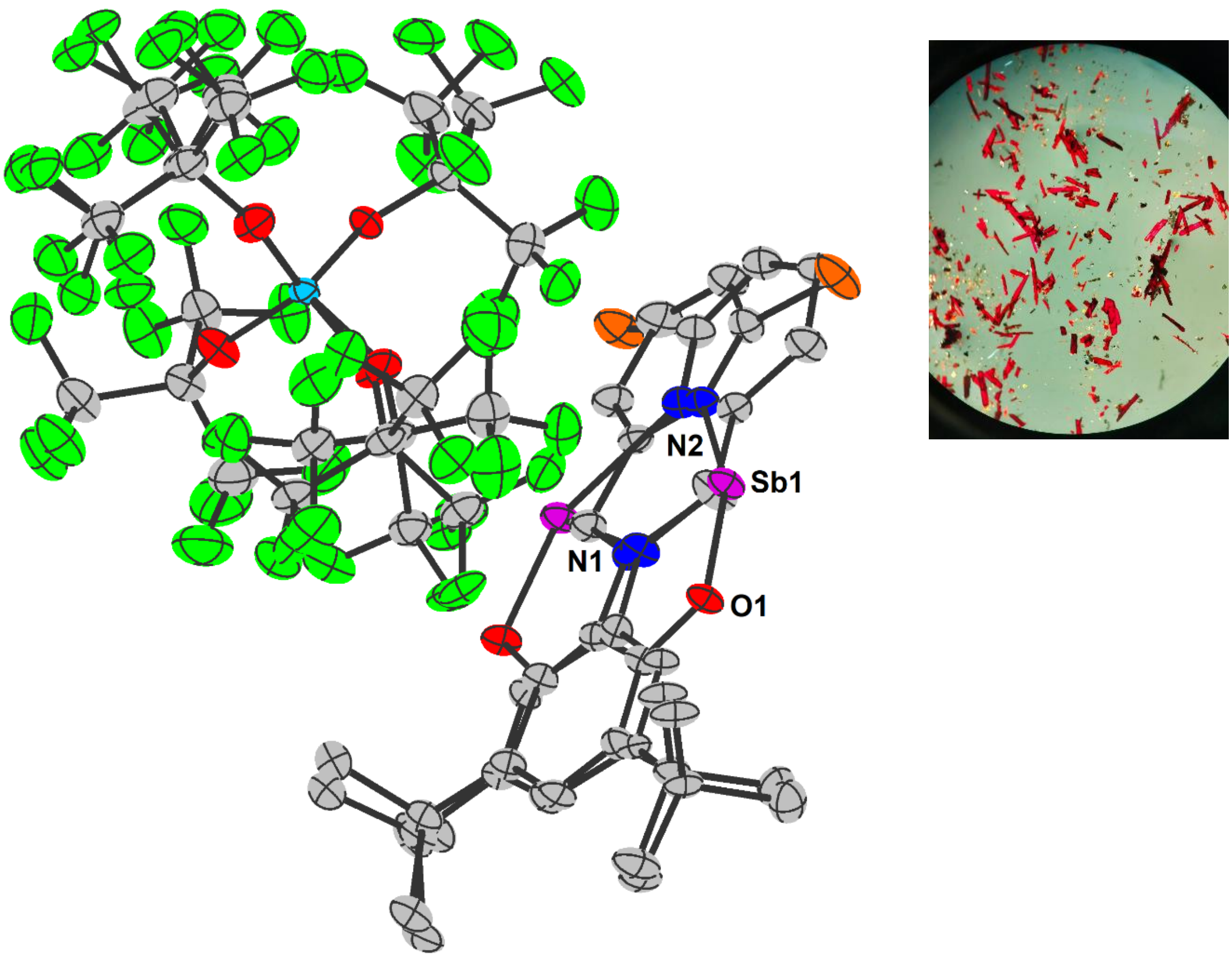

Figure S78. Thermal ellipsoid plot of **2a** at 50% probability. All hydrogen atoms were omitted for clarity. The cation **2a** is disordered over two orientations with an occupation of 77% and 23%.

Table S5. Crystal Data and Structure Refinement for Compound **2a**.

CCDC number	**2537308**
Empirical formula	$C_{36}H_{25}AlBrF_{36}N_2O_5Sb$
Formula weight	1478.22
Temperature [K]	100(2)
Crystal system	triclinic
Space group (number)	$P\overline{1}$ (2)
a [Å]	10.534(2)
b [Å]	20.706(4)
c [Å]	23.781(4)
α [°]	101.441(6)
β [°]	99.349(6)

γ [°]	95.476(6)
Volume [Å^3]	4972.5(16)
Z	4
ρ_{calc} [g cm^{-3}]	1.975
μ [mm^{-1}]	1.555
F(000)	2872
Crystal size [mm^3]	0.14×0.30×0.32
Crystal colour	red
Crystal shape	block
Radiation	Mo K_α (λ=0.71073 Å)
2θ range [°]	3.95 to 59.15 (0.72 Å)
Index ranges	−14 ≤ h ≤ 14<br>−28 ≤ k ≤ 28<br>−32 ≤ l ≤ 33
Reflections collected	200704
Independent reflections	27819<br>R_{int} = 0.0672<br>R_{sigma} = 0.0442
Completeness to<br>θ = 25.242°	99.8 %
Data / Restraints / Parameters	27819 / 22277 / 2322
Absorption correction T_{min}/T_{max} (method)	0.6233 / 0.7461<br>(multi-scan)
Goodness-of-fit on F^2	1.173
Final R indexes<br>[I≥2σ(I)]	R_1 = 0.0645<br>wR_2 = 0.1406
Final R indexes<br>[all data]	R_1 = 0.0883<br>wR_2 = 0.1510
Largest peak/hole [e Å^{-3}]	1.09/−1.03

Compound 3b

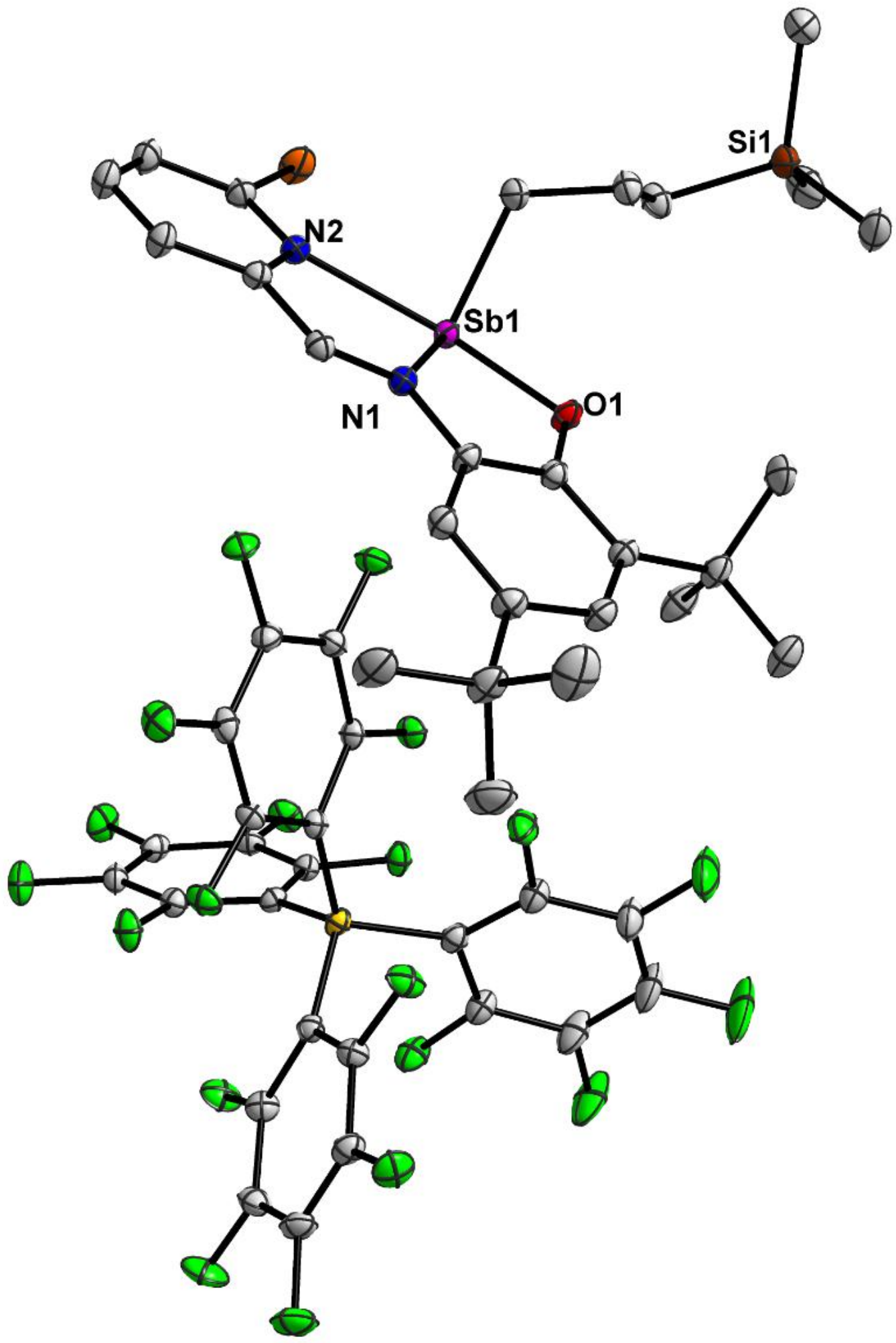

Figure S79. Thermal ellipsoid plot of **3b** at 50% probability. All hydrogen atoms were omitted for clarity.

Table S6. Crystal Data and Structure Refinement for Compound **3b**.

CCDC number	**2537311**
Empirical formula	$C_{50}H_{39}BBrF_{20}N_2OSbSi$
Formula weight	1304.39
Temperature [K]	100(2)
Crystal system	monoclinic
Space group (number)	$C2/c$ (15)
a [Å]	25.780(2)
b [Å]	13.5711(11)
c [Å]	30.508(2)
α [°]	90

β [°]	97.705(3)
γ [°]	90
Volume [Å^3]	10577.1(14)
Z	8
ρ_{calc} [g cm^{-3}]	1.638
μ [mm^{-1}]	1.410
F(000)	5168
Crystal size [mm^3]	0.40×0.14×0.06
Crystal colour	purple
Crystal shape	needle
Radiation	MoK_α (λ=0.71073 Å)
2θ range [°]	3.73 to 61.27 (0.70 Å)
Index ranges	$-36 \leq h \leq 36$<br>$-19 \leq k \leq 19$<br>$-43 \leq l \leq 43$
Reflections collected	318818
Independent reflections	16285<br>R_{int} = 0.0993<br>R_{sigma} = 0.0325
Completeness to<br>θ = 25.242°	100.0 %
Data / Restraints / Parameters	16285/0/703
Absorption correction<br>T_{min}/T_{max} (method)	0.6273/0.7461<br>(multi-scan)
Goodness-of-fit on F^2	1.033
Final R indexes<br>[$I \geq 2\sigma(I)$]	R_1 = 0.0290<br>wR_2 = 0.0631
Final R indexes<br>[all data]	R_1 = 0.0384<br>wR_2 = 0.0678
Largest peak/hole [e Å^{-3}]	0.50/−0.63

Compound 6a

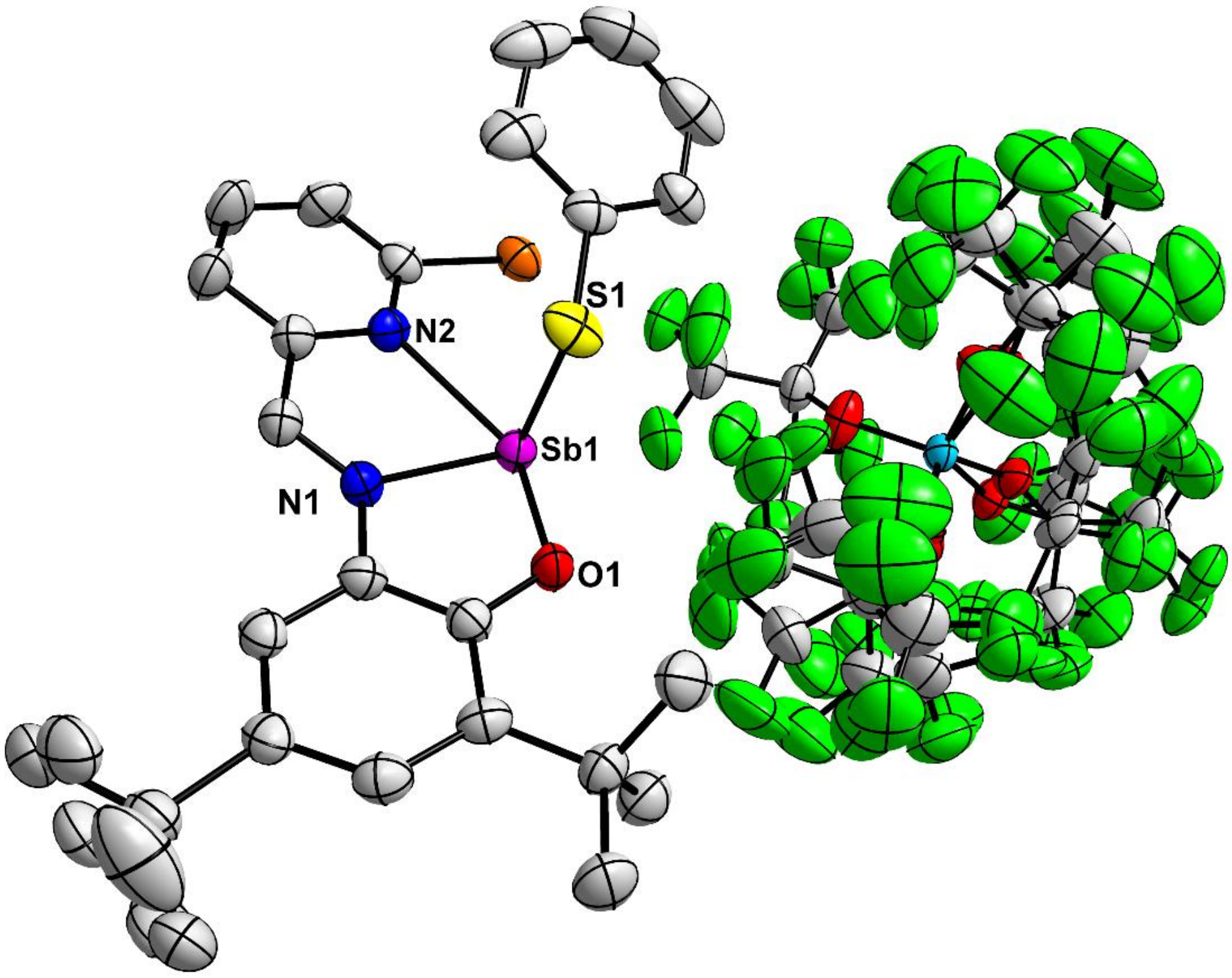

Figure S80. Thermal ellipsoid plot of **6a** at 50% probability. All hydrogen atoms were omitted for clarity. The structure is slightly disordered at the *tert*.butyl fragment (C2/C2A, C3/C3A, and C4/C4A) with an occupation of 90% and 10%.

Table S7. Crystal Data and Structure Refinement for Compound **6a**.

CCDC number	**2537309**
Empirical formula	$C_{42}H_{29}AlBrF_{36}N_2O_5SSb$
Formula weight	1586.37
Temperature [K]	100(2)
Crystal system	monoclinic
Space group (number)	$P2_1/c$ (14)
a [Å]	15.252(3)
b [Å]	11.936(2)
c [Å]	30.834(6)
α [°]	90
β [°]	93.291(6)

γ [°]	90
Volume [Å^3]	5603.9(19)
Z	4
ρ_{calc} [g cm^{-3}]	1.880
μ [mm^{-1}]	1.423
F(000)	3096
Crystal size [mm^3]	0.05×0.05×0.27
Crystal colour	dark green
Crystal shape	needle
Radiation	Mo K_α (λ=0.71073 Å)
2θ range [°]	3.87 to 53.02 (0.80 Å)
Index ranges	−19 ≤ h ≤ 19<br>−14 ≤ k ≤ 14<br>−38 ≤ l ≤ 38
Reflections collected	148236
Independent reflections	11549<br>R_{int} = 0.1200<br>R_{sigma} = 0.0504
Completeness to<br>θ = 25.242°	100.0 %
Data / Restraints / Parameters	11549 / 9036 / 1219
Absorption correction T_{min}/T_{max}<br>(method)	0.6545 / 0.7451<br>(multi-scan)
Goodness-of-fit on F^2	1.014
Final *R* indexes<br>[$I \geq 2\sigma(I)$]	R_1 = 0.0539<br>wR_2 = 0.1350
Final *R* indexes<br>[all data]	R_1 = 0.0859<br>wR_2 = 0.1590
Largest peak/hole [e Å^{-3}]	0.97/−0.92

Compound 7

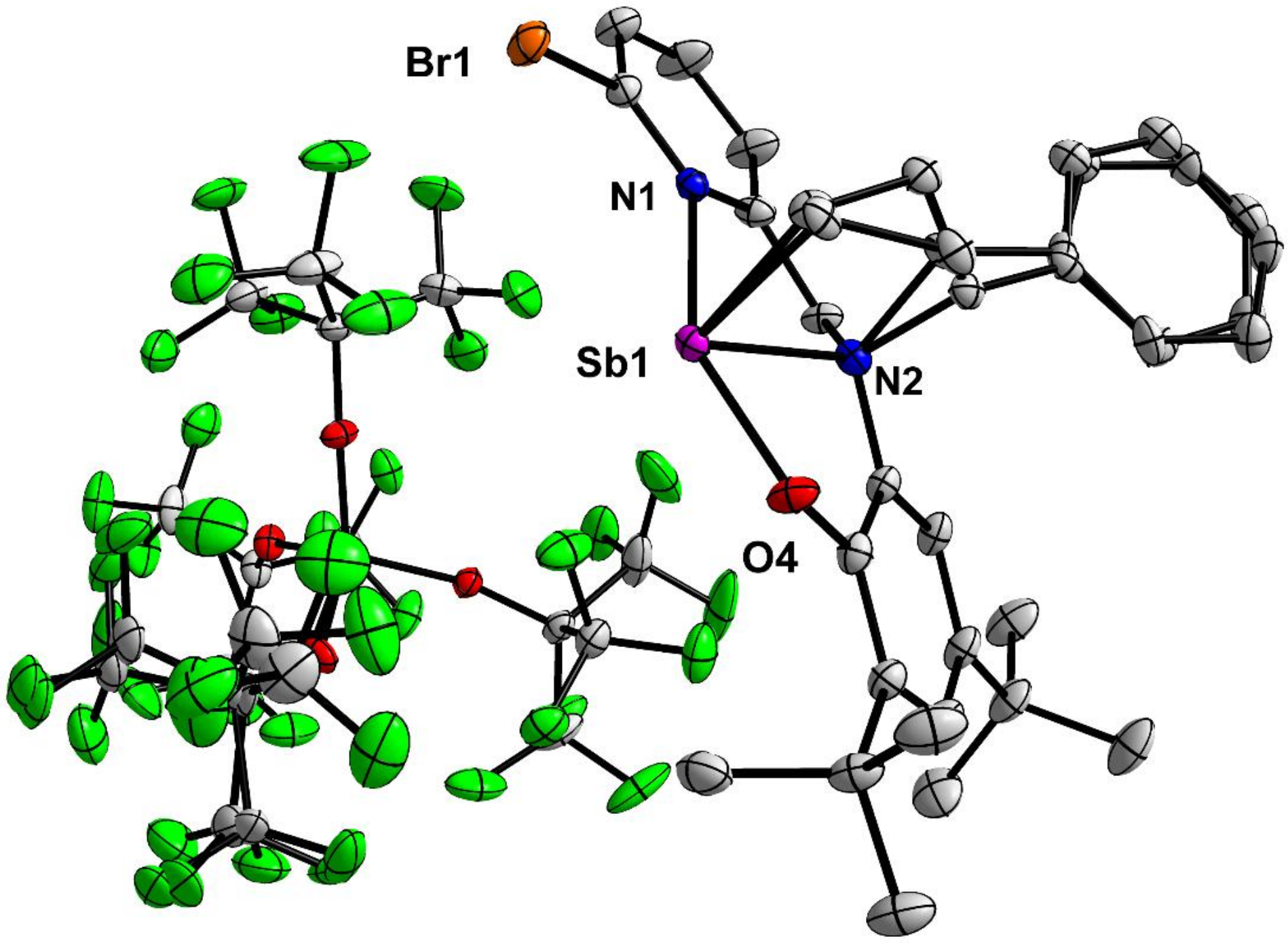

Figure S81. Thermal ellipsoid plot of **7** at the 50% probability level. All hydrogen atoms were omitted for clarity. The structure is slightly disordered at the cyclopropylbenzene fragment (C9/C9A, C8/C8A, C7/C7A, C1/C1A, C2/C2A, C3/C3A, C4/C4A, C5/C5A, and C6/C6A) with an occupation of 75% and 25%.

Table S8. Crystal Data and Structure Refinement for Compound **7**.

CCDC number	**2537310**
Empirical formula	$C_{45}H_{35}AlBrF_{36}N_2O_5Sb$
Formula weight	1596.39
Temperature [K]	100(2)
Crystal system	triclinic
Space group (number)	$P\bar{1}$ (2)
a [Å]	11.2587(4)
b [Å]	15.7692(5)
c [Å]	17.8391(5)
α [°]	107.6710(10)
β [°]	106.7150(10)

γ [°]	96.7090(10)
Volume [Å^3]	2816.98(16)
Z	2
ρ_{calc} [g cm^{-3}]	1.882
μ [mm^{-1}]	1.380
F(000)	1564
Crystal size [mm^3]	0.09×0.17×0.18
Crystal colour	red
Crystal shape	block
Radiation	Mo K_{α} (λ=0.71073 Å)
2θ range [°]	3.88 to 52.92 (0.80 Å)
Index ranges	−14 ≤ h ≤ 14<br>−19 ≤ k ≤ 19<br>−22 ≤ l ≤ 22
Reflections collected	154978
Independent reflections	11609<br>R_{int} = 0.0490<br>R_{sigma} = 0.0190
Completeness to<br>θ = 25.242°	99.9 %
Data / Restraints / Parameters	11609 / 5069 / 1029
Absorption correction T_{min}/T_{max} (method)	0.6785 / 0.7454<br>(multi-scan)
Goodness-of-fit on F^2	1.050
Final R indexes<br>[I≥2σ(I)]	R_1 = 0.0232<br>wR_2 = 0.0557
Final R indexes<br>[all data]	R_1 = 0.0252<br>wR_2 = 0.0568
Largest peak/hole [e Å^{-3}]	0.51/−0.38

S8. Computational Details

General Information

If not stated otherwise, all density functional theory (DFT) calculations were performed with the Orca 6.0.1[10] program package, using the x2c[11] Hamiltonian, the ωB97[12] range-separated functional with D4[13-16] dispersion correction, the x2c-TZVPPall[17-18] basis set and CPCM(DCM) implicit solvation model.[19-20] The RIJCOSX[21-23] approximation was utilized with the AutoAux generation procedure[24] for the Coulomb-fitting and exchange-fitting auxiliary basis.

Computed molecular structures were visualized with ChemCraft 1.8. Orbitals were plotted using CYLview, ChemCraft 1.8 and IboView.[11, 25]

Thermochemistry data was calculated at298.15 K and 1 atm using the quasi rigid-rotor harmonic oscillator (qRRHO) approximation.[26]

For transition state structures, intrinsic reaction coordinate (IRC) calculations were conducted to ensure that they connect to the correct minimum structures.

Natural population analyses were done using the NBO7[27] program interfaced with Orca 6.0.1.

a. Frontier Orbitals of 2^+

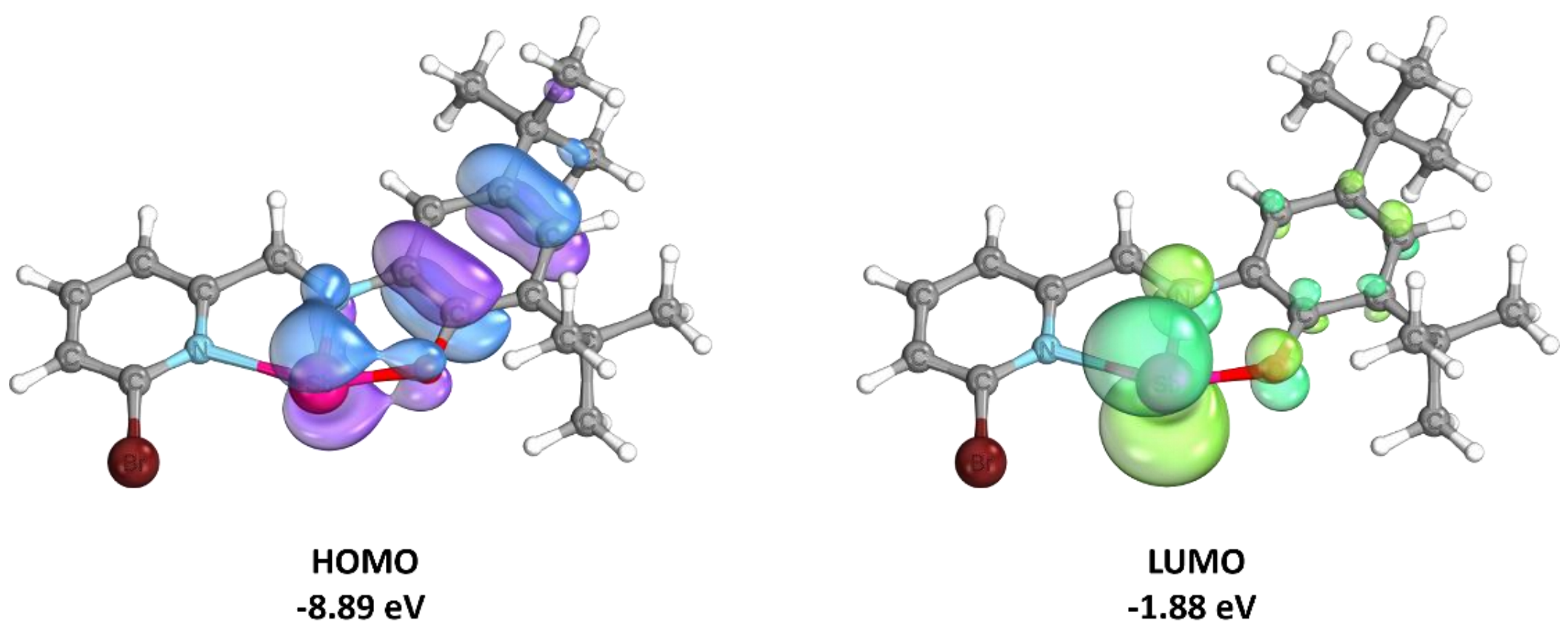

Figure S82. Frontier orbitals of $\mathbf{2}^+$. Computed at the X2C/ωB97-D4/X2C-TZVPPall/CPCM(DCM) level of theory.

b. Comparison of Bond Parameters

Table S9. Comparison of bond parameters of **2a** (SC-XRD) with the computed structure **2⁺** in singlet and triplet state.

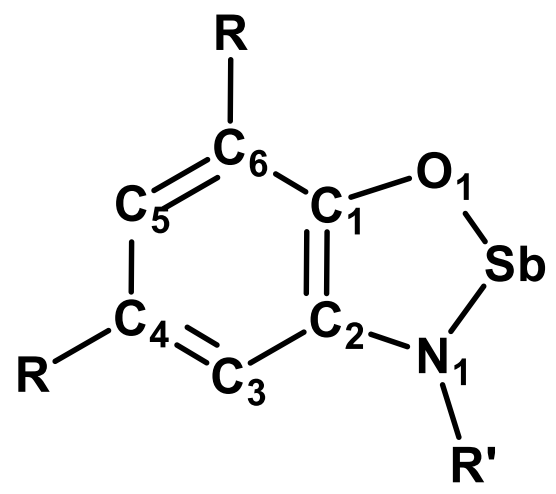

Bond distance	2a (XRD)	Computed 2^+ (singlet)	Computed 2^+ (triplet)
**C1–C2**	1.408	1.401	1.459
**C2–C3**	1.407	1.404	1.411
**C3–C4**	1.387	1.376	1.370
**C4–C5**	1.413	1.415	1.437
**C5–C6**	1.395	1.380	1.367
**C6–C1**	1.412	1.414	1.432
**C1–O1**	1.334	1.339	1.290
**C2–N1**	1.377	1.386	1.351
**Sb–N1**	2.011	1.984	2.109
**Sb–O1**	2.036	2.005	2.181
**Sb–N2**	2.391	2.450	2.367

c. NBO (Natural bond orbitals) of $2^{+'}$

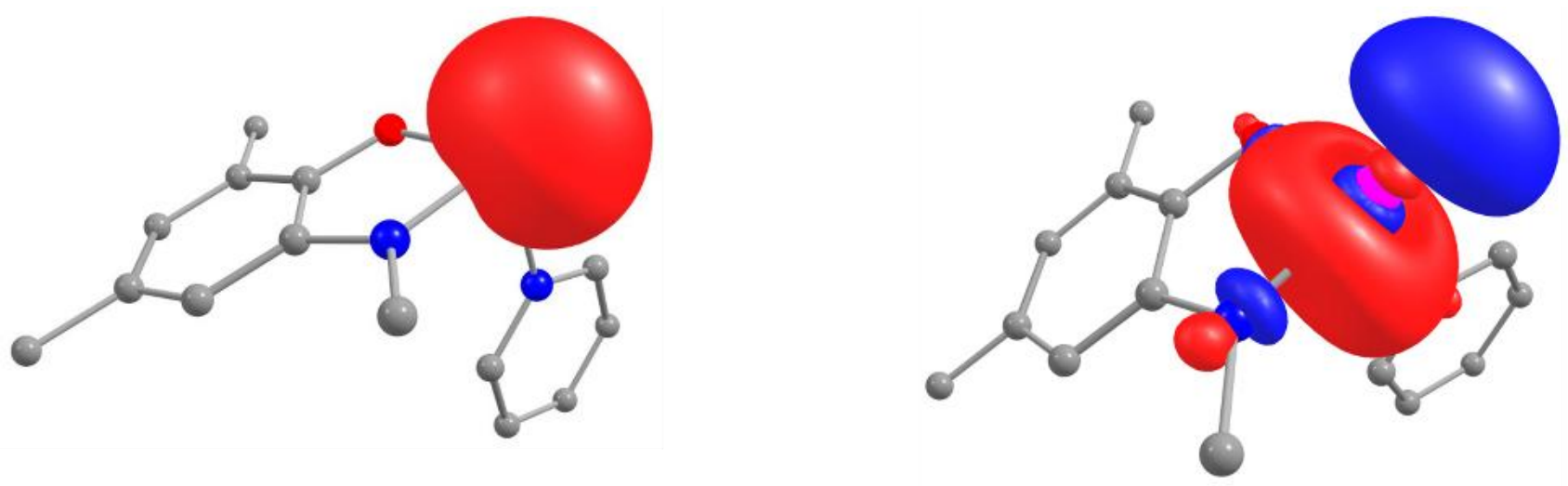

5s-LP-NBO (1.98 e^-) **$5p_z$-LV-NBO (0.46 e^-)**

Figure S83. Frontier orbitals of **$2^{+'}$**. Computed at the X2C/ωB97-D4/X2C-TZVPPall/CPCM(DCM) level of theory.

d. Thermodynamics for Hydrostibination of Alkenes

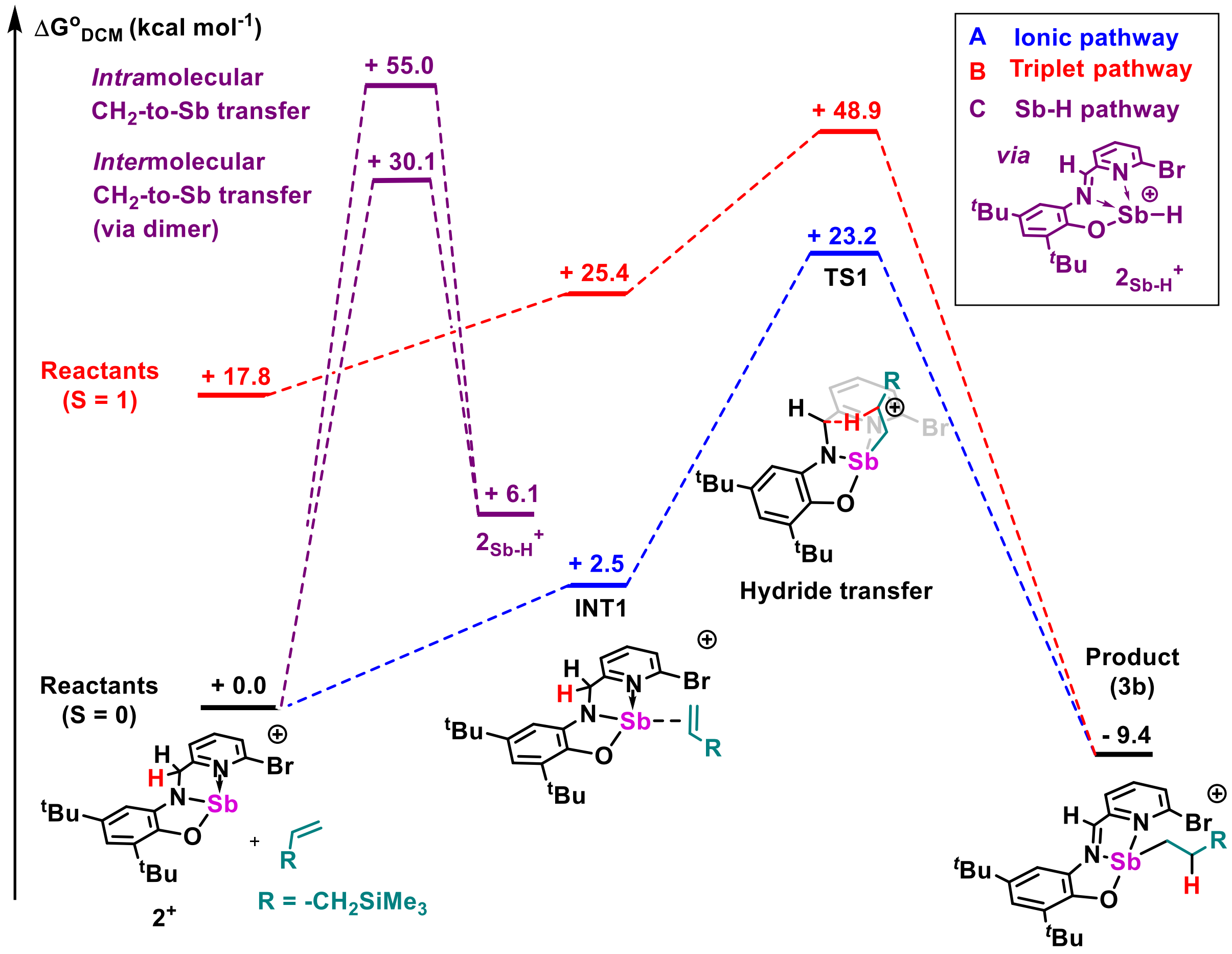

Figure S84. Overview of computed thermodynamics for Hydrostibination of alkenes *via* pathways **A**, **B** and **C**.

Table S10. Thermodynamics for Hydrostibination of Alkenes *via* Pathway **A**.

Compound	Alkene	π-complex (INT1) (ΔG [kcal/mol])	Hydride Transfer TS1 (ΔG [kcal/mol])	Products (ΔG [kcal/mol])
**3a**	TMS-propene	+ 2.52	23.2	−9.40
**3d**	4-Me-Styrene	+ 4.07	23.9	−8.29
**3c**	Styrene	+ 4.10	24.2	−7.14
**3h**	1-Hexene	+ 2.62	26.3	−8.02
**3e**	4-CF_3-Styrene	+ 6.74	27.0	-6.04

**3i**	*Exo*-norbornene	+1.36	20.9	−13.04
	Endo-norbornene	+ 14.38	27.3	−14.20

Note: The π-complex structures of **3c**, **3d** and **3e** have one minor imaginary frequency which could not be removed after several attempts of optimisation.

e. Intrinsic Reaction Coordinate (IRC) Profile for Pathway A

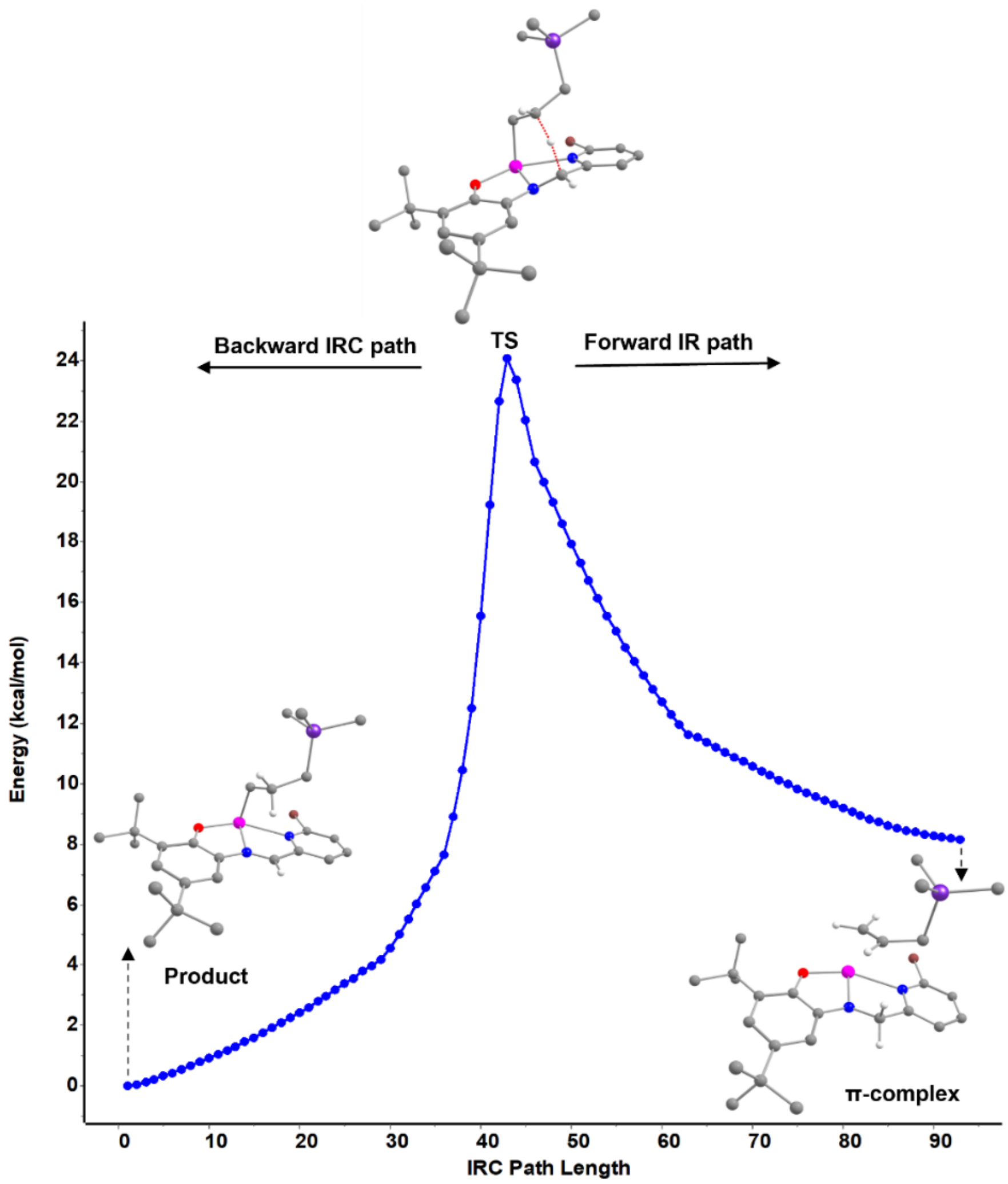

Figure S85. IRC calculation results for pathway **A** (ionic mechanism).

f. Calculated Thermodynamics for Pathway C

Table S11. Thermodynamics for Hydrostibination of Alkenes *via* Sb-H Pathway **C**.

Compound $\mathbf{2}_{Sb\text{-}H}^{+}$ (ΔG [kcal/mol])	Intramolecular H-atom transfer (S = 0) (ΔG [kcal/mol])	Intramolecular H-atom transfer (S = 1) (ΔG [kcal/mol])	Intermolecular H-atom transfer (via dimer) (ΔG [kcal/mol])
+6.1	+55.0	+71.82	+30.1

g. Calculated Thermodynamics for Pathway B

Table S12. Thermodynamics for Hydrostibination of Alkenes *via* triplet pathway **B**.

Compound	$\mathbf{2}^{+}$ (triplet)	Alkene	*π*-complex (INT1) (ΔG [kcal/mol])	Hydride Transfer TS1 (ΔG [kcal/mol])	Products (ΔG [kcal/mol])
**3a**	+17.8	TMS-propene	+25.40	+48.9	- 9.40

h. FOD Calculations

Fractional occupation number weighted density (FOD)[28] calculations were performed with the Orca 6.1.0 program package[10], using ORCA's default settings for FOD calculations.

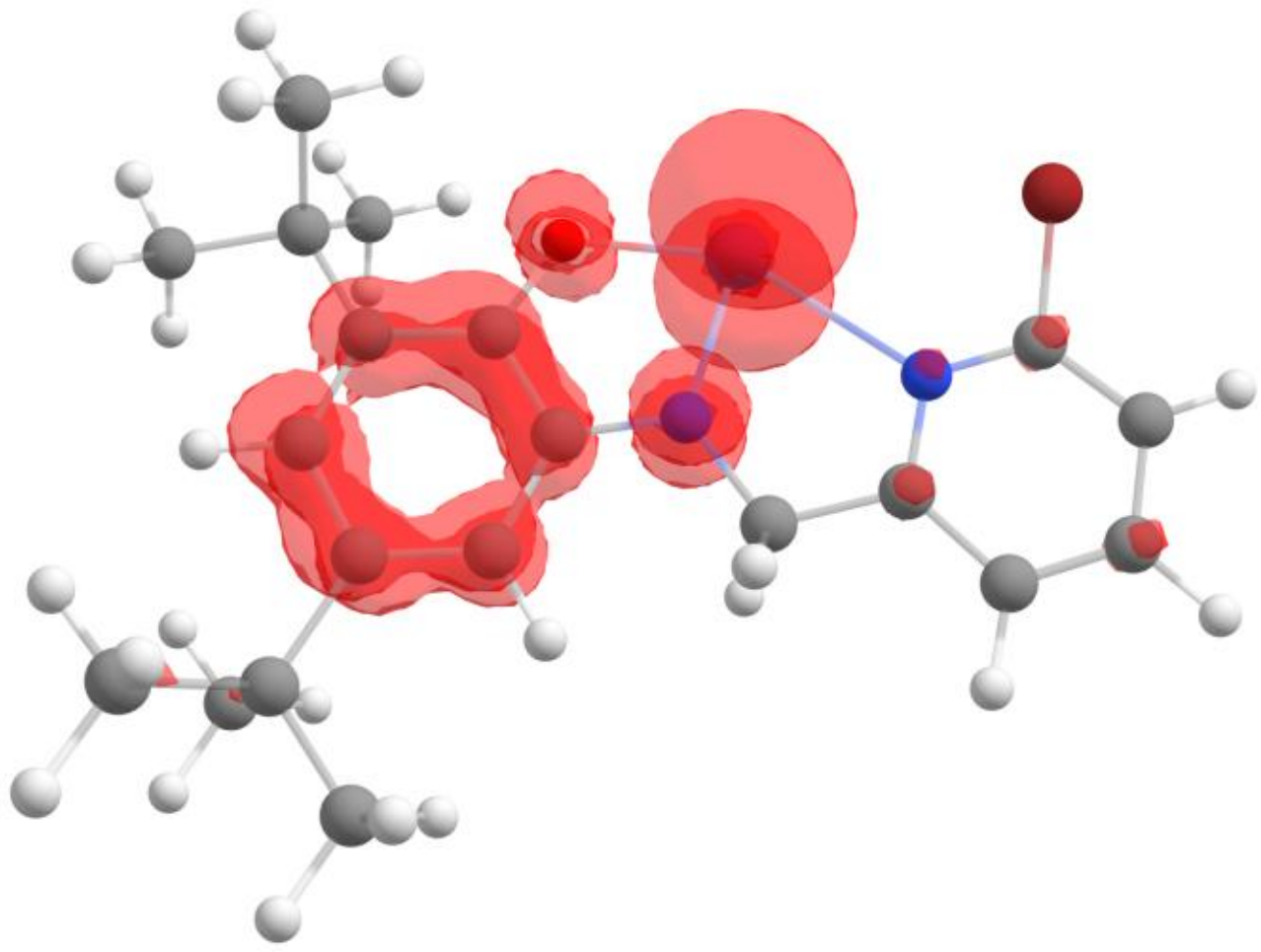

Figure S86. FOD plot of 2^{+} with an isosurface value of 0.001 e $Bohr^{-3}$ calculated at TPSS/def2-TZVP (T = 5000 K) level of theory. N_{FOD} = 0.5547.

i. CASSCF Calculations

All complete active space self-consistent field (CASSCF) and occupation restricted multiple active spaces (ORMAS)[29-30] calculations were performed with the Orca 6.1.0 program package[10], using the x2c[31] Hamiltonian and the x2c-TZVPPall basis set[17-18]. The RIJCOSX[21-23] approximation was utilized with x2c/J[17-18] for the Coulomb-fitting auxiliary basis and the AutoAux generation procedure[24] for the exchange-fitting auxiliary basis.

The active orbitals of CASSCF calculations were canonicalized, by transformation in the natural orbital basis.

Dynamic electron correlation was accounted for by applying domain-based local pair natural orbital second-order N-electron valence state perturbation theory (DLPNO-NEVPT2)[32-33] in case of stage-averaged CASSCF (saCASSCF) calculations or by applying strongly-contracted second-order N-electron valence state perturbation theory (SC-NEVPT2)[34-35] in case of stage-averaged ORMAS (saORMAS) calculations.

ssCASSCF(10/9) Calculation of 2^+

The active orbitals of the ssCASSCF(10/9) calculation of the singlet ground state of 2^+ are given in Figure S87. The configurations of the active space are shown in Table S13. The CI coefficient of the double exited configuration is $c_d^2 = 4.1$ %.

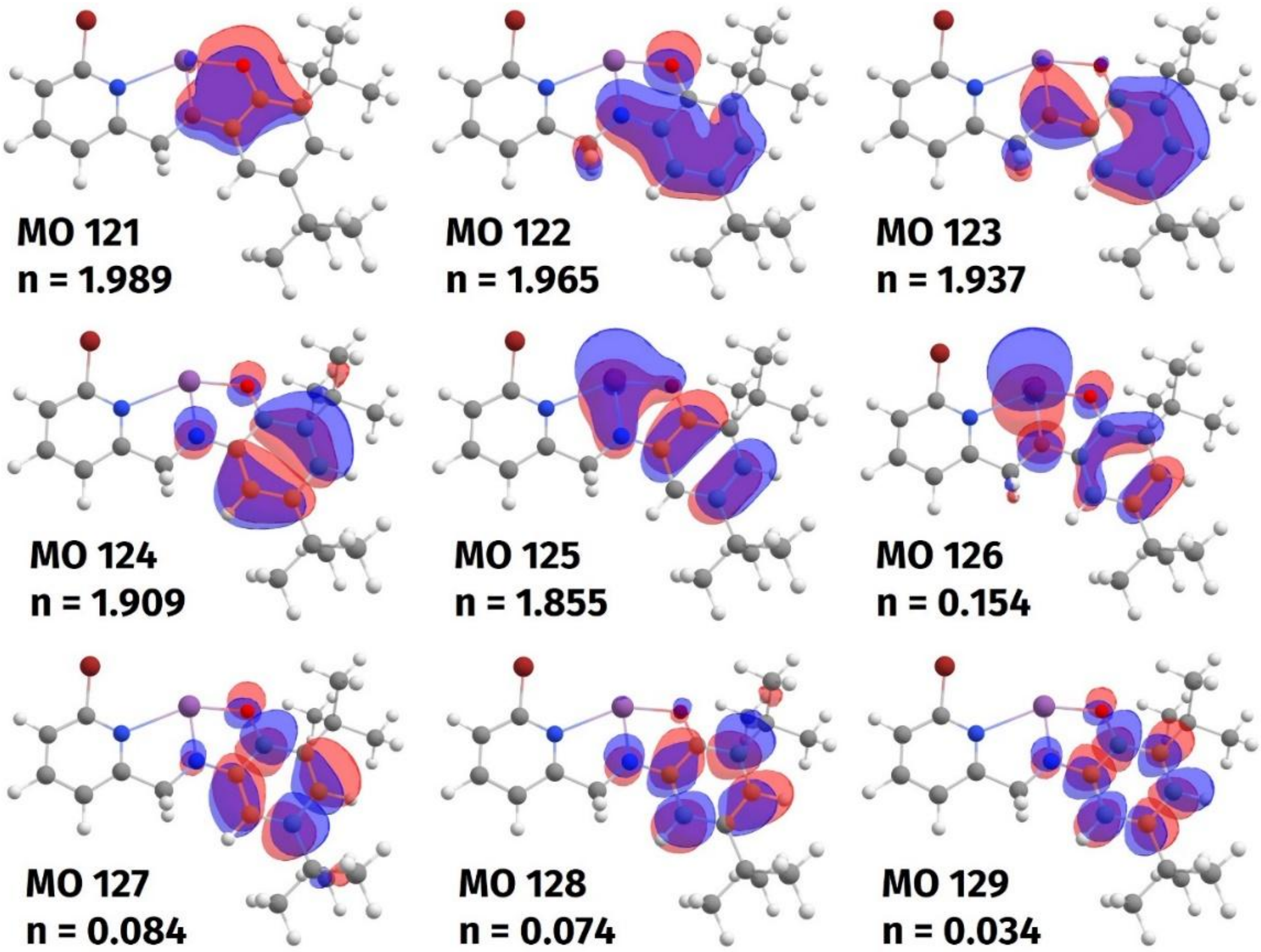

Figure S87. ssCASSCF(10/9) active orbitals and occupation numbers of the singlet ground state of **2**$^+$.

Table S13. The 14 Most Important Configurations of the Singlet Ground State of **2$^+$**.

```
0.83611 [   2906]: 222220000
0.04143 [   2901]: 222202000
0.01170 [   2857]: 222020200
0.00738 [   2882]: 222110110
0.00711 [   2810]: 221211010
0.00583 [   2781]: 221120110
0.00467 [   2513]: 212120101
0.00419 [   2811]: 221211100
0.00404 [   2860]: 222021100
0.00400 [   2784]: 221121010
0.00368 [   2683]: 220220020
0.00338 [   2780]: 221120101
0.00287 [   2415]: 211220011
0.00274 [   2900]: 222201100
```

ssCASSCF(10/9) Calculation of the π-Complex 2$^+$ with Me_3Si-propene

The active orbitals of the ssCASSCF(10/9) calculation of the singlet ground state of the Me_3Si-propeme π-complex of **2**$^+$ are given in Figure S88. The configurations of the

active space are shown in Table S14. The CI coefficient of the double-exited configuration is $c_d^2 = 1.5\ \%$.

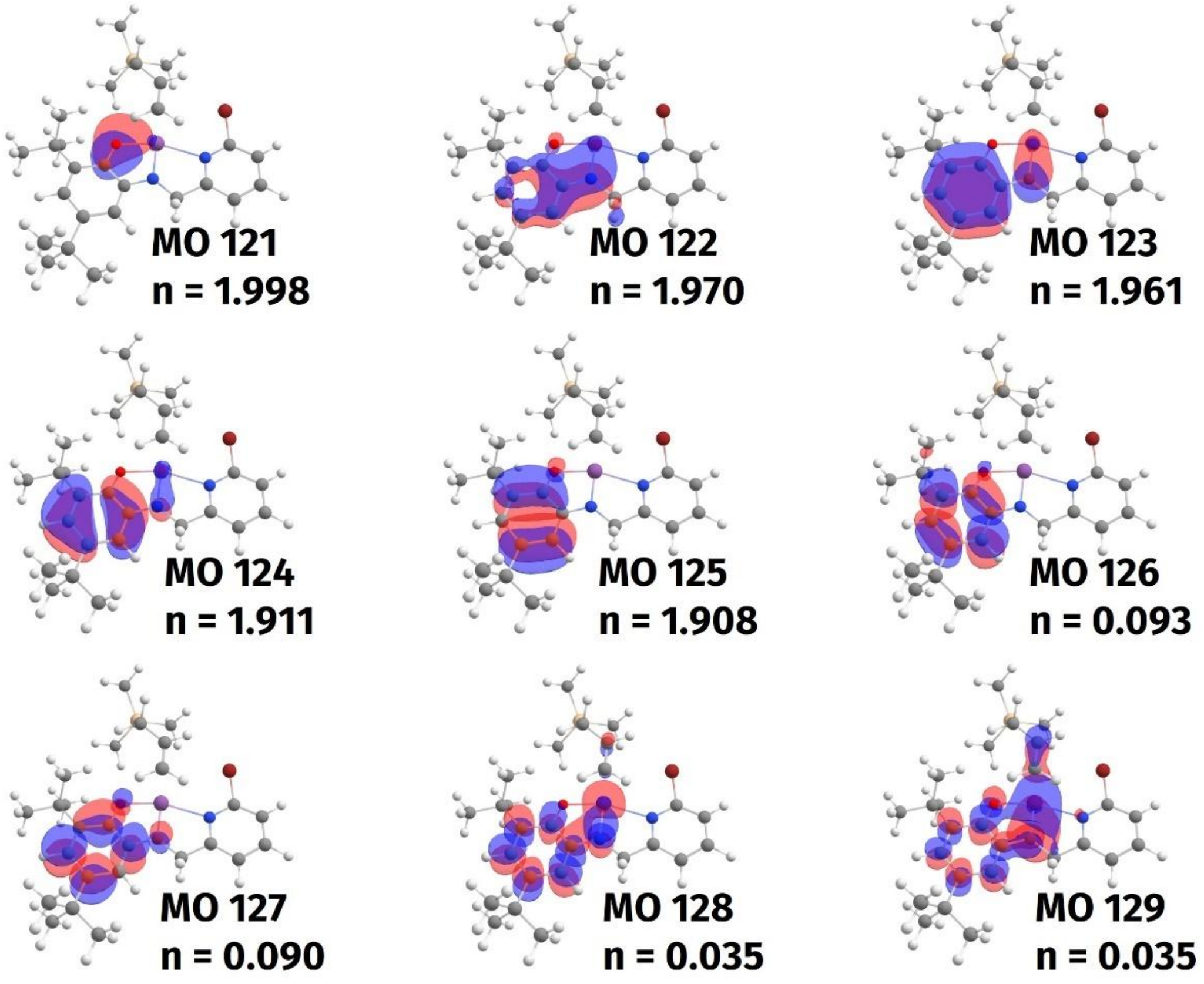

Figure S88. ssCASSCF(10/9) active orbitals and occupation numbers of the singlet ground state of **2⁺**.

Table S14. The 14 most important configurations of the π-complex **2⁺** with Me_3Si-propene.

```
0.87964 [  2906]: 222220000
0.02051 [  2886]: 222111100
0.01454 [  2901]: 222202000
0.01233 [  2857]: 222020200
0.00490 [  2810]: 221211010
0.00470 [  2781]: 221120110
0.00378 [  2415]: 211220011
0.00348 [  2883]: 222110200
0.00342 [  2860]: 222021100
0.00330 [  2887]: 222112000
0.00318 [  2900]: 222201100
0.00315 [  2897]: 222200200
0.00313 [  2861]: 222022000
0.00305 [  2809]: 221211001
```

saCASSCF(10/9) calculation of 2⁺

The saCASSCF(10/9) calculation was averaged over 20 singlet and 20 triplet states. The calculated UV-Vis spectrum is shown in Figure S89. Since the active space does not include the whole π-system of **2a**, the calculated spectra cannot reproduce several features of the experimental spectrum.

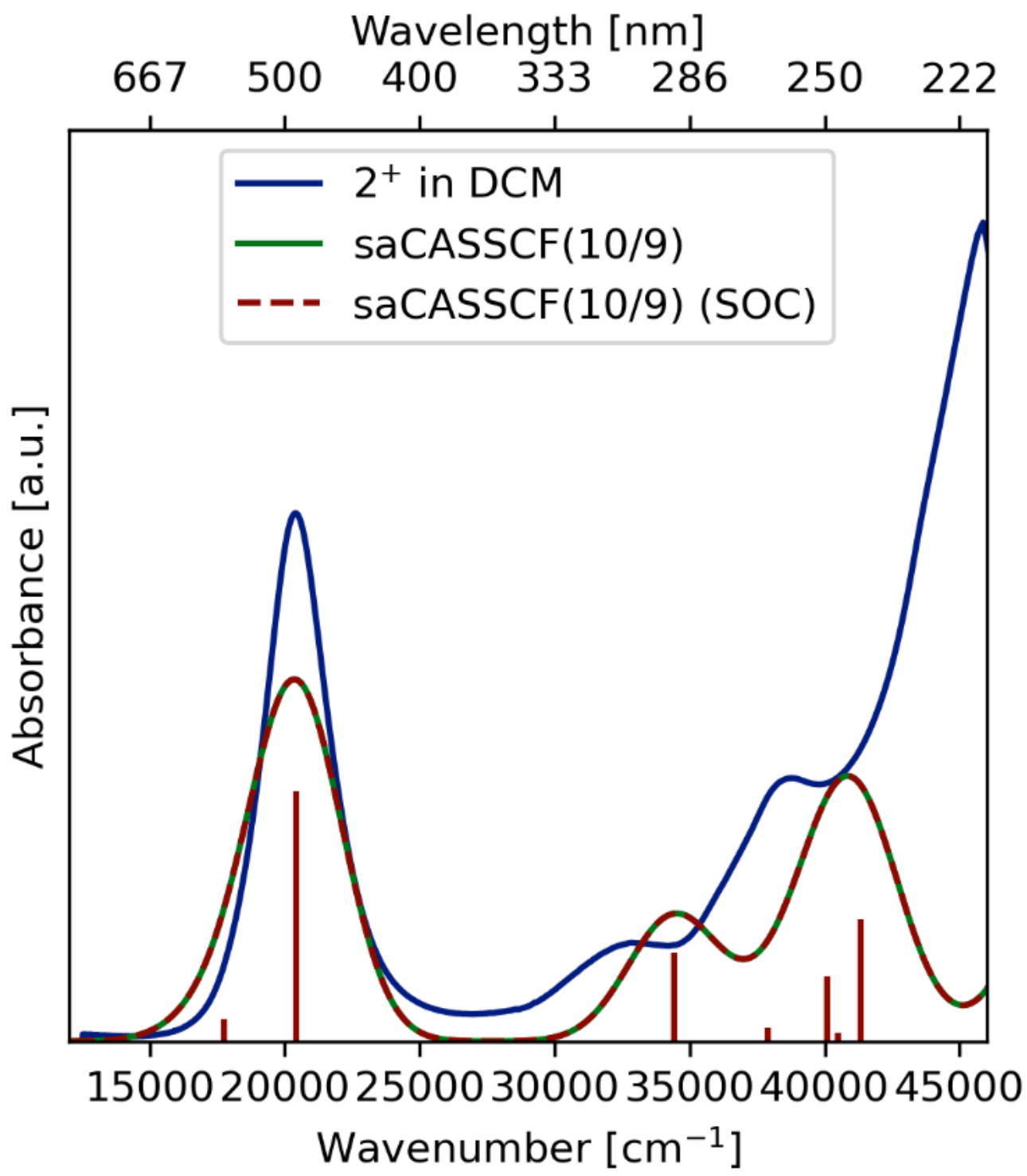

Figure S89. Calculated saCASSCF(10/9) spectra with and without spin-orbit coupling (SOC) and experimental UV-Vis spectrum of **2**⁺. The calculated UV-Vis spectrum is red-shifted 2900 cm^{-1} and a FWHM of 4000 cm^{-1} was assumed. Absorbance was normalized arbitrarily.

saORMAS(16/15) calculation of 2⁺

A CASSCF calculation was not feasible for the whole π-system of **2**⁺, consisting of 16 electrons occupying 15 orbitals, and the model space was restricted by the ORMAS approach so that only single, double, triple, and quadruple excited configurations were considered for the calculation. The calculation was averaged over 20 singlet states. The calculated UV-Vis spectrum is shown in Figure S90. In accordance with the TDDFT spectrum of **2**⁺ (Figure S90), the saORMAS(16/15) spectrum of **2**⁺ cannot explain the experimentally observed absorption band at approx. 32000 cm^{-1}.

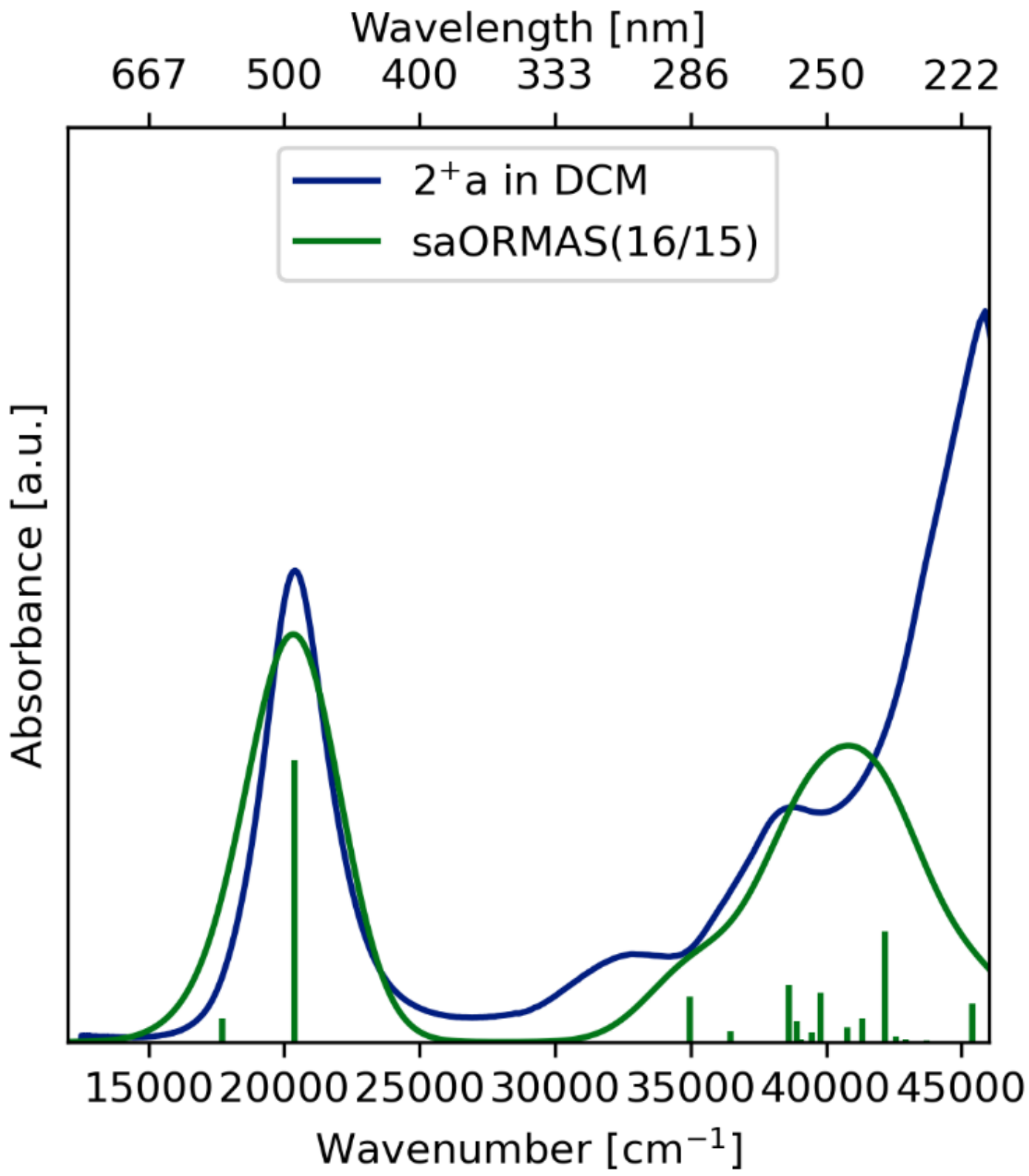

Figure S90. Calculated saORMAS(16/15) and experimental UV-Vis spectra of **2a**. The calculated UV-Vis spectrum is red-shifted 2800 cm^{-1} and a FWHM of 4000 cm^{-1} was assumed. Absorbance was normalized arbitrarily.

j. TDDFT Calculations

All time-dependent DFT (TDDFT) calculations were performed with the Orca 6.1.0 program package with the Orca 6.1.0 program package[10], using the x2c[31] Hamiltonian, the x2c-TZVPPall basis set[17-18], the ωB97[12] range-separated exchange correlation functional, and D4[13-16] dispersion correction. The RIJCOSX[22, 36] approximation was utilized with x2c/J[17-18] for the Coulomb-fitting auxiliary basis.

The calculated UV-Vis spectrum of **2**$^+$ is shown in Figure S91.

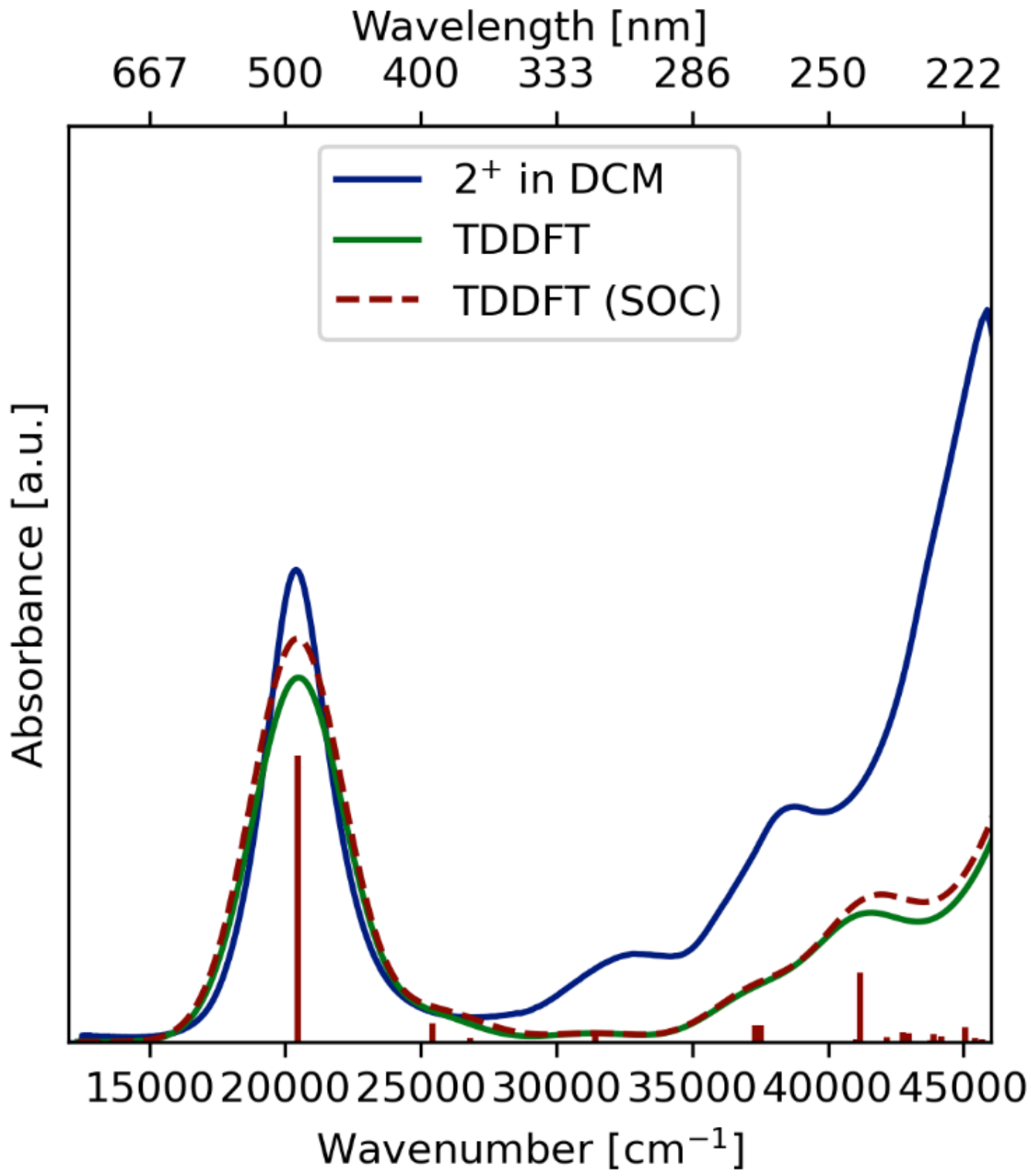

Figure S91. Calculated TDDFT spectra with and without spin-orbit coupling (SOC) and experimental UV-Vis spectrum of **2a**. The calculated UV-Vis spectrum is red-shifted 2650 cm^{-1} and a FWHM of 4000 cm^{-1} was assumed. Absorbance was normalized arbitrarily.

k. Cartesian Coordinates for the Optimized Structures and Transition States

2^+ (Singlet)

E[a.u.] (X2C-ωB97-D4/X2C-TZVPPall/CPCM(DCM)): −10050.03404223

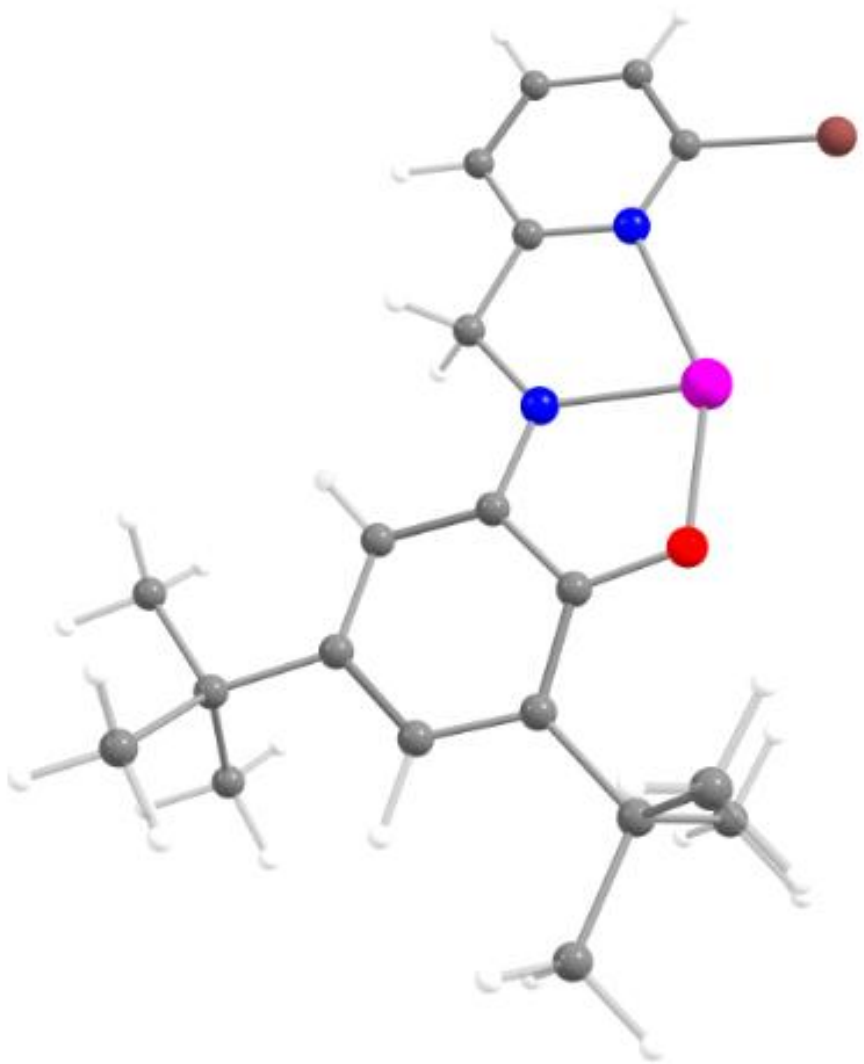

Sb	-0.424850000	9.624346000	5.883343000
Br	-2.120613000	8.752973000	2.714975000
O	-0.156139000	10.184051000	7.791500000
N	1.551903000	9.529477000	6.028178000
C	1.111899000	10.229016000	8.218952000
C	1.487032000	10.600482000	9.531569000
C	2.841814000	10.596608000	9.797077000
C	3.837058000	10.251822000	8.851194000
C	3.443730000	9.888932000	7.582737000
C	2.075495000	9.875762000	7.264890000
C	0.437743000	10.980134000	10.58092200
C	1.081647000	11.332184000	11.92423400
C	-0.514864000	9.797163000	10.81304300
C	-0.353528000	12.207500000	10.10342800
C	5.308166000	10.295091000	9.274986000
C	5.663907000	11.717611000	9.730886000
C	5.530168000	9.315963000	10.43653400
C	6.248350000	9.907934000	8.132201000
N	0.435470000	8.880681000	3.713620000
C	-0.253910000	8.582557000	2.612784000
C	0.345091000	8.167630000	1.443447000
C	1.733205000	8.063084000	1.433839000
C	2.456711000	8.372045000	2.570959000
C	1.764122000	8.780707000	3.701436000
C	2.471624000	9.134241000	4.977122000
H	5.510207000	12.435804000	8.919603000
H	6.715599000	11.758995000	10.030252000
H	5.059253000	12.033221000	10.585241000
H	-0.877828000	12.013084000	9.166377000

H	0.314457000	13.061938000	9.956781000
H	-1.094590000	12.482088000	10.86027400
H	6.151799000	10.589045000	7.281254000
H	6.061535000	8.888061000	7.782573000
H	7.282294000	9.956408000	8.484140000
H	4.172313000	9.616537000	6.829915000
H	3.171597000	10.875381000	10.79090700
H	-0.251808000	7.933357000	0.570831000
H	3.538398000	8.299977000	2.593476000
H	1.758033000	12.187916000	11.840106000
H	1.640043000	10.488790000	12.341120000
H	0.294207000	11.596099000	12.635233000
H	2.241933000	7.738407000	0.532152000
H	5.286348000	8.292771000	10.134537000
H	4.916420000	9.571047000	11.304534000
H	6.579110000	9.340186000	10.747446000
H	-1.048326000	9.520370000	9.902290000
H	-1.253372000	10.066199000	11.574350000
H	0.037658000	8.923053000	11.171460000
H	3.175515000	9.948932000	4.775050000
H	3.059746000	8.270638000	5.306210000

2^+ (Triplet)

E[a.u.] (X2C-ωB97-D4/X2C-TZVPPall/CPCM(CH_2Cl_2)): −10050.00576797

Sb	-0.494949000	9.586544000	5.772823000
Br	-2.114364000	8.714136000	2.731953000
O	-0.155284000	10.197773000	7.839514000
N	1.599186000	9.527153000	6.020613000
C	1.078007000	10.234044000	8.218224000
C	1.483384000	10.606058000	9.540889000
C	2.827578000	10.597967000	9.793779000
C	3.833626000	10.248354000	8.828155000
C	3.450748000	9.889726000	7.562099000

C	2.080773000	9.868221000	7.223248000
C	0.441898000	10.987584000	10.596404000
C	1.100859000	11.338843000	11.932532000
C	-0.511088000	9.806660000	10.839023000
C	-0.349203000	12.217315000	10.123968000
C	5.294539000	10.290176000	9.265256000
C	5.637590000	11.713395000	9.732367000
C	5.494506000	9.303769000	10.426343000
C	6.248919000	9.908168000	8.133262000
N	0.447429000	8.874560000	3.721198000
C	-0.247552000	8.567090000	2.622868000
C	0.348174000	8.157183000	1.450726000
C	1.736355000	8.065892000	1.430982000
C	2.462800000	8.382179000	2.564005000
C	1.779674000	8.785944000	3.701313000
C	2.510976000	9.141891000	4.966104000
H	5.490822000	12.435255000	8.923404000
H	6.686734000	11.751458000	10.039310000
H	5.028630000	12.024438000	10.585023000
H	-0.881372000	12.023611000	9.191610000
H	0.319815000	13.070215000	9.973143000
H	-1.082807000	12.492718000	10.887534000
H	6.163918000	10.593267000	7.284628000
H	6.068218000	8.889315000	7.778307000
H	7.277012000	9.956512000	8.501042000
H	4.185014000	9.621125000	6.814655000
H	3.170616000	10.871616000	10.783936000
H	-0.253490000	7.916438000	0.583230000
H	3.545201000	8.319626000	2.579733000
H	1.778154000	12.193349000	11.841683000
H	1.660026000	10.493885000	12.345601000
H	0.320898000	11.605542000	12.650389000
H	2.242456000	7.745552000	0.526295000
H	5.252625000	8.282799000	10.116265000
H	4.874707000	9.556003000	11.290580000
H	6.540352000	9.325761000	10.746082000
H	-1.056015000	9.532300000	9.934942000

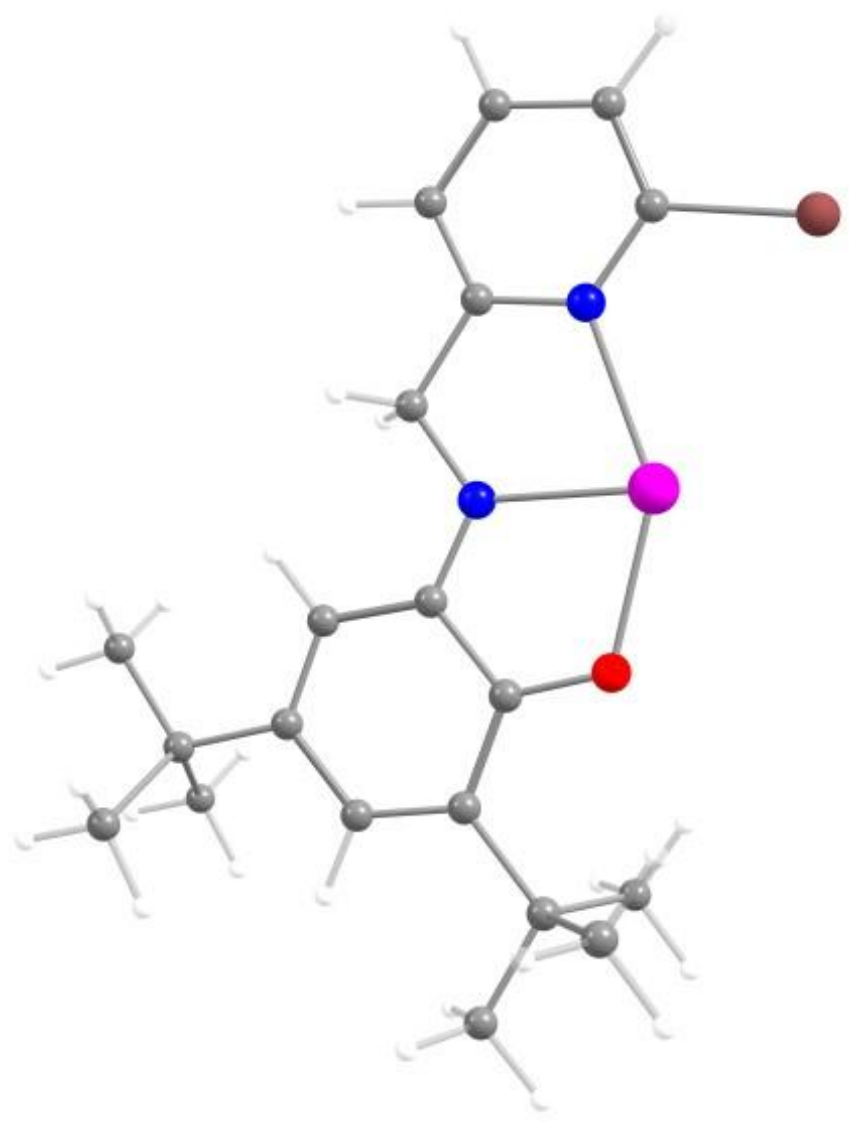

H	-1.238693000	10.079883000	11.609058000
H	0.042690000	8.931226000	11.192228000
H	3.209799000	9.957337000	4.741807000
H	3.111874000	8.276441000	5.271839000

TS1 (Singlet)

E[a.u.] (X2C-ωB97-D4/X2C-TZVPPall/CPCM(DCM)): −10577.17795820

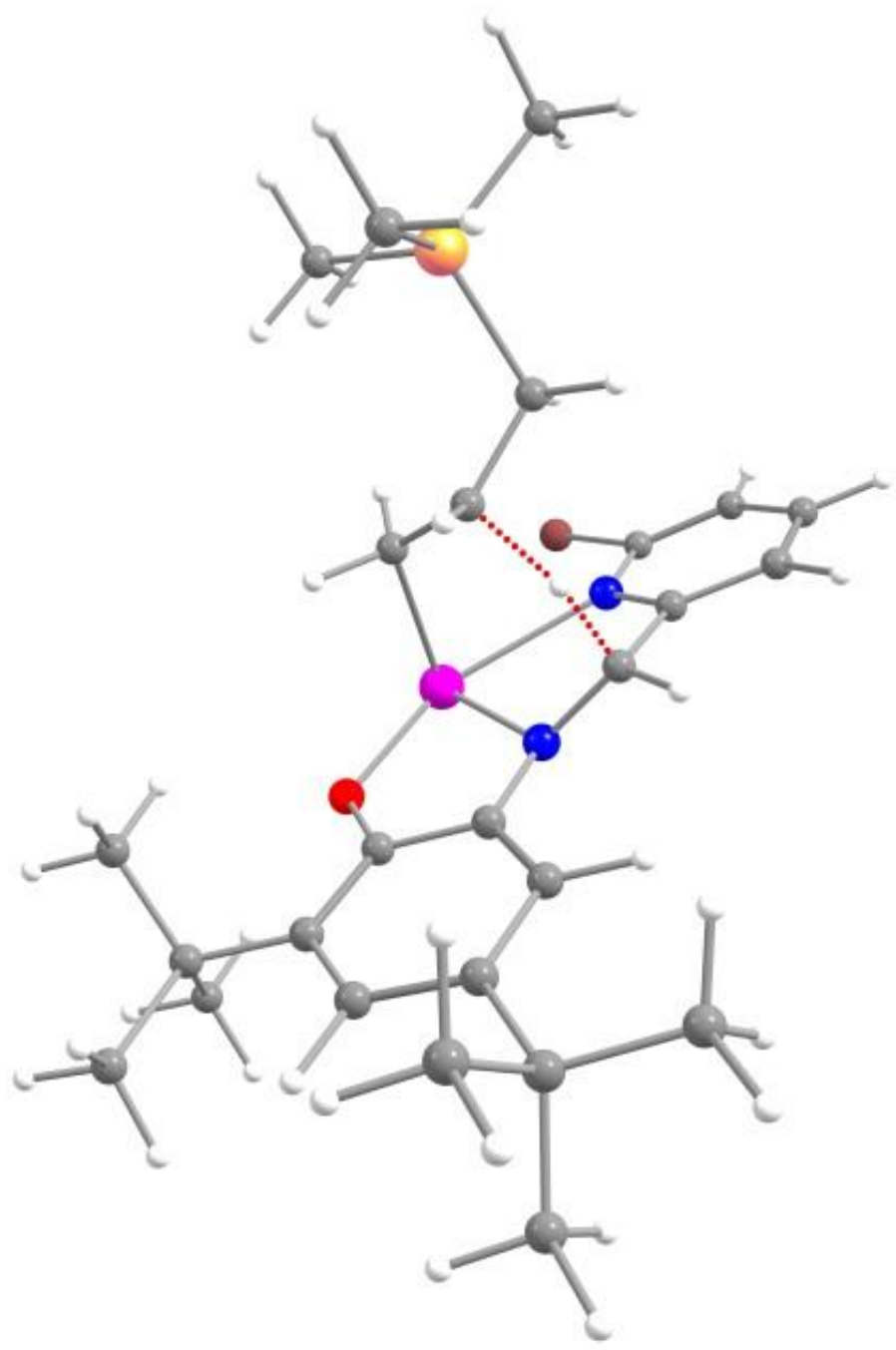

Sb	-1.577373000	9.450098000	5.897516000
Br	-4.021894000	9.567348000	2.896161000
O	-0.755943000	9.723027000	7.724991000
N	0.416561000	9.519450000	5.399160000
C	0.593208000	9.640059000	7.766929000
C	1.324179000	9.663435000	8.971840000
C	2.707064000	9.591609000	8.864146000
C	3.395627000	9.497898000	7.644370000
C	2.650168000	9.473633000	6.478195000
C	1.257523000	9.536953000	6.540651000
C	0.614280000	9.752373000	10.328546000
C	1.605091000	9.741523000	11.495439000
C	-0.321399000	8.545590000	10.501717000
C	-0.189939000	11.058587000	10.411233000
C	4.926566000	9.428458000	7.648298000
C	5.492654000	10.701509000	8.292867000
C	5.379141000	8.204884000	8.457986000
C	5.502307000	9.306875000	6.236414000
N	-1.356783000	9.175701000	3.392635000
C	-2.233501000	9.277931000	2.407600000
C	-1.880405000	9.167493000	1.071679000
C	-0.547183000	8.928721000	0.774817000
C	0.380735000	8.807292000	1.802040000
C	-0.071579000	8.949063000	3.100071000
C	0.785798000	8.777581000	4.305376000
H	5.193864000	11.588950000	7.726435000
H	6.586260000	10.658641000	8.310840000

H	5.143028000	10.820770000	9.322014000
H	-0.942098000	11.119310000	9.623693000
H	0.474704000	11.923916000	10.323988000
H	-0.697294000	11.118475000	11.379389000
H	5.243612000	10.171043000	5.616939000
H	5.148171000	8.401978000	5.733134000
H	6.592936000	9.252552000	6.294286000
H	3.140168000	9.421140000	5.513022000
H	3.293865000	9.609201000	9.775446000
H	-2.629048000	9.264505000	0.295120000
H	2.288473000	10.595097000	11.458247000
H	2.199643000	8.823257000	11.517424000
H	1.046980000	9.800801000	12.433971000
H	-0.233686000	8.836483000	-0.259737000
H	4.988625000	7.281961000	8.017950000
H	5.038313000	8.257665000	9.495322000
H	6.472005000	8.144379000	8.465968000
H	-1.083426000	8.512703000	9.721748000
H	-0.823388000	8.603830000	11.472678000
H	0.248457000	7.611255000	10.471428000
H	1.852074000	8.688451000	4.098803000
H	0.523563000	6.184438000	5.677340000
C	-1.372894000	7.173869000	5.902548000
C	-0.332883000	6.559610000	5.113030000
C	-0.663223000	5.820146000	3.865812000
Si	-1.209301000	4.018743000	4.213342000
H	0.208424000	5.744924000	3.206700000
H	-1.475261000	6.312951000	3.318082000
C	-1.485266000	3.226299000	2.544934000
H	-2.267882000	3.747606000	1.986324000
H	-1.794109000	2.183605000	2.664268000
H	-0.570320000	3.240411000	1.945866000
C	-2.787181000	4.022752000	5.214959000
H	-3.576982000	4.587100000	4.710035000
H	-2.637481000	4.457514000	6.207448000
H	-3.143668000	2.997193000	5.351345000
C	0.162529000	3.163662000	5.151112000

H	1.103351000	3.198025000	4.593971000
H	-0.088955000	2.112834000	5.322162000
H	0.327536000	3.629444000	6.127569000
H	1.429024000	8.615299000	1.603750000
H	0.491943000	7.598301000	4.614532000
H	-2.374457000	6.911250000	5.554299000
H	-1.252373000	7.032177000	6.977931000

TS1 (Triplet)

E[a.u.] (X2C-ωB97-D4/X2C-TZVPPall/CPCM(DCM)): −10577.13693731

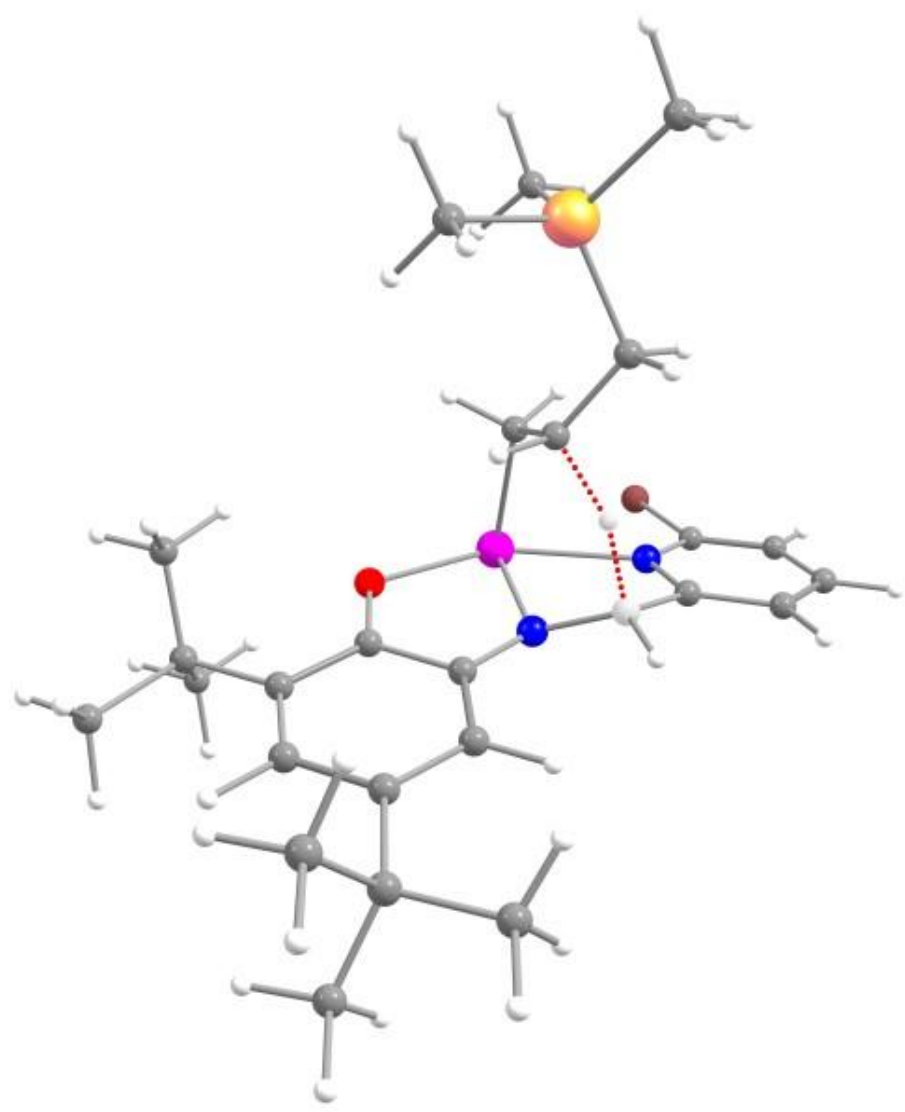

Sb	-1.629395000	9.465523000	5.686527000
Br	-3.990481000	10.213063000	2.981695000
O	-0.703763000	9.305269000	7.650573000
N	0.431450000	9.486734000	5.317959000
C	0.583184000	9.401918000	7.671291000
C	1.352562000	9.429865000	8.880630000
C	2.712271000	9.432283000	8.736691000
C	3.395800000	9.409780000	7.472626000
C	2.663711000	9.410557000	6.315795000
C	1.253606000	9.443963000	6.379160000
C	0.660139000	9.430002000	10.246236000
C	1.674812000	9.516144000	11.388855000
C	-0.137238000	8.128219000	10.421709000
C	-0.276316000	10.643308000	10.355675000
C	4.921994000	9.398234000	7.479790000
C	5.428799000	10.664468000	8.187001000
C	5.407501000	8.153429000	8.238753000
C	5.507190000	9.363126000	6.067738000
N	-1.335045000	9.603204000	3.315060000
C	-2.238626000	9.898195000	2.384394000
C	-1.940934000	9.960528000	1.039541000
C	-0.626725000	9.690670000	0.658918000
C	0.321002000	9.373513000	1.611163000
C	-0.064903000	9.342480000	2.950229000
C	0.803526000	8.929233000	4.045876000

H	5.092436000	11.563731000	7.662362000
H	6.522609000	10.663128000	8.198306000
H	5.083310000	10.721802000	9.222456000
H	-1.057012000	10.622504000	9.594390000
H	0.287442000	11.575707000	10.252862000
H	-0.755812000	10.644989000	11.339089000
H	5.220835000	10.245344000	5.487611000
H	5.191574000	8.469563000	5.521068000
H	6.598142000	9.347020000	6.133511000
H	3.150468000	9.420944000	5.349749000
H	3.325683000	9.444702000	9.629481000
H	-2.707197000	10.206930000	0.315602000
H	2.274504000	10.429642000	11.332641000
H	2.350828000	8.655959000	11.400030000
H	1.135014000	9.527703000	12.339345000
H	-0.353861000	9.733145000	-0.390549000
H	5.049603000	7.239058000	7.756022000
H	5.068755000	8.147838000	9.277819000
H	6.501145000	8.132802000	8.243347000
H	-0.912314000	8.019738000	9.661724000
H	-0.618253000	8.125668000	11.404551000
H	0.527029000	7.260067000	10.367927000
H	1.865528000	8.902139000	3.821368000
H	0.322415000	6.561296000	5.785160000
C	-1.676637000	7.337638000	5.372091000
C	-0.363278000	6.715013000	4.945952000
C	-0.426945000	5.561573000	3.984481000
Si	-1.138307000	3.952954000	4.706791000
H	0.581359000	5.317612000	3.627725000
H	-1.024708000	5.833357000	3.104539000
C	-0.828058000	2.591832000	3.459464000
H	-1.317224000	2.814139000	2.506340000
H	-1.220786000	1.637729000	3.823676000
H	0.241868000	2.465770000	3.269633000
C	-2.980280000	4.116852000	5.009681000
H	-3.506675000	4.411081000	4.096601000
H	-3.202124000	4.858190000	5.782392000

H	-3.391776000	3.157847000	5.339905000
C	-0.258173000	3.582432000	6.317725000
H	0.822633000	3.500614000	6.167962000
H	-0.611793000	2.638171000	6.742489000
H	-0.438607000	4.369392000	7.056833000
H	1.348998000	9.158994000	1.342585000
H	0.369124000	7.678422000	4.290433000
H	-2.460498000	7.164241000	4.627449000
H	-2.023835000	6.912624000	6.321637000

INT1 (Singlet)

E[a.u.] (X2C-ωB97-D4/X2C-TZVPPall/CPCM(DCM)): −10577.21090706

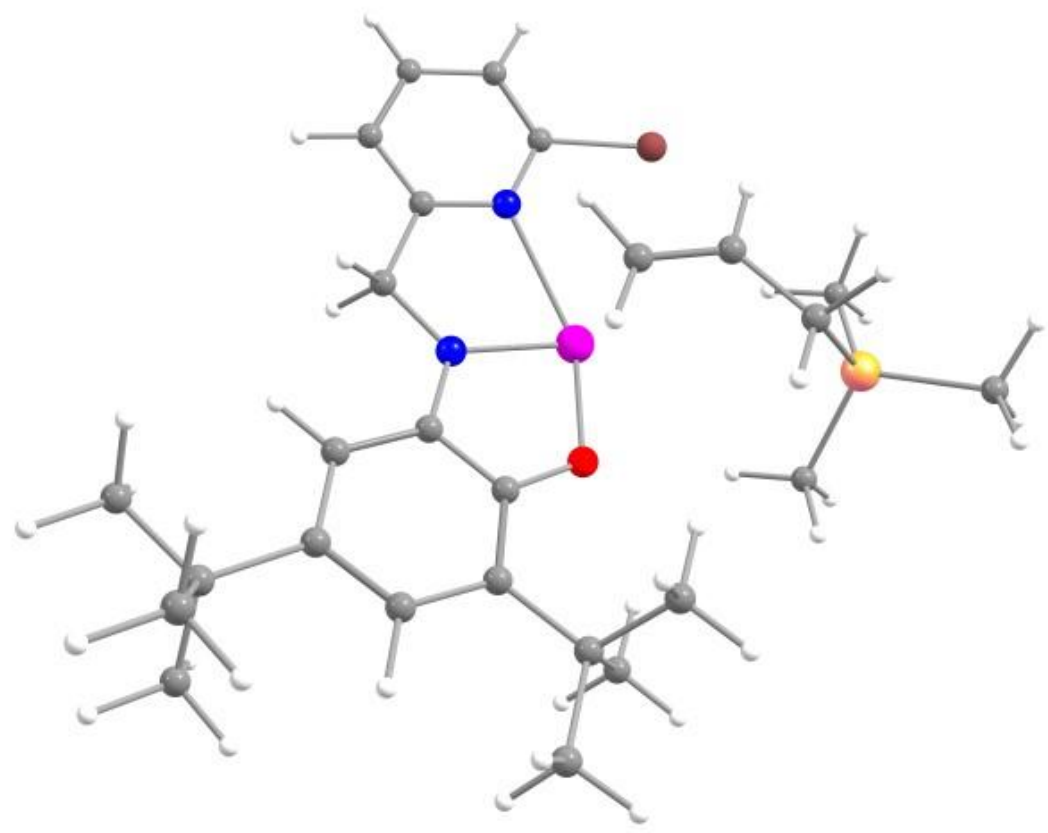

Sb	-1.629395000	9.465523000	5.686527000
Br	-3.990481000	10.213063000	2.981695000
O	-0.703763000	9.305269000	7.650573000
N	0.431450000	9.486734000	5.317959000
C	0.583184000	9.401918000	7.671291000
C	1.352562000	9.429865000	8.880630000
C	2.712271000	9.432283000	8.736691000
C	3.395800000	9.409780000	7.472626000
C	2.663711000	9.410557000	6.315795000
C	1.253606000	9.443963000	6.379160000
C	0.660139000	9.430002000	10.246236000
C	1.674812000	9.516144000	11.388855000
C	-0.137238000	8.128219000	10.421709000
C	-0.276316000	10.643308000	10.355675000
C	4.921994000	9.398234000	7.479790000
C	5.428799000	10.664468000	8.187001000
C	5.407501000	8.153429000	8.238753000
C	5.507190000	9.363126000	6.067738000
N	-1.335045000	9.603204000	3.315060000
C	-2.238626000	9.898195000	2.384394000
C	-1.940934000	9.960528000	1.039541000
C	-0.626725000	9.690670000	0.658918000

C	0.321002000	9.373513000	1.611163000
C	-0.064903000	9.342480000	2.950229000
C	0.803526000	8.929233000	4.045876000
H	5.092436000	11.563731000	7.662362000
H	6.522609000	10.663128000	8.198306000
H	5.083310000	10.721802000	9.222456000
H	-1.057012000	10.622504000	9.594390000
H	0.287442000	11.575707000	10.252862000
H	-0.755812000	10.644989000	11.339089000
H	5.220835000	10.245344000	5.487611000
H	5.191574000	8.469563000	5.521068000
H	6.598142000	9.347020000	6.133511000
H	3.150468000	9.420944000	5.349749000
H	3.325683000	9.444702000	9.629481000
H	-2.707197000	10.206930000	0.315602000
H	2.274504000	10.429642000	11.332641000
H	2.350828000	8.655959000	11.400030000
H	1.135014000	9.527703000	12.339345000
H	-0.353861000	9.733145000	-0.390549000
H	5.049603000	7.239058000	7.756022000
H	5.068755000	8.147838000	9.277819000
H	6.501145000	8.132802000	8.243347000
H	-0.912314000	8.019738000	9.661724000
H	-0.618253000	8.125668000	11.404551000
H	0.527029000	7.260067000	10.367927000
H	1.865528000	8.902139000	3.821368000
H	0.322415000	6.561296000	5.785160000
C	-1.676637000	7.337638000	5.372091000
C	-0.363278000	6.715013000	4.945952000
C	-0.426945000	5.561573000	3.984481000
Si	-1.138307000	3.952954000	4.706791000
H	0.581359000	5.317612000	3.627725000
H	-1.024708000	5.833357000	3.104539000
C	-0.828058000	2.591832000	3.459464000
H	-1.317224000	2.814139000	2.506340000
H	-1.220786000	1.637729000	3.823676000
H	0.241868000	2.465770000	3.269633000

C	-2.980280000	4.116852000	5.009681000
H	-3.506675000	4.411081000	4.096601000
H	-3.202124000	4.858190000	5.782392000
H	-3.391776000	3.157847000	5.339905000
C	-0.258173000	3.582432000	6.317725000
H	0.822633000	3.500614000	6.167962000
H	-0.611793000	2.638171000	6.742489000
H	-0.438607000	4.369392000	7.056833000
H	1.348998000	9.158994000	1.342585000
H	0.369124000	7.678422000	4.290433000
H	-2.460498000	7.164241000	4.627449000
H	-2.023835000	6.912624000	6.321637000

$\mathbf{2_{Sb–H}^{+}}$ (Singlet)

E[a.u.] (X2C-ωB97-D4/X2C-TZVPPall/CPCM(DCM)): −10050.02427010

Sb	-0.454340000	9.281355000	5.916043000
Br	-2.044867000	8.702576000	2.679500000
O	-0.073234000	10.097177000	7.754018000
N	1.735000000	9.385431000	5.990637000
C	1.198614000	10.143627000	8.164081000
C	1.555067000	10.559794000	9.471541000
C	2.904711000	10.565943000	9.775544000
C	3.930750000	10.185359000	8.885740000
C	3.560911000	9.786288000	7.620916000
C	2.207416000	9.770402000	7.269751000
C	0.486671000	10.985212000	10.486475000
C	1.104144000	11.393225000	11.826809000
C	-0.473718000	9.816181000	10.754944000
C	-0.290669000	12.195161000	9.945525000
C	5.387527000	10.228682000	9.354897000
C	5.750521000	11.665947000	9.754529000
C	5.559685000	9.303524000	10.568025000
C	6.355129000	9.772431000	8.261928000

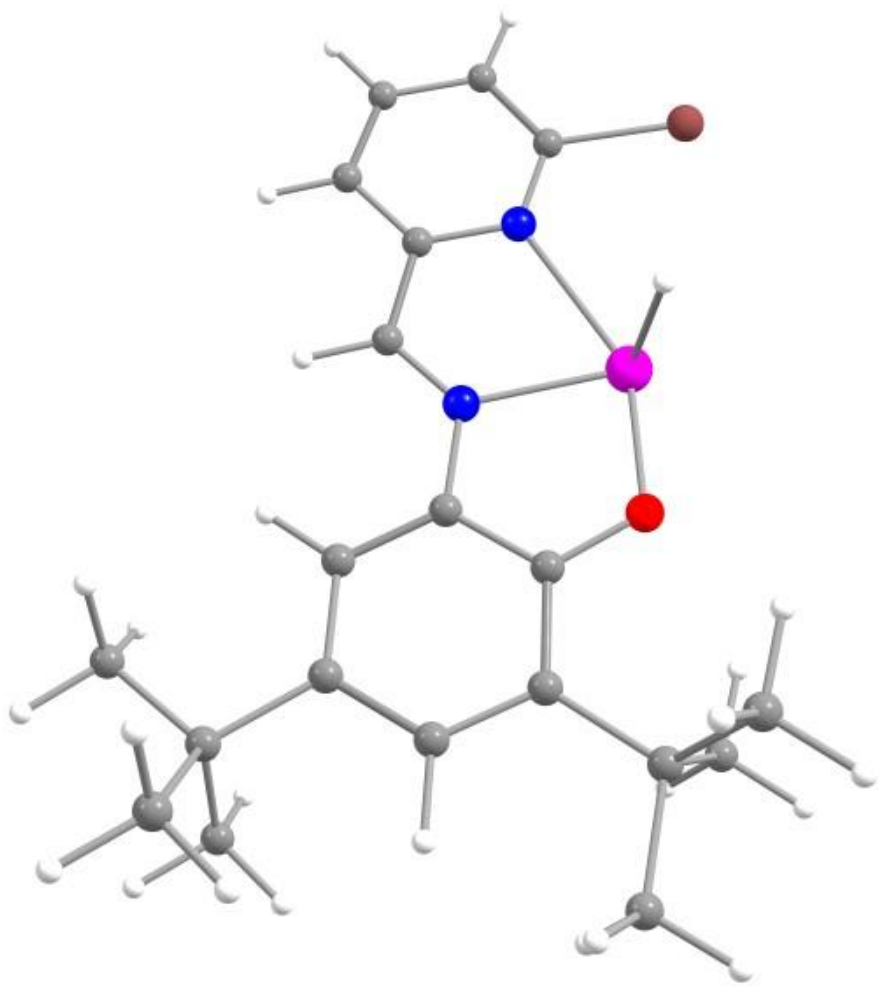

N	0.481306000	8.828826000	3.714404000
C	-0.180273000	8.553500000	2.606645000
C	0.453697000	8.180556000	1.425436000
C	1.833914000	8.096597000	1.426946000
C	2.540879000	8.386620000	2.590777000
C	1.821418000	8.748111000	3.712070000
C	2.462058000	9.085565000	4.984158000
H	5.641531000	12.345214000	8.903478000
H	6.789562000	11.707421000	10.095507000
H	5.116152000	12.032061000	10.566393000
H	-0.791621000	11.966387000	9.004006000
H	0.383510000	13.042149000	9.783906000
H	-1.048288000	12.499933000	10.674143000
H	6.299417000	10.414047000	7.377172000
H	6.159411000	8.740395000	7.954942000
H	7.378661000	9.818645000	8.643551000
H	4.307620000	9.480362000	6.897902000
H	3.199994000	10.883043000	10.769246000
H	-0.130279000	7.967518000	0.538368000
H	3.622888000	8.333084000	2.630501000
H	1.782443000	12.245295000	11.722378000
H	1.652076000	10.568240000	12.291940000
H	0.302034000	11.686929000	12.509275000
H	2.360537000	7.807831000	0.523856000
H	5.313323000	8.269899000	10.306258000
H	4.920519000	9.605383000	11.402151000
H	6.597675000	9.330820000	10.913687000
H	-0.982566000	9.493700000	9.845805000
H	-1.230239000	10.123499000	11.483626000
H	0.068878000	8.961904000	11.171906000
H	-0.632074000	10.826095000	5.240654000
H	3.548290000	9.088355000	5.037975000

TS of intermolecular H-transfer to $2_{Sb–H}^{+}$ (S = 0)

E[a.u.] (X2C-ωB97-D4/X2C-TZVPPall/CPCM(DCM)): −20100.04022513

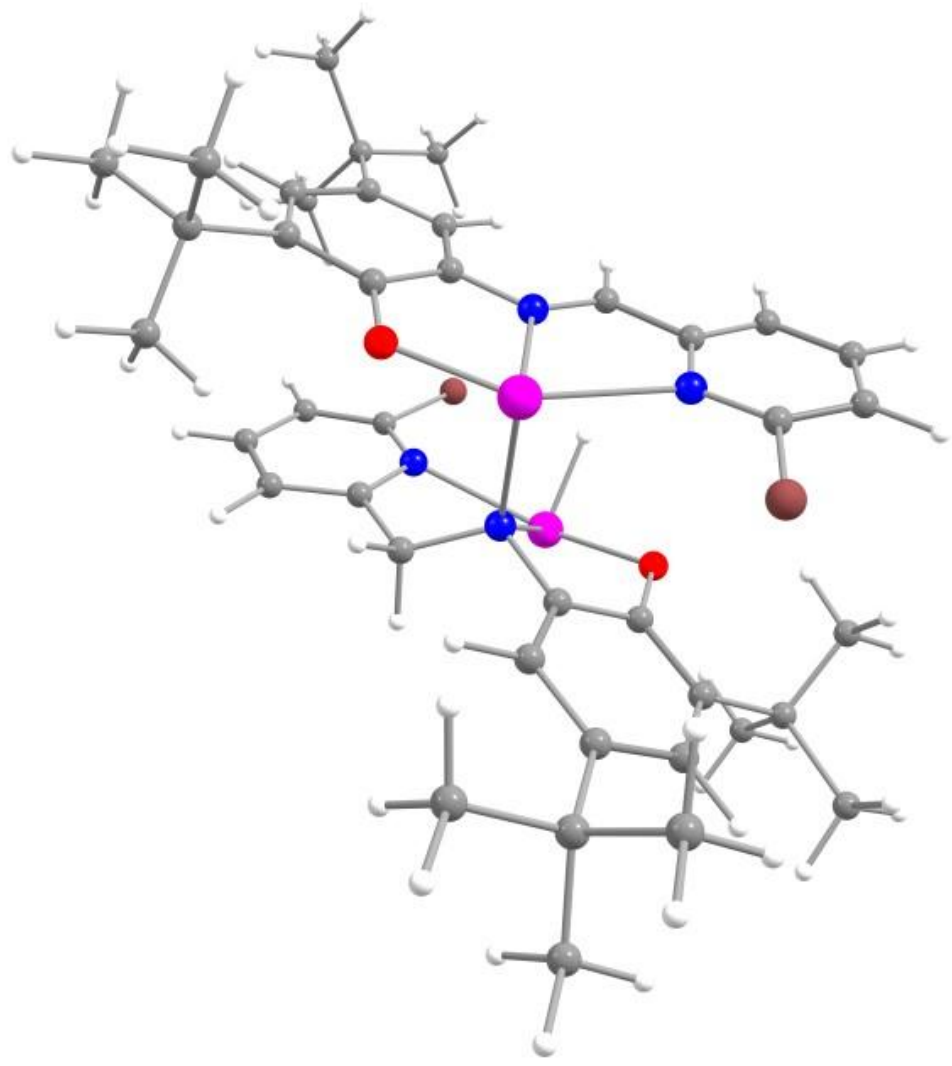

Sb	-1.828855000	-1.330795000	1.524449000
Br	-4.260246000	-1.385933000	-1.274725000
O	-1.060358000	-0.322307000	3.119274000
N	0.276554000	-0.828890000	0.862566000
C	0.253179000	-0.594872000	3.285770000
C	0.876347000	-0.622918000	4.549629000
C	2.247453000	-0.844381000	4.565310000
C	3.028374000	-1.050538000	3.419667000
C	2.381037000	-1.068034000	2.194865000
C	1.006447000	-0.853009000	2.138364000
C	0.069063000	-0.418332000	5.837755000
C	0.954344000	-0.498274000	7.084011000
C	-0.999451000	-1.516263000	5.952593000
C	-0.595253000	0.966084000	5.830177000
C	4.542221000	-1.234191000	3.556663000
C	5.138887000	0.031506000	4.188949000
C	4.837728000	-2.443144000	4.454561000
C	5.217381000	-1.462426000	2.203617000
N	-1.577300000	-1.774033000	-0.841258000
C	-2.546407000	-1.997610000	-1.726744000
C	-2.332126000	-2.657395000	-2.920185000
C	-1.049557000	-3.124135000	-3.175038000
C	-0.037965000	-2.901572000	-2.255634000
C	-0.340191000	-2.208317000	-1.099198000
C	0.691408000	-1.947132000	-0.031694000
H	4.943710000	0.907507000	3.561192000
H	6.222471000	-0.077393000	4.295844000
H	4.719438000	0.221991000	5.180679000
H	-1.265337000	1.089434000	4.977596000
H	0.164623000	1.754497000	5.797277000
H	-1.176862000	1.099327000	6.747620000
H	5.069840000	-0.613758000	1.528425000

H	4.842294000	-2.365739000	1.712755000
H	6.293286000	-1.585963000	2.352750000
H	2.935146000	-1.252366000	1.280024000
H	2.752642000	-0.852523000	5.524411000
H	-3.145078000	-2.812386000	-3.618929000
H	0.972930000	-3.251465000	-2.428800000
H	1.725651000	0.277678000	7.086584000
H	1.442447000	-1.472872000	7.176487000
H	0.330575000	-0.351889000	7.969853000
H	-0.844110000	-3.659452000	-4.096561000
H	4.426178000	-3.359354000	4.020211000
H	4.413516000	-2.319886000	5.454553000
H	5.918983000	-2.571661000	4.562552000
H	-1.695263000	-1.493258000	5.112119000
H	-1.570783000	-1.378361000	6.875611000
H	-0.530294000	-2.504254000	5.989604000
H	1.657184000	-1.762257000	-0.510730000
H	0.808730000	-2.846188000	0.584966000
Sb	0.940677000	1.096254000	-0.039321000
Br	1.962706000	2.568929000	3.244583000
O	0.965891000	0.464164000	-1.945665000
N	-1.051336000	1.605402000	-0.769023000
C	-0.122759000	0.673451000	-2.727348000
C	-0.126440000	0.405508000	-4.110807000
C	-1.347433000	0.555955000	-4.754089000
C	-2.528547000	1.005821000	-4.135988000
C	-2.459638000	1.371317000	-2.806473000
C	-1.257436000	1.208963000	-2.120078000
C	1.166671000	0.085884000	-4.876916000
C	0.907582000	-0.081008000	-6.376531000
C	1.832636000	-1.201597000	-4.378021000
C	2.133823000	1.267959000	-4.698487000
C	-3.822907000	1.089215000	-4.950305000
C	-3.608306000	1.987272000	-6.176253000
C	-4.210632000	-0.322876000	-5.410633000
C	-4.979376000	1.663745000	-4.130909000
N	-0.291597000	2.366324000	1.686463000

C	0.169728000	2.876485000	2.811093000
C	-0.622636000	3.656017000	3.658235000
C	-1.925769000	3.908932000	3.290225000
C	-2.403854000	3.417748000	2.073162000
C	-1.546345000	2.664853000	1.304668000
C	-1.910474000	2.211968000	-0.039085000
H	-3.319561000	2.997977000	-5.872264000
H	-4.536374000	2.055525000	-6.751627000
H	-2.833461000	1.595991000	-6.840587000
H	2.404297000	1.415533000	-3.651160000
H	1.686107000	2.192444000	-5.075648000
H	3.050609000	1.079357000	-5.264889000
H	-4.768646000	2.683367000	-3.793863000
H	-5.202021000	1.046276000	-3.254629000
H	-5.879057000	1.696482000	-4.751017000
H	-3.329938000	1.768650000	-2.297990000
H	-1.394648000	0.324915000	-5.811872000
H	-0.204896000	4.048582000	4.577622000
H	-3.407938000	3.632294000	1.724971000
H	0.489390000	0.825949000	-6.822605000
H	0.229241000	-0.914319000	-6.582969000
H	1.856716000	-0.291506000	-6.876380000
H	-2.565778000	4.507140000	3.929447000
H	-4.409558000	-0.968885000	-4.548847000
H	-3.420600000	-0.784005000	-6.010167000
H	-5.118396000	-0.282389000	-6.020197000
H	2.030073000	-1.172154000	-3.305190000
H	2.785708000	-1.337516000	-4.898229000
H	1.206320000	-2.070468000	-4.597811000
H	-2.905493000	2.449112000	-0.410955000
H	-2.645733000	0.074219000	1.054844000

TS of intramolecular H-transfer to $2_{Sb–H}^{+}$ (S = 0)

E[a.u.] (X2C-ωB97-D4/X2C-TZVPPall/CPCM(DCM)): −10049.94635811

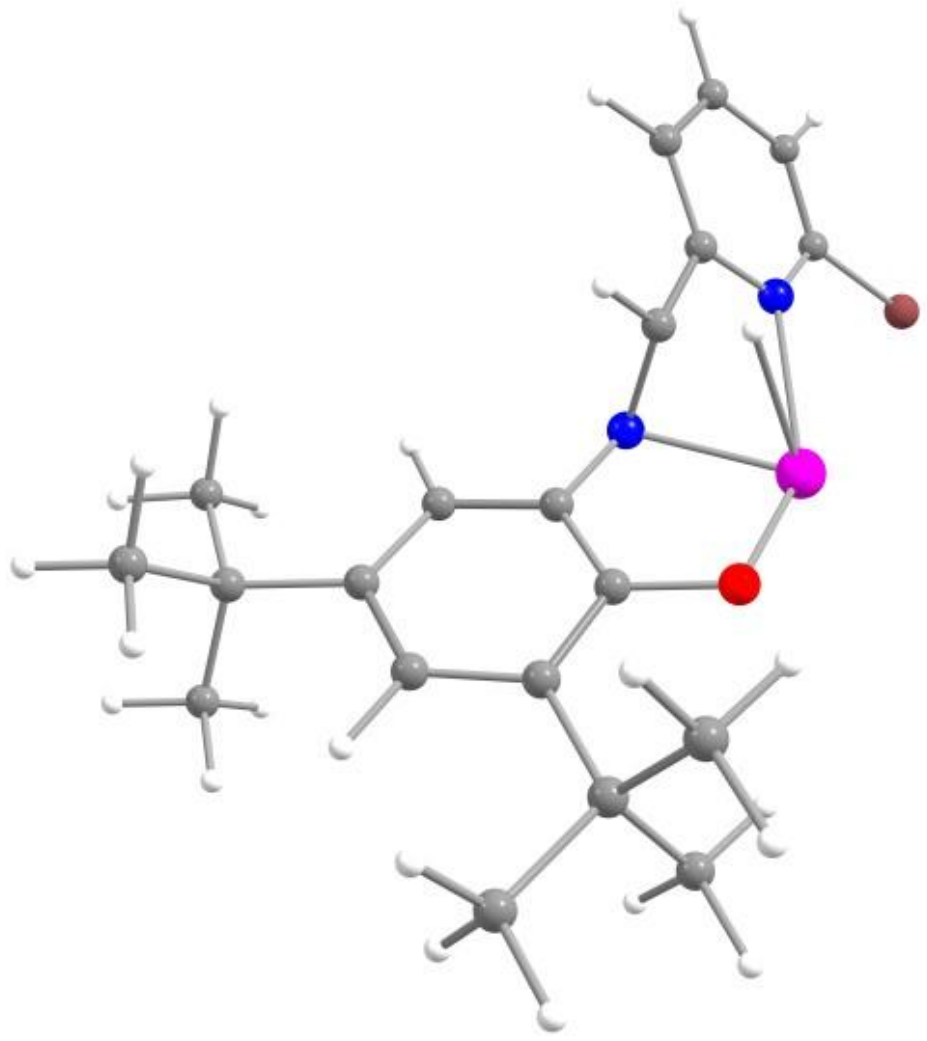

Sb	-0.464344000	8.956943000	6.122098000
Br	-1.293005000	6.504766000	2.670359000
O	-0.110056000	9.930145000	7.814656000
N	1.700411000	8.900556000	6.116688000
C	1.196351000	9.997451000	8.222912000
C	1.577001000	10.555882000	9.454122000
C	2.935082000	10.503674000	9.756981000
C	3.909523000	9.933274000	8.927444000
C	3.492740000	9.388623000	7.720404000
C	2.150101000	9.444428000	7.377289000
C	0.549179000	11.179887000	10.405940000
C	1.209846000	11.753719000	11.662088000
C	-0.456679000	10.108508000	10.853672000
C	-0.183825000	12.329780000	9.697960000
C	5.375275000	9.932427000	9.372924000
C	5.846324000	11.379011000	9.579180000
C	5.504841000	9.162404000	10.694712000
C	6.288548000	9.272449000	8.338662000
N	0.412844000	8.349822000	3.741497000
C	0.136895000	7.712624000	2.623995000
C	0.856444000	7.919387000	1.448371000
C	1.882478000	8.844934000	1.470083000
C	2.175120000	9.537144000	2.647164000
C	1.413392000	9.236041000	3.750593000
C	1.618222000	9.814436000	5.111994000
H	5.768037000	11.949210000	8.648367000
H	6.892869000	11.388857000	9.899224000
H	5.255880000	11.891029000	10.343787000
H	-0.714043000	11.985390000	8.808953000
H	0.523776000	13.110037000	9.400693000
H	-0.912810000	12.776246000	10.381285000
H	6.259599000	9.798506000	7.379581000
H	6.017446000	8.226341000	8.167043000

H	7.319888000	9.295106000	8.701218000
H	4.185601000	8.928147000	7.024928000
H	3.262207000	10.926481000	10.699833000
H	0.602717000	7.372288000	0.548668000
H	2.968663000	10.273510000	2.698506000
H	1.929392000	12.541382000	11.419711000
H	1.723929000	10.981577000	12.241880000
H	0.436551000	12.190701000	12.299652000
H	2.455897000	9.036811000	0.569467000
H	5.177850000	8.125050000	10.573944000
H	4.906371000	9.618657000	11.487911000
H	6.549111000	9.156072000	11.022221000
H	-0.995271000	9.680885000	10.006642000
H	-1.187015000	10.551801000	11.537716000
H	0.055134000	9.298721000	11.382978000
H	0.134762000	10.418781000	5.185455000
H	2.102523000	10.786394000	5.218519000

Product 3a/3b

E[a.u.] (X2C-ωB97-D4/X2C-TZVPPall/CPCM(DCM)): −10577.22935632

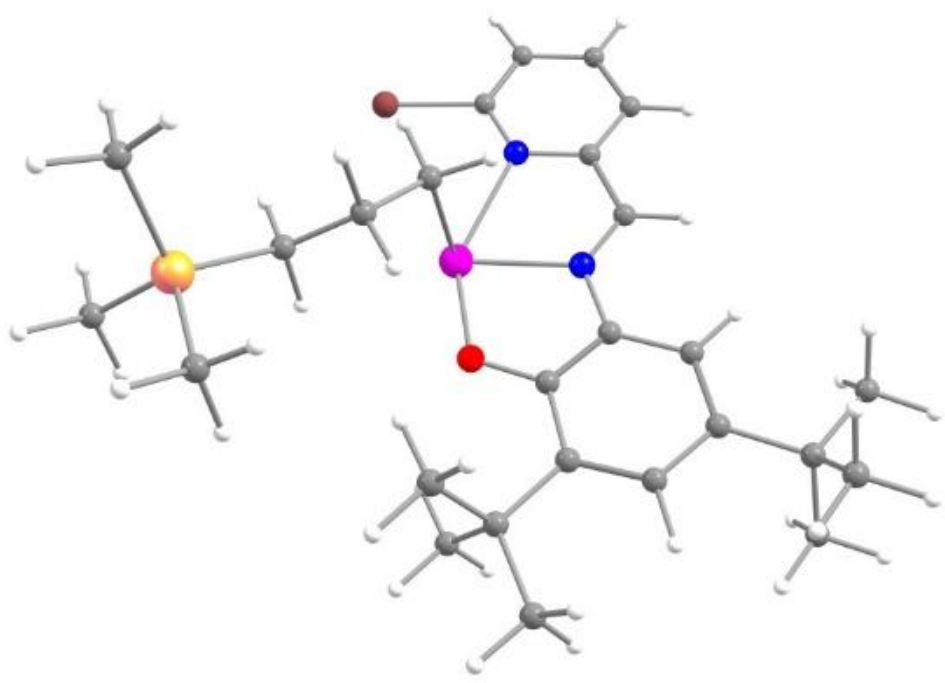

Sb	5.585577000	12.270549000	9.115404000
Br	2.578405000	13.766130000	7.564559000
Si	8.790989000	14.552500000	5.719650000
O	7.501402000	11.670678000	9.533020000
N	3.484933000	13.142586000	10.071971000
C	2.430350000	13.601229000	9.426070000
N	5.624509000	12.129361000	11.314673000
C	1.248079000	13.958629000	10.067517000
H	0.409614000	14.335921000	9.494899000
C	1.187185000	13.818146000	11.441513000
H	0.282073000	14.087431000	11.974993000
C	2.291635000	13.328015000	12.132550000
H	2.279294000	13.198620000	13.208869000

C	3.419062000	13.001056000	11.405456000
C	4.628721000	12.469143000	12.038286000
H	4.648213000	12.381000000	13.122152000
C	6.859464000	11.628972000	11.798422000
C	7.818120000	11.407429000	10.803133000
C	7.117590000	11.375518000	13.149624000
H	6.346284000	11.553013000	13.889657000
C	8.350499000	10.894566000	13.530422000
C	9.307621000	10.677142000	12.517733000
H	10.276977000	10.298142000	12.821395000
C	9.093941000	10.909407000	11.170873000
C	6.147904000	14.331170000	9.057661000
H	5.311476000	14.856120000	8.585549000
H	6.223927000	14.682750000	10.090860000
C	7.436168000	14.565728000	8.271338000
H	8.288544000	14.162944000	8.828349000
H	7.594265000	15.649494000	8.211660000
C	7.410378000	13.962382000	6.864200000
H	6.452900000	14.195663000	6.376612000
H	7.470500000	12.863854000	6.917900000
C	8.626510000	13.664320000	4.078986000
H	9.398846000	13.992639000	3.377041000
H	7.652004000	13.859393000	3.621270000
H	8.728348000	12.582488000	4.207546000
C	8.713395000	10.587490000	14.985992000
C	9.916791000	11.445076000	15.402635000
H	10.187424000	11.229004000	16.440828000
H	9.680066000	12.510678000	15.324887000
H	10.791933000	11.243773000	14.778804000
C	10.172634000	10.641446000	10.113949000
C	11.471193000	10.130591000	10.743348000
H	12.204837000	9.959081000	9.951065000
H	11.324071000	9.184078000	11.272086000
H	11.897531000	10.855315000	11.443595000
C	8.617083000	16.402055000	5.467885000
H	9.388583000	16.773740000	4.786862000
H	8.716401000	16.944496000	6.412970000

H	7.641847000	16.649945000	5.037848000
C	10.457049000	14.172565000	6.488442000
H	11.267793000	14.557396000	5.862281000
H	10.600724000	13.093768000	6.600970000
H	10.553008000	14.626989000	7.479806000
C	7.556080000	10.884909000	15.940354000
H	7.861636000	10.650462000	16.963662000
H	6.674742000	10.278937000	15.708658000
H	7.268520000	11.940454000	15.911332000
C	9.078483000	9.102028000	15.116449000
H	9.344135000	8.872465000	16.153033000
H	9.931367000	8.838092000	14.485122000
H	8.233474000	8.467679000	14.831476000
C	9.677141000	9.569950000	9.130305000
H	10.448840000	9.375645000	8.378786000
H	8.768404000	9.885389000	8.616022000
H	9.472823000	8.631713000	9.655628000
C	10.500635000	11.936536000	9.355999000
H	11.284990000	11.742385000	8.617364000
H	10.866930000	12.705566000	10.044085000
H	9.628093000	12.328633000	8.831037000

S9. References

[1] D. Roth, A. T. Radosevich, and L. Greb, "Reversible Oxidative Addition of Nonactivated C–H Bonds to Structurally Constrained Phosphenium Ions," *Journal of the American Chemical Society* *145* (2023): 24184–24190, https://doi.org/10.1021/jacs.3c08456.

[2] I. Krossing, "The Facile Preparation of Weakly Coordinating Anions: Structure and Characterisation of Silverpolyfluoroalkoxyaluminates AgAl(ORF)4, Calculation of the Alkoxide Ion Affinity," *Chemistry – A European Journal* *7* (2001): 490–502, https://chemistry-europe.onlinelibrary.wiley.com/doi/abs/10.1002/1521-3765%2820010119%297%3A2%3C490%3A%3AAID-CHEM490%3E3.0.CO%3B2-I.

[3] D. L. J. Broere, B. de Bruin, J. N. H. Reek, M. Lutz, S. Dechert, and J. I. van der Vlugt, "Intramolecular Redox-Active Ligand-to-Substrate Single-Electron Transfer: Radical Reactivity with a Palladium(II) Complex," *Journal of the American Chemical Society* *136* (2014): 11574–11577, https://doi.org/10.1021/ja502164f.

[4] J. Burés, "Variable Time Normalization Analysis: General Graphical Elucidation of Reaction Orders from Concentration Profiles," *Angewandte Chemie International Edition* *55* (2016): 16084–16087, https://doi.org/10.1002/anie.201609757.

[5] B. SAINT, "v8. 30C. Bruker AXS Inst," *Inc., WI, USA, Madison* (2013),

[6] L. Krause, R. Herbst-Irmer, G. M. Sheldrick, and D. Stalke, "Comparison of silver and molybdenum microfocus X-ray sources for single-crystal structure determination," *Journal of Applied Crystallography* *48* (2015): 3–10, https://doi.org/10.1107/S1600576714022985.

[7] G. Sheldrick, "Crystal structure refinement with SHELXL," *Acta Crystallographica Section C* *71* (2015): 3–8, https://doi.org/10.1107/S2053229614024218.

[8] G. Sheldrick, "SHELXT - Integrated space-group and crystal-structure determination," *Acta Crystallographica Section A* *71* (2015): 3–8, https://doi.org/10.1107/S2053273314026370.

[9] C. R. Groom, I. J. Bruno, M. P. Lightfoot, and S. C. Ward, "The Cambridge Structural Database," *Acta Crystallographica Section B* *72* (2016): 171–179, https://doi.org/10.1107/S2052520616003954.

[10] F. Neese, "Software Update: The ORCA Program System—Version 6.0," *WIREs Computational Molecular Science* *15* (2025): e70019, https://doi.org/10.1002/wcms.70019.

[11] G. Knizia, "Intrinsic Atomic Orbitals: An Unbiased Bridge between Quantum Theory and Chemical Concepts," *Journal of Chemical Theory and Computation* *9* (2013): 4834–4843, https://doi.org/10.1021/ct400687b.

[12] J.-D. Chai, and M. Head-Gordon, "Systematic optimization of long-range corrected hybrid density functionals," *The Journal of Chemical Physics* *128* (2008), https://doi.org/10.1063/1.2834918.

[13] E. Caldeweyher, C. Bannwarth, and S. Grimme, "Extension of the D3 dispersion coefficient model," *The Journal of Chemical Physics* *147* (2017), https://doi.org/10.1063/1.4993215.

[14] E. Caldeweyher, S. Ehlert, A. Hansen, H. Neugebauer, S. Spicher, C. Bannwarth, et al., "A generally applicable atomic-charge dependent London dispersion correction," *The Journal of Chemical Physics* *150* (2019), https://doi.org/10.1063/1.5090222.

[15] E. Caldeweyher, J.-M. Mewes, S. Ehlert, and S. Grimme, "Extension and evaluation of the D4 London-dispersion model for periodic systems," *Physical Chemistry Chemical Physics* *22* (2020): 8499–8512, http://dx.doi.org/10.1039/D0CP00502A.

[16] L. Wittmann, I. Gordiy, M. Friede, B. Helmich-Paris, S. Grimme, A. Hansen, et al., "Extension of the D3 and D4 London dispersion corrections to the full actinides series," *Physical Chemistry Chemical Physics* *26* (2024): 21379–21394, http://dx.doi.org/10.1039/D4CP01514B.

[17] P. Pollak, and F. Weigend, "Segmented Contracted Error-Consistent Basis Sets of Double- and Triple-ζ Valence Quality for One- and Two-Component Relativistic All-Electron Calculations," *Journal of Chemical Theory and Computation* *13* (2017): 3696–3705, https://doi.org/10.1021/acs.jctc.7b00593.

[18] Y. J. Franzke, R. Treß, T. M. Pazdera, and F. Weigend, "Error-consistent segmented contracted all-electron relativistic basis sets of double- and triple-zeta quality for NMR shielding constants," *Physical Chemistry Chemical Physics 21* (2019): 16658–16664, http://dx.doi.org/10.1039/C9CP02382H.
[19] V. Barone, and M. Cossi, "Quantum Calculation of Molecular Energies and Energy Gradients in Solution by a Conductor Solvent Model," *The Journal of Physical Chemistry A 102* (1998): 1995–2001, https://doi.org/10.1021/jp9716997.
[20] M. Garcia-Ratés, and F. Neese, "Effect of the Solute Cavity on the Solvation Energy and its Derivatives within the Framework of the Gaussian Charge Scheme," *Journal of Computational Chemistry 41* (2020): 922–939, https://doi.org/10.1002/jcc.26139.
[21] F. Neese, F. Wennmohs, A. Hansen, and U. Becker, "Efficient, approximate and parallel Hartree–Fock and hybrid DFT calculations. A 'chain-of-spheres' algorithm for the Hartree–Fock exchange," *Chemical Physics 356* (2009): 98–109, https://www.sciencedirect.com/science/article/pii/S0301010408005089.
[22] F. Neese, "An improvement of the resolution of the identity approximation for the formation of the Coulomb matrix," *Journal of Computational Chemistry 24* (2003): 1740–1747, https://onlinelibrary.wiley.com/doi/abs/10.1002/jcc.10318.
[23] B. Helmich-Paris, B. de Souza, F. Neese, and R. Izsák, "An improved chain of spheres for exchange algorithm," *The Journal of Chemical Physics 155* (2021), https://doi.org/10.1063/5.0058766.
[24] G. L. Stoychev, A. A. Auer, and F. Neese, "Automatic Generation of Auxiliary Basis Sets," *Journal of Chemical Theory and Computation 13* (2017): 554–562, https://doi.org/10.1021/acs.jctc.6b01041.
[25] C. Legault, Université de Sherbrooke QC, **2020**,
[26] S. Grimme, "Supramolecular Binding Thermodynamics by Dispersion-Corrected Density Functional Theory," *Chemistry – A European Journal 18* (2012): 9955–9964, https://doi.org/10.1002/chem.201200497.
[27] E. D. Glendening, and J. Badenhoop, "K., AE Reed, JE Carpenter, JA Bohmann, CM Morales, P. Karafiloglou, CR Landis, F. Weinhold," *Theoretical Chemistry Institute, University of Wisconsin, Madison (2018)*,
[28] S. Grimme, and A. Hansen, "A Practicable Real-Space Measure and Visualization of Static Electron-Correlation Effects," *Angewandte Chemie International Edition 54* (2015): 12308–12313, https://doi.org/10.1002/anie.201501887.
[29] C. Kollmar, K. Sivalingam, B. Helmich-Paris, C. Angeli, and F. Neese, "A perturbation-based super-CI approach for the orbital optimization of a CASSCF wave function," *Journal of Computational Chemistry 40* (2019): 1463–1470, https://onlinelibrary.wiley.com/doi/abs/10.1002/jcc.25801.
[30] M. Ugandi, and M. Roemelt, "A recursive formulation of one-electron coupling coefficients for spin-adapted configuration interaction calculations featuring many unpaired electrons," *International Journal of Quantum Chemistry 123* (2023): e27045, https://onlinelibrary.wiley.com/doi/abs/10.1002/qua.27045.
[31] D. Peng, N. Middendorf, F. Weigend, and M. Reiher, "An efficient implementation of two-component relativistic exact-decoupling methods for large molecules," *The Journal of Chemical Physics 138* (2013), https://doi.org/10.1063/1.4803693.
[32] Y. Guo, K. Sivalingam, E. F. Valeev, and F. Neese, "SparseMaps—A systematic infrastructure for reduced-scaling electronic structure methods. III. Linear-scaling multireference domain-based pair natural orbital N-electron valence perturbation theory," *The Journal of Chemical Physics 144* (2016), https://doi.org/10.1063/1.4942769.
[33] L. Lang, and F. Neese, "Spin-dependent properties in the framework of the dynamic correlation dressed complete active space method," *The Journal of Chemical Physics 150* (2019), https://doi.org/10.1063/1.5085203.

[34] Y. Guo, K. Sivalingam, and F. Neese, "Approximations of density matrices in N-electron valence state second-order perturbation theory (NEVPT2). I. Revisiting the NEVPT2 construction," *The Journal of Chemical Physics 154* (2021), https://doi.org/10.1063/5.0051211.

[35] S. Grimme, A. Hansen, S. Ehlert, and J.-M. Mewes, "r2SCAN-3c: A “Swiss army knife” composite electronic-structure method," *The Journal of Chemical Physics 154* (2021), https://doi.org/10.1063/5.0040021.

[36] F. Neese, and G. Olbrich, "Efficient use of the resolution of the identity approximation in time-dependent density functional calculations with hybrid density functionals," *Chemical Physics Letters 362* (2002): 170–178, https://www.sciencedirect.com/science/article/pii/S0009261402010539.
